# Phylogenomic Analysis of “Red” Genes from Two Divergent Species of the “Green” Secondary Phototrophs, the Chlorarachniophytes, Suggests Multiple Horizontal Gene Transfers from the Red Lineage before the Divergence of Extant Chlorarachniophytes

**DOI:** 10.1371/journal.pone.0101158

**Published:** 2014-06-27

**Authors:** Yi Yang, Motomichi Matsuzaki, Fumio Takahashi, Lei Qu, Hisayoshi Nozaki

**Affiliations:** 1 Department of Biological Sciences, Graduate School of Science, University of Tokyo, Bunkyo, Tokyo, Japan; 2 Department of Biomedical Chemistry, Graduate School of Medicine, University of Tokyo, Bunkyo, Tokyo, Japan; 3 College of Life Sciences, Ritsumeikan University, Kusatsu, Shiga, Japan; 4 JST, PRESTO, Kawaguchi, Saitama, Japan; 5 School of Computer Science, Fudan University, Shanghai, P. R. China; University of Connecticut, United States of America

## Abstract

The plastids of chlorarachniophytes were derived from an ancestral green alga via secondary endosymbiosis. Thus, genes from the “green” lineage via secondary endosymbiotic gene transfer (EGT) are expected in the nuclear genomes of the Chlorarachniophyta. However, several recent studies have revealed the presence of “red” genes in their nuclear genomes. To elucidate the origin of such “red” genes in chlorarachniophyte nuclear genomes, we carried out exhaustive single-gene phylogenetic analyses, including two operational taxonomic units (OTUs) that represent two divergent sister lineages of the Chlorarachniophyta, *Amorphochlora amoeboformis* ( = *Lotharella amoeboformis*; based on RNA sequences newly determined here) and *Bigelowiella natans* (based on the published genome sequence). We identified 10 genes of cyanobacterial origin, phylogenetic analysis of which showed the chlorarachniophytes to branch with the red lineage (red algae and/or red algal secondary or tertiary plastid-containing eukaryotes). Of the 10 genes, 7 demonstrated robust monophyly of the two chlorarachniophyte OTUs. Thus, the common ancestor of the extant chlorarachniophytes likely experienced multiple horizontal gene transfers from the red lineage. Because 4 of the 10 genes are obviously photosynthesis- and/or plastid-related, and almost all of the eukaryotic OTUs in the 10 trees possess plastids, such red genes most likely originated directly from photosynthetic eukaryotes. This situation could be explained by a possible cryptic endosymbiosis of a red algal plastid before the secondary endosymbiosis of the green algal plastid, or a long-term feeding on a single (or multiple closely related) red algal plastid-containing eukaryote(s) after the green secondary endosymbiosis.

## Introduction

Approximately one billion years ago, an ancient cyanobacterium was engulfed by a heterotrophic eukaryote. A primary plastid is assumed to have evolved directly from the uptake of this cyanobacterium [1], [2], [3]. Thus acquiring photosynthetic activity, the eukaryotic host then diversified into the extant members of the supergroup Archaeplastida, which consists of the Chloroplastida (green algae and land plants), Rhodophyceae (red algae), and Glaucophyta [4]. Recent studies suggest that primary endosymbiosis might have also occurred in another lineage: the cercozoan amoeba *Paulinella chromatophora* [5]. The red and green algal ancestors were then involved in secondary endosymbioses, becoming complex secondary plastids. All of the eukaryotes with red algal-derived secondary and tertiary plastids (hereafter, “CASH lineage” [6]; cryptophytes, alveolates, stramenopiles [Heterokontophyta], and haptophytes) were assigned to the supergroup Chromalveolata in 2005 [4]. However, the number of red secondary endosymbioses has been debated; successive endosymbioses of red algal plastids in the CASH lineage, as a model, is generally growing in popularity [6], [7], [8], [9]. In contrast, green secondary plastids are found in two separate lineages: chlorarachniophytes (supergroup Rhizaria) and euglenophytes (supergroup Excavata) [9]. Recent studies suggest that the green secondary plastids in these lineages may originate from two independent endosymbioses of green algae [10], [11]. Multigene phylogenetic analyses of various eukaryotic lineages has demonstrated that stramenopiles, alveolates, and Rhizaria form a monophyletic group [8], [9], [12], [13], [14] that was very recently reclassified as the supergroup “SAR” (Stramenopiles, Alveolates, and Rhizaria) [15].

During the endosymbiotic process of engulfing, reducing, and integrating plastid ancestors, endosymbiotic gene transfer (EGT) occurred and a bulk of the endosymbiont genes were transferred to the host genomes to remain and function as host nuclear genes; these genes may be relics of past endosymbiotic events that can now be traced back [16], [17]. EGT is a special case of horizontal gene transfer (HGT).

The Chlorarachniophyta is a relatively small algal division inhabiting marine environments. They are rhizarian amoeboflagellates that possess green plastids; their amoeboid single cells often have branching cytoplasmic extensions called reticulopodia. The fact that this algal group has four layers of plastid membranes and a nucleomorph (or endosymbiotically derived eukaryotic nucleus) indicates that the plastids were derived from secondary endosymbiosis of a green algal ancestor [18], [19], [20], [21]. Phylogenetic analyses and pigment composition also point to the green algal origin of the chlorarachniophyte plastids [21], [22], [23], [24]. Thus, genes of green algal ancestry (i.e., genes of the “green lineage”) are expected to reside in the nuclear genomes of the chlorarachniophytes, having been transferred from the nucleomorph to the secondary host nucleus via EGT. However, several recent studies have shown presence of “red” genes of cyanobacterial origin in their nuclear genomes [25], [26], [27]. [28].

In 2003 Archibald et al. [25] showed that eight genes of the chlorarachniophyte *Bigelowiella natans* were derived from red algae or red secondary plastid-bearing algae. More recently, Curtis et al. [28] identified 45 red algal-type genes in the nuclear genome sequence of *B. natans*. However, Curtis et al. [28] discussed that their results have to be treated with caution, and that the testing of hypotheses of possible biological explanations for the diversity of algal nuclear genes seen in *B. natans*, such as the relative contributions of EGT versus HGT, cannot currently be carried out without careful consideration of taxon sampling and methodological artifacts. The precise origins of these red genes were not resolved, in part because their datasets typically included only one operational taxonomic unit (OTU) (*B. natans*) from the Chlorarachniophyta. Genes from more remote chlorarachniophytes are needed to determine whether these red genes originate from the common ancestor or the recent lineage of the chlorarachniophytes.

To expand the diversity of the chlorarachniophyte lineage used in these phylogenetic analyses we chose the chlorarachniophyte species *Amorphochlora amoebiformis* as an additional OTU, because *A. amoebiformis* and *B. natans* belong to two sister, basally divergent lineages of the Chlorarachniophyta [32]. We obtained transcriptome data from *A. amoebiformis* by next-generation sequencing and combined it with the *B. natans* nuclear genome data. To extract more “red” genes from the Chlorarachniophyta, we established another original pipeline and manually checked as many positive outputs as possible. Based on this pipeline and the red genes extracted by Curtis et al. [28], a total of 10 “red” genes of cyanobacterial origin were found from the chlorarachniophyte lineage.

## Materials and Methods

### Strain and culture conditions

*Amorphochlora amoebiformis* CCMP2058 (designated as *Lotharella amoeboformis*) was obtained from NCMA (Provasoli-Guillard National Center for Marine Algae and Microbiota; https://ncma.bigelow.org/) and cultured in L1 medium [33] in which the natural seawater was replaced with Daigo’s artificial seawater SP (Nihon Pharmaceutical Co. Ltd., Tokyo, Japan). The cultures were grown at 20°C with a 14-h light: 10-h dark cycle. For RNA extraction, 4 L of culture, grown in two flasks containing 2 L each, was cultivated for a period of 57 days.

### RNA extraction

Cells of *A. amoebiformis* CCMP2058 were ruptured manually using quartzone sand in liquid nitrogen for 10 min, and RNA was subsequently extracted using the SV Total RNA Isolation System (Promega, Madison, WI, USA). The quantity of total RNA was measured with a NanoDrop 2000 UV-Vis Spectrophotometer (Thermo Scientific, Wilmington, DE, USA) and a Qubit 2.0 Fluorometer (Life Technologies, Carlsbad, CA, USA), until the quantity reached 300 µg with a concentration of 6 µg/µl. The extracted total RNA was then sent to Takara Bio Inc. (Otsu, Shiga, Japan) for further processing, including poly(A) purification and GS FLX+ (Roche Applied Science, Mannheim, Germany) analysis (http://catalog.takara-bio.co.jp/jutaku/basic_info.asp?unitid=U100005162).

### Transcriptome data assembly

The GS FLX+ output FASTA data provided by Takara Bio Inc., containing all 197,073 single reads, were assembled using Trinity (http://trinityrnaseq.sourceforge.net/) [34] on a 2× quad-core Xeon E5-2650 (2.00 GHz, Sandy Bridge-EP) platform (Intel Corporation, Santa Clara, CA, USA). The resulting 11,669 mRNA-derived contigs were translated in both directions to form 23,338 amino acid sequences (with the longest coding sequences among the three frames in each direction), which were subsequently formatted for analysis using local BLASTP.

### Phylogenetic methods

The predicted 21,708 amino acid sequences available from the *B. natans* nuclear genome data [http://genome.jgi-psf.org/pages/dynamicOrganismDownload.jsf?organism=rhizaria] were used as queries for BLASTP (http://blast.ncbi.nlm.nih.gov/Blast.cgi). The BLASTP was carried out against the National Center for Biotechnology Information database (NCBI; http://www.ncbi.nlm.nih.gov/) and local databases that were retrieved from NCBI (expressed sequence tag [EST] data), the DOE Joint Genome Institute (JGI; http://www.jgi.doe.gov/), several unpublished datasets, and our *A. amoebiformis* sequences that were prepared as described above (Table S1). Multiple sequence alignments were generated using Muscle (v3.7 by Robert C. Edgar, http://www.drive5.com/muscle) [35], [36]. We limited the local databases used in the first round (group A in Table S2), as several EST databases resorted to stretching the length of alignment gaps when the quality of their sequences was low. Meanwhile, we used a trimming script to exclude mainly sequences with more than 15% gaps in each alignment. When the FASTA data output contained less than four sequences, the trimming process was redone using an alternative trimming option, which preserved those with less than 70% gaps, considering the “gap-stretch effect” of a rough local BLAST database. Redundant OTUs with the same specific name were also excluded automatically. ‘First-round’ RaxML (7.2.7) [37] phylogenetic analyses were carried out with the WAG+Г4 model as a fast filter (which ignored bootstrap values) to remove trees containing less than three cyanobacterial OTUs and those with eukaryote genes that did appear to show plastid EGT (with basally positioned cyanobacterial OTUs). Results that passed the first-round filter were checked manually for tree topology supporting the cyanobacterial origin of eukaryote genes. All possible plastid EGT queries were searched in BLASTP once more with an extensive local BLAST database (group B in Table S2). According to alignments and tree topologies, long branched OTUs were excluded manually. As the final outputs of second-round phylogenetic analyses, all RaxML analyses were repeated with 1000 replications of bootstrap analysis. Analyses based on PhyloBayes 3.3 (http://www.atgc-montpellier.fr/phylobayes/) [38], [39], [40] were also carried out with the WAG+Г4 model, and the “good run” option was used.

Almost all of the alveolate OTUs were automatically removed during our gene-mining process via BLAST due to their divergent or long-branched sequences. However, alveolates belong to the SAR supergroup, along with chlorarachniophytes and stramenopiles [6], [12], [13], [14], [15]. Thus, analyses were also carried out using the single-gene data matrix with additional alveolate OTUs for comparison (“B” series of figures, if present).

Recently, Deschamps and Moreira [41] pointed out problems for automated massive phylogenomic analyses based on the restriction of available genomic data that are unevenly distributed among the tree of eukaryotes. Therefore, original local databases were constructed for the first- and second-round filters and they were added to the NCBI database (see above). Curtis et al.’s supplementary data [28] have been compared and added to evaluate and cross check our results.

In addition, we carried out approximate unbiased tests (AU test) [29] to examine the phylogenetic position of the chlorarachniophyte OTUs, except for one tree in which two chlorarachniophyte OTUs showed separate phylogenetic positions (see Results). We used nine series of the phylogenetic trees by RaxML (without alveolate OTUs), where topologies of all the OTUs, excluding chlorarachniophytes, were fixed. The alignment was used as input data. All possible topologies were generated by re-grafting the branch of chlorarachniophytes, using the in-house ruby script. The pools of topologies were analyzed with the AU test, using the site-wise log-likelihood values calculated with PhyML (ver. 3.0 [30]) (with the WAG model+F+I+Г4). The AU test was conducted using Consel (ver. 0.2 [31]).

## Results

### Gene mining

**Figure 1:**
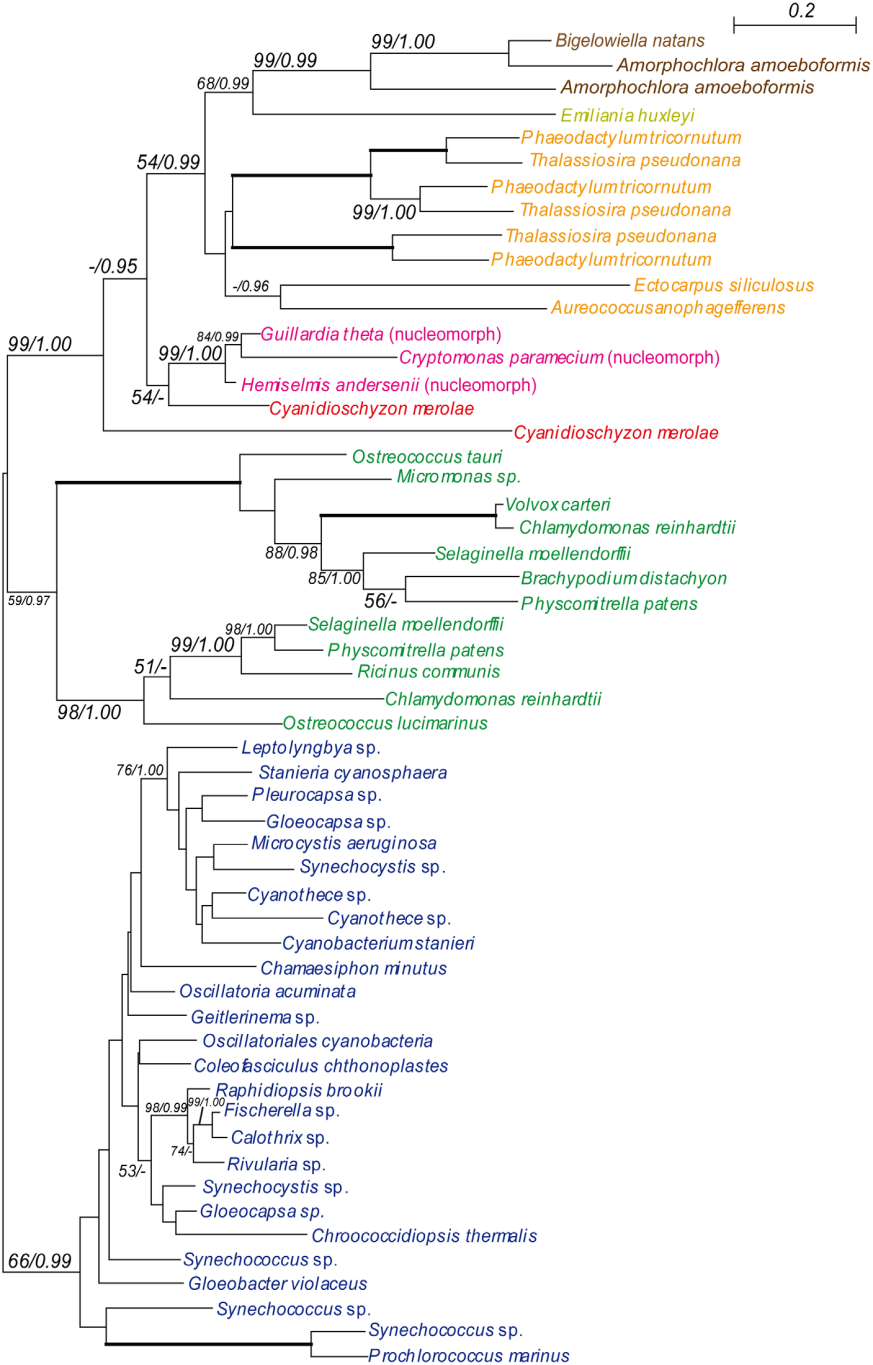
Phylogeny of PDP (FtsZ) showing chlorarachniophyte proteins closely related to red algal plastid-containing eukaryote homologues. The tree was inferred using the RaxML method with the WAG+I+gamma model. Numbers at branches represent support values (bootstrap values ≥50% or posterior probability ≥0.95) from RaxML/PhyloBayes. Thick branches represent RaxML and PhyloBayes support values of 100% and 1.00, respectively. Colors of taxa: dark blue-Cyanobacteria; navy blue-Glaucophyta; green-Chloroplastida; red-Rhodophyceae; pink-Cryptophyta; yellow-Haptophyta; baby pink-Alveolata; orange-stramenopiles; brown-Chlorarachniophyta.

**Figure 2:**
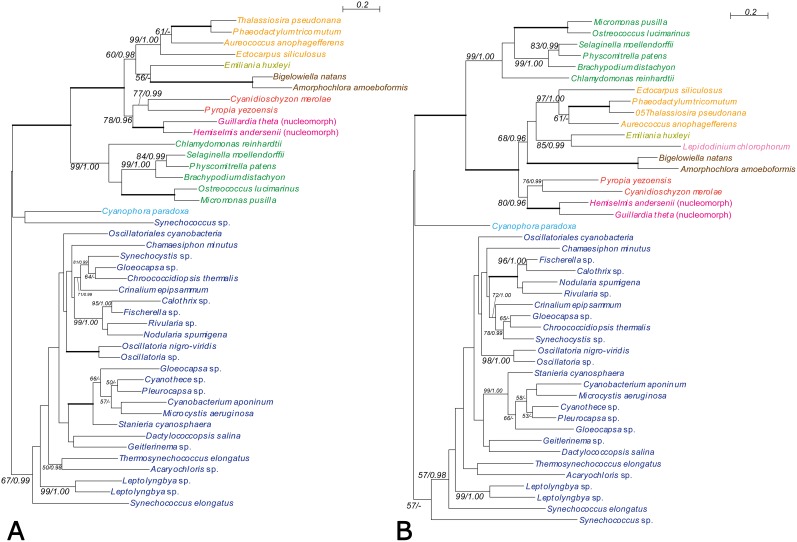
Phylogeny of PS2SAF showing chlorarachniophyte proteins closely related to algal plastid-containing eukaryote homologues. The trees were inferred using the RaxML method with the WAG+I+gamma model. Numbers at branches represent support values (bootstrap values ≥50% or posterior probability ≥0.95) from RaxML/PhyloBayes. Thick branches represent RaxML and PhyloBayes support values of 100% and 1.00, respectively. Colors of taxa: dark blue-Cyanobacteria; navy blue-Glaucophyta; green-Chloroplastida; red-Rhodophyceae; pink-Cryptophyta; yellow-Haptophyta; baby pink-Alveolata; orange-stramenopiles; brown-Chlorarachniophyta. (A) Lacking alveolate OTUs. (B) Containing alveolate OTUs.

**Figure 3:**
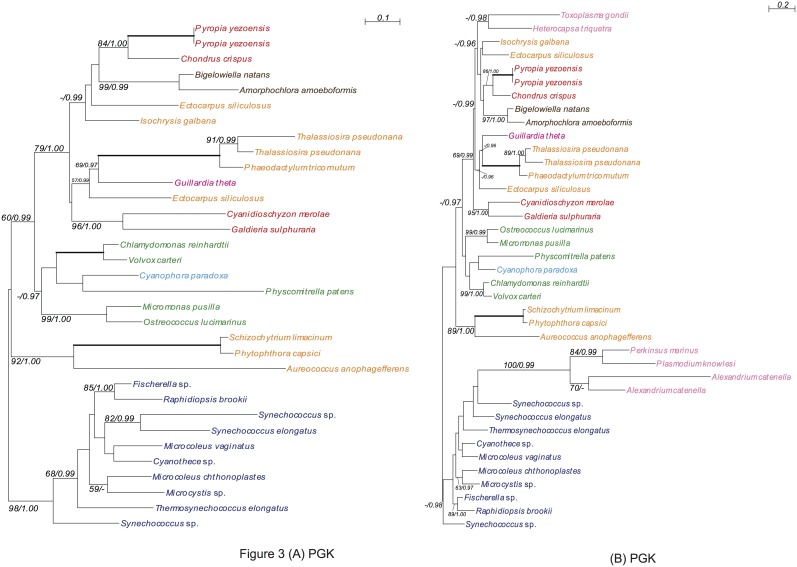
Phylogenies of PGK showing chlorarachniophyte proteins closely related to red algal plastid-containing eukaryote homologues. The trees were inferred using the RaxML method with the WAG+I+gamma model. Numbers at branches represent support values (bootstrap values ≥50% or posterior probability ≥0.95) from RaxML/PhyloBayes. Thick branches represent RaxML and PhyloBayes support values of 100% and 1.00, respectively. Colors of taxa: dark blue-Cyanobacteria; navy blue-Glaucophyta; green-Chloroplastida; red-Rhodophyceae; pink-Cryptophyta; yellow-Haptophyta; baby pink-Alveolata; orange-stramenopiles; brown-Chlorarachniophyta. (A) Lacking alveolate OTUs. (B) Containing alveolate OTUs.

To elucidate the contribution of genes of the red lineage to genome mosaicism in Chlorarachniophyta, we searched the proteome of *B. natans* for proteins showing red algal affiliations. First, we used the 21,708 predicted proteins in *B. natans* available from NCBI as queries to conduct a thorough phylogenomic search. Out of the 21,708 queries, 3,436 proteins had more than 10 OTUs with which to construct phylogenetic trees, and their affinities were examined in an automated fashion using a ruby script. Less than half (1,551) of the 3,436 proteins were categorized as showing a chlorarachniophyte phylogenetic affiliation with stramenopiles/alveolates, red algae, or Chloroplastida. Furthermore, only a small portion of these (259 candidates) had more than two cyanobacterial OTUs in their phylogenetic trees, which is the minimum requirement for a cyanobacterial origin in eukaryote OTUs. Approximately half (129) of the 259 eukaryotic genes of possible cyanobacterial origin showed affinities of the chlorarachniophyte OTUs with Chloroplastida. The remainder might include genes that show a “red lineage” affinity for the chlorarachniophyte homolog. After further refinement of our sampling sequence pool and the sequence pool from the supplementary data of Curtis et al. [27], we selected 6 and 7 cyanobacteria-type hits, respectively, which ultimately resulted in a total of 10 genes that were likely representative of almost all eukaryote OTUs originating from primary plastid EGT and chlorarachniophytes positioned in the red lineage (within, or sister to, the red algae and/or CASH lineages) (Figure S1, File S1). These 10 cyanobacterial gene trees (without alveolate OTUs) were supported by bootstrap values (BV) ≥75% and posterior probability (PP) ≥0.95 for the affiliation of the chlorarachniophytes with the red lineage (Figures 1–3, Figures S2–S8 in File S2, Table S2). Our in-house ruby scripts for gene mining and the amino acid alignments and tree topologies of the 10 genes are available from GtHub (https://github.com/djmyabbay).

Among the 10 genes, genes encoding an ATP binding cassette transporter (ABC), an mRNA binding protein (RNABP), and a geranylgeranyl reductase (GGR), were identified by both pipelines (the present study and Curtis et al. [28]). Three other queries, including one putative membrane protein (PMP), one hypothetical protein (HP), and a phosphoribulokinase (PRK), were found only by our mining pipeline (Figure S1).

### Single-gene phylogenetic analyses

Seven of the ten trees showed robust monophyly of *B. natans* and *A. amoeboformis* (representing the two divergent sister lineages of the Chlorarachniophyta) (with BV ≥97% and PP ≥0.99). The GGR tree exhibited moderate monophyly of the chlorarachniophytes (with 71–73% BV and 0.99 PP), whereas in the other two trees, one showed separation between *B. natans* and *A. amoeboformis* and the other lacked an *A. amoeboformis* sequence (Table S2). Four of the eight trees with chlorarachniophyte monophyly showed affinity of the chlorarachniophytes with the CASH lineage (CASH type), although the support values were weak to moderate (with 54–76% BV and 0.96–0.99 PP), even in analyses without alveolate OTUs. Chlorarachniophyte PRK genes indicated origins directly from a red algal ancestor, as reported previously [27] (Red type). In the remaining three trees showing chlorarachniophyte monophyly, the phylogenetic position of the chlorarachniophytes within the red lineage was ambiguous (Ambiguous type).

#### CASH-type trees

Four genes of CASH lineage encoded plastid-targeted proteins that were directly or indirectly related to photosynthesis or plastid functions: GGR, RNABP, plastid division protein FtsZ (PDP, filamenting temperature-sensitive mutant Z), and photosystem II stability assembly (PS2SAF). FtsZ is a prokaryotic homologue of the eukaryotic protein tubulin and can be considered a functional housekeeping gene of plastids (plastid division) [42], [43], [44]. Figure 1 shows the robust monophyly of the *B. natans* and *A. amoeboformis* FtsZ proteins (with 99% BV and 0.99 PP). Chlorarachniophytes, stramenopiles, and the haptophyte *Emiliania* formed a clade (with 54% BV and 0.99 PP), to which the clade composed of three species of cryptophytes (red algal nucleomorphs) and the red alga *Cyanidioschyzon* was basal. In contrast, PS2SAF is one of the four major multi-subunit protein complexes of the thylakoid membrane of oxygenic photosynthetic organisms. PS2SAF is essential for photosystem II (PSII) biogenesis and required for assembly of an early intermediate in PSII assembly that includes D2 (psbD) and cytochrome b559, and it has been suggested to be required for chlorophyll *a* binding [45], [46]. Phylogenetic analysis of PS2SAF revealed a robust chlorarachniophyte clade (with 100% BV and 1.00 PP) (Figure 2). As with FtsZ, the chlorarachniophytes, stramenopiles, and the haptophyte *Emiliania* constituted a clade (with 60–68% BV and 0.96–0.98 PP) from which two red algae and two cryptophytes were separated. Although the remaining two genes, GGR and RNABP, showed affinity of the chlorarachniophytes with the CASH lineage (with BV ≥60% and PP ≥0.96), the addition of alveolate OTUs lowered the branch support (below 50% BV and 0.95 PP) in the RNABP tree (Figures S3, S5 in File S2).

#### Red-type trees

In the tree of PRK sequences, the chlorarachniophytes, including *A. amoebiformis* sequences, formed a robust clade that was closely related to the red algae [27] (Figure S8 in File S2). As in our previous study [27], this tree topology may indicate that the PRK genes were transferred directly from a red algal ancestor to the common ancestor of the extant chlorarachniophytes. However, OTUs from secondary/tertiary eukaryotes with red algal plastids (CASH lineage) were separated from the red algae. This suggests that the PRK genes of CASH lineage might have experienced a gene replacement after the typical secondary/tertiary EGT from the red algal plastid-containing eukaryotes [9]. A similar separation between the CASH lineage and the lineage composed of red algae and chlorarachniophytes was present in the plastid-targeted sedoheptulose–bisphosphatase (SBP) tree [27] (Figure S9 in File S2).

#### Ambiguous-type trees

Due to the limited number of available sequences, the phylogenetic position of the chlorarachniophyte clade within the red lineage was ambiguous in the three genes: ribosomal protein rps22 (RPS22), hypothetical protein Y (HP), and phosphoglycerate kinase (PGK). For example, PGK is present in all living organisms as one of the two ATP-generating enzymes in glycolysis. In the gluconeogenic pathway, PGK catalyzes the reversible transfer of a phosphate group from 1,3-bisphosphoglycerate to ADP, producing 3-phosphoglycerate and ATP [47], [48], [49]. Chlorarachniophyte PGK gene sequences were robustly monophyletic (with 97–99% BV and 0.99 PP). Chlorarachniophytes, red algae, and red algal secondary/tertiary algae formed a clade with 79% BV and 1.00 PP (Figure 3A). However, for all three genes, no statistical support was obtained regarding the phylogenetic position of the chlorarachniophytes within the red lineage (Figure 3, Figures S4, S7 in File S2).

#### Other trees

Two protein trees did not demonstrate monophyly of the chlorarachniophytes. The ABC protein contained only a single chlorarachniophyte OTU (*B. natans*), and the putative membrane protein (PMP) demonstrated a phylogenetic split between *B. natans* and *A. amoeboformis*. Gene duplications or replacements may have resulted in the split in chlorarachniophyte PMP genes portrayed by the three separate lineages of diatoms (each including a *Thalassiosira* OTU) (Figures S2, S6 in File S2).

### AU tests

Based on our AU tests of nine trees (Figures S10–S18 in File S3), only RPS22 rejected the possibility that the chlorarachniophyte lineage is positioned within the Chloroplastida (excluding the land plants) with p≤0.05 (Figure S15 in File S3). However, the possibility that the chlorarachniophyte lineage is positioned in the basal lineage of the UTC group (origin of the secondary plastids of the chlorarachniophytes [9], [10]; including *Chlamydomonas*, *Volvox*, and *Chlorella*) was rejected by PS2SAF, RNABP, and PRK. For the other trees, our tests did not reject the possibilities, possibly due to the limited information of the single-gene trees.

## Discussion

Based on the extensive analysis of single-gene trees using OTUs from two evolutionarily distinct chlorarachniophytes, we identified 10 genes of cyanobacterial origin that supported an affiliation of chlorarachniophytes with the red algae and/or CASH lineage. For four of the nine trees, our AU test rejected the possibility that the chlorarachniophyte genes originated from secondary endosymbiosis of the extant chlorarachniophyte green plastids (Figures S10–S18 in File S3). Therefore, although these genes are of cyanobacterial origin, they do not likely originate from the green algal ancestor of the secondary plastids in the extant chlorarachniophytes; they most likely originate from the red lineage. Of the 10 single-gene trees, seven demonstrated robust monophyly of the two chlorarachniophyte OTUs, and the GGR tree showed moderate monophyly of the chlorarachniophytes (Table S2). Because the two chlorarachniophyte OTUs represent two sister (basally divergent) lineages of the Chlorarachniophyta [32], multiple HGTs from the red lineage likely occurred in the common ancestor of the extant chlorarachniophytes.

Four of the nine gene trees (CASH type trees; GGR, RNABP, PDP and PS2SAF) showed weak to moderate statistical support for the affiliation of the chlorarachniophyte homologues with those of the CASH lineage (Figures 1, 2, Figures S3, S5 in File S2), whereas three other gene trees were ambiguous (Ambiguous type; RPS22, HP and PGK). Although the chlorarachniophyte PRK genes apparently originated directly from a red algal ancestor (Figure S8 in File S2) as reported previously [27], the genes from the CASH lineage did not belong to the red algal lineage. Therefore, PRK genes of the CASH lineage may have experienced gene replacement after the typical secondary EGT scenario from the red algal ancestor. Thus, the apparent affiliation of chlorarachniophyte PRK homologues with those from red algae (Figure S8 in File S2) [27] may be the result of the removal of CASH-lineage PRK genes from the red lineage by such a gene replacement. A similar situation is possible with the red algal and chlorarachniophyte SBP homologues (Figure S9 in File S2) [27]. Alternatively, the ancestor of the chlorarachniophyte PRK and/or SBP genes may have experienced a chance HGT directly from the red algae lineage. Therefore, although the statistical support for affiliation between chlorarachniophyte OTUs and the CASH lineage was not robust in the four CASH type trees, the CASH lineage may be the most probable origin of the red genes that are believed to have invaded the nuclear genome of the common ancestor of extant Chlorarachniophyta via HGT.

Among the 10 genes, 4 (PDP, GGR, PS2SAF and PRK) were obviously photosynthesis- and/or plastid-related [27], [41], [42], [43], [44], [45], [46], [50]. Furthermore, all eukaryotic OTUs in the ten trees possessed plastids, except for several plastid-lacking stramenopile and excavate taxa in the PGK and RNABP trees (Figure 3, Figure S5 in File S2). Thus, those 10 genes most likely originated directly from photosynthetic eukaryotes. As discussed above, the multiple HGTs likely took place in the common ancestor of the extant chlorarachniophytes, most likely from a red algal plastid-containing eukaryote. Multiple HGTs directly from photosynthetic eukaryotes may be explained by three alternative hypotheses: 1) A cryptic endosymbiosis of a red algal plastid-containing eukaryote in the common ancestor of the extant chlorarachniophytes, 2) Multiple HGTs from a long-term feeding on a single (or multiple closely related) red algal plastid-containing eukaryote(s), or 3) Vertical gene transmission following HGTs from the red alga to the common ancestor of SAR.

Under the first hypothesis, the ancestor of the chlorarachniophytes might have harbored a red-algal plastid prior to the secondary endosymbiosis that gave rise to the green algal plastid that currently exists. Subsequently, the secondary endosymbiosis of the green alga might have resulted in the discarding of such pre-existing red-algal plastids by the ancestor of the chlorarachniophytes. Thus, the origin of the green secondary plastids of extant chlorarachniophytes would represent a replacement of the pre-existing red algal-plastid. This scenario considers that the pre-existing red algal-plastid might have originated from secondary, tertiary, or quaternary endosymbiosis of the cryptic red plastid from the ancestral red lineage [6]. During the proposed cryptic endosymbiosis, EGT could have occurred, resulting in nuclear-encoded, plastid-targeted genes of red lineage. Then, the majority of such red lineage genes could have been replaced by green genes via the secondary EGT of the green plastid of the extant chlorarachniophytes. However, the rest of the red lineage genes in the host nucleus might have been retained, not affected by the green EGT gene replacement, and we now identify them as red lineage genes in the chlorarachniophyte nuclear genome.

The second hypothesis considers that the multiple HGTs from the red lineage might have occurred after the common ancestor of the extant chlorarachniophytes established its green secondary plastid. The ancestor with a green secondary plastid could have experienced a long period in which it fed on a single (or multiple closely related) red algal plastid-containing eukaryote(s). During the long-term feeding, the ancestors of the red genes resolved in the chlorarachniophyte nuclear genome (Table S2) could have replaced pre-existing green genes via multiple HGTs. This evolutionary scenario requires an explanation as to why the HGTs from the red lineage are concentrated in the common ancestors of the chlorarachniophytes. Probably, the secondary endosymbiosis that gave rise to green plastid was based on active feeding on the eukaryotic cells of the common ancestor of the extant chlorarachniophytes, and such phagotrophy could have been active and prominent immediately after the secondary endosymbiosis. Recently, a very interesting, eukaryote-eating euglenophyte was discovered [51]. This new genus, *Rapaza*, has typical green secondary plastids, but actively engulfs a prasinophyte green alga with its feeding apparatus. Since *Rapaza* is positioned most basally within the euglenophyte lineage [51], it may exhibit a strong phagotrophic activity retained from the ancestral stage of the euglenophytes. However, no such a basal chlorarachniophyte has yet been discovered [32].

The third hypothesis is based on the sister relationship between Rhizaria and stramenopiles-alveolates (SA) that harbor the red secondary or tertiary plastids [15], and on the assumption that the common ancestor of SAR might have harbored secondary or tertiary red plastids. Based on the third hypothesis, the red genes found in the chlorarachniophytes and the red secondary or tertiary plastids in the extant SA might have been vertically transmitted from the common ancestor of SAR. This scenario may be supported by the present suggestion that the CASH lineage (including SA) might be the most probable origin of the red genes in the nuclear genome of the Chlorarachniophyta (see above). Because the red genes of chlorarachniophyte nuclear genomes identified in the present study (Figures 1–3, Figures S2–S8 in File S2, Table S2) most likely originated directly from photosynthetic eukaryotes (see above), the common ancestor of the extant chlorarachniophytes might have retained the red plastid vertically transmitted from the common ancestor of SAR. The red plastid in the common ancestor of the chlorarachniophytes might have been then replaced by the green plastid of the extant Chlorarachniophyta. The red genes found in the chlorarachniophye nuclear genomes might be relics of host genomic contents after the secondary endosymbiosis of the green plastid. However, provided extant lineages in Rhizaria lack plastids except for *Paulinella chromatophora* [5] and chlorarachniophytes that are positioned distally within the rhizarian lineage [52], there must have been multiple losses of red plastids during evolution from the common ancestor of SAR in order to explain the presence of various plastid-lacking basal lineages and the derived photosynthetic lineage Chlorarachniophyta within Rhizaria. Thus, parsimony principles seem to disagree with the multiple losses of red plastids, and vertical gene transmission following HGTs from the red alga to the common ancestor of SAR may not likely explain the presence of “red” nuclear genes in chlorarachniophytes.

## Conclusion

The ancestor of extant chlorarachniophytes most likely experienced multiple HGTs from the red lineage prior to or soon after the secondary endosymbiosis that gave rise to the green plastid of the chlorarachniophytes. A recent study of the plastid-possessing euglenid *Euglena gracilis* and plastid-lacking euglenid *Peranema trichophorum* suggested multiple HGTs from the red lineage in the common ancestor of the euglenids [53]. Likewise, multiple gene transfers from the green lineage were recently suggested in the ancestor of the CASH lineage such as diatoms with red algal plastids [54], [55]. Thus, the actual evolutionary histories of both of the green and red plastid-containing secondary/tertiary phototropic lineages are likely more complex than previously thought.

## Supporting Information

Figure S1 **Red-derived genes of cyanobacterial origin resolved by two pipelines.**
(PDF)

Table S1 **Sequence data used in construction of local-databases for BLASTP in this study.** For groups A and B, see Materials and Methods in the main text.
(XLS)

Table S2 **List of the 10 chlorarachniophyte red-derived genes of cyanobacterial origin resolved in this study.**
(XLS)

File S1 **Amino acid alignments of ten genes extracted based on the present gene mining and** ***SBP*** **(** **Figures 1** **–** **3** **, Figures S2–S9 in File S2).**
(PDF)

File S2 **Phylogenetic trees of ABC, GGR, RPS22, RNABP, PMP, HP, PRK, and SBP sequences (Figures S2–S9).** The trees were inferred using the RaxML method with the WAG+I+gamma model. Numbers at branches represent support values (bootstrap values ≥50% or posterior probability ≥0.95) from RaxML/PhyloBayes. Thick branches represent RaxML and PhyloBayes support values of 100% and 1.00, respectively. Colors of taxa: dark blue-Cyanobacteria; navy blue-Glaucophyta; green-Chloroplastida; red-Rhodophyceae; pink-Cryptophyta; yellow-Haptophyta; light pink-Alveolata; orange-stramenopiles; brown-Chlorarachniophyta; purple-Euglenophyta; black-Fungi; violet-Kinetoplastida. (A) Lacking alveolate OTUs. (B) Containing alveolate OTUs.
(PDF)

File S3 **Results of the AU tests for assessing placement of chlorarachniophyte lineage in nine trees: PDP, PS2SAF, PGK, ABC, GGR, RPS22, RNABP, HP, and PRK (Figures S10–S18).** The trees were inferred using the RaxML method with the WAG+I+gamma model. Branches that were rejected by AU test (p≤0.05) for placement of chlorarachniophyte lineage are indicted with dashed lines. Colors of taxa: dark blue-Cyanobacteria; navy blue-Glaucophyta; green-Chloroplastida; red-Rhodophyceae; pink-Cryptophyta; yellow-Haptophyta; baby pink-Alveolata; orange-stramenopiles; brown-Chlorarachniophyta; purple-Euglenophyta; black-Fungi; violet-Kinetoplastida.
(PDF)
