# Supplementary figures and images for: Phylogenomic Analysis of “Red” Genes from Two Divergent Species of the “Green” Secondary Phototrophs, the Chlorarachniophytes, Suggests Multiple Horizontal Gene Transfers from the Red Lineage before the Divergence of Extant Chlorarachniophytes
Source: PLoS One. 2014 Jun 27;9(6):e101158. doi: 10.1371/journal.pone.0101158 (PMC4074131; doi:10.1371/journal.pone.0101158)

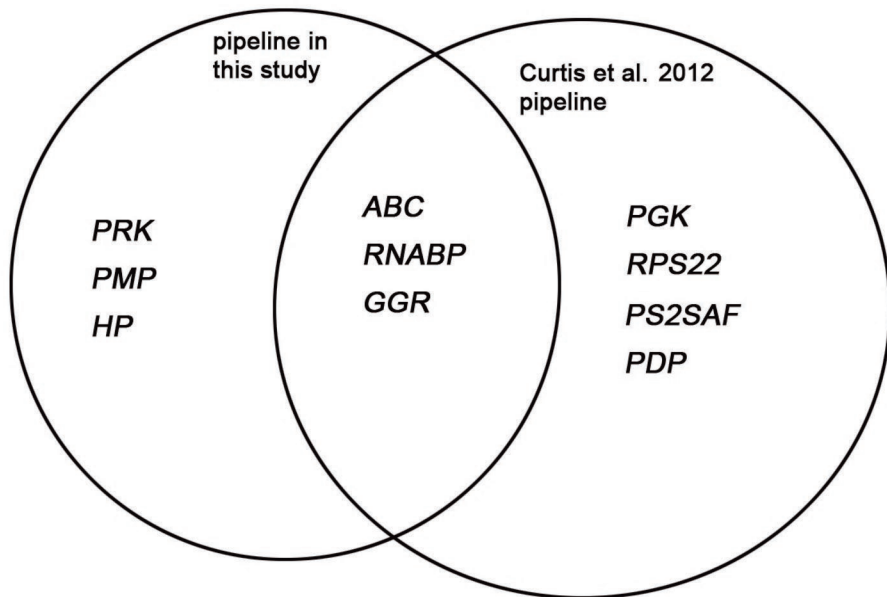

Figure S1

Supplement: Figure S1 — Red-derived genes of cyanobacterial origin resolved by two pipelines. (PDF) [file pone.0101158.s001.pdf]
