# Supplementary material for: Phylogenomic Analysis of “Red” Genes from Two Divergent Species of the “Green” Secondary Phototrophs, the Chlorarachniophytes, Suggests Multiple Horizontal Gene Transfers from the Red Lineage before the Divergence of Extant Chlorarachniophytes
Source: PLoS One. 2014 Jun 27;9(6):e101158. doi: 10.1371/journal.pone.0101158 (PMC4074131; doi:10.1371/journal.pone.0101158)
Supplement: File S1 — Amino acid alignments of ten genes extracted based on the present gene mining and SBP ( Figures 1 – 3 , Figures S2–S9 in File S2). (PDF) [file pone.0101158.s004.pdf]

Alignment: ABC

```
11Micromonas_sp__RCC299_Eukary  FDRKSVGTFV RTLEIWGFVF NFLFRRVALN LKFTKGGTEA KLERTEALAK
23Ostreococcus_tauri_Eukaryota  IERKSIGTIA RSFEIWSFVA AFVFRRVFIG VKASDSSWPA G----ESL--
09Volvox_carteri_f__nagariensi  WSQKTYSVFQ RTFEIWSFAF QFAWRYVLLN QKFTKEGVPE ASARKKELAI
60Chlamydomonas_reinhardtii_Eu  WSQKTYSTFQ RTYEIWSFAF QFAWRYALLG QKVMAALRCT LGACRAQAGT
05Selaginella_moellendorffii_E  WNRKAYSTLQ RSLEIWAFVF SFLFKQWLLG RKFMKGGTEE KMERRKRLAK
02Physcomitrella_patens_subsp_  WNKKTYSTIQ RSLEIWGFVF TFLFKWWLNN QKFMKGGTEL KQEKRKVLAK
03Oryza_sativa_Japonica_Group_  WNRKTYSTIQ RTLEIWGFVF KFIFRSWLNN QKFTRGGTEE KVMRRKVLAK
20Gloeobacter_violaceus_PCC_74  WNRVNYSRTR RVIDIWGFFL LILFYRWWDS KKWTRSGSEE NSRRQRMRAI
35Synechococcus_sp__PCC_7502_B  WSRDNYSPLA RSIDIWRTML TLVLYSWLDA RAWSVGGTPE KQKRRRSRAI
37Synechococcus_elongatus_PCC_  WDRRRYPRWQ RSADIWRFVL LLSFRLWLIQ RPWTFGGSSD RNQRRRKLAS
28Synechococcus_sp__JA-3-3Ab_B  WARENYTRWG RLIDIWRFVL TWLFYLWLDQ QAWSGGQTAE RAKRRRARAI
40Chamaesiphon_minutus_PCC_660  WNRTNYSKRR RTIDIWSFFL TFLSSSWIDG KKWSINGTEA KAARRRKQAI
29Synechococcus_sp__PCC_7335_B  WNKGTYSRPQ RFIDIWSFVL KLLAKRWLYG KAWSGGKTPE KTKRRKDMAV
30Leptolyngbya_sp__PCC_7375_Ba  WNR-RYSRPE RFVDIWSFFL SFLGKRWLYN KSWSAGGSPE KAARRRAQAR
26Microcystis_aeruginosa_PCC_9  WNRESYSINR RRLDIWRFVL TVLYQFWLNG KKWSNGGSQE KAGRRRKQAA
16Stanieria_cyanosphaera_PCC_7  WNRENYSRTR RRIDIWIFVF TLLFKFWRNG KKWSSGGTEE KAQRRKAQAI
27Gloeocapsa_sp__PCC_73106_Bac  WNQENYSRNR RRFDIWVFVS TLLFKLWRNG RKWSEGGSEE KIARRQIQAV
18Cyanothece_sp__PCC_7822_Bact  WNRENYSVNR RRFDIWRFVL LLLLKLTLNG KKWSIGGTEE KTDRRKKQAV
14Pleurocapsa_sp__PCC_7327_Bac  WNQENYSINR RRFDIWRFIL LLLLKLWLNG KKWSKGGEEA KFQRRKVQAA
39Leptolyngbya_sp__PCC_6406_Ba  WSRRRYSRSR RTIDIWSFVL RLLGARWLYG KAWSGGTTPE KAQRRRQQAV
33Geitlerinema_sp__PCC_7407_Ba  WNRERYSRPR RFIDIWTFVW LFLGSLWLNN KAWSRGGTDA KAARRRAQAI
31Crinalium_epipsammum_PCC_933  WNRENYSRHR RFVDIWGFVF TLMTGTWLNG KSWSQGGTEA KAKRRKQQAI
21Moorea_producens_3L_Bacteria  WNQENYSRQR RFIDIWRFVL TLMTGLWLDG KPWSRGGTEQ KAYRRKTQAI
24Calothrix_sp__PCC_6303_Bacte  WNRENYSSRR RFVDIWSFVL TLMFRLWLYG KSWSPGGTEI KSARRRKYAV
22Gloeocapsa_sp__PCC_7428_Bact  WNRENYSSKR RFVDIWLFVI RLLAGFWYNN KSWSSGGTDA KSARRKRQAI
32Chroococcidiopsis_thermalis_  WNRENYSRQR RFVDIWMFVL TLLFGLWLDG KAWSPGGTEA RIARRKKQAI
25Raphidiopsis_brookii_D9_Bact  WNREKYSSKR RFVDIWSFVL TFMFKLWRYN KAWSPGGTEA KAARRYAQAV
17Rivularia_sp__PCC_7116_Bacte  WNRKNYSSRR RFVDIWSFVL TLMLRLWLYN KSWSRGGTET KAARRKWQAI
19Fischerella_sp__JSC-11_Bacte  WNRENYSSRR RFVDIWSFVL TLLYRLWLYN KSWSAGGTEA KAARRKVLAV
34Cyanothece_sp__PCC_7425_Bact  WNRERYSRIR RFIDIWSFVL QFLGGRWLLR QPWSWGGTEA KTARRRAQAI
36Oscillatoriales_cyanobacteri  WNRENYSSQR RFIDIWMFVL ALLYAQWLYN KPWSRGGTEA KQKRRRSQAI
13Volvox_carteri_f__nagariensi  -MQDNYNATQ RNIDTWLFFT LFRSRLWLLD QKWSPGGTEA KGQRAKGLAR
10Arabidopsis_lyrata_subsp__ly  WADENYSSLQ RSIDVWSFVI SLRIRVLFDN AKWAVGGTEE KKSRRRETAS
12Physcomitrella_patens_subsp_  WARDDYSTTQ RQIDVWSFVL TLRARVWFLD SKWTLGGTEE KKARRRSLAV
15Selaginella_moellendorffii_E  WSKENYSKLQ RTIDVWSFVL SLRAKLFLID AKWSVGGSEE KISRRRSIAV
04Phaeodactylum_tricornutum_CC  GKWRKWAILR RSAEIWAYFS SFYIKDRRIS AKYNSGASEE KKAERSQLGK
08Thalassiosira_pseudonana_CCM  -----WATFR RSAEIWFYFS SFYIKDTWIL KNYDSGRSED RKEERGKLGA
01QUERY_Bigel_abc_86002         WQMGFGNKYS RTVEIWAFLI SA---ELKLR KV---KDPEE KSLKRMEYAE
```

```
463Emiliania_huxleyi_Localdb__  WESAQGSALK RTLEVWAFLA SAALKVLKAS K--AKGGPEA ASAAKTAAAE
06Thalassiosira_pseudonana_CCM  ----NASALR RNAEVWKFAL KCVFKALRAR KLTKAGADEA TSASKVEAAT
07Phaeodactylum_tricornutum_CC  WELANSSSIV RNAEVWKFAL ASVFRVLKPR KMKAKGASEE EKKAQTEAAE
283Aureococcus_anophagefferens  --RRRLWAAG RTASIWRTAV SFGWRVARQR RKFADKQSAD AVAARGALAA
83Porphyridium_cruentum_Eukary  ---------- ---------- ---------- ---------- ----------
295Ectocarpus_siliculosus_Loca  WNKGYGSAIS RSVEVWIFGA KMLFKEIKLR KV---EDAAE KSQKRSAIAV
537Pyropia_yezoensis_Localdb__  ---------- ---------- -------RLK R--------- ----------
75Cyanidioschyzon_merolae_Euka  WARGFGSPAS RAVEIWSFAL HQVWMELRLR RV---QDSKQ RAALRRRNAR

                               51
11Micromonas_sp__RCC299_Eukary  WLRLGLLRLG PTFIKIGQQF STRVDVLSKP FIRELEKLQD RVPPFPTPMA
23Ostreococcus_tauri_Eukaryota  -----YFDSG PTFIKVGQQF STRVDVLSPQ FIRELEKLQD RVPPFPTSMA
09Volvox_carteri_f__nagariensi  WLREGLVRLG PTFIKIGQQF STRVDVLSPE FVKELEKLQD NVPPFDREAA
60Chlamydomonas_reinhardtii_Eu  WTRQGVRTRP PRFIKIGQQF STRVDVLSPE FVKELEKLQD NVPPFDREAA
05Selaginella_moellendorffii_E  WLKEGLLRLG PTFIKIGQQF STRSDILPKE YVDELAELQD QVPPFESEVA
02Physcomitrella_patens_subsp_  WLKEGLLRLG PTFIKIGQQF STRVDILAKE YVDELAELQD QVPPFSSETA
03Oryza_sativa_Japonica_Group_  WLKESILRLG PTFIKIGQQF STRVDILPQE YVDQLSELQD QVPPFPSETA
20Gloeobacter_violaceus_PCC_74  WTRETMLELG PTFIKVGQLF STRADLFPKE YIEELSRLQD EVPAFPYEQV
35Synechococcus_sp__PCC_7502_B  WLRESMLQLG PTFIKVGQLF STRADLFPAE YVEELSKLQD RVPAFSYEIA
37Synechococcus_elongatus_PCC_  WIRETCLDLG PTFIKVGQLF STRADLFPAE YVEELSKLQD QVPAFDLEQV
28Synechococcus_sp__JA-3-3Ab_B  WIRETLLHLG PTFIKVGQFF STRADLFPSE YVEELSKLQD RVPAFGYEQV
40Chamaesiphon_minutus_PCC_660  WIRDTFLELG PTFIKLGQLF STRADLFPVE YVEELSKLQD RVPAFSYAQV
29Synechococcus_sp__PCC_7335_B  WIRETCLSLG PTFIKIGQLF STRADLFPIE YVEELAKLQD RVPAFSYEKV
30Leptolyngbya_sp__PCC_7375_Ba  WIRLTFLNLG PTFIKLGQLF STRSDLFPTE YVEELSKLQD QVPAFSYEQV
26Microcystis_aeruginosa_PCC_9  WIRETMLELG PTFIKVGQLF STRADLFPLE YVEELSKLQD QVPAFTYEQA
16Stanieria_cyanosphaera_PCC_7  WIRESLLELG PTFIKVGQLF STRADLFPSE YVEELAKLQD RVPAFAYEQV
27Gloeocapsa_sp__PCC_73106_Bac  WIRKSILELG PTFIKVGQLF STRADLFPKE YVEELSKLQD QVPAFSYEQA
18Cyanothece_sp__PCC_7822_Bact  WIRESLLELG PTFIKVGQLF STRADLFPSE YVEELSKLQD QVPAFTYEQV
14Pleurocapsa_sp__PCC_7327_Bac  WIRESLLELG PTFIKVGQLF STRADLFPAE YVEELAKLQD QVPAFTYEQV
39Leptolyngbya_sp__PCC_6406_Ba  WIRETLLDLG PTFIKVGQLF STRADLFPAE YVEELSKLQD RVPAFSYEQA
33Geitlerinema_sp__PCC_7407_Ba  WVRETFLDLG PTFIKVGQLF STRADIFPIE YVEELSKLQD RVPAFSFEQV
31Crinalium_epipsammum_PCC_933  WIRETLLDLG PTFIKVGQLF STRADLFPSE YVEELTKLQD QVPAFSYEQV
21Moorea_producens_3L_Bacteria  WIRDTFLDLG PTFIKVGQLF STRADLFPSE YVEELSKLQD RVPAFSYEQV
24Calothrix_sp__PCC_6303_Bacte  WIRNTMLDLG PTFIKVGQLF STRADIFPAE YVEELSKLQD RVPAFSYEIV
22Gloeocapsa_sp__PCC_7428_Bact  WIRNKLLDLG PTFIKVGQLF STRADLFPSE YVEELAKLQD KVPAFSYAQV
32Chroococcidiopsis_thermalis_  WIRNTLLDLG PTFIKVGQLF STRADLFPGE YVEELAKLQD RVPAFGYEQV
25Raphidiopsis_brookii_D9_Bact  WVRNTFLDLG PTFIKVGQLF STRADIFPSE YVDELSKLQD RVPAFDYEQV
17Rivularia_sp__PCC_7116_Bacte  WIRNTLLDLG PTFIKVGQLF STRADIFPSE YVEELAKLQD KVPAFSYEQV
19Fischerella_sp__JSC-11_Bacte  WIRNTLLDLG PTFIKVGQLF STRADIFPSE YVEELSKLQD KVPAFTYEQV
34Cyanothece_sp__PCC_7425_Bact  WVRETFLDLG PTFIKVGQLF STRADLFPAE YVEELTKLQD KVPAFAYEQI
```

```
36Oscillatoriales_cyanobacteri  WIRDTLLDLG PTFIKVGQLF STRSDLFPSE YVEELSKLQD KVPAFSYEQA
13Volvox_carteri_f__nagariensi  YLLNSVLQLG PTFIKIGQLS STRSDLLPAE FVEELSTLQD RVPAFAASKA
10Arabidopsis_lyrata_subsp__ly  WLRESVLQLG PTFIKLGQLS STRSDLFPRE FVDELSKLQD RVPAFSPEKA
12Physcomitrella_patens_subsp_  WVREKILQLG PTFIKLGQLS STRSDLFPAE IVEELAKLQD RVPAFSAGKA
15Selaginella_moellendorffii_E  WVKETILQLG PTFIKLGQLF STRSDLFPAE FVEELAKLQD RVPAFSPEKA
04Phaeodactylum_tricornutum_CC  EITQNLLKLG PTFIKVGQLF STRIDIVPKE YIEELKQLQD NVPAFSGDLA
08Thalassiosira_pseudonana_CCM  QLTQNLLKLG PTFIKLGQIF STRIDIVPKE YIESLKLLQD NVPAFSGEKA
01QUERY_Bigel_abc_86002         DLCRGLLRLG PTFIKLGQLL STRRDVLTPE YIKALERLQD DVPGFTGSKA
463Emiliania_huxleyi_Localdb__  FIRDSLFKLG PTFVKLGQVV STRTDVLEKE YIEVLRDLQD NVPGFGGAKA
06Thalassiosira_pseudonana_CCM  FIRDGLLRLG PTFVKLGQVV STRTDVLPVE YTDVLKTLQD DVPGFSGKRA
07Phaeodactylum_tricornutum_CC  FIRDGLLKLG PSFVKLGQVI STRTDVLPPT YTDVLKTLTD DVPGFSGERA
283Aureococcus_anophagefferens  LFRDALLDLG PTFIKFGQLL STRVDVLPPE VIAELATLQN EVPCFSTARA
83Porphyridium_cruentum_Eukary  ---------- ---------- ---------- ---------- ----------
295Ectocarpus_siliculosus_Loca  QLKDGLLRLG PTFIKLGQLL STRIDVVPKE YIKELVMLQD NVPGFPFESA
537Pyropia_yezoensis_Localdb__  ----GLLALG PTFIKLGQLL STRIDVLPRE YIEELVDLQD RVPGFSAADA
75Cyanidioschyzon_merolae_Euka  ALKEGLLRLG PTFIKLGQLA STRVDLFPRE YIEELVSLQD RVPPFNIRVV

                                101
11Micromonas_sp__RCC299_Eukary  KQIIEEELGK PIDEVYTDFQ DEALAAASLG QVHLAKLKGE QVIVKVQRPG
23Ostreococcus_tauri_Eukaryota  KEIIQQELGG PVESFFDDFE DTPLAAASLG QVHRANMKGE QVIIKVQRPG
09Volvox_carteri_f__nagariensi  RSILEASLGK PVEEVFEEFE MEPIAAASLG QVHLARLRGQ RVVVKVQRPG
60Chlamydomonas_reinhardtii_Eu  RAKLEASLGK KVEEVFDEFE SEPIAAASLG QVHLAKLRGQ RVVVKVQRPG
05Selaginella_moellendorffii_E  VSILEEELGC SVDQVFEKFD RDPIAAASLG Q--------- -----IQRPG
02Physcomitrella_patens_subsp_  VQIVEEELGR PVDVIFDRFD RDPIAAASLG QVHRAKLRGK EIVVKVQRPG
03Oryza_sativa_Japonica_Group_  VSIIEEELGA SVNKIFDRFD FEPIAAASLG QVHRACLNGK EVVIKVQRPG
20Gloeobacter_violaceus_PCC_74  VEIVEDQFGK PIPQVFQFFD PTPIAAASLG QVHRAQLHGE DVVVKVQRPG
35Synechococcus_sp__PCC_7502_B  QQITETDLGK SLDQMYQYFD PVPIAAASLG QVHKARLHGE EVVVKVQRPG
37Synechococcus_elongatus_PCC_  QQILSNELGA SPEELFSDFD PVPLAAASLG QVHRARLKGE AVVVKVQRPG
28Synechococcus_sp__JA-3-3Ab_B  AAIVQQELGK PIEQIYSYFD PTPLAAASLG QVHRAKLKGE EVVVKVQRPG
40Chamaesiphon_minutus_PCC_660  EEIIERDLGK KIPELFAYFD PTPLAAASLG QVHLARLYGE DVVVKVQRPG
29Synechococcus_sp__PCC_7335_B  KSTIEDDFGR PLAELYKSFE PVPIAAASLG QVHCAELHGE EVVVKIQRPG
30Leptolyngbya_sp__PCC_7375_Ba  KSIIEDDLGR PIGELYRTFD PIPLAAASLG QVHRAQLHGE EIVVKVQRPG
26Microcystis_aeruginosa_PCC_9  SKIIEVSLGK PLNKLFKSFD PIPLAAASLG QVHRAQLNGE DVVVKVQRPG
16Stanieria_cyanosphaera_PCC_7  AAIIEKDLGK PIGQLFHKFD PTPLAAASLG QVHKAQLHGE EVVVKVQRPG
27Gloeocapsa_sp__PCC_73106_Bac  SAIVQTDLGK SIPELFLNFE PIPLAAASLG QVHKAQLQGE EVVVKIQRPG
18Cyanothece_sp__PCC_7822_Bact  EKIIKADLGK PISKLFRSFD PSPIAAASLG QVHKAQLHGE EVVVKVQRPG
14Pleurocapsa_sp__PCC_7327_Bac  VAIVEADLGK PMNKLFRHFD PVPIAAASLG QVHKAQLHGE EVVVKVQRPG
39Leptolyngbya_sp__PCC_6406_Ba  QEIVEADLGK RIPDLFRSFD PIPLAAASLG QVHRAQLHGE EVVVKVQRPS
33Geitlerinema_sp__PCC_7407_Ba  QRIIEEDLGK PVSNLFQNFE QIPIAAASLG QVHKAQLHGE TVVVKVQRPG
31Crinalium_epipsammum_PCC_933  ERIIEQDLGK KVSKLFRSFD PVPLAAASLG QVHKAILHDE EVVVKVQRPG
```

```
21Moorea_producens_3L_Bacteria  EQIIQEDLGK PIKELFSGFD PIPLAAASLG QVHKAQLRGE EVAIKVQRPG
24Calothrix_sp__PCC_6303_Bacte  EAIVEKELDK KIPELFQDFE QIPLAAASLG QVHKAVLHGE SVVVKVQRPG
22Gloeocapsa_sp__PCC_7428_Bact  ETIIEQELGK KLPELFTSFE PIPIAAASLG QVHKAQLHGE IVVVKVQRPG
32Chroococcidiopsis_thermalis_  ETTVERELGK KIPELFHSFE PIPLAAASLG QVHKAKLRGE EVVVKIQRPG
25Raphidiopsis_brookii_D9_Bact  AKIIEQELGK TIAELFASFE PIPLAAASLG QVHKAELHGE TVVVKVQRPG
17Rivularia_sp__PCC_7116_Bacte  EEIIEYELGK KIPELFASFE PVPLAAASLG QVHKAVLHGE AVVVKIQRPG
19Fischerella_sp__JSC-11_Bacte  ETIIDQELGK KIPELFASFE PIPLAAASLG QVHKAVLRGE AVVVKVQRPG
34Cyanothece_sp__PCC_7425_Bact  EAIILQDFGK TIPELYRSFD PIPLAAASLG QVHKAQLHGE EVVVKVQRPG
36Oscillatoriales_cyanobacteri  KAIIEQDLGK PLQELYRNFD PIPIAAASLG QVHRAQLHGE EVVVKVQRPG
13Volvox_carteri_f__nagariensi  ISIIEKDLGR PISQLFASFD QRPIAAASLG QVHRAVLFGE EVVVKVQRPG
10Arabidopsis_lyrata_subsp__ly  KRFIEAELGA PISVMFKEFE EQPIAAASLG QVHRAVLHGE KVVVKVQRPG
12Physcomitrella_patens_subsp_  MAFIEKELGA PVDALFADFE RQPIAAASLG QVHRAILPGE RVVVKVQRPG
15Selaginella_moellendorffii_E  ELMIENELDT PMQVLFSEFE RQPLAAASLG QVHRAVLRGE QVVVKIQRPG
04Phaeodactylum_tricornutum_CC  VRIIEEELGK PIDQLFDEFD RKSLAAASLG QVHIAR-KDE MLAIKVQRQY
08Thalassiosira_pseudonana_CCM  QEIIEAELGK PINELFDTFN IEPLAAASLG QVHIAT-KDE TYAVKIQRQF
01QUERY_Bigel_abc_86002         KAIIEEDFGK PIDEIFQSFD ETPIAAASLG QVHKAVLKGQ EVAVKIQRQG
463Emiliania_huxleyi_Localdb__  MEIVGRELGA PVEEVFDSFE EEPIAAASLG QVHRAVYKGM PVAVKVQRAG
06Thalassiosira_pseudonana_CCM  KDIVSKELDL PCDDVFTNFS EEPLAAASLG QVHTAMYKGK KVAIKVQRAG
07Phaeodactylum_tricornutum_CC  KEIVEKELGK PVDQVFTDFS AKPLKAASLG QVHTATYKGQ KVAIKVQRSG
283Aureococcus_anophagefferens  VAIVKEELGN KLDEVFASFD ATPLAAASLA QVHRATLKGT EVVVKVQRDG
83Porphyridium_cruentum_Eukary  ---------- ---------- ---------- ---------- ----------
295Ectocarpus_siliculosus_Loca  KRIIEEDLGQ PLEELYETVS EVPLAAASLG QVHLAKIKGE QVAVKVQRAG
537Pyropia_yezoensis_Localdb__  IATVERELGQ PLALLFDTFE EVPLAAASLG QVHRATLDGE ALAVKVQRAG
75Cyanidioschyzon_merolae_Euka  RQIIREDLGR DIEELFDTFE EEPMAAASLG QVHKARINGQ DVAVKVQRAG

                              151
11Micromonas_sp__RCC299_Eukary  LKEIFDIDLK NLRVIAQWLQ KDPKTGAARD WVAIFDETAR CLYDEVDYQN
23Ostreococcus_tauri_Eukaryota  LKEIFDIDLK NLRVIAKWLQ KDPKNGAKRD WVAIFDETAR VLYDEVDYTN
09Volvox_carteri_f__nagariensi  LKDLFDIDLK NIRALAVWLQ KDPKTGAARD WVAIYDECSR ILYQEIDYRL
60Chlamydomonas_reinhardtii_Eu  LKDLFDIDLK NIRALAVWLQ KDPKTGAARD WVAIYDECSR ILYQEIDYRE
05Selaginella_moellendorffii_E  LKALFDIDLK NLRVIAENLQ KDPKSGAKRD WVAIYDECAN VLYQEIDYNR
02Physcomitrella_patens_subsp_  LKALFDIDLK NLRVIAQNLQ KDPKSGAKRD WVAIYDECAN VLYEEIDYTK
03Oryza_sativa_Japonica_Group_  LKELFDIDLK NLRVIAEYLQ KDPKSGAKRD WVAIYDECAS VLYQEIDYTK
20Gloeobacter_violaceus_PCC_74  LEKLFNVDLG ILRGIAQYLQ NHPRYRGGRE WVPIYDECAR ILMQEIDYLN
35Synechococcus_sp__PCC_7502_B  LLKLFAIDLG ILKQIAQYYQ NHPKHGKNRD WLGIYDECQR ILYQEADYLN
37Synechococcus_elongatus_PCC_  LQQLFTVDLA ILRGIAEYFQ RHRRWGQGRD WVGIYEECCR ILWQETDYLR
28Synechococcus_sp__JA-3-3Ab_B  LTRLFTIDLE ICRGIAEFFQ YHTRWGPGRD WIGIYEECRR TLWEEVDYLN
40Chamaesiphon_minutus_PCC_660  LNKLFTIDLE ILRGIAKYFQ NHPKWGGGRD WLGIYDECCR ILWEETDYIS
29Synechococcus_sp__PCC_7335_B  LSRLFEIDLS ILKGIAEYFQ NHKEWGRGRD WLGIYAECCR LLWLEIDFLH
30Leptolyngbya_sp__PCC_7375_Ba  LQRLFQIDLS ILKGIANYFQ SHPEWGRGRD WIGIYEECCR ILWLEIDFLH
```

```
26Microcystis_aeruginosa_PCC_9  LKKLFSIDLA ILKKIAQYFQ NHPKWGKGRD WTGIYEECCK ILWEETDYLN
16Stanieria_cyanosphaera_PCC_7  LKQLFTIDLA ILKQIARYFQ NHPRWGQGKD WIGIYEECCR ILWEETDYIN
27Gloeocapsa_sp__PCC_73106_Bac  LQKLFTIDLA ILKKITQYFQ NHPRWGKGRD WVGIYEECCR ILWEETDYLK
18Cyanothece_sp__PCC_7822_Bact  LKKLFTIDLD ILKRIAQYFQ NHPKWGRGRD WLGIYAECCR ILWLETDYLN
14Pleurocapsa_sp__PCC_7327_Bac  LKKLFTIDLA ILKKIAHYFQ NHPKWGKGRD WLGIYEECCR ILWLETDYLN
39Leptolyngbya_sp__PCC_6406_Ba  LKKLFEIDLS ILKGITHYFQ NHPDWGKGRD WLGIYDECCR ILWEEIDYLN
33Geitlerinema_sp__PCC_7407_Ba  LKKLFSIDLD ILRGIARYFQ NHPDWGRGRD WTGIYEECCR ILWLEIDYLN
31Crinalium_epipsammum_PCC_933  LRKLFTIDLQ ILKGITRYFQ NHPNWGRGRD WLGIYEECCR ILWEEIDYIS
21Moorea_producens_3L_Bacteria  LKKLFTIDLQ ILKGIAYYFQ NHPDWGRGRD WSGIYEECCR ILWQEIDYLN
24Calothrix_sp__PCC_6303_Bacte  LKKLFEIDLE ILKGIARYFQ NHRKWGKGRD WMGIYEECCK ILWQEIDYLG
22Gloeocapsa_sp__PCC_7428_Bact  LRKLFEIDLK ILRGITQYFQ NHPKWGRGRD WIGIYEECCR ILWEEIEYIN
32Chroococcidiopsis_thermalis_  LKKLFEIDLA ILKGITRYFQ NHPDWGRGRD WLGIYEECCR ILWEEIDYLN
25Raphidiopsis_brookii_D9_Bact  LKKLFEIDLQ ILKGITNYFQ NHPEWGRGRD WMGIYEECCR ILWEEIDYLN
17Rivularia_sp__PCC_7116_Bacte  LKQLFEIDLK ILKGITKYFQ NHPKWGRGRD WVGIYEECCR ILWEEIDYLN
19Fischerella_sp__JSC-11_Bacte  LKKLFEIDLK ILKGIARYFQ NHPKWGRGRD WIGIYEECCR ILWEEIDYLN
34Cyanothece_sp__PCC_7425_Bact  LKQLFRIDLA ILKGITYYFQ NHPDWGPGRD WLGIYEECCR ILYEEIDYLN
36Oscillatoriales_cyanobacteri  LVKLFQIDLA ILKGITRYFQ NHPDWGRGRD WLGIYDECCK ILYEEIDYLN
13Volvox_carteri_f__nagariensi  LKQLFDIDLN NLRILAEQLD K----DENRD FKGIYQECAT VLYQEIDYLN
10Arabidopsis_lyrata_subsp__ly  LKKLFDIDLR NLKLIAEYFQ KSESF--TND WVGIYEECAS ILYKEIDYIN
12Physcomitrella_patens_subsp_  LKRLFDIDLN NLKVIAQYFQ NSESMGPTRD WLGIYEECAT ILYQEIDYLR
15Selaginella_moellendorffii_E  LKELFDIDLS NLKFIAEYFQ KDEMLGPLRD WVGIYDECAT VLYREIDYIN
04Phaeodactylum_tricornutum_CC  LRELFEVDLG QLRQVAVFAD ADLTSGTQRD WVSVFQENQR LLYEEIDYIN
08Thalassiosira_pseudonana_CCM  LRELFDVDLG QLKRLAEFAD ADLTSGTKRS WVSVYFEMKR LLYEEIDYLK
01QUERY_Bigel_abc_86002         LDNLFKSDLV NLRLLAVILD KDPKTGAQRD WVRIYEESAR LLYKEIDYTN
463Emiliania_huxleyi_Localdb__  LRELFDTDLK NLKVLVKLLD KDPKSGADRS YSDIYEESSK LLYEEIDYGL
06Thalassiosira_pseudonana_CCM  LKELFDVDLK NLKKLAELLD KDPKTGADRD WVSIYEESER LLYLEIDYLN
07Phaeodactylum_tricornutum_CC  LKELFDIDLK NLKKLAVLLD KDPKTGADRN WVSIYEESER LLYLEIDYLN
283Aureococcus_anophagefferens  LVEQFDVDCK NIRFLASVAD RDPENGVSSN WRGIADTSEG VLYREIDFLV
83Porphyridium_cruentum_Eukary  ---------- ---------- ---------- ---------- ----------
295Ectocarpus_siliculosus_Loca  LKALFDQDLK NLKLLVKVLD KDPKFGADRD WVSIYEESAK LLYKEIDYIN
537Pyropia_yezoensis_Localdb__  LRELFDVDLK NLRALAGLLD RDPKTGAQRN WLGIFEESAV LLYEEIDYTH
75Cyanidioschyzon_merolae_Euka  LRELFDVDLK NLRLLAQMLD WDPKTGASRN WVEIYNESAR LLYEEIDYEN

                             201
11Micromonas_sp__RCC299_Eukary  EAKNAKDFAA QFAGTDWIKV PRVYDQYTRK RTMCMEYAPA TKINDLEAIQ
23Ostreococcus_tauri_Eukaryota  EAKNAEEFKN QFAGVDWIKV PKIYWEFTKR RTLCMEYAPA TKINDLEGLK
09Volvox_carteri_f__nagariensi  EGKNADRFRE NFADVEWVKV PKVYWEYSGQ EVLVLEYVPG TKINDGPAID
60Chlamydomonas_reinhardtii_Eu  DEREAEEGGE GEAEKKDLSA SSRHHPHPS- ---ILSPLTG VKINDAAKID
05Selaginella_moellendorffii_E  EAANAERFAA NFKDLSYVKV PRIYWKYTTP QVLTMEYVPG IKINKIKALD
02Physcomitrella_patens_subsp_  EATNAERFAE NFKDMPYVKA PAIYREFSTP QVLVMEYVPG IKINRIAALD
```

```
03Oryza_sativa_Japonica_Group_   EAFNAEKFSE NFKNMDYVKV PEILWEYTTP QVLTMEYVPG IKINRIKQLD
20Gloeobacter_violaceus_PCC_74   EGRNADTFRR NFKDSPEICV PRVYWRYSSP RVLTLEYLPG IKISNYEALE
35Synechococcus_sp__PCC_7502_B   EGRNADTFRR NFRSSDRILV PKVYWRYTSK RILTLEYLPG IKISDYSSLE
37Synechococcus_elongatus_PCC_   EGRNADRFRR DFRDCDWLLV PRVYWRYASP RVLTLEYLPG IKISDYTALE
28Synechococcus_sp__JA-3-3Ab_B   EGRNADTFRR NFRDMPQIAV PKVYWRYTSP RLLTLEYLPG IKISDYEALS
40Chamaesiphon_minutus_PCC_660   EGQNADTFRR NFRSQDWVMV PRVYWRYSST RTLTLEYLPG IKISSYEGLE
29Synechococcus_sp__PCC_7335_B   EGRNGDKFRR NFRGVDWVKV PRIYWRYTSP KVLTLEYMPG IKISHYEALE
30Leptolyngbya_sp__PCC_7375_Ba   EGRNADTFRR NFRAIDWVKA PRIYWRYASR RVLTLEYLPG IKISHYESLE
26Microcystis_aeruginosa_PCC_9   EGRNADTFRR NFRGEDWVKV PKVYWRYTSP QVLTLEYLPG IKISHYEALE
16Stanieria_cyanosphaera_PCC_7   EGRNADTFRR NFRQENWVHV PRVYWRYASP RVLTLEYLPG IKISHYEAIE
27Gloeocapsa_sp__PCC_73106_Bac   EGRNADTFRR NFRSKNWVQV PKVYWRYTSP RVLTLEYMPG IKISHYEALE
18Cyanothece_sp__PCC_7822_Bact   EGRNADTFRR NFRAADWVKV PRVYWRYTSP QVLTLEYLPG IKISHYEAIE
14Pleurocapsa_sp__PCC_7327_Bac   EGRNADTFRR NFRTQNWVKV PKVYWRYTSP RVLTLEYVPG IKISHYEALE
39Leptolyngbya_sp__PCC_6406_Ba   EGRNADTFRR NFRGQDWVKV PRVYWRYASP RVLTLEYLPG IKISHYEALE
33Geitlerinema_sp__PCC_7407_Ba   EGRNADHFRR NFRNESWVKV PRVYWRYSSP RTLTLEYLPG IKISHYEALE
31Crinalium_epipsammum_PCC_933   EGSNADTFRR NFREEDWVKV PRVYWRYASP RVLTLEYLPG IKISHYQALE
21Moorea_producens_3L_Bacteria   EGRNADTFRR NFRSYDWVKV PRVYWRYTSS RVLTLEFLPG IKISSYEALE
24Calothrix_sp__PCC_6303_Bacte   EGRNADTFRR NFRGYNWVKV PRVYWRYASS RVLTLEYLPG IKVTQYEALE
22Gloeocapsa_sp__PCC_7428_Bact   EGRNADTFRR NFRAYDWVKV PRVYWRYTSS RVLTLEYVPG IKISHYEALE
32Chroococcidiopsis_thermalis_   EGRNADTFRR NFREYDWVKV PRIYWRYTSP QVLTLEYAPG IKISHYEAIE
25Raphidiopsis_brookii_D9_Bact   EGRNADTFRR NFRSYNWVKV PRVYWRYTTS KIITLEYVPG IKISQYEALE
17Rivularia_sp__PCC_7116_Bacte   EGRNADTFRR NFRAQEWVKV PRVYWRYASP RVLTLEYVPG IKISQYEALE
19Fischerella_sp__JSC-11_Bacte   EGRNADTFRR NFRAYGWVKV PRVYWRYTSS RVLTLEYVPG IKISQYEAIE
34Cyanothece_sp__PCC_7425_Bact   EGQNADTFRR NFREFAWACV PRVYWRYSSP RVLTLEYLPG IKISHYEALE
36Oscillatoriales_cyanobacteri   EGRNADTFRR NFRGESWVQV PRVYWRYASP RVLTLEYLPG IKISHYEALE
13Volvox_carteri_f__nagariensi   EGRNADRFRR NFRDASWARA PKVYWEYCSP RVLVLEYLPG AKISDKARLQ
10Arabidopsis_lyrata_subsp__ly   EAKNADRFRR DFRNINWVRV PLVYWDYSAM KVLTLEYVPG VKINNLDALA
12Physcomitrella_patens_subsp_   EGRNADRFRR DFRKQKWVRV PQVFWDYASQ KVLTLEYVPG IKINDVALLD
15Selaginella_moellendorffii_E   EGRNADKFRR DFRNIKWVKV PKVYWDFTSR KVITLEYLPG IKINDLAALD
04Phaeodactylum_tricornutum_CC   EMNNCNRFRE NFAKFRHIRA PKTYPEFTTD KVMAMEFLPG IKVTDKEKIE
08Thalassiosira_pseudonana_CCM   EIDNCNRFRC NFPKFSHIKA PKTYPEYTTD KVLTMEYCPG IKITDVDRIK
01QUERY_Bigel_abc_86002          EAKNAERFKE NFADVPWIKV PDIYWETTSK RVLTMEYVPS VKINNIEEIE
463Emiliania_huxleyi_Localdb__   EAANCLRFAE SLAGLDYVRV PQVYNEVTTP RVLTLEYVPS FKLTDLPRVE
06Thalassiosira_pseudonana_CCM   EAANCERFAR DF-DIEYVRV PEVYREVSTP RVLTMEFVES FKLTNIEKIE
07Phaeodactylum_tricornutum_CC   EADNTDRFAK DF-GYDWVRI PKVIREVTTP RLLVMEFVES FKLTDIEEIN
283Aureococcus_anophagefferens   ERDAAERFRR AFKPLPWVKV PKTFDEYCTS RVLVMEYVPG TKINDVPALE
83Porphyridium_cruentum_Eukary   ---------- ---------- ---------- ---------- ----------
295Ectocarpus_siliculosus_Loca   EAENAIRFKE NFQDTPWVKV PDVYWNMTSE RVVTMEFVPG VKINNIDEID
537Pyropia_yezoensis_Localdb__    EAANAERMRE NFAGTPWVRI PSVLWERTSE AVLTMEFVQG VKINDYAAME
75Cyanidioschyzon_merolae_Euka   EAQNAREFAD NFGANDWVRV PRILDAYTSK RVLVMEYVKG TKISAVEALD
```

```
                                251
11Micromonas_sp__RCC299_Eukary  KMGVDPDRMA RLAVESYLMQ VLRFGFFHAD PHPGNVAVGR LVIYDYGMMG
23Ostreococcus_tauri_Eukaryota  KIGVDPDRMA RLAVEAYLQQ VLRFGFFHAD PHPGNVAVGR LVVYDYGMMG
09Volvox_carteri_f__nagariensi  RLGLDRKRLA RLSVESYLQQ ILRHGFFHAD PHPGNVAVGR LIYYDFGMMG
60Chlamydomonas_reinhardtii_Eu  ALGLDRQKLA RLSVESYLQQ ILRHGFFHAD PHPGNVAVGR LIYYDFGMMG
05Selaginella_moellendorffii_E  RLGVDRQRLA RYCVESYLEQ ILRHGFFHAD PHPGNIAVGR LIFYDFGMMG
02Physcomitrella_patens_subsp_  ELGVDRKRLA RYAVESYLEQ ILRHGFFHAD PHPGNIAVGR LIFYDFGMMG
03Oryza_sativa_Japonica_Group_  KLGVDRKRLG RYAVESYLEQ ILSHGFFHAD PHPGNIAVGR LIFYDFGMMG
20Gloeobacter_violaceus_PCC_74  AAGLDRRSLA RIGARSYLQQ LLNDGFFHAD PHPGNIAVGA LIFYDFGMMG
35Synechococcus_sp__PCC_7502_B  AAGIDRKILA KLGAESYLRQ LLNDGFFHAD PHPGNLAVGS LIFYDFGMMG
37Synechococcus_elongatus_PCC_  AANLDRQKIS QLNAEAYLRQ VLNHGFFHAD PHPGNLAVGR LIFYDFGMMG
28Synechococcus_sp__JA-3-3Ab_B  AAGLDRKLLA RLGAEAYLRQ LLKDGFFHAD PHPGNIAVGT LIFYDFGMMG
40Chamaesiphon_minutus_PCC_660  AAGLDRQTIA RQSATAYLQQ LLTDGFFHAD PHPGNVAIGA LIFYDFGMMG
29Synechococcus_sp__PCC_7335_B  SAGLDRKRLA RLGAEAYLRQ LLTDGFFHAD PHPGNIAVGA LIFYDFGMMG
30Leptolyngbya_sp__PCC_7375_Ba  AAGLDRQQLA QLGAQAYLHQ LLNDGFFHAD PHPGNIAVGA LIFYDFGMMG
26Microcystis_aeruginosa_PCC_9  AAGLDRKLLA KLGAKAYLIQ LLNNGFFHAD PHPGNIAVGS LIFYDFGMMG
16Stanieria_cyanosphaera_PCC_7  AAGLDRKLLA RLGAKAYLQQ LLNSGFFHAD PHPGNIAVGS LIFYDFGMMG
27Gloeocapsa_sp__PCC_73106_Bac  AAGLDRKRLA RLGAEAYLQQ LLNDGFFHAD PHPGNLAVGS LIFYDFGMMG
18Cyanothece_sp__PCC_7822_Bact  AAGLDRKLLA RLGAKAYLQQ LLNDGFFHAD PHPGNIAVGA LIFYDFGMMG
14Pleurocapsa_sp__PCC_7327_Bac  AAGLDRKLLA KLGAKAYLQQ LLNDGFFHAD PHPGNLAVGA LIFYDFGMMG
39Leptolyngbya_sp__PCC_6406_Ba  ASGLDRKRLA NLGARAYLHQ LLNDGFFHAD PHPGNIAVGA LIFYDFGMMG
33Geitlerinema_sp__PCC_7407_Ba  AAGLDRKLLA QLGARAYLQQ LLNDGFFHAD PHPGNIAVGS LIFYDFGMMG
31Crinalium_epipsammum_PCC_933  AAGIDRKLVA QLGAKAYLHQ LLNDGFFHAD PHPGNIAVGS LIFYDFGMMG
21Moorea_producens_3L_Bacteria  AAGLDRKLIA RLGAEAYLQQ LLNDGFFHAD PHPGNIAVGS LIFYDFGMMG
24Calothrix_sp__PCC_6303_Bacte  AAGLDRKVLA RQSAEAYLHQ LLDSGFFHAD PHPGNIAVGA LIFYDFGMMG
22Gloeocapsa_sp__PCC_7428_Bact  AAGIDRKLVA RQGAEAYLQQ LLNDGFFHAD PHPGNIAVGS LIFYDFGMMG
32Chroococcidiopsis_thermalis_  AAGLDRKLIA RQGAEAYLHQ LLHNGFFHAD PHPGNIAVGS LIFYDFGMMG
25Raphidiopsis_brookii_D9_Bact  AAGVDRKAIA RYGAQAYLHQ LLNNGFFHAD PHPGNLAVGA LIFYDFGMMG
17Rivularia_sp__PCC_7116_Bacte  AAGLDRKVLA RQGAEAYLHQ LLNNGFFHAD PHPGNIAVGA LIFYDFGMMG
19Fischerella_sp__JSC-11_Bacte  AAGLDRKQIA RQGAQAYLLQ LLDNGFFHAD PHPGNIAVGA LIFYDFGMMG
34Cyanothece_sp__PCC_7425_Bact  AAGLDRKELA QLGAKAYLHQ LLNDGFFHAD PHPGNIAVGG LIFYDFGMMG
36Oscillatoriales_cyanobacteri  AAGLDRRRLA QLGAEAYLHQ LLNNGFFHAD PHPGNIAVGA LIFYDFGMMG
13Volvox_carteri_f__nagariensi  AAGLDLDTIA RRATEAYLIQ ILKHGFFHAD PHPGNVSVGD LLFYDFGMMG
10Arabidopsis_lyrata_subsp__ly  ARGFNRSRIA SRAIEAYLIQ ILKTGFFHAD PHPGNLAIES IIYYDFGMMG
12Physcomitrella_patens_subsp_  AGGFDKPKLA RLAIEAYLIQ ILRTGFFHAD PHPGNLAVGS LIYYDFGMMG
15Selaginella_moellendorffii_E  AGSYKRSLIA SRAIEAYLIQ ILKTGFFHAD PHPGNLAVGS LIYYDFGMMG
04Phaeodactylum_tricornutum_CC  QAGLDPIDIS VKMAEGFLEQ LCRHGFFHSD PHPGNVAVAV IIFYDFGMMD
08Thalassiosira_pseudonana_CCM  EEGLDPADIS KKSAEAFLEQ LCRHGFFHCD PHPGNVAVAG LIFYDFGMMD
01QUERY_Bigel_abc_86002         RRGIDR---- ---------- ---------- ---------- --------MD
```

```
463Emiliania_huxleyi_Localdb__  AAGLDPKLLA KRTADAFLTQ ---------- --------GK LVYYDCGMMN
06Thalassiosira_pseudonana_CCM  SLGLDREVLA KRTADAFLRQ IVETGYFHCD PHPGNLCVGN LVYYDFGMMD
07Phaeodactylum_tricornutum_CC  RNGLDRKVIS KRVADAFLRQ IVETGYFHAD PHSGNLCVGN LVYYDYGMMD
283Aureococcus_anophagefferens  KMDVDLPLIS QRLTTSYLEQ LARHGFFHCD PHPGNVAVGR LIYYDFGMME
83Porphyridium_cruentum_Eukary  ---------- ---------- ---------- ---------- ----------
295Ectocarpus_siliculosus_Loca  RRGIDRKLLA KRSAEAYLTQ LCRHGFFHCD PHPGNVACGR LIFYDFGMMD
537Pyropia_yezoensis_Localdb__  AMGVDRTLVA RRAAQSFLTQ LLRHGFFHDD CHPGNLHVGG LIYYDFGMCS
75Cyanidioschyzon_merolae_Euka  ALGLDRKLLA ERLGRFFLEQ TLRHGFIHCD PHPGNIAVGR LLVYDFGMCQ

                               301
11Micromonas_sp__RCC299_Eukary  RIDPKVRSGF LDLFYAVFEK NSDSAVKALG KMGVLVEGGD MTAVKRTADF
23Ostreococcus_tauri_Eukaryota  RIPSTTRDGL LDLFYATYEG QSDSAVKALM KMGVLVDGGD LTAVKRTADF
09Volvox_carteri_f__nagariensi  SLAPEVKSGL LELFYGVYNR DPDRCLEALT TMGVYLPTGD KTAVRRTAEF
60Chlamydomonas_reinhardtii_Eu  SLAPEVKGGL LELFYGVYNR DPDRCLEALT AMGVYVPSGD KTAVRRTAEF
05Selaginella_moellendorffii_E  SISSNIREGL LEAFYGVYEK DADKVLEAMV QMGVLVPTGD MTAVRRTAQF
02Physcomitrella_patens_subsp_  SISPNIREGL LEVFYGVYEK DPDKVLAAMV QMGVLVPSSD LTAVRRTAQF
03Oryza_sativa_Japonica_Group_  SISPNIREGL LEAFYGVYEK DPDKVLQSMI QMGVLVPTGD MTAVRRTAQF
20Gloeobacter_violaceus_PCC_74  HIQPGTKEKL MDTFLGVAQS DADKVIDSLI ELGAIKKSAD RVPIRRSIEF
35Synechococcus_sp__PCC_7502_B  QIQPITKTKL VNVFFGIAQK DAELVIASLI DLGALEISGD IDPVRRSVQY
37Synechococcus_elongatus_PCC_  EIRTDVRSKL MQVFLGIARK DADDIVSALV ELGALLPTGD LGPVRRSVQY
28Synechococcus_sp__JA-3-3Ab_B  RIRPGIKGKL VAMLAAVVAK NADLVVASLV ELGVLVPTAD LAPVRRSVQY
40Chamaesiphon_minutus_PCC_660  RIAPGTKEKL MDTLLAIAGR NADRVVRGLV GLGAIAAEAD TDPIRRSVQY
29Synechococcus_sp__PCC_7335_B  QIQAVTRTRL MDTFFGIAQR DAGLVMKSLV ELGALAQIDD MGPVRRSIQY
30Leptolyngbya_sp__PCC_7375_Ba  QIQPLTRDGL MRTFMGVSQR NADMVMASLI DLGALTETED MGPVRRSIQY
26Microcystis_aeruginosa_PCC_9  QIKTNVREKL MQTLLGIAQK DADRVVTSLV DLGALTANGD MGAVRRSIQY
16Stanieria_cyanosphaera_PCC_7  QIKNNIREKL MDTLFGIAQK NADLVVNSLI EVGALTPTGD LSPVRRSVQY
27Gloeocapsa_sp__PCC_73106_Bac  QIKTDVREKL MQTLFGIAEK NAERVVRSLV ELGALTPIGD MGPVRRSVQF
18Cyanothece_sp__PCC_7822_Bact  KITSNIREDL METLFGIAEK NAERVVNSLI ALGALAPTND MGPVRRSVQY
14Pleurocapsa_sp__PCC_7327_Bac  EIKTNIREKL MQTLFGIAEK NADRVVNSLI DLGALEPTGD MSSVRRSIQF
39Leptolyngbya_sp__PCC_6406_Ba  QVQPATRDRL MVTFMGVAQR DAGLVVASLV DLGALVEVDD MGPVRRSIQY
33Geitlerinema_sp__PCC_7407_Ba  QVRTDIREKL MDTFFGIAQK DAARVVDSLI ALEAIAPTDD IGPVRRSVQY
31Crinalium_epipsammum_PCC_933  TVNPITREKL MDTLFGISQK DADRVVKSLV ELGALAPVDD MGPVRRSVQY
21Moorea_producens_3L_Bacteria  QIKANVREQL METLFGIAQK DGDRVVTSLI ELGALSPVSD MGPVRRSVQY
24Calothrix_sp__PCC_6303_Bacte  QIKSNVRESL METLFGVASK DGDRVLQSMV DLGVIAPTDD MGPVRRSVQY
22Gloeocapsa_sp__PCC_7428_Bact  QIKTGVREQL MKTLFGIAQK DAQQVMDSLV ALGALAPTDD MGPVRRSIQY
32Chroococcidiopsis_thermalis_  RIKANVREQL METLFGIAQK DGGRVVASLV ELGALAPTDD MGPVRRSVQF
25Raphidiopsis_brookii_D9_Bact  IIKSNVREGL METLFGIAQK DGDRVVQSLI NLGAIAPVED MGPVRRSVQY
17Rivularia_sp__PCC_7116_Bacte  RIQSNVREGL MDTLFGVASK DGERVVNSLV NLGALAPTDD MGPVRRSVQY
19Fischerella_sp__JSC-11_Bacte  RIKSNVREGL METLFGIASK DGDRVVQSLI NLGALAPVED MGPVRRSVQY
34Cyanothece_sp__PCC_7425_Bact  RVQPITREKL VRTFMGIARR DGQMVMNALV DLGALAPVED MGPVRRSIQY
```

```
360scillatoriales_cyanobacteri  QVKPVTREKL MQTFFGIANK NADQVIESLI ELGALAPTED MGPVRRSIQF
13Volvox_carteri_f__nagariensi  EIVPDVRTRL LDVFYGVYRK DTDQVLRALV ALQVIKPTGD SLSLRRAINY
10Arabidopsis_lyrata_subsp__ly  EIKTFTRKRL LDLFYSVYEK DAKKVMQNLI DLEALQPTGD LSSVRRSVQF
12Physcomitrella_patens_subsp_  EIKSFTREKL LEMFYAVYEK DARKIIQALI DLGALVPTGD MGSVRRSVQY
15Selaginella_moellendorffii_E  EIKSFTKEKL LELFYAVYEE DASKVIQGLV DLGALVPTGD MGPVKKTIQF
04Phaeodactylum_tricornutum_CC  SFGDVQRKGL VDFFFAVYDA NVKDAMDALE RLGMLRNGID RVAVERVGKD
08Thalassiosira_pseudonana_CCM  EFGAVERKGL VDFFFALYDA DAKDVCNALE RLGMLRKGVD RMSVEKVGQD
01QUERY_Bigel_abc_86002         EFSTGVRKGL VNLIYSVYEN DEVGLCNALE EMGILQPDSD RLSVERISRF
463Emiliania_huxleyi_Localdb__  ELQPNVFSGF KEACGAVFAT NAKRLVDALE LMGVLAKSAD RLAVEKLARY
06Thalassiosira_pseudonana_CCM  ELKPNVRSGF RKFCTALFAE NAKMLVDGVE EAGVLARGAD RLAVEKLARY
07Phaeodactylum_tricornutum_CC  ELSPNVKAGF RKFCTALFAQ NAKELVAGVE QAGVLAKGAD RLAVEKLARY
283Aureococcus_anophagefferens  SIEPDVKKGF VDLVYSLYKN QPITACDALE QMGVLRPGLD RYSIERIATN
83Porphyridium_cruentum_Eukary  ---------- ---------- ---------- ---------- ----------
295Ectocarpus_siliculosus_Loca  EFKPNVRSGL VNLIFSTYEN DPRAVCDALV EMGILKAGSD RISVEKIARS
537Pyropia_yezoensis_Localdb__  SISPSVRQGF INTVFAIYEG TPQDFCDGLA AMGILRPTAD RLSVEKIGRY
75Cyanidioschyzon_merolae_Euka  RLTAKVRRAV VNLVFATYEN DVAAFVDALA EMGVLKQTAD RLTVYRIARY

                               351
11Micromonas_sp__RCC299_Eukary  FLGSFDNRRK QILSNIGEDL LVVSKDQPFR FPAELTFVVR AFSVLDGIGK
230streococcus_tauri_Eukaryota  FLTQFDARRK KILSNIGEDL LVVSKDQPFR FPAEFTFVVR AFSVLDGIGK
09Volvox_carteri_f__nagariensi  FLKGFQERRR QILASIGEDL LLAANDQPFR FPATFTFVVR SFTVLDGIGK
60Chlamydomonas_reinhardtii_Eu  FLKGFQDRRK QILSSIGEDL LLAANDQPFR FPATFTFVVR SFTVLDGIGK
05Selaginella_moellendorffii_E  FLKSFQDRKK LRLAAIGEDL LSISSDQPFR FPATFTFVVR AFSVLDGIGK
02Physcomitrella_patens_subsp_  FLNSFEERKK LRLAAIGEDL LSIGADQPFR FPATFTFVVR AFSVLDGIGK
030ryza_sativa_Japonica_Group_  FLDSFEERKK QRLAAIGEDL LSIAADQPFR FPATFTFVVR AFSVLDGIGK
20Gloeobacter_violaceus_PCC_74  LLTNFVNQQS FNFAELTDDI YEMAYEQPFR FPATFTFVLR AVSTLEGLGK
35Synechococcus_sp__PCC_7502_B  MLDNFMGKAS QSITAISDDL FEVAYAQPFR FPATFTFVLR ALSTLEGLGK
37Synechococcus_elongatus_PCC_  LLDNFLDREG QSISAISDDI YEIAYDQPFR FPATFTFVMR AFSTLEGVGK
28Synechococcus_sp__JA-3-3Ab_B  MLDHFMDKDE ISVMAISEDL YELAYDQPFR FPATFTFVMR ALTTLEGLGK
40Chamaesiphon_minutus_PCC_660  LLDNFLDKES QSIAQITDDL YDIAYGQPFR FPATFTFVMR AFSTIEGVGK
29Synechococcus_sp__PCC_7335_B  ILDNMMDKEE QSVTAISDDL YAVAYDQPFR FPATFTFVMR AFSTLEGVGK
30Leptolyngbya_sp__PCC_7375_Ba  ILDNFMDKEE QSITAISDDL YAVAYDQPFR FPATFTFVMR AFSTLEGVGK
26Microcystis_aeruginosa_PCC_9  MLDNFMDKEE QSVASISDDL YEIAYDQPFR FPATFTFVMR AFSTLEGVGK
16Stanieria_cyanosphaera_PCC_7  MLDNFMDKEE QSIEAISEDL YDIAYGQPFR FPATFTFVMR AFSTLEGVGK
27Gloeocapsa_sp__PCC_73106_Bac  MLDNFMDQEN QSVAAISEDL YEIAYDQPFR FPATFTFVMR AFSTLEGVGK
18Cyanothece_sp__PCC_7822_Bact  MLDNFMDKED QSVAAISEDL YEIAYDQPFR FPATFTFVMR AFSTLEGVGK
14Pleurocapsa_sp__PCC_7327_Bac  MLDNFMDKEE QSIDQISEDL YEIAYDRPFR FPATFTFVMR AFSTLEGVGK
39Leptolyngbya_sp__PCC_6406_Ba  ILDNFMDQEE QSVTAIGDDL YAIAYDQPFR FPATFTFVMR AFSTLEGVGK
33Geitlerinema_sp__PCC_7407_Ba  MLDNFMDQEN QSVSAISEDL YEIAYNNPFR FPATFTFVMR AFSTLEGVGK
31Crinalium_epipsammum_PCC_933  MLDHFMDKEN QSVAQISDDL YEIAYDQPFR FPATFTFVMR AFSTLEGVGK
```

21Moorea_producens_3L_Bacteria MLDNFMDKEE QSVSNISDDL YEIAYGQPFR FPATFTFVMR AFSTLEGVGK
24Calothrix_sp__PCC_6303_Bacte MLDNFMDGEN KSVAAISDDL YEIAYNQPFR FPATFTFVMR AFSTLEGVGR
22Gloeocapsa_sp__PCC_7428_Bact MLDHFMDKEN QSVAAISDDL YEIAYDQPFR FPATFTFVMR AFSTLEGVGK
32Chroococcidiopsis_thermalis_ MLDNFMDKEN QSVSAISDDL YEIAYGQPFR FPATFTFVMR AFSTLEGVGK
25Raphidiopsis_brookii_D9_Bact MLDNFMDKEN QSVATISEDL YEIAYNQPFR FPATFTFVMR AFSTLEGVGK
17Rivularia_sp__PCC_7116_Bacte MLDHFMDKEK QSVASISEDL YEIAYDQPFR FPATFTFVMR AFSTLEGVGK
19Fischerella_sp__JSC-11_Bacte MLDNFMDKEN QSVAAISDDL YEIAYDQPFR FPATFTFVMR AFSTLEGVGK
34Cyanothece_sp__PCC_7425_Bact MLDNFMDQES QSVAQISDDL YDIAYNQPFR FPATFTFVMR AFSTLEGVGK
36Oscillatoriales_cyanobacteri MLDNFMDQEA QSVTEISDDL YEIAYGQPFR FPATFTFVMR AFSTLEGVGK
13Volvox_carteri_f__nagariensi FIENL-SRRQ ETIQAIGEDL FAIALDQPFR FPATFTFVLR AFSTLEGIGK
10Arabidopsis_lyrata_subsp__ly FLDNLLSQQQ QTLAAIGEDL FAISQDQPFR FPSTFTFVIR AFSTLEGIGY
12Physcomitrella_patens_subsp_ FLDNLMDQQA ATFSAIGEDL FAIAVDQPFR FPATFTFVLR AFSTLEGIGN
15Selaginella_moellendorffii_E FLKNLTSQQA TTFTAIGEDL FAIAVDQPFR FPSTFTFVLR AFSTLEGIGY
04Phaeodactylum_tricornutum_CC FIDRFQETTR ERRRKLGEEF LSLNRDSPFV FPPTWTFVFR AFFSIDGIGK
08Thalassiosira_pseudonana_CCM FIDRFQATNR QRRKELGEEF LSMNAESPFV FPPTWTFVLK AFFTLDGIGK
01QUERY_Bigel_abc_86002 FLEDFQKTPE EKKKARAERI STVYDDRE-K KKIVFLFLSY SLRFLEGIGK
463Emiliania_huxleyi_Localdb__ FIRTFKQIAS NIKTTLGADL QALTEQQVFR FPSTFTFIFR AFASVDGIGK
06Thalassiosira_pseudonana_CCM FMRTFKDSGS NIKETVGSDL QTLTENDVFR FPSTFTFIFR SFASIDGIGK
07Phaeodactylum_tricornutum_CC FMRAFKDKSG NIKQTIGTDL QTLTENNVFR FPSTFTFIFR AFASVDGIGK
283Aureococcus_anophagefferens YE------RR ERRAQIGKDL FATQAERPFV FPPKFTFIFR AITTIDGIGK
83Porphyridium_cruentum_Eukary ---------- ---------- ---------- ---------- ---TLEGIGK
295Ectocarpus_siliculosus_Loca FLGEFTNTRK ERRAKLGEDL LSVSGDVPFK FPPTFTFVFR AFTSLDGIGK
537Pyropia_yezoensis_Localdb__ FISAFRSSLT TRLTAIGDEL LAVGEDQPFV FPPVFTFVFR AFTTLEGVGK
75Cyanidioschyzon_merolae_Euka FLKAFRERLE KRLSAIGEEL LAVADDQPFQ FPAVFTFVFR ALTSIDGAAK

401
11Micromonas_sp__RCC299_Eukary SLNKKFDIGE ISAPYARNLL IDDNAPPQVR QRDWERRFDR QNKAIVNLPE
23Ostreococcus_tauri_Eukaryota TLNKKFDISE IAAPYARNLL IEANSPPQVA QREWLRRADA QTKALVNLPS
09Volvox_carteri_f__nagariensi SLDPRFDISE IAAPYARELL LEGN----GL QREFKKGLEN QNRALKNLPD
60Chlamydomonas_reinhardtii_Eu SLDPRFDISE IAAPYARELL LEGN----GF QKEFAKGLQQ QNRALKNLPE
05Selaginella_moellendorffii_E GLDPYFDISE IAKPYALEIL RFRE----YV IRDFKKRWQR QAEAFNNLPA
02Physcomitrella_patens_subsp_ GLDPRFDISE IAKPYALELL RFRE----VV VKDLRKRWDR QSQAFVNVAE
03Oryza_sativa_Japonica_Group_ GLDPRFDITE IAKPYAMELL RFNE----VI VKDARKRWER QSRAFYNLPE
20Gloeobacter_violaceus_PCC_74 GLDPDFNFMD VAQPFADQLM AVNSEGMREL FNQLGQQVGQ VSSMTVNLPR
35Synechococcus_sp__PCC_7502_B GLDPDFNFME VAKPFATDLM GNNNGASS-L LGELGKQAAQ VGNTAFNLPR
37Synechococcus_elongatus_PCC_ GLDPDFNFMT VAKPYALEIM SDATPPAG-L LNEFSRQALA VGNSALGLPR
28Synechococcus_sp__JA-3-3Ab_B SLDPEFNFLE VAKPFAEELM -NGSKETETL LAQVSRQAAE FTSSSLSLPR
40Chamaesiphon_minutus_PCC_660 GLDPDFNFME AAQPFAFQIM TNGNSPDANF LAEIGRQAAQ VGTSALGLPR
29Synechococcus_sp__PCC_7335_B GLDPEFNFME AAKPYATELM TNGMPEPNGL LGEISRQAAQ VGSSALSLPR
30Leptolyngbya_sp__PCC_7375_Ba GLDPDFNFME AAKPFANQLM TNVSNSTNGL IGEIGRQAAQ VGSSAFGLPR

```
26Microcystis_aeruginosa_PCC_9  GLDPEFNFME VAQPFALQLV NDMTGNNGSI LDELGRQAAQ VSSTALSLPR
16Stanieria_cyanosphaera_PCC_7  GLDPEFNFME VAQPFAMQLM TNSNGRNNSI FDEIGRQAAQ VGSTALGLPR
27Gloeocapsa_sp__PCC_73106_Bac  GLDPEFNFME VAQPFAMELI TGVNGKNGNI LDELGKQAAQ VSSTALGLPR
18Cyanothece_sp__PCC_7822_Bact  GLDPDFNFME VAQPFAIQLM TNDAGNYSSI LDELGRQAAQ MSNTALGLPR
14Pleurocapsa_sp__PCC_7327_Bac  GLDPEFNFME VAQPFAIQLM SNTNGRNGSI FDELGREAAR VGSTALGLPR
39Leptolyngbya_sp__PCC_6406_Ba  GLDPDFNFME AAKPFAAQLM SNGNSNSTDL FGELGRQAAQ MSSTALGLPR
33Geitlerinema_sp__PCC_7407_Ba  GLDPEFNFME VAKPFAMQLM AGGNQQETTL FNELGRQAAQ VSSTALNLPR
31Crinalium_epipsammum_PCC_933  GLDPDFNFME VARPFAMQIM TDGNGDTGKF LNELGRQAAQ VGSTALGLPR
21Moorea_producens_3L_Bacteria  GLDPEFNFME VAKPFALEIM TNGNTPDNNF INELGRQAAQ MSSTALGLPQ
24Calothrix_sp__PCC_6303_Bacte  GLDSEFNFME VAKPYAMQLM TNMNGSEGNI LNELSRQAVQ VSTTALGLPR
22Gloeocapsa_sp__PCC_7428_Bact  GLDPEFNFME VARPFAMDLM TNGNGIEGNF LNELGRQAAQ VGSTAFGLPR
32Chroococcidiopsis_thermalis_  GLDPEFNFME VAKPFAFQLM TDGNGVDGNF LNELSRQAAQ VGSTAFGLPR
25Raphidiopsis_brookii_D9_Bact  GLDPEFNFMA VAQPYAMELM NNNNGSQANF LNELSRQAVQ VSSSALSLPR
17Rivularia_sp__PCC_7116_Bacte  GLDPEFSFME VAKPYAMELM SDMNGSEGNI INELSRQAVQ VSTSAFGLPR
19Fischerella_sp__JSC-11_Bacte  GLDPEFNFME VAQPYAMQLM TNMNGSEGNF FNELSRQAAQ VSSTAFGLPR
34Cyanothece_sp__PCC_7425_Bact  GLDPDFNFME VAKPFATQLM TNGSFNNENI FSELSRQAAQ VGSTAFGLPR
36Oscillatoriales_cyanobacteri  GLDPQFNFME VAKPFAMQLM TNGNPSDANI LSELSRQAAQ VSSTAFGLPR
13Volvox_carteri_f__nagariensi  TLNPNYRFNE VAQPYAAELL QLQDGRGGAL LEQVQQQASE LGAAAAAMPR
10Arabidopsis_lyrata_subsp__ly  ILDPDFSFVK VAAPYAQELL DLKQRRSGTL VQEIRKQADD ARSSTLSMPQ
12Physcomitrella_patens_subsp_  TLDPDFSFAK IAAPYAQELL DTRNGRNGSF VEELQRQAND ARDATVQMPQ
15Selaginella_moellendorffii_E  ILDPKFSFAK IAAPYAQ--- ---------- ---------- TRDATVAMPQ
04Phaeodactylum_tricornutum_CC  TLNPQYDLTK LTLPYLKELL DLKDGAFKTT LIRIGKRLGL RPEDINQAPA
08Thalassiosira_pseudonana_CCM  TLDPKYDLTR LTSPYLKELL DLKDGAFQTT LSRVLKRAGW RPVDFNMAPA
01QUERY_Bigel_abc_86002         TLDKGYDLTR LAVPYLKELL ELREGVVRAV VKQYAEKLGW RKEDINALPA
463Emiliania_huxleyi_Localdb__  GLDPEYDLAK FAQPFIESLQ DSS-EPLAKQ LRVFGTATGL RASDIDTAPA
06Thalassiosira_pseudonana_CCM  GLSDNFDVGK LAQPFIEKFT EVQKEPAEKN FNIFSKATGL NQADILKAPA
07Phaeodactylum_tricornutum_CC  ELDKDFDIGK LAQPFIEDFT ESSKKEADKN FQIFGKATGL NVDDIETAPA
283Aureococcus_anophagefferens  SLDPGYDLTR LSAPYLQELA DLRDGRYKTG VLELLERVGW RPIDVNHLPA
83Porphyridium_cruentum_Eukary  TLDPSYDLTR IAQPYLKDLL DLKDGAFVSI AKSWQKRLGW RWEDIKSLPA
295Ectocarpus_siliculosus_Loca  GLDTKYDLTR LAQPYLKELL DVRDGFALSV AKTFVKKVGW RPEDLEAVPA
537Pyropia_yezoensis_Localdb__  GLDPKYDLTL ISAPYLRELV DLKDGAALTV VKSGLKAVGW RPVDLAQTPA
75Cyanidioschyzon_merolae_Euka  ALDPAFDITR LTKPYLRDLI DLRDGIAKTA LKTATKALGW RKEDLASVPA

                             451
11Micromonas_sp__RCC299_Eukary  EIADVVRAIE RGRLKIRVRA LEAERALERV QVMQGLMLKM MVACAACNVG
23Ostreococcus_tauri_Eukaryota  DVADILRRIE TGKLKIRVRA LEAERAIERM SIMQGVMMQA VIACAATNIG
09Volvox_carteri_f__nagariensi  DIAVTMQRLE RGDLKLRVRA LEAERALTRV QAWQRVIAAA LAASTLVNIG
60Chlamydomonas_reinhardtii_Eu  DIALTMQRLE RGDLKLRVRA LEAERALTRV QAWQRVIAAA LAASTLVNIG
05Selaginella_moellendorffii_E  KLADVIERLE QGDLKLRVRT LESERSFKRV SAVQQNIAQA VFAGTFLNLG
02Physcomitrella_patens_subsp_  KISNMIQRLE QGDLKLRVRV LESERAFKRV ATMQQTIGQA ILAATCLQIA
```

```
03Oryza_sativa_Japonica_Group_  KLAQIIERLE QGDLKLRVRT LESERAFQRV AAVQKTIGYG VAAGSLVNLA
20Gloeobacter_violaceus_PCC_74  RIEQTLERAD RGELRVRTKS IETERLLKRL NSAAIGGIYS VLLAAFLICS
35Synechococcus_sp__PCC_7502_B  RMEETIVKIE RGDIKLRVRS QETDRLLRRL NGVGTSGIYA MIGVGLSLVA
37Synechococcus_elongatus_PCC_  RLDDTLDKLE QGDLRVRVRA SESDRLLRRL LLTQQSQTWA ILTAALWIGS
28Synechococcus_sp__JA-3-3Ab_B  RLEATLSKLE QGELRLRVRS SEAERLLRKL NSTGRGVIYA VLFATFFLSA
40Chamaesiphon_minutus_PCC_660  RLEDTIDKLE RGDIRVRVRS SETDRYLRRL GTMQQNTNYA ILVAAFTLSA
29Synechococcus_sp__PCC_7335_B  RIEDTLDRLD RGDIRVRVRS IESDRILRRN SDLSLANSYT LLVGTFTLSA
30Leptolyngbya_sp__PCC_7375_Ba  RIEDSLDRLD RGDIRVRVRS IETDRAIRRL SSVNLMTNYT LLLGTFTLSA
26Microcystis_aeruginosa_PCC_9  RIDDTIEKLE RGDIRVRVRS SESDRLLRRL GMIQMGTNYT LLISALLISA
16Stanieria_cyanosphaera_PCC_7  RIEDTIDKLE RGDLRVRVRS IESERALRRL SAVQMGTNHT ILIGALILSA
27Gloeocapsa_sp__PCC_73106_Bac  KIEDTIEKLE RGDVLVRVRS QESDRLLRRL GLLQLSTNYT VLTSAFILAA
18Cyanothece_sp__PCC_7822_Bact  RLEDTLDKLE RGDIRVRVRS LESERTFRRL SAIQLGTNYT LFICSLMISA
14Pleurocapsa_sp__PCC_7327_Bac  RIEDTIEKLE RGDIKVRVRS SESDRILRRL SALQLGTNYT LLIGTFILSA
39Leptolyngbya_sp__PCC_6406_Ba  RLEDTLDKLE RGDIRVRVRS IETDRILRRV SGVNMATNYT LLVGAFLLSA
33Geitlerinema_sp__PCC_7407_Ba  RIEDTLGKLE QGDIRVRVRS TETDRALRRL SSVTMGINYM LLICTFILSA
31Crinalium_epipsammum_PCC_933  RIEDTIEKLE RGDLRVRVRS TETDRVLRRI SSVQLGTNYT LLIGTFTLSA
21Moorea_producens_3L_Bacteria  RIESTIDKLE RGDLRIRVRS IESDRVLRRL SGIQLGTNYT LLISAFTLSA
24Calothrix_sp__PCC_6303_Bacte  RLEDVIEKLE RGDVRLRVRA IETERLLRKQ SSLQLASVYA VILSGFTLSA
22Gloeocapsa_sp__PCC_7428_Bact  RLEDTLEKLE RGDIRVRVRS IETERLLRRQ SNLQLGTNYT IIISAFTLSA
32Chroococcidiopsis_thermalis_  RLEDTLEKLE RGDLRVRVRS IETERLLRRQ SSVQVAITYA LIVSSFTISA
25Raphidiopsis_brookii_D9_Bact  RLEDTLEKLE RGDIRLRVRA LETERLLRRQ SNIQMGISYA LIISAFTISA
17Rivularia_sp__PCC_7116_Bacte  RLEDTLEKLE RGDVRVRVRS IETERLLRRQ TNIQLGGTYA LIISGFTLSA
19Fischerella_sp__JSC-11_Bacte  RLEDTLEKLE RGDVRLRVRS IETERLLRRQ SNIQLGIAYA LIISAFTLSA
34Cyanothece_sp__PCC_7425_Bact  RLEETLEKLE NGDIRIRTRS TETDRILRRL SSVNMGTNYV LLIGSFTLSA
36Oscillatoriales_cyanobacteri  RIEDTLDKLE RGDIRVRVRS TETDRILRRM SSINMGTNHT LLVCTFTLSA
13Volvox_carteri_f__nagariensi  RIEATVAQLE TGDLKLRVRV LEGERADRRA GVLQMATLNT VACMGLLNVG
10Arabidopsis_lyrata_subsp__ly  RIEEFVKELD SGDLKLRVRV LESERAARKA TILQMATMYT VLGGTLLNIG
12Physcomitrella_patens_subsp_  RVDQIMRQLE SGDLKLRVRS LEAERAARRA SVMQEAIINS LFSVGLANIG
15Selaginella_moellendorffii_E  HIEKFMQQLE DGDIKLRVRV LEAERAARKS SVLQTTTMNL VAAATLVNVG
04Phaeodactylum_tricornutum_CC  RVEDVVTRME QGDFKLRVRA LEVERELERS KLVQKNTFQA ILSGLLFQGA
08Thalassiosira_pseudonana_CCM  KIEDITTRLE KGELKPRVRA MEVERMIKRN KLVQSNIFSA VLSCLFLNSA
01QUERY_Bigel_abc_86002         YISNVIRKLE KGDLKLRTRT LESERAFERS AMVQQNQGKL IVASALLNSA
463Emiliania_huxleyi_Localdb__  YLEQTVRAME QGQLKIRVRS IENEQALARL ALSGEVTNKL LASSILLNLG
06Thalassiosira_pseudonana_CCM  YIEDTLRSME RGELKIRVRS LENEKALERM ALTQGKMEKL LVASFLLNLA
07Phaeodactylum_tricornutum_CC  YIEETMRSME DGTLKIRTRS LENEKALERV GLRQGIMENV LLGSLFFNMA
283Aureococcus_anophagefferens  STDASIKRIE AGDLTLRTRS VELEAQLTRV EARQRMFQYG TVAALALRLA
83Porphyridium_cruentum_Eukary  YVSQTLEKME QGDLKVRVRA SRRQRCAFIG HIVEKLLQLR LDQGITLAFL
295Ectocarpus_siliculosus_Loca  YVEDITRKLE QGDLKLRVRV LESERAFERL ELTQTSMYDA IAFSTFLNAA
537Pyropia_yezoensis_Localdb__  YVERTLRRME EGELRL---- ---------- ---------- ----------
75Cyanidioschyzon_merolae_Euka  YIESVLRKIE TGDLKIRVRT LEIERSFKLL SVMVEILGHG MLASIFMNMA
```

```
                                                  501
11Micromonas_sp__RCC299_Eukary   VVLYVSGMLL QAKGVFGVAG LFAMQALVAQ MKLGKLIKKE
23Ostreococcus_tauri_Eukaryota   VVMYVSGLML QAQCAFGFAG LLAFKALTGQ LKVAKLLKKE
09Volvox_carteri_f__nagariensi   TVLSVSALTT GATASFVGAA LFGFMLLKNY LKVLQLEKKE
60Chlamydomonas_reinhardtii_Eu   TVLSVSALTA GATASFAGAA LFGFMLLKNW LTVVKLEKKE
05Selaginella_moellendorffii_E   TLLYINAMRV QGTAAFFCSA LFGLKLLVGL FKVKRLDKQE
02Physcomitrella_patens_subsp_   TMLHLSAITV PATAAFTGSA LFGLQAFIGI MKVKKLDKQE
03Oryza_sativa_Japonica_Group_   TVLYLNSIRL PATIAYSLCA FFGLQVLVGL LKVKKLDQQE
20Gloeobacter_violaceus_PCC_74   VVLYTADHIL EAIAAFGLAG LLSFALGRVL LRLQRIDTDL
35Synechococcus_sp__PCC_7502_B   TILFVTDWYW LAAIALILGS ICIITSLRML ARMDRMEKMF
37Synechococcus_elongatus_PCC_   AIVSLSALPL LAIAPFGLGL IPLGLLIRTQ LRLRRSERMG
28Synechococcus_sp__JA-3-3Ab_B   TQLFSAGHAT LAIVTLALAV LAALALVRVL LKANGSEPLM
40Chamaesiphon_minutus_PCC_660   TILFVNAQAI PAGVVGTMAA IALVAWFRQM RKLDRQDRMS
29Synechococcus_sp__PCC_7335_B   TLLLIFEYTL AALIMGAIAV LSGVTLIRLL MRIKKADRMP
30Leptolyngbya_sp__PCC_7375_Ba   TLLLISQLPW VALGMILPAI ASGIALVRAL MRLGRTDRMP
26Microcystis_aeruginosa_PCC_9   TLLFISGFGW LTLVPMAISL LPGIALLRLW RKIERQDRMM
16Stanieria_cyanosphaera_PCC_7   TLLFVNGSIN LAAIAAGLAL VPAWALLRLY RKIDRSDRMF
27Gloeocapsa_sp__PCC_73106_Bac   TILLVNGYVK IAIAAILIAL VPAFALLRLL RRIDRLDRMF
18Cyanothece_sp__PCC_7822_Bact   TLLFVHQYVT IAVVVLLVAI FPAWALFRLL RQLDRLDRMF
14Pleurocapsa_sp__PCC_7327_Bac   TILYVYGKVQ AAIAVLLLAI APAWAFFRLL RRLDRLDRMF
39Leptolyngbya_sp__PCC_6406_Ba   TILLVYEYLW LAIAAAAVAT VAGFALVRLL LRIGRADRLP
33Geitlerinema_sp__PCC_7407_Ba   TILFVHGKGW LAGAALVAAG AIAFFLIRMF KRIDRYDRMF
31Crinalium_epipsammum_PCC_933   TILLVNNYVW LAGVVAFIAV SLLVILIRLL MRLDKFDRTF
21Moorea_producens_3L_Bacteria   TILFVNGNVV LALVVVLVAA MLGFALIRLL RRLDRFDRML
24Calothrix_sp__PCC_6303_Bacte   SILLIKDYIW LAVGAGLIAA FVSWLLIRLL LRLDRNDRSY
22Gloeocapsa_sp__PCC_7428_Bact   TLLFVNHYIW LAIIAAAIAA VVGVALVRLL IRLDRYDRMY
32Chroococcidiopsis_thermalis_   TILLISQFAW LALIPGAIAA GTGFSLIRLL MRLDRYDKMF
25Raphidiopsis_brookii_D9_Bact   TILLVNRFYW LATIAGLIVA GISFIFLRLV SRLDRYDQKY
17Rivularia_sp__PCC_7116_Bacte   TILLVHDYVW FALVAGLIAA AVSWMLIRLL LRLDRYDRMY
19Fischerella_sp__JSC-11_Bacte   TILFVNNYFW QAVFIGLIAT AVLWLLIRLL MRLDRYDRMY
34Cyanothece_sp__PCC_7425_Bact   TILLVNQFAG LAAIAATIAV LATIAYIRLL LRLDKYERML
36Oscillatoriales_cyanobacteri   TILLVNGYVW LAVIAGLAAL IAGFVLLRLM MRIDRADRMP
13Volvox_carteri_f__nagariensi   TMLAISGGPG AAAAVATGSA VFGVLVFRGL KRVQRLDKFE
10Arabidopsis_lyrata_subsp__ly   VTFSNQGSQL VANGSFIGAG IFMALVLRSM QRVNKLDKFE
12Physcomitrella_patens_subsp_   VTLSTQGITA VSNSSFVGAG IFLLLLANNV RRIRNLDKFE
15Selaginella_moellendorffii_E   VTLSTQGLQD FAIASFLGAG K--------- ----------
04Phaeodactylum_tricornutum_CC   VSLATVGSGL YAATLFAVSA VLGIQIPFGI FRLRKLDKYN
08Thalassiosira_pseudonana_CCM   MSVAVLGQNM L--------- ---------- ----------
01QUERY_Bigel_abc_86002          MSMFSTAPAV ASQAAFGLAI FFALQFPLGA LKVRSFDKKS
```

```
463Emiliania_huxleyi_Localdb__  L---AGVGAV PRAVWFAAAA VMGAQTAAAN LKISLFDKKA
06Thalassiosira_pseudonana_CCM  G---VATNRL LRSAGVVGAV AFGLQAFMAS AKLKKFDKTQ
07Phaeodactylum_tricornutum_CC  G---LASRTV FRTAGIVGAS FFLFQALMAN AKVKKFDKTQ
283Aureococcus_anophagefferens  TLALAEAPLK YAALGAAWAA AEGFGAYCAL CKLDKNQRRF
83Porphyridium_cruentum_Eukary  L-DTSGASEV FFSVSDADPS ISSFSSPTRS -SAPLSSSSS
295Ectocarpus_siliculosus_Loca  LTLSAGSPLL PVRVAWTLAG VFGLRIPVGL FKIRKQDQKY
537Pyropia_yezoensis_Localdb__  ---------- ---------- ---------- ----------
75Cyanidioschyzon_merolae_Euka  L------AGV FASACYLGAA VCGVRALVGF LNLRIIQQRQ
```

Alignment: GGR

```
009Lotharella_amoeboformis_Euk   ---------- ---------- ---------- ---------- ----------
101Cyanidioschyzon_merolae_Euk   KFRVAVVGGG PAGACAAEAL ARCEIETFLI ERKMDNCKPC GGAIPLCMVD
120Emiliania_huxleyi_Localdb__   PLRVAVVGGG PSGACAAEIF AKANIDCTLF ERKMDNCKPC GGAIPLCMVD
006Isochrysis_galbana            --RVAVVGGG PAGACAAEIF AQANIECTMF ERKMDNCKPC GGAIPLCMVE
001QUERY_ggr                     KYRVAVIGGG PSGSAAAEIF AKEGIDTFIF ERKMDNVKPC GGAIPLCMVD
003Guillardia_theta              KMRVAIVGGG PSGSCAAEIF AQENIDTYIF ERKMDNAKPC GGAIPLCMIE
004Ectocarpus_siliculosus        KYKVAVVGGG PSGSCAAEIL ADCDVDVTLF ERKMDNAKPC GGAIPLCMID
005Aureococcus_anophagefferens   KYRVAVVGGG PSGACAAEIC AQENIETYII ERKFDNAKPC GGAIPLCMVD
010Thalassiosira_pseudonana_CC   KYKVAVVGGG PSGACAAEIF AQEGIDTVMF ERKMDNAKPC GGAIPLCMIG
011Phaeodactylum_tricornutum_C   KYKVAVVGGG PAGACAAEIF AQEGIDTVLF ERKLDNAKPC GGAIPLCMVG
036Brachypodium_distachyon_Euk   RLRVAVVGGG PAGGAAAEAL AKGGVETVLI ERKMDNCKPC GGAIPLCMVS
031Selaginella_moellendorffii_   KLRVAVIGGG PAGGCAAETL AKNGIETFLI ERKMDNAKPC GGAIPLCMVG
021Physcomitrella_patens_subsp   KLRVAVVGGG PAGGCAAETL AMGGIETFLI ERKLDNAKPC GGAIPLCMVG
015Ostreococcus_tauri_Eukaryot   KLRVAVIGGG PAGACAAETL AKGGCETYLI ERKMDNCKPC GGAIPLCMVG
028Micromonas_pusilla_CCMP1545   ---------- ---------- ---------- ---MDNCKPC GGAIPLCMVG
012Volvox_carteri_f__nagariens   KLRVAVIGGG PSGACAAETL AKGGVETYLI ERKLDNCKPC GGAIPLCMVE
013Chlamydomonas_reinhardtii_E   KLRVAVIGGG PSGACAAETL AKGGVETFLL ERKLDNCKPC GGAIPLCMVE
007Pyropia_yezoensis             --RVAVVGGG PAGSCCAEEL AKAGIEVTLF ERKMDNAKPC GGAIPLCMVD
019Synechococcus_elongatus_PCC   ALRVAVVGGG PAGSCAAETL VKAGIETYLI ERKLDNAKPC GGAIPLCMVS
035cyanobacterium_UCYN-A_Bacte   VLRVAVVGSG PAGSSAAETL VKAGIETYLF ERNLSYVKPC GGAIPLCMVD
024Thermosynechococcus_elongat   SLRVAVVGGG PAGSSAAETL AKAGIQTYLF ERKLDNAKPC GGAIPLCMVS
014Cyanothece_sp__PCC_7425_Bac   ALRVAVVGGG PAGSSAAEVL ARAGIETYLI ERKLDNVKPC GGAIPLCMVS
032Acaryochloris_sp__CCMEE_541   ALRVAVVGSG PAGSSAAETL VKAGIETYLF ERKLDNAKPC GGAIPLCMVD
020Microcoleus_vaginatus_FGP-2   TLRVAVVGSG PAGSSAAETL VKAGIETYLF ERKLDNAKPC GGAIPLCMVS
030Trichodesmium_erythraeum_IM   TLRVAVVGSG PAGSSAAETL VKAGIETYLF ERKLNNAKPC GGAIPLCMVS
025Lyngbya_sp__PCC_8106_Bacter   TLRVAVVGSG PAGSSAAETL VKAGIETYLF ERKLDNAKPC GGAIPLCMVS
018Cylindrospermopsis_racibors   TLRVAVVGSG PAGSSAAETL AKAGIETYLF ERKLDNAKPC GGAIPLCMVS
017Fischerella_sp__JSC-11_Bact   TLRVAVVGSG PAGSSAAETL AKAGIETYLF ERKLDNAKPC GGAIPLCMVS
027Nostoc_azollae_0708_Bacteri   TLRVAVVGSG PAGSSAAETL AKAGIETYLI ERKLDNAKPC GGAIPLCMVS
023Microcoleus_chthonoplastes_   ALRVAVVGSG PAGSSAAETL AKAGIETYLF ERKLDNAKPC GGAIPLCMVS
026Cyanothece_sp__PCC_7822_Bac   MLRVAVVGSG PAGSSAAETL AKAGIETYLF ERKLDNAKPC GGAIPLCMVS
008Galdieria_sulphuraria         ---------- ---------- ---GIETFIF ERKFDNAKPC GGAIPLCMIE
002Cyanophora_paradoxa           KLRVAIVGGG PAGSCAADVL AKAGIETYLF ERKLDNAKPC GGAIPLCMVS

                                 51
009Lotharella_amoeboformis_Euk   ---------- ---------- ---------- ---------- ----------
101Cyanidioschyzon_merolae_Euk   EFGLPPDIID RKVRRMLMVS PSGREIPVRT LKPHEYIGMT RREILDSYLR
120Emiliania_huxleyi_Localdb__   EFELPPEIVD RRVRKMKMIS PSGREVDVRT LKENEYIGMT RREVLDAYLR
```

```
006Isochrysis_galbana            EFSLPPDIVD RRVRKMKMIS PSGREVDVRT LKEDEYIGML RREVMDKYLR
001QUERY_ggr                     EFDLPESVID RKVRKMKMIS PSNRVVDVRT LKPNEYIGMC RREVLDGFMR
003Guillardia_theta              EFQIPEEVVD RKVRKMLMIS PTGKEAPIQT LRDDEYIGMT RREILDGFLR
004Ectocarpus_siliculosus        EFDLPTSIID RKVRKMKMIS PTNREVDIQT LGDDEYIGMV RREVMDGFMR
005Aureococcus_anophagefferens   EFDLPADIID RKVRKMKMIS PSNREVDIKT LKEDEYIGMC RREVMDGYLR
010Thalassiosira_pseudonana_CC   EFDIPESTVD RKVRRMKLIS PSNNEVDIDT LQPNEYIGMC RREIMDKYLR
011Phaeodactylum_tricornutum_C   EFDLPESVVD RKVRRMNLIS PKGVEVDIET LKPDEYIGMT RREILDGYLR
036Brachypodium_distachyon_Euk   EFDLPLDLVD RKVTKMKMIS PSNVAVDIRT LAPHEYIGMV RREVLDDFLR
031Selaginella_moellendorffii_   EFDLPLDIID RKVTKMKMIS PSNVAVDVKT LNENEYIGMV RREVLDSYLR
021Physcomitrella_patens_subsp   EFDLPPEIID RKVTKMKMIS PSNVAVDVKT LNDDEYIGMV RREVLDSFLR
015Ostreococcus_tauri_Eukaryot   EFDLPEEIID RKVTKMKMIS PSNRAVDVQT LNDKEYIGMC RREVMDDFLR
028Micromonas_pusilla_CCMP1545   EFDLPEEIID RKVTKMKMIS PSNRAVDVQT LNDKEYIGMC RREVMDDFLR
012Volvox_carteri_f__nagariens   EFDLPLEIID RKVTKMKMIS PSNREVDVKT LSETEWIGMC RREVFDDFLR
013Chlamydomonas_reinhardtii_E   EFDLPMEIID RRVTKMKMIS PSNREVDVKT LSETEWIGMC RREVFDDYLR
007Pyropia_yezoensis             EFSLPEEIID RKVRKMAMIS PSNKVSQIAT LKPHEYIGMT RREILDGYLR
019Synechococcus_elongatus_PCC   EFDLPAEIID RRVRNMKMIS PSNREVNINL DNADEYIGMT RREVLDGFLR
035cyanobacterium_UCYN-A_Bacte   EFELPPTIID RRVRKMKMIS PSNIEVDLNL DNKDEYIGMC RREILDGFLR
024Thermosynechococcus_elongat   EFDLPPEIID RRVRKMKMIS PSNIEVNIHT LKEHEYIGMC RREVLDSFMR
014Cyanothece_sp__PCC_7425_Bac   EFDLPPEIID RRVRKMKMIS PSNIEVNIST LKQDEYIGMC RREVLDGFLR
032Acaryochloris_sp__CCMEE_541   EFDLPLEIID RQVRKMRMIS PSNVEVDINI KNEGEYIGMC RREVLDGFMR
020Microcoleus_vaginatus_FGP-2   EFDLPQSIID RQVRKMKMIS PSNVEVDINI ENAHEYIGMC RREVLDGFMR
030Trichodesmium_erythraeum_IM   EFDLPPEIID RRVRKMKMIS PSNVEVDIHI EKEDEYIGMC RREVLDGFLR
025Lyngbya_sp__PCC_8106_Bacter   EFDLPPEIID RRVRKMKMIS PSNREVDINL INENEYIGMC RREVLDGFMR
018Cylindrospermopsis_racibors   EFDLPADIID RRVRNMKMIS PSNREVDINL INQEEYIGMC RREVLDGFLR
017Fischerella_sp__JSC-11_Bact   EFDLPQDIID RQVRKMKMIS PSNREVDINL VKQEEYIGMC RREVLDGYLR
027Nostoc_azollae_0708_Bacteri   EFDLPPEIID RRVRKMKMIS PSNREVDINL VNEEEYIGMC RREVLDGFLR
023Microcoleus_chthonoplastes_   EFDLPPEIID RRVRKMKMIS PSNIEVDINL VKEDEYIGMC RREILDGFMR
026Cyanothece_sp__PCC_7822_Bac   EFDLPPEIID RRVRKMKMIS PSNIEVDINL DNQEEYIGMC RREVLDGYMR
008Galdieria_sulphuraria         EFSLPLDIVD RKVRRMKMIS PSNREVDIKT LKPHEYIGMT RREVLDSFLR
002Cyanophora_paradoxa           EFDLPESIID RKVTRMKMIS PSNVEVDIQT LKPNEYIGMT RREVLDGFLR

                                101
009Lotharella_amoeboformis_Euk   ---------- ---------- ---------- ---------- ----------
101Cyanidioschyzon_merolae_Euk   ERAYELGAIR VHGLVQEVEL PDPRDGKYRI KYADYKIDGG RATTSSLEVD
120Emiliania_huxleyi_Localdb__   DRAVELGAKP VTGLVQKVEF -EDANGPYTI KYSEYKPGEK KGTPSEAEFD
006Isochrysis_galbana            DRAVQFGATP VNGLVQKVEF -ADTDGPYTI KYTKYEEGTP KGTPAEAEFD
001QUERY_ggr                     NRSVDLGAVP INGLVTKIDV PRGDSDPYVI HYQDYKTAGG AGTPKTMEFD
003Guillardia_theta              DRAIKLGAKP INGLVTGIDL PEGNSKPYKI HYTNY-NGQK VGTPEELDVD
004Ectocarpus_siliculosus        DRAIEKGAHA INGLVNDIDV PADKDGKYKI HFARYEKGSR KGVQDSEEFD
005Aureococcus_anophagefferens   DRAVEYGAIP INALVTKIDT PADSDGRYTI HYSRNDLG-R KAPEESLDVD
```

```
010Thalassiosira_pseudonana_CC  DRAISYGAEP INGLVTAIDV PEDHKKQYTL HYQEFQAGSS AGVPKSMNVD
011Phaeodactylum_tricornutum_C  DRAIDYGAEI VNGLVTSIDI PADHKNQYTI NYQAYEEGSS AGTPKNMKVD
036Brachypodium_distachyon_Euk  TRAQKAGAEV LNGLFLRYEA PKEANGTYTV HYNHYDSSSG KGEKRSFEVD
031Selaginella_moellendorffii_  ERAASFGANL VNGLFLRMEM PSDANAPYVL HYNLYKPGSS VGVPATLEVD
021Physcomitrella_patens_subsp  ERAQKNGATI INGLFLRMEV PKDNDSPYKL HYTNFDGSSK VGTPAVLEVD
015Ostreococcus_tauri_Eukaryot  KRANSLGANL VNGLFMKIED PGQGNGPILV QYNDYEEESK TGVPKTLEVD
028Micromonas_pusilla_CCMP1545  KRAKSLGTNL VNGLFMKLED PGQGNGAITI HYNDYEADSK VGEPKTMEVD
012Volvox_carteri_f__nagariens  KRAEKLGANI VNGLFMRSEK -QGSDGPFTI HYTSYADGSK MGTPSTLEVD
013Chlamydomonas_reinhardtii_E  NRAQKLGANI VNGLFMRSEQ -QSAEGPFTI HYNSYEDGSK MGKPATLEVD
007Pyropia_yezoensis            DRAISLGAKP INGLVTDIKI PSNSSEPYVI SYSDYAEGGA QG--KTLEVD
019Synechococcus_elongatus_PCC  DRAAKLGTKL INGTLFRLEL PKGDRDPYVL HYADHSNGSL EGIPSTLEVD
035cyanobacterium_UCYN-A_Bacte  DRAVELGANL INGTVYQLDT PQNSQDPYTI YYADHSNGQA LGEKKTLQVD
024Thermosynechococcus_elongat  NRAADLGANL INGTVFKLEQ PTTSDRPYTL HYVQED---- -GTAQTLEVD
014Cyanothece_sp__PCC_7425_Bac  DRAAHCGATV INGTMHTLDL PSNDRDPYVL HYADHSNGSA AGENKTLKVD
032Acaryochloris_sp__CCMEE_541  DRAAKLGAKL INGTVNKLDF PTGANQPYTI HYADLTGGRA EGVMKTLQVD
020Microcoleus_vaginatus_FGP-2  DRAASLGAKL INGTVHKLEI PGNNTDPYTL HYADHSDGSA MGIQKTLKVD
030Trichodesmium_erythraeum_IM  DRAASLGANL INGTVYKLDL PNNDREPYTI HYSDHSQGDI QGTAKTLKVD
025Lyngbya_sp__PCC_8106_Bacter  DRAAKLGANL INGTVYKLDI PTKKNEAYTL HYTDHSEGNS RGTAKTLKVD
018Cylindrospermopsis_racibors  NRAAKLGANL INATVHKLDI PNNNSDPYTI HYVDHNEGGS VGVAKTLKVD
017Fischerella_sp__JSC-11_Bact  NRAAKLGANL INATVHKLDI PRNNTDPYTI HYVDHTEGGA QGIAKTLKVD
027Nostoc_azollae_0708_Bacteri  ERAAKLGANL INATVHKLDI PTNNTDPYTI HYVDHTEGGA QGITKTLKVD
023Microcoleus_chthonoplastes_  NRAAKLGATL INGTVHKLDI PTSNTEPYTL HYADHSDGSL SGVQKTLKVD
026Cyanothece_sp__PCC_7822_Bac  NRAAKLGATL INATVYKLDI PTNNTDPYTI HYADHSDGSI EGIKKTLKVD
008Galdieria_sulphuraria        ERAIQLGAQP IHGLVTDIIL PKTDREPYII CYSDFSENG- IGKPNTLQVD
002Cyanophora_paradoxa          ERAEK-NTKV INGLFLSMDK PKSGDGAYTL HFTDYSNG-I KGEPNSIDVD

                              151
009Lotharella_amoeboformis_Euk  -------ANS RVAKAIDAGE YNYAIAFQER IAINDEQMKY YEELAEMYVG
101Cyanidioschyzon_merolae_Euk  MVVGADGANS RVAKAIDAGD YSYAIAFQER IKIPDDKMAF YEDLAEMYVG
120Emiliania_huxleyi_Localdb__  IIVGADGANS RTAKAIDAGE YNFAIAFQER IKIPDEKMKY YEDLAEMYVG
006Isochrysis_galbana           IIVGADGANS RTAKAIDAGE YNYAIAFQER IKIADEKMAY YEDLAEMYVG
001QUERY_ggr                    VLVGADGANS RVAKAIDAGE YNYAIAFQER IAIDEEKMEY YKDLAEMYVG
003Guillardia_theta             LIIGADGANS RVAKAIDAGE YNYAIAFQER IRINDQQMAF YEELAEMYVG
004Ectocarpus_siliculosus       LIIGSDGANS RVAKAMDAGA YNYAIAFQER IKISEKKMEF YEEMAEMYVG
005Aureococcus_anophagefferens  VIIGADGANS RVAKAIDAGE YNFAIAFQER IKIDDEKMAF YDELAEMYVG
010Thalassiosira_pseudonana_CC  LIIGADGANS RVAKAMDAGE YNFAIAFQER IKITDEQMSF YEEMAEMYVG
011Phaeodactylum_tricornutum_C  LIVGADGANS RVAKAMDAGQ YNFAIAFQER IKIPDDKMKF YEEMAEMYVG
036Brachypodium_distachyon_Euk  AIVGADGANS RVAKDMGAGD YEYAIAFQER VKIPDDKMRY YEERAEMYVG
031Selaginella_moellendorffii_  AVIGADGANS RVAKDIDAGD YDYAIAFQER IKIPDDKMVY YENLAEMYVG
021Physcomitrella_patens_subsp  AVIGADGANS RVAKDIDAGE YDYAIAFQER IKIPADKMEY YENLAEMYVG
```

```
015Ostreococcus_tauri_Eukaryot  VVIGADGANS RVAKCIDAGE YDYAIAFQER IKIPDEKMEY YKDLAEMYVG
028Micromonas_pusilla_CCMP1545  CIIGADGANS RVAKNIDAGE YDYAIAFQER IKIPDEKMEY YKDLAEMYVG
012Volvox_carteri_f__nagariens  MIIGADGANS RVAKEIDAGE YDYAIAFQER IRIPDDKMKY YENLAEMYVG
013Chlamydomonas_reinhardtii_E  MIIGADGANS RIAKEIDAGE YDYAIAFQER IRIPDDKMKY YENLAEMYVG
007Pyropia_yezoensis            YIIGADGANS RVAKAIDAGD YQYAIAFQER IKLPAAQMEY YEELAEMYVG
019Synechococcus_elongatus_PCC  VVIGADGANS RIAKAIDAGD YNYAIAFQER IRLPEDKMHY YENLAEMYVG
035cyanobacterium_UCYN-A_Bacte  VIIGADGANS RIAKFIDAGD YNYAIAFQER IRLPKDKMNR YEDLAEMYVG
024Thermosynechococcus_elongat  VVIGADGANS RIAKEIDAGD YNYAIAYQER IRLPEDKMAY YNELAEMYVG
014Cyanothece_sp__PCC_7425_Bac  LVIGADGANS RVARAIDAGD YNYAIAFQER IRLPEDKMAY YEDLAEMYVG
032Acaryochloris_sp__CCMEE_541  LVIGADGANS RIAKEIKAGD YNYAIAFQER IRLPDEYMAY YQDRAEMYVG
020Microcoleus_vaginatus_FGP-2  LVIGADGANS RVAKAIDAGD YNYAIAFQER IRLPEDKMAY YEDLAEMYVG
030Trichodesmium_erythraeum_IM  LVIGADGANS RVAKAIDAGD YNYAIAFQER IRLPDHLMAY YEDLAEMYVG
025Lyngbya_sp__PCC_8106_Bacter  LVIGADGANS RIAKAIDAGD YNYAIAFQER IRISDEKMAY YQDRAEMYVG
018Cylindrospermopsis_racibors  LIIGADGANS RIAKEMDAGD YNYAIAFQER IRLPEDKMAY YNNLAEMYVG
017Fischerella_sp__JSC-11_Bact  LIIGADGANS RIAKEIDAGD YNYAIAFQER IRLPQEQMAY YNDLAEMYVG
027Nostoc_azollae_0708_Bacteri  LVIGADGANS RIAKEMDAGD YNYAIAFQER IRLPQDKMAY YNDMAEMYVG
023Microcoleus_chthonoplastes_  LVIGADGANS RVAKAIDAGN YNYAIAFQER IRLPGDMMNY YEDLAEMYVG
026Cyanothece_sp__PCC_7822_Bac  VVIGADGANS RIAKAIDAGD YNYAIAFQER IRLPQDKMAY YEDMAEMYVG
008Galdieria_sulphuraria        MIIGADGANS RVAKVIDAGD YNYAIAFQER IRLPPEKMAY YEELAEMYVG
002Cyanophora_paradoxa          MVIGADGANS RVAKEIDAGD YEYAMAFQER IRLPKDKMEF YENLAE-YVG

                              201
009Lotharella_amoeboformis_Euk  DDVS-DFY-W VFPKYDHVGV GT-------- --KYQKAIRE RCKERIEGGK
101Cyanidioschyzon_merolae_Euk  DDVSPDFYAW VFPKYDHVGV GTGTVIDKPN IKRYKEGIRQ RAAPKIAGGK
120Emiliania_huxleyi_Localdb__  DDVSPDFYAW VFPKYDHVGV GTGTVVNRPA ISEYQAAIRE RCADRLGDPK
006Isochrysis_galbana           DDVSPDFYAW VFPKYDHVGV GTGTVVNRPA ISEYQAAIRE RCAEKLGDPK
001QUERY_ggr                    DDVSPDFYAW VFPKYDHVGV GTGTVVNRPD IPKYQKAIRE RAKDRLEGGK
003Guillardia_theta             DDVSPDFYGW VFPKYDHVGV GTGTVVNRPA IKQYQNAIRE RAGKKIEGGK
004Ectocarpus_siliculosus       DDVSPDFYGW VFPKYDHVGV GTGTVVNRPK IKDYQDAIRK RAGEKIEGGK
005Aureococcus_anophagefferens  DDVSPDFYGW VFPKYDHVGV GTGTVINRPG IKMYQDAIRE RAGDKIAGGK
010Thalassiosira_pseudonana_CC  DDVSPDFYGW VFPKYDHVGV GTGTVVNRPA IKQYQKAMRD RAGDKIAGGK
011Phaeodactylum_tricornutum_C  DDVSPDFYGW VFPKYDHVGV GTGTVVNRPA IKQYQKAIRD RAGDKIAGGK
036Brachypodium_distachyon_Euk  DDVSPDFYGW VFPKCDHVAV GTGTVTHKAD IKKFQAATRL RAKDKIDGGR
031Selaginella_moellendorffii_  DDVSPDFYGW VFPKCDHVAV GTGTVINKPA IKKYQVATRH RARDKIAGGK
021Physcomitrella_patens_subsp  DDVSPDFYGW VFPKCDHVAV GTGTVINKPS IKKYQTATRN RAKDKIAGGK
015Ostreococcus_tauri_Eukaryot  DDVSPDFYGW VFPKYDHVAV GTGTVVNKTA IKQYQTAMRN RAEEKCEGGK
028Micromonas_pusilla_CCMP1545  DDVSPDFYGW VFPKYDHVAV GTGTVVNKTA IKTYQAAMRK RAEEKCAGGK
012Volvox_carteri_f__nagariens  DDVSPDFYGW VFPKYDHVAV GTGTVVNKTA IKQYQQATRD RSKVKTEGGK
013Chlamydomonas_reinhardtii_E  DDVSPDFYGW VFPKYDHVAV GTGTVVNKTA IKQYQQATRD RSKVKTEGGK
007Pyropia_yezoensis            DDVSPDFYAW VFPKYDHVGV GTGTVIDRPS IKKYQQAIRT RAADKLAGGK
```

```
019Synechococcus_elongatus_PCC  GDVSPDFYAW VFPKYDHVAV GTGTMVNQRL IKKLQAGIRA RAANRLEGGK
035cyanobacterium_UCYN-A_Bacte  KDVSPDFYAW IFPKYDHVAV GTGTMNNQAM IKKLQAGIRS RAFSRLEGGE
024Thermosynechococcus_elongat  DDVSPDFYAW VFPKYDHVAV GTGTMVNKDR IRELQAGIRS RAAAKLEGGQ
014Cyanothece_sp__PCC_7425_Bac  DDVSPDFYAW VFPKYDHVAV GTGTMPNQAR IKQLQAGIRE RAAAKLRGGK
032Acaryochloris_sp__CCMEE_541  DDVSTDFYAW VFPKYDHVAV GTGTMVNQAD IKKLQAGIRA RAAHRLVGGE
020Microcoleus_vaginatus_FGP-2  DDVSTDFYAW VFPKYDHVAV GTGTMVNQAS IKKLQAGVRA RAAKKLMGGE
030Trichodesmium_erythraeum_IM  DDVSPDFYAW VFPKYDHVAV GTGTMVHQAK IKQLQAGIRA RAAKRLEGGQ
025Lyngbya_sp__PCC_8106_Bacter  NDVSPDFYAW VFPKYDHVAV GTGTMVNQAM IKQLQAGIRA RAAHRIEGGK
018Cylindrospermopsis_racibors  DDVSTDFYAW IFPKYDHVAV GTGTMVNKAS IKQLQAGIRA RAAEKLAGGT
017Fischerella_sp__JSC-11_Bact  DDVSTDFYAW VFPKYDHVAV GTGTMVNKAS IKQLQAGIRA RAARKLVGGQ
027Nostoc_azollae_0708_Bacteri  NDVSTDFYAW VFPKYDHVAV GTGTMVNKAN IKQLQAGIRA RASKKLAGGQ
023Microcoleus_chthonoplastes_  NDVSPDFYAW VFPKYDHVAV GTGTMVNQAK IKKLQAGIRA RAAKKLVGGE
026Cyanothece_sp__PCC_7822_Bac  KDVSPDFYAW VFPKYDHVAV GTGTMVNKAM IKDLQAGIRT RAARKLEGGE
008Galdieria_sulphuraria        DDVSPDFYAW VFPKYDHVGV GTGTVLDRPS IKKYQAAIRR RAASKLNGGH
002Cyanophora_paradoxa          DDVSPDFYAW VFPKYDHVAV GTGTVVNRPG IKGYQTAIRE RAARKLEGGE

                                251
009Lotharella_amoeboformis_Euk  IIKVEGSSHS RAPSPPPCTR RAALV----- ---------- ----------
101Cyanidioschyzon_merolae_Euk  VIKVEAHPIP EHPRPRRVVG RVALVGDAAG YVTKCSGEGI YFAAKSGRMC
120Emiliania_huxleyi_Localdb__  VMKVEAHPIP EHMRPRRVVG RVALVGDAAG YVTKCSGEGI YFAAKSGRMA
006Isochrysis_galbana           VMKVEAHPIP EHMRPRRVVG RVALVGDAAG YVTKCSGEGI YFAAKSGRMA
001QUERY_ggr                    IIKVEAHPIP EHPRPRRVQG RAALVGDAAG YVTKCSGEGI YFAAKSGRMA
003Guillardia_theta             IIKVEAHPIP EHYRPKRVLN RAALVGDAAG YVTKCSGEGI YFAAKSGRMA
004Ectocarpus_siliculosus       IIKIEAHPIP ENYRPRRVVG RMALVGDAAG YVTKCSGEGI YFAAKSGRMA
005Aureococcus_anophagefferens  IIKVEAHPIP EHYRPRRVKN RAMLVGDAAG YVTKCSGEGI YFAAKSGRMA
010Thalassiosira_pseudonana_CC  IIKVEAHPIP EHYRPNRVQG RMALVGDAAG YVTKCSGEGI YFAAKSGRMA
011Phaeodactylum_tricornutum_C  IIKVEAHPIP EHYRPRRVQG RIALVGDAAG YVTKCSGEGI YFAAKSGRMA
036Brachypodium_distachyon_Euk  IIRVEAHPIP EHPRPKRVSG RVTLVGDAAG YVTKCSGEGI YFAAKSGRMC
031Selaginella_moellendorffii_  IIRVEAHPIP EHPRPRRVRD RVALVGDAAG YVTKCSGEGI YFAAKSGRMC
021Physcomitrella_patens_subsp  IIRVEAHPIP EHPRPRRVSS RVALVGDAAG YVTKCSGEGI YFAAKSGRMC
015Ostreococcus_tauri_Eukaryot  IIRVEAHPIP EHPRPNRVKG RVALVGDAAG YVTKCSGEGI YFAAKSGRMA
028Micromonas_pusilla_CCMP1545  IIRVEAHPIP EHPRPRRVQG RVTLVGDAAG YVTKCSGEGI YFAAKSGRMA
012Volvox_carteri_f__nagariens  IIRVEAHPIP EHPRPRRCKG RVALVGDAAG YVTKCSGEGI YFAAKSGRMC
013Chlamydomonas_reinhardtii_E  IIRVEAHPIP EHPRPRRCKG RVALVGDAAG YVTKCSGEGI YFAAKSGRMA
007Pyropia_yezoensis            IIKIEAHPIP EHPRPRRCVG RVALIGDAAG YVTKASGEGI YFAAKSGRMA
019Synechococcus_elongatus_PCC  IIKVEAHPIP EHPRPRRVVG RAALVGDAAG YVTKSSGEGI YFAAKSGRMC
035cyanobacterium_UCYN-A_Bacte  VIKVEAHPIP EHPRPRRVVN RVALVGDAAG TVTKSSGEGI YFAAKSARMC
024Thermosynechococcus_elongat  IIKVEAHPIP EHPRPRRVVG RVALVGDAAG TVTKSSGEGI YFAAKSARMC
014Cyanothece_sp__PCC_7425_Bac  IIKVEAHPIP EHPRPRRVVG RVALVGDAAG TVTKSSGEGI YFAAKSARMC
032Acaryochloris_sp__CCMEE_541  IIKVEAHPIP EHPRPRRVVG RVALVGDAAG TVTKSSGEGI YFAAKSARMC
```

```
020Microcoleus_vaginatus_FGP-2  IIKVEAHPIP EHPRPRRVVG RVALVGDAAG YVTKSSGEGI YFAAKSGRMC
030Trichodesmium_erythraeum_IM  IIKVEAHPIP EHPRPHRVRG RVALVGDAAG TVTKSSGEGI YFAAKSARMC
025Lyngbya_sp__PCC_8106_Bacter  IIKVEAHPIP EHPRPRRVVG RVALVGDAAG TVTKSSGEGI YFAAKSARMC
018Cylindrospermopsis_racibors  IIKVEAHPIP EHPRPRRVVG RIALVGDAAG YVTKSSGEGI YFAAKSGRMC
017Fischerella_sp__JSC-11_Bact  IIKVEAHPIP EHPRPRRVVG RVALVGDAAG YVTKSSGEGI YFAAKSGRMC
027Nostoc_azollae_0708_Bacteri  IIKVEAHPIP EHPRPRRVVG RIALVGDAAG YVTKSSGEGI YFAAKSGRMC
023Microcoleus_chthonoplastes_  IIKVEAHPIP EHPRPRRVVG RVALVGDAAG TVTKSSGEGI YFAAKSARMC
026Cyanothece_sp__PCC_7822_Bac  IIKVEAHPIP EHPRPRRVVG RVALVGDAAG TVTKSSGEGI YFAAKSARMC
008Galdieria_sulphuraria        IIKVEAHPIP EHPRPRRVVG RVALVGDAAG YVTKCSGEGI YFAAKSGRMC
002Cyanophora_paradoxa          LIKVEAHPIP EHPRPRRVVD RVALV-DAAG YVTKCSGEGI YFAAKSGRMC
```

```
                                301
009Lotharella_amoeboformis_Euk  ---------- ---------- ---------- ---------- ----------
101Cyanidioschyzon_merolae_Euk  GEAIVRASLN GSRIPTEQDL RGYLRPYDRQ YGPTYTVLDI LQKVFYTNNA
120Emiliania_huxleyi_Localdb__  AEEIVALTAG GTRLPTEMEI KTYLKKFDAA YGPTYLVLDI LQKVFYTNNG
006Isochrysis_galbana           AQEIVALTAG GSRLPTEQEI KTYLKKFDQK YGPTYLVLDI LQKVFYTNNG
001QUERY_ggr                    AQAIVQLMQG GTRLPTQQEI ETYIADYDKK YQATYVVLDL LQKVFYSSNA
003Guillardia_theta             AEAITKLTQG GTVLPTQEEI ETYLRRYDQL YGPTYAVLDV LQQVFYRSNP
004Ectocarpus_siliculosus       AEAVVQCIKQ TGKLPTEAQL KTYLKDYDKL YKPTYLVLDI LQKVFYSNNA
005Aureococcus_anophagefferens  AEAVVKLMKG GTVLPTEEGI TEYIKPYDGL YGPTYTVLDI LQKVFYTNNA
010Thalassiosira_pseudonana_CC  AEAIVKLMDG GKRLPTQNEI ETYLADYDKL YGPTYTVLDI LQKVFYSNNG
011Phaeodactylum_tricornutum_C  AEEIVKLMKN GSVLPTQADI ETYLKKYDNL YGPTYTVLDL LQKVFYVNNG
036Brachypodium_distachyon_Euk  AEAIVAGSAN GTRLVEEGDL RKYLAEFDRL YWPTYKVLDI LQKVFYRSNA
031Selaginella_moellendorffii_  AEAIVEGSEK GVRMINEADL RSYLQKWDTK YWPTYKVLDI LQKVFYRSNP
021Physcomitrella_patens_subsp  AEAIVEGSAN GTRMVDESDL RVYLDKWDKK YWATYKVLDI LQKVFYRSNP
015Ostreococcus_tauri_Eukaryot  AEAVVEQSMN GTKEIDESDL RVYLDKWDRK YWATYKVLDI LQKVFYRSNP
028Micromonas_pusilla_CCMP1545  AEAVVERSEN GTKEVDESDL RLYLDKWDRK YWATYKVLDI LQKVFYRSNP
012Volvox_carteri_f__nagariens  AEAIVEASAN GTKMAGEDAI RVYLDKWDRK YWATYKVLDI LQKVFYRSNP
013Chlamydomonas_reinhardtii_E  AEAIVEGSAN GTKMCGEDAI RVYLDKWDRK YWTTYKVLDI LQKVFYRSNP
007Pyropia_yezoensis            GEALVALSNK GARTPTEADI KSYIKDFDRK YWATYKALDI LQSVFYKNDK
019Synechococcus_elongatus_PCC  AETIVETSNN GARIPTEADL KQYLRRWDKK YGTTYKVLDL LQTVFYRSDA
035cyanobacterium_UCYN-A_Bacte  AEIIVETSNS GRTIPTEDDL KLYLKRWDKQ YGTTYLVLDI LQHIFYRSDA
024Thermosynechococcus_elongat  AETIVETSNG GRRIPTEADL KLYLKRWDKA YGMTYLVLDL LQRVFYRSDA
014Cyanothece_sp__PCC_7425_Bac  AETIVEFSQG GQRVPTEQEL KIYLKRWDRK YGITYKVLDL LQTVFYRSDA
032Acaryochloris_sp__CCMEE_541  AEAIVEFSNN GQRIPTEDEL KIYLKRWDKA YGMTYKVLDI LQRVFYRSDA
020Microcoleus_vaginatus_FGP-2  AETIVEISNK GARIPTEEEI KIYLKRWDKA YGITYKVLDI LQRVFYRSDA
030Trichodesmium_erythraeum_IM  AETIVEASNN GAIIPTEADL KLYLKRWDKK YGMTYKVLDI LQRVFYRSDA
025Lyngbya_sp__PCC_8106_Bacter  AETIVEMSNN GAKIPTEDDL KIYLKRWDKQ YGMTYLVLDI LQRVFYRTDA
018Cylindrospermopsis_racibors  AETIVEVSNS GARIPNEREL KLYIKRWDKK YGLTYKVLDL LQTVFYRSDA
017Fischerella_sp__JSC-11_Bact  AETIVEVSNG GIRIPTEADL KVYLKRWDKK YGLTYKVLDL LQTVFYRSDA
```

```
027Nostoc_azollae_0708_Bacteri  AETIVEVSNN GVRIPTENDL KIYLKRWDKK YGLTYKVLDI LQTVFYRSDA
023Microcoleus_chthonoplastes_  AETIVETSNG GTRIPTEADL KLYLKRWDKK YGITYKVLDI LQTVFYRSDA
026Cyanothece_sp__PCC_7822_Bac  AETIVETSNG GQRIPTEDDL KLYLKRWDKK YGMTYLVLDI LQRVFYRSDA
008Galdieria_sulphuraria        AEAIAEISQQ GERIPSESEL KTYLKRWDQK YWSTYKVLDV LQAVFYRNNI
002Cyanophora_paradoxa          AEAIVETSKN GERVPTEAEL KTYLRRWDQK YWATYKVLDI LQAVFYRSNP

                                351
009Lotharella_amoeboformis_Euk  ---------- ---------- ---------- ---------- ----------
101Cyanidioschyzon_merolae_Euk  AREAFCELCG DDYVQQVTFD SYLYKKVTRT SPVEDVKLLF RTIGSLMRGH
120Emiliania_huxleyi_Localdb__  AREAFVDMCA SDYVQKVTFD SYLYKKVQGN DPINDIKLLF NTIGSVIKGN
006Isochrysis_galbana           AREAFVDMCA SDYVQKVTFD SYLYKKVQGN NPLDDVKLLF NTIGSVIKGN
001QUERY_ggr                    AREAFVEMCD SDYVQKVTFD SYLYKTVQGG NPVDDIKLLF STVGSIFRAQ
003Guillardia_theta             AREAFVEMCE SEYVQKVTFD SYLYKKVTSG GPVEDLKLLF NTVRSLVKQR
004Ectocarpus_siliculosus       AREAFVELCE SEYVQKVTFD SYLYKKVQGN NPVEDLKLLF GTFGSLMRGQ
005Aureococcus_anophagefferens  AREAFVDMCQ SEYVQDVTFQ SYLYKRLVTP NPVEDIKLLF GTVGSLLRGR
010Thalassiosira_pseudonana_CC  AREAFVELCN SKYVQQVTFD SYLYKKVQGN NPLDDIKLLG ETIGCLIKGY
011Phaeodactylum_tricornutum_C  AREAFVELCN SKYVQQVTFD SYLYKRVQGN NPLDDLKLLG ETIGCLIKGY
036Brachypodium_distachyon_Euk  AREAFVEMCA DDYVQRMTFD SYLYKRVVPG NPLEDIKLAV NTIGSLVRAT
031Selaginella_moellendorffii_  AREAFVELCA DEYVQKMTFD SYLYKTVVPG NPLDDIKLAV NTIGSLIRAN
021Physcomitrella_patens_subsp  AREAFVEMCA DDYVQKMTFD SYLYKVVVPG NPLDDIKLAV NTIGSLIRAN
015Ostreococcus_tauri_Eukaryot  AREAFVEMCA DEYVQKMTFD SYLYKTVVPG NPLDDAKLLA NTVASLVRSN
028Micromonas_pusilla_CCMP1545  AREAFVEMCA DEYVQKMTFD SYLYKTVVPG NPLDDMKLLG NTVASIVRAN
012Volvox_carteri_f__nagariens  AREAFVELCE DSYVQKMTFD SYLYKTVVPG NPLDDVKLLV RTVSSLLRSN
013Chlamydomonas_reinhardtii_E  AREAFVELCE DSYVQKMTFD SYLYKTVVPG NPLDDVKLLV RTVSSILRSN
007Pyropia_yezoensis            TKESFVELCD DEYVQRVTFD SYLYKTVTTV NPVEDMKLLF RTVGSLVK--
019Synechococcus_elongatus_PCC  TREAFVEMCD DKDVQRLTFD SYLYKTVVPA NPLVQLKITA KTLGSLLRGN
035cyanobacterium_UCYN-A_Bacte  SREAFVEMCA DKNAQRMTFD SYLYKTVVPM NPLVQMKLTA KTIGSLLRGH
024Thermosynechococcus_elongat  TREAFVEMCS DLDVQKLTFD SYLYKTVVPA NPLVQLKITA KTIGSLMRGN
014Cyanothece_sp__PCC_7425_Bac  TREAFVEMCA DLDVQKLTFD SYLYKTVVPA NPLVQLKITA KTIGSLLRGH
032Acaryochloris_sp__CCMEE_541  TREAFVEMCA DEDVQQMTFD SYLYKTVVPM NPLKQIKITA KTIGSLIRGN
020Microcoleus_vaginatus_FGP-2  TREAFVEMCA DRDVQKLTFD SYLYKTVVPA NPFVQMKITA KTIGSLLRGN
030Trichodesmium_erythraeum_IM  TREAFVEMCA DRDVQKLTFD SYLYKTVVPA NPLIQMKITA KTIGSMLRGH
025Lyngbya_sp__PCC_8106_Bacter  TREAFVEMCS DLDVQKMTFD SYLYKTVVPA NPLVQMKITA KTLGSLLRGH
018Cylindrospermopsis_racibors  TREAFVEMCD DMDVQKLTFD SYLYKTVVPA NPITQLKITA KTIGSLLRGN
017Fischerella_sp__JSC-11_Bact  TREAFVEMCA DLDVQRLTFD SYLYKTVVPA NPITQLKITA KTIGSLIRGN
027Nostoc_azollae_0708_Bacteri  TREAFVEMCD DMDVQRLTFD SYLYKTVVPA NPITQLKITA KTIASLLRGN
023Microcoleus_chthonoplastes_  TREAFVEMCS DLDVQRLTFD SYLYKTVVPA NPLVQLKITA KTIGSLLRGN
026Cyanothece_sp__PCC_7822_Bac  AREAFVEMCS DKDVQRLTFD SYLYKTVVPA NPLIQMKITA KTIGSLLRGY
008Galdieria_sulphuraria        AREAFVELCE DEYVQQMTFD SYLYKTVAKG NPLQDVRLLF HTLGAFARAP
002Cyanophora_paradoxa          AREAFVELCA DEYVQKMTFD SYLYKTVVPG NPISDFKLLV NTVGSLLRGN
```

```
                                                401
009Lotharella_amoeboformis_Euk  ----
101Cyanidioschyzon_merolae_Euk  ALAP
120Emiliania_huxleyi_Localdb__  AIAK
006Isochrysis_galbana           AIAS
001QUERY_ggr                    ATTP
003Guillardia_theta             SDIP
004Ectocarpus_siliculosus       ALAP
005Aureococcus_anophagefferens  AIAT
010Thalassiosira_pseudonana_CC  SIAK
011Phaeodactylum_tricornutum_C  ADAK
036Brachypodium_distachyon_Euk  ALRR
031Selaginella_moellendorffii_  ALRK
021Physcomitrella_patens_subsp  ALRK
015Ostreococcus_tauri_Eukaryot  ALRI
028Micromonas_pusilla_CCMP1545  ALRA
012Volvox_carteri_f__nagariens  ALRS
013Chlamydomonas_reinhardtii_E  ALRS
007Pyropia_yezoensis            ----
019Synechococcus_elongatus_PCC  ALAP
035cyanobacterium_UCYN-A_Bacte  ALAP
024Thermosynechococcus_elongat  ALAP
014Cyanothece_sp__PCC_7425_Bac  ALAP
032Acaryochloris_sp__CCMEE_541  ALAP
020Microcoleus_vaginatus_FGP-2  ALAP
030Trichodesmium_erythraeum_IM  ALAP
025Lyngbya_sp__PCC_8106_Bacter  ALAP
018Cylindrospermopsis_racibors  ALAP
017Fischerella_sp__JSC-11_Bact  ALAP
027Nostoc_azollae_0708_Bacteri  ALAP
023Microcoleus_chthonoplastes_  ALAP
026Cyanothece_sp__PCC_7822_Bac  ALAP
008Galdieria_sulphuraria        ALAP
002Cyanophora_paradoxa          ALAP
```

Alignment: RNABP

```
143Phytophthora_capsici_Locald  ------GVDV FIG------- ---------- ---------- ----------
59Cyanidioschyzon_merolae_Euka  CRAIIVGGHA SIGLYLARQL LSLDLEVYLL GQEEAEAAIS FGDPSNQTRS
57Calliarthron_tuberculosum_Eu  ADVLVLGGHA FLGAHLSSEL LVGGNNKVRV DDKSAKHSFS VEAPSS---L
082Aureococcus_anophagefferens  GHAVIVGGHG EIGYHLAKAL AAKDLDVTIV ASAS--ATVA WCDAAAEKCA
127Emiliania_huxleyi_Localdb__  ADVLVIGGHG EIGFHLAQHL VSRGDAVTIL GDGPSDVKVI WGGEAALALE
144Pyropia_yezoensis_Localdb__  QRCLVIGGTR FSGLYLVQEL LSRGHEVVMY GNTNARTSVI VGDGEERDLA
086Cyanophora_paradoxa_Localdb  RKVLVIGGTR FSGVYLVKAL QEAGHEVTVF GKRASKIKTI VGDPESKQLA
24Synechococcus_elongatus_PCC_  MRILVIGGSR FIGVALVRQL LAAGHAVTVF GSRLAGVEQL VGDPAAAQLQ
26Synechococcus_sp__PCC_7335_B  MRVLVIGGTR FIGVYLTRQL VKQGHAVTLL GNHVDEVETI VCDPEAKQLS
18Leptolyngbya_sp__PCC_6406_Ba  MRILVMGGTR FIGVYLTRLL VEQGHEVVLL GNKVSGVAQI HCDTTAKTLV
22Geitlerinema_sp__PCC_7407_Ba  MRILIMGGTR FIGVYLTRIL VEQGHEVVLF GNKVAGVQQI QGDAGQKELA
02Thalassiosira_pseudonana_CCM  SSALIVGGHG ELGFQLAKNL SSNSKITSIT QDSPNVVKAD FADTASQSLQ
06Phaeodactylum_tricornutum_CC  SAALIVGGHG ELGFQLAKVL SDNDKITSVT QDDPDVVKAS LGDTATQDLK
09Volvox_carteri_f__nagariensi  ANVLIAGGHA FIGLYLAKEL LKKGHKVTIM GDEARDATIA WGDPTKPSYP
10Chlamydomonas_reinhardtii_Eu  SNVLIAGGHA FIGLYLAKEL LKKGHKVTIM GDSERQLNVV WADPAKPSYP
05Micromonas_sp__RCC299_Eukary  QKVLIVGGHA NIGFWLAKTL AAQGHSVTLN GSKTSAVQTV WADPGEATAA
08Ostreococcus_tauri_Eukaryota  KKVLIVGGHA NIGFWLAKTL AGAGHEVTMN GAERSMVTTT WADPADATHA
04Oryza_sativa_Japonica_Group_  KSVLIVGGHA VIGFYLAKDL LAAGHAVTVL GDETSAATTV WGDPADGAVG
03Physcomitrella_patens_subsp_  KKVLIVGGHA VIGFWTAKDL VDAGHSVTIL GEEREIVETV WGEPSDGAVG
07Selaginella_moellendorffii_E  RKVLIVGGHA VIGFWLAKDL VAAGHEVTVM GEERDLVSTV WGDPKDGAVG
090Ectocarpus_siliculosus_Loca  GKALIAGGHG ELGYHLALKL IEKDISVTLL QDGESKATIV WGDFSEGKIE
01QUERY_Bigel_86677             ------HTIA RLRYHLASQL ATDGHAVTLL GDPMGKVTIK YGDACSPGLE
99Lth_N10_86677                 ---------- ---------- ---------- ---------- ----------

                                51
143Phytophthora_capsici_Locald  ---------- ------EAAV VAHIIE---- ------SGLL RTENELP---
59Cyanidioschyzon_merolae_Euka  AGPIAAFIDN RSQTLDEALL LHKFAMLAER YLYVSSCGIY EPGD-YAFIE
57Calliarthron_tuberculosum_Eu  SESYDAVYDN YSKGAGDIKL ALQQAA-GAT VHYVSSAGAY APLA-YGAPS
082Aureococcus_anophagefferens  VDGLTHVYDN FAKSPADAAP MMAAAKSDAF YAFVSSAGMY TAKGL--KEE
127Emiliania_huxleyi_Localdb__  GASFSAVVDN WSKSPEAIAP YATAAKWVSN YAYVSSAGMY TPDKDYGAVG
144Pyropia_yezoensis_Localdb__  GLKFDAVFDN NGRSLEDSAP LVDAAAMVKH YVYMSSAGVY LKSDVMPHVE
086Cyanophora_paradoxa_Localdb  KEEFDVIYDN NGRELTDTKP LADMFKRLEH FVYMSSAGTY LMNDSLPYRE
24Synechococcus_elongatus_PCC_  GRSFDVVFDN TGREAAETQA LVASLDQFQQ LIYVSSAGVY AASDQLPLRE
26Synechococcus_sp__PCC_7335_B  DQSFDAIFDN NGRELAHTKP LADLFKKLKH LVYVSSAGVY AKSDQMPHVE
18Leptolyngbya_sp__PCC_6406_Ba  GQNFDAIFDN NGREQSDTQP LVELFGQVQH LIYVSSAGVY LKSDQMPHVE
22Geitlerinema_sp__PCC_7407_Ba  GESFDAVFDN NGRELSDTQP LVEIFQRLQH FVYVSSAGVY QKSDQMPHVE
02Thalassiosira_pseudonana_CCM  --SYDYVWDN ASKKASAGKA VIDCVKWSKL LTYVSSAGIY KPKDEFPMPE
06Phaeodactylum_tricornutum_CC  DAAFDYVWDN ASKSPKAGQA ICDLAKWVKL FTYVSSAGMY QPTADFPMPE
```

```
09Volvox_carteri_f__nagariensi   RGSFDVVYDN NGKDLSSCQP MIDHFKKVDH YVFVSSAGAY KADSIEPHVE
10Chlamydomonas_reinhardtii_Eu   RGTFDVVYDN NGKDLASCQP LIDHFKKVDH YVFVSSAGAY KADPIEPHVE
05Micromonas_sp__RCC299_Eukary   GAQFDVVVDN NGKDLDSVGP VAAFAKCAKQ FLFVSSAGMY KPTPTPPHLE
08Ostreococcus_tauri_Eukaryota   GAKFDVVVDN NGKDMDTVGP VADFAVAASQ FLFVSSAGIY KPTPCPPHVE
04Oryza_sativa_Japonica_Group_   GASFDVVLDN NGKDLDAVKP VVDWAKAVAQ FLFVSSAGIY TPSDEPPHVE
03Physcomitrella_patens_subsp_   SASFDVVLDN NGKTLDVVQP VADWAKNAKQ FLFISSAGIY KSTFEQPHVE
07Selaginella_moellendorffii_E   GKSFHAVLDN NGKDLDAVKP VVDWAKQVEQ FLFISSAGIY KTSDEPPHVE
090Ectocarpus_siliculosus_Loca   GESFDYVFDN YAKDVDTCKD LADCSKWVKN YAYVSSGGMY KDSDEVPFTE
01QUERY_Bigel_86677              LGEFDAVFDN ISKGKDSCKV AADKAKWVKH YAYVSSAGMY KPGVIFPMSE
99Lth_N10_86677                  ---------- ---------- ---------- ---------- ----------

                                101
143Phytophthora_capsici_Locald   ---------- -----FMQE- ---------- ---------- ----------
59Cyanidioschyzon_merolae_Euka   TDQVRQSAGQ AQVESFLRDS VIPAAFRPMY IIGKAAKLDY TNFFLDRITR
57Calliarthron_tuberculosum_Eu   RVGDSAAGGT VDAEEALRSA GARASFRPIY MYGPSAKRAY LDFFFDRVVR
082Aureococcus_anophagefferens   KKVKDPPTGQ REVELALEAE LAGCAFRPQY IYGPTNKRDY LDWFLNRAAR
127Emiliania_huxleyi_Localdb__   EECAVKSSGQ RQAEEKIAEM GLPSYFRPQY IYGPAQGKSY LAFFFDRITR
144Pyropia_yezoensis_Localdb__   GDATDPKSRH LQTEAYMVDA GIPTSIRPTY IYGALNYNPL EQWFFERLAA
086Cyanophora_paradoxa_Localdb   SDPTDPKSRH KETEAFLKES GQPTSIRPVY IYGPQNYN-V EQWFFHRIHA
24Synechococcus_elongatus_PCC_   SDPVDPQSRH RETENWLQQQ GLPTAFRPVY IYGPGNYNPL EQWFFDRILR
26Synechococcus_sp__PCC_7335_B   GDRVDPNSRH KHTEDYLREQ GIPTAIRPVY IYGPQNYNPL EKWFFDRLVR
18Leptolyngbya_sp__PCC_6406_Ba   GDAVDPNSRH KETEAYLAAQ GVPTAIRPVY IYGPQNYNDL EAWFFDRLVR
22Geitlerinema_sp__PCC_7407_Ba   GDAVDPKSRH RETEDYLATQ GVPTSIRPVY IYGPQNYNPL ESWFFDRIVR
02Thalassiosira_pseudonana_CCM   TTPVKDTAGQ VEYEKYAVEK GLPVSFRPQY IYGESNKWDY IDWYFDRLVR
06Phaeodactylum_tricornutum_CC   TTPIKESAGQ NQFDQYAIQQ GLPVTFRPQY IYGPANKHDY IDWYFDRLVR
09Volvox_carteri_f__nagariensi   GDARKSTAGH VEVEAYLEKA RVPTVFQPLY IYGPNTAKDC EQWFVDRIIR
10Chlamydomonas_reinhardtii_Eu   GDARKSTAGH VEVEAYLEKA RLPTVFQPLY IYGPNTAKDC EQWFVDRIIR
05Micromonas_sp__RCC299_Eukary   GDAVKESAGH AQVEAKLATM PFSASFRPQY FTGYGNNKDC EEYFFDRLVR
08Ostreococcus_tauri_Eukaryota   GDAVKETAGH AVVEAHLKTL PLKSSFRPQY LTGYGSNKDC EEWFFDRLVR
04Oryza_sativa_Japonica_Group_   GDAVKESAGH VGVEKYIAEQ -FGASFRPQY MIGSGNNKDC EEWFFDRIVR
03Physcomitrella_patens_subsp_   GDAVKEDAGH KQVENYLAEL GLEASFRPQY MTGDGNNKDC EEWFFDRIAR
07Selaginella_moellendorffii_E   GDPVKVDAGH VGVEDYISKS AFKSSFRPQY MTGSGNNKDC EEWFFDRIVR
090Ectocarpus_siliculosus_Loca   SSDVKES-GQ RQVEKYVADL GLPTSFRPQY IYGPTNKRDY LDWFFDRVVH
01QUERY_Bigel_86677              SLPVKESAGQ KEVEDYLNSL GLPSSFRPQY IYGPTNKRDY LDYFFDRIVR
99Lth_N10_86677                  ---------- ---------- ---------- ---------- ----------

                                151
143Phytophthora_capsici_Locald   ---IP----- ---SELTHVN HEA----AVQ DGEDREAE-- --VIVRASQI
59Cyanidioschyzon_merolae_Euka   KRPIPLPGKG NAFVSLTHAE DVASMLAAVL ARPDESGQVF NAVSPRYVTL
57Calliarthron_tuberculosum_Eu   KRPVYIPGTG AELTSLTDVR DVAAMMAAAL GKPLDA--VF NAVSPRAITF
```

```
082Aureococcus_anophagefferens   DIPMAVPADA QQPVSLTHCE DVAALMASVV GKEGAADQIF NCGTNKMCSY
127Emiliania_huxleyi_Localdb__   GRPVPVPNGG DQLVTMTHAA DNAAMIAAAV GNEAAVGEAF NCATPSLVTY
144Pyropia_yezoensis_Localdb__   GRPVCVPGHG AHLTGLGHVR DLATAMAATI ETDASVGQVY NIQDRHAVTF
086Cyanophora_paradoxa_Localdb   GRPICVPGHG QHLTGLGHVE DLAVAMANVI GNKKAVGQIY NIAGDRCVTF
24Synechococcus_elongatus_PCC_   DRPLPIPGTG LHLTQLGHVE DLATAMVAAV KNPRAIGQIY NLSGDRYVSF
26Synechococcus_sp__PCC_7335_B   DRPIPIPGSG MALTHLGHCQ DLAAAMVSVL GNDNAVGEIY NISGDKAVTF
18Leptolyngbya_sp__PCC_6406_Ba   DRPIPIPGDG MALTQLGHVQ DLAAAMAAVL SNPRAVGQTY NISGDRAVTF
22Geitlerinema_sp__PCC_7407_Ba   DRPVPIPGNG AHLTQLGHVQ DLAKAMAAVL GNSQAIGQIY NISGDRAVTF
02Thalassiosira_pseudonana_CCM   GEPLPIPGDG SQKVSLTNSE DVASLLASVL NDESAVGQFF NCGTDQLVTY
06Phaeodactylum_tricornutum_CC   ELPLPIPGDG TQKLSLTNAE DVASLLAAPL NDEAAIAQVF NCGTDQLVSY
09Volvox_carteri_f__nagariensi   DRPVPIPSPG IQLTSLTHVE DVAAMLALVP GNREAIGQMY NVCSDRCISF
10Chlamydomonas_reinhardtii_Eu   DRPVLLPAPG VQLTSLTHVE DVASMLAAVP GNRAAIGQHY NVCSDRCITF
05Micromonas_sp__RCC299_Eukary   GRPVLVPGSG DQLSVVAHAE DVATMMAAAV GNPAANGVIF NAVTNKAVTL
08Ostreococcus_tauri_Eukaryota   GRPVLVPGSG DQLSSVTHAE DLATMIAAAI GNDGAAGEIF NCVMPKAVTL
04Oryza_sativa_Japonica_Group_   GRPVPIPGSG MQVTNISHVR DLASMVALAV ESPGAAGRIF NCVSDRAVTF
03Physcomitrella_patens_subsp_   GRPVPIPSPG IQVTNISHVR DLSSMLTLAV GKPEANGSIF NCVSDRGTTF
07Selaginella_moellendorffii_E   DKPVPIPSPG IQVTNIAHVS DLSSMITLAI GKPSANATIF NAVSDRAVTF
090Ectocarpus_siliculosus_Loca   GLEIPLPLHG DQLVALTHAE DVASMLASVV -GNERVKQVF NCASDRYITY
01QUERY_Bigel_86677              GRPVPVAGNG QQLVTLTHAA DVASMLGSVL DAGEKHMKVF NCATDQLITV
99Lth_N10_86677                  ---------- ---------- ---------- ---------- ----------

                               201
143Phytophthora_capsici_Locald   DTSVQLSQGT MQD------- ---------- -----LFGSP TPSEVEL---
59Cyanidioschyzon_merolae_Euka   KGLAEMCHRV VHGDSKPIYY DPVKLATGLP FRVSYFIADP GKAMRLLAWQ
57Calliarthron_tuberculosum_Eu   DGVAQIVASV VGHE-AIVHY DPADNKKVFP FRIRHFFADP KEAMAELGWE
082Aureococcus_anophagefferens   DDVCIAAAKA LGKSEALVAL PPDTK-SSFP FRPNAFAVRV RKAMDVLEWP
127Emiliania_huxleyi_Localdb__   DDLVRLCAGA AGKEE--IHY DPAGFKYKFP FRDTPFYVSA DKASRLLGFS
144Pyropia_yezoensis_Localdb__   DGVARTAATA MGMDASVLHY NPKDFNKAFP FRPQHFFTSP NKALRELDWS
086Cyanophora_paradoxa_Localdb   DGLVKACAEA AGKPPEI--- -------AFP FRQQHFFASI QKAEDELN-S
24Synechococcus_elongatus_PCC_   DGLARACAIA AGRDQALLHY DPKQLRKAFP MRAQHFITAI DQARQDLEWV
26Synechococcus_sp__PCC_7335_B   DGLARACAIA MEKDDAVIHY NPKDFKKAFP MRVQHFFTDI SKAKAELDWQ
18Leptolyngbya_sp__PCC_6406_Ba   DGLARACAIA AGKDQALLHY DPKQFRKAFP MRVQHFFTAI DRAKADLGWQ
22Geitlerinema_sp__PCC_7407_Ba   DGLARACAAA AGKDGTLLHY DPKAFRKAFP MRVQHFFTSI DKAKHELGWQ
02Thalassiosira_pseudonana_CCM   DEVALMCAEV AGVMAK-IHY D-DSLKAKFP FRLTDFYVSP DMAKAKLGWE
06Phaeodactylum_tricornutum_CC   DEVAYLCAEA AGIDDKVIHY DADMFKATFP FRMTDFYVAP DTAKEKLGWS
09Volvox_carteri_f__nagariensi   VGICKSVAKA LGKEANIVLY SPEKVKEGFP FRTVHFFASS DKAKRELGWK
10Chlamydomonas_reinhardtii_Eu   TGIAKAIGKA LGKDPEIILY SPEKVKEGFP FRTVHFFASA DKAKRELGWK
05Micromonas_sp__RCC299_Eukary   NGMVQLCAAA AGVEPKIVNY DPKKLKKAFP FRPIHFYSYP ANALKLLDWQ
08Ostreococcus_tauri_Eukaryota   NGMVELCAAA AGVEAKIINY DPKDVKKAFP FRPIHFYSSS AKAQKVLGWS
04Oryza_sativa_Japonica_Group_   NGLVKMCAAA AGAQPEILHY DPAAVKKAFP FRNMHFYAEP RAAKEVLGWR
```

```
03Physcomitrella_patens_subsp_  DGLVKMCAKA AGKEAKIVHY DPKAIKKAFP FRNMHFYAEP RAAKTKLGWE
07Selaginella_moellendorffii_E  DGLVRLCARA AAKEAKIVHY DAKALKKAFP FRNMHFYAEP RAAKEKLGWT
090Ectocarpus_siliculosus_Loca  NGLFREVGKV AKPASKMAYY EPRDYKGWFP FRNNHFVVNS EKAKRLLGWS
01QUERY_Bigel_86677             DDLIHVCAKI AGVPPRIVHY DPKKVKKAFP FRDSNFFVAP DRAKAELGWS
99Lth_N10_86677                 ---------- -----QIVHY DPKKVKKAFP FRDSHFFVAP NKAKEDFGYK

                                251
143Phytophthora_capsici_Locald  ----SQGAVR AFQDDAQD-G IPERDV---I EQHLSGA
59Cyanidioschyzon_merolae_Euka  PRHDLQNDLE MY-EEYLELG LHERRVDLTN DDRIARS
57Calliarthron_tuberculosum_Eu  PKYGDLGAMG SFYEEYKSLG LHEKELKFDL EDAIARA
082Aureococcus_anophagefferens  ATHDVLADLG FYTEDFLALG LDKGDLDTSK DENMVQD
127Emiliania_huxleyi_Localdb__  PAHSIADDIW YYSEQYVAKG GLDKELDFAE DDVVLGR
144Pyropia_yezoensis_Localdb__  VQYELASGWD TYENDFVPKA QGKLKNDWVT DDMILGR
086Cyanophora_paradoxa_Localdb  PKYDLVAGLD SY-NDYVHRT V---ALDFET DDIILGK
24Synechococcus_elongatus_PCC_  PRFSLIDGLN SLQNDYLARG LDQQAVDFSL DEEILAA
26Synechococcus_sp__PCC_7335_B  PQFSLIDGLD SYENDYLANN LHKAEIDFSL DDQILS-
18Leptolyngbya_sp__PCC_6406_Ba  PQFNLVNGLD AFEKDYLVSG RHQQTIDFDL DDQILAA
22Geitlerinema_sp__PCC_7407_Ba  PEFDLVGGLD SFQQDYLATH QDQREVDFST DEEIVAA
02Thalassiosira_pseudonana_CCM  AKHSLKEDLT WYFDSYKARG GPAKEMTFVE DKEVLV-
06Phaeodactylum_tricornutum_CC  PLHSLKDDLS FYYESYVARG GPTKKMSLIK DWEITVG
09Volvox_carteri_f__nagariensi  PKHDFQKDVA ALVADYKAQG RDKKDVDFSI DDKILEA
10Chlamydomonas_reinhardtii_Eu  PKHDFQKDVQ GLVNDYKANG RDKKEVDFSV DDKILAA
05Micromonas_sp__RCC299_Eukary  PKHDLAADLK ERFEFYKASG RANKDMSFEL DDKILAS
08Ostreococcus_tauri_Eukaryota  PKHDLGAELK ERFAYYKSTG RDAKEMAFEV DDKILAA
04Oryza_sativa_Japonica_Group_  SSTNLPEDLK ERFAEYASSG RGQKEMSFDL DDKIIAA
03Physcomitrella_patens_subsp_  SKTNLAEDLK ARWEDYVKIG RDKKDIKFEL DDKILEV
07Selaginella_moellendorffii_E  SSTNLSEALK ERYEEYIKIG RDKKDIKFEI DDTILSK
090Ectocarpus_siliculosus_Loca  PKHTITDDLA EYFEGYKAAG KVEAEPNFNK DKQYQDM
01QUERY_Bigel_86677             CQHDLEKELK AYFEGYRALG KTEKDMSFPI DDTILGQ
99Lth_N10_86677                 CEHDLVKELK AYYEGYKAIG KDQKEMSFPI DDQILNQ
```

Alignment: RPS22

```
44Calliarthron_tuberculosum_Eu  TAIRITGNNV AMTDSLRTYV REKLRKVIRK HASLLTKIDV HLAVEHNPAI
46Porphyridium_cruentum_Eukary  ARISISGTNI VSYRTSRDRV RSKVGRSIGK YDQMVIRADV HLLVAKNPAI
65Emiliania_huxleyi_Localdb__L  TKLDVTSSKL PLTPALRDAT DEKLGKQLDR YDKYLNSVSV NLKVHDREHV
48Aureococcus_anophagefferens_  SMVLSDGNNL KMTDSMRDYV EDKIGTVISK FAGVAQRCDT HLSVMRNPAV
01QUERY_Bigel_rps22_51185       --MVMTGNNI ELTESIAAYT NDKLGQMLKK YKDFVKSTEI HFTINKNPAV
42Lotharella_amoeboformis_Euka  ---------- ---------- -------LKK YKEFVKSCEI HFTINKNPSV
38Brachypodium_distachyon_Euka  ARLIMQGRNV KLTEKLKEHI EEKVGRAVSN HSHLVREVDV RLSAGKGPKA
37Selaginella_moellendorffii_E  IRLVIQGKHL ELTEALKAYV EDKVGKAVHN HAYLVMEVDV RLSVGRGQKL
41Physcomitrella_patens_subsp_  VRLVIQGKHL ELTDAIKQYV EEKVGNAVHN QSALVKEVDV RMSVGRGERL
77Galdieria_sulphuraria_Locald  KLIVVTGNNI ELTEALHKYV EEKVGKAILK YIHFVVKVEV HLSVAHNPSI
45Cyanidioschyzon_merolae_Euka  TPITVTGDNI DLTSALREHV LDKIGHVVEK FPGLVTRVEV HLSVAHNPRI
49Cyanophora_paradoxa_Localdb_  MAFVLHGGGI ELTEAIRAYV EEKIGHSVET FKNFVTGVDV HLQVSKNPRV
33Prochlorococcus_marinus_str_  MKILIHGKNI ELTGALKEYT EAKIEKATHH YKDIVKEADI HLSIEKNPRL
31Prochlorococcus_marinus_str_  MKLLIHGRNL ELTQSLRDYT KTKIDKATHN FQEMVQEADV HLSVARNPRV
20Synechococcus_sp__RCC307_Bac  MKLLIHGRNL EVTPALRDYT ETKLSRAVSH FNGMVQEADV HLSVARNPRV
39Synechococcus_sp__WH_7803_Ba  MKLLIHGRNL DVTPALRDYT ETKLDRAIHN FGDLVKEADV HLSVARNPRV
27Synechococcus_sp__WH_8102_Ba  MKLLIHGRNL EITPALRDYT QTKLERATHH FGNAVREADV HLSVARNPRV
05Prochlorococcus_marinus_str_  MKLLIHGRNL ELTPALREYT QSKLERAIQH FEDMVKEADV HLSVARNPSV
08Synechococcus_sp__RS9917_Bac  MKLLIHGRNL ELTPALRDYT QSKLERAVQH FDDMVKEADV HLSVARNPRV
34Gloeobacter_violaceus_PCC_74  MQFAIHGKNI EVTEPIRDYV SSKLDKVFAH FDQLTMGVDV YLSVARNPRI
24Chamaesiphon_minutus_PCC_660  MKLVLQSKNI EITDAIRDYV NQKIEKAVSH FEQITTEIDI NLSVAKNPRI
16Halothece_sp__PCC_7418_Bacte  MKLLIQGNNI EVTEAIRNYV QEKLENAVKH FQQITSKVDV HLSVAPNSRV
29Synechococcus_sp__JA-2-3Ba_2  TKLVIQGKNV EITDAIRSYV QEKIERAVSH FAQITSEVDV NLSVARNPRI
13Stanieria_cyanosphaera_PCC_7  MKLLIQGNNI TVTDAINDYV HNKLEKAVKH YQNITTKVDV HLSVAKNARV
03Cyanothece_sp__PCC_8802_Bact  MKLLIQGNNI DVTESIHDYV QEKLEKAVKH FQSITTKVDV HLSVARNARI
02Cyanobacterium_aponinum_PCC_  MKLLIQGNNI EVTESIHDYV EEKLEKAVKH FQNLATKVDV HLSVARNARI
10Gloeocapsa_sp__PCC_73106_Bac  MKLLIQGNNI VVTDAIHDYV QQKLERAVKH FQALTTKVDV HLSVAKNDRI
07Cyanothece_sp__PCC_7424_Bact  MKLLIQGNNI TVTEAIHDYV QQKLEKAVKH FQTLTTKVDV HLSVAKNARI
06Synechocystis_sp__PCC_6803_B  MKLLIQGNNI TVTEAIHDYV EEKVERAVKH FQNLTTKVDV HLSVARNARI
09Pleurocapsa_sp__PCC_7327_Bac  MKLLIQGNNI TVTEAIHDYV EQKLEKAVKH FQGMTTKVDV HLSVARNARI
04Oscillatoriales_cyanobacteri  MKLVIQGKNI EITDSIREYV NQKIEKAVNH FQTMITEVDV HLSVARNPRI
36Rivularia_sp__PCC_7116_Bacte  MKLVIHGKNI EITDAIREYV HQKIEKAANH FHHITNEVDV HLSVAKNPRI
22Synechocystis_sp__PCC_7509_B  MKLVIHGKNI ELTDAIREYV HQKIEKAVNH FQSLTTEVDV HLSVARNPRI
19Cyanothece_sp__PCC_7425_Bact  MKLVIHGKNI EITDAIRDYV QQKIEKAANH FQNLTTEIDV HLSVARNPRI
17Microcoleus_sp__PCC_7113_Bac  MKLVIQGKNI EITDAIREYV HQKIEKAVNH FQNMTTEVDV HLSVARNPRI
21Oscillatoria_acuminata_PCC_6  MKLVIHGKNI EITEAIREYV NQKVEKAVSH FQNLTTEVDV HLSVAKNPRN
30Oscillatoria_sp__PCC_6506_Ba  MKLVIQGKNI EITDAIREYV NQKIDKAVNH FQTLTTEVDV HLSVARNPRI
28Calothrix_sp__PCC_6303_Bacte  MKLVIHGKNI EITDAIREYV HQKIEKAVSH FQTLTTEVDV HLSVARNNR-
```

```
23Chroococcidiopsis_thermalis_  MKLVIHGKNI EITDAIREYV NQKIEKAVNH FQTLTTEVDV HLSVARNPRI
11Gloeocapsa_sp__PCC_7428_Bact  MKLVIHGKNI EITDGIREYV HQKIEKAVNH FQNLTNEVDV HLSVARNPRI
12Fischerella_sp__JSC-11_Bacte  MKLVIHGKNI EITDAIREYV HQKIEKAVSH FQNITNEVDV HLSVARNPRI
32Nostoc_punctiforme_PCC_73102  MKLVIHSKNI EITDAIREYV HQKIEKAASH FQSITNEVDV HLSVARNPRI
25Calothrix_sp__PCC_7507_Bacte  MKLVIHGKNI EITDAIREYV HQKIEKAVNH FQNITNEVDV HLSVARNPRI
26Anabaena_variabilis_ATCC_294  MKLVIHGKNI EITDAIREYV HQKIEKAVNH FQNITNEVDV HLSVARNPRI
14Nostoc_sp__PCC_7107_Bacteria  MKLVIHGKNI EITEAIRDYV HQKIEKAVSH FQNITNEVDV HLSVARNPRI
18Nostoc_sp__PCC_7524_Bacteria  MKLVIHGKNI EITDAIREYV HQKIEKAVNH FQNITNEVDV HLSVARNPRI
51Ectocarpus_siliculosus_Local  APIIVTGNNV EVTEPLKEYV EKKMAKVLDK VGSSVSKVDV HLSVNKNPSV
35Phaeodactylum_tricornutum_CC  VPVIINGQNI ELTPALVEHV NKRIGGQINK LASAIQECDV ILSVSKNPKV
15Thalassiosira_pseudonana_CCM  APIVVTGDNI DLTPALSDYV NSKIEKTVGK LPLAVSHCDV YLTVNKNPKV

                                51
44Calliarthron_tuberculosum_Eu  ARHKAEVVAF AGKTILRAEV RAVDMYAAID LVEERVGRTL RKYKERKAGR
46Porphyridium_cruentum_Eukary  NGHSAEVVVQ VKGNVIRAET KSENMYASID AVTDKLTRTL RKYKERRNSH
65Emiliania_huxleyi_Localdb__L  EAHIAEVTAL CRDQVIRVRH ESEDMYASLD LLADQLSRKL RKYKERRGGT
48Aureococcus_anophagefferens_  DSDVAEVVVF CKGEVVRAEE RSPSMYSSID LVADKVARKL RKVKERKEGK
01QUERY_Bigel_rps22_51185       NAHKIEVTVY VKDKVIRGSV SSVSEYSSID LVTDLVNRKL RKYKERKEGK
42Lotharella_amoeboformis_Euka  NAHKVEVTVY VKDKVMRGTV SSISEYSSID LVTDLVNRKL RKYKERKFDK
38Brachypodium_distachyon_Euka  --SRCEITLF TKRGVVRAEE EEESSYASID VAAAVVKRKL RKIKEKE-TD
37Selaginella_moellendorffii_E  --QKCEVTMF TKKGVVRAEE ATDSMYSAID RVSDVVARKL RKIKEKD-GH
41Physcomitrella_patens_subsp_  --QRCEVTLF TKKGVVRAEE EAESMYASID RVSDVISRKL RKIKEKDGGH
77Galdieria_sulphuraria_Locald  LRHTSEVTVF ARKHVLRASE TAETMYAAID LVSDITERKL QRYKERLHIH
45Cyanidioschyzon_merolae_Euka  NAHDCEVTAY ARGRVLRASV KMENMYAAID LTADKLKRTL RKYKERLHDR
49Cyanophora_paradoxa_Localdb_  FTQSAEVTVY AQGQVLRASE RTENLYASID LVSEKIGRNL RKYKEKRQDK
33Prochlorococcus_marinus_str_  SYQTAEVTIF ASGTIIRAEE KTENLYSSID LVSNKLCRKL RKYKERHYKN
31Prochlorococcus_marinus_str_  PQQTAEVTVF ANGTVIRAQE RSENLYASID LVANKLARQL RKYKERHNSH
20Synechococcus_sp__RCC307_Bac  PQQTAEVTLF ASGTVIRAQE RSQNLYASID LVAGKLARQL QRHKDRRQGH
39Synechococcus_sp__WH_7803_Ba  PQQTAEVTVF ANGTVIRAQE RSENLYASID LVASKLARQL RRFKDRHSDH
27Synechococcus_sp__WH_8102_Ba  PQQTAEVTVF ANGTVIRAQE RSENLYASID LAASKLARQL RRWKERHSDH
05Prochlorococcus_marinus_str_  PQQTAEVTVF ANGTVIRAQE RSENLYASID LVASKLCRQL RRYKERHSDH
08Synechococcus_sp__RS9917_Bac  PQQTAEVTVF ANGTVIRAQE RSENLYASID LVASKLCRQL RRFKERHSDH
34Gloeobacter_violaceus_PCC_74  RSHSAEVTVQ ASGTIIRAEE ETENLYASID LVADKLHRQM RKYKERMQKK
24Chamaesiphon_minutus_PCC_660  AAQVAEVTIY VNGAVIRAEE ASEHLYASID LVADKISRKL RKYKERNFTK
16Halothece_sp__PCC_7418_Bacte  DRTKAEVTVH ANGKVIRAQE QSGDLYASID LVADKIARQL RKYKEKHLHK
29Synechococcus_sp__JA-2-3Ba_2  ASQSAEVTVY ANGTVIRAEE SSENLYASID RVADKLARKL RKYKERNGIH
13Stanieria_cyanosphaera_PCC_7  NKHKAEVTVY ANGTVIRAQE GSENLYASID MVSDKIARQL RKYKEKLLDQ
03Cyanothece_sp__PCC_8802_Bact  NKHKAEVTVY ANGTVIRAQE GSESLYASID LVSDKIARQL RKYKEKLNNK
02Cyanobacterium_aponinum_PCC_  NKHKAEVTVY ANGTIIRAQE HSENLYASID LVSDKITRQL RKYKERNLAK
10Gloeocapsa_sp__PCC_73106_Bac  DKHKAEVTVY ANGTVIRAQE GSENLYASID LVADKIARQL RKYKERKLEK
```

```
07Cyanothece_sp__PCC_7424_Bact  NKHKAEVTVY ANGTVIRAQE GSENLYASID LVSDKIARQL RKYKERKLEQ
06Synechocystis_sp__PCC_6803_B  NKHKAEVTVY ANGTVIRAQE GSENLYASID LVADKIARQL RKYKER-IQD
09Pleurocapsa_sp__PCC_7327_Bac  DKHKAEVTVY ANGAVIRAQE GSENLYASID LVSDKIARQL RKYKER-LQD
04Oscillatoriales_cyanobacteri  SKQVAEVTIY VNGSVVRAEE GSENLYASID MVADKISRQL RKYKEKRYDK
36Rivularia_sp__PCC_7116_Bacte  PNQTAEVTIY ANGNIIRAEE SSENLYASID LVANKIARQL RKYKERRRDK
22Synechocystis_sp__PCC_7509_B  SKQSAEVTIY ANGSVIRAEE SSESLYASID LVADKIARQL RKYKERRQAN
19Cyanothece_sp__PCC_7425_Bact  PKQTAEVTIY TNGSVIRAEE SSENLYASID LVSDKIARQL RKFKERRQDK
17Microcoleus_sp__PCC_7113_Bac  SKQTAEVTIY ANGTVIRAQE GSEDLYASID LVADKIQRQL RKYKEKLQHK
21Oscillatoria_acuminata_PCC_6  SKQTAEVTIF ANGTIIRAEE SSENLYASID LVADKIARQL RKYKEKRHAQ
30Oscillatoria_sp__PCC_6506_Ba  TKQTAEVTIY ANGSVIRAEE SSESLYASID LVADKIARQL RKFKEKRQDK
28Calothrix_sp__PCC_6303_Bacte  PVQAAEVTIY ANKNVIRAEE SSENLYASID LVADKISRQL RKYKERLQNK
23Chroococcidiopsis_thermalis_  PKQAAEVTIY ANGTVIRAEE SSENLYASID LVADKIARQL RKYKEKLQDK
11Gloeocapsa_sp__PCC_7428_Bact  DRQAAEVTIY ANGSVIRAEE SSENLYASID MVADKIARKL RKYKEKRHDK
12Fischerella_sp__JSC-11_Bacte  PKQAAEVTIY ANGNVIRAEE SSENLYASID LVADKIARQL RKYKERRHDK
32Nostoc_punctiforme_PCC_73102  TRQAAEVTIY ANGSVIRAEE SSENLYASID LVADKIARQL RKYKERRQDQ
25Calothrix_sp__PCC_7507_Bacte  TRQAAEVTIY ANGSVIRAEE SSENLYASID LVADKIARQL RKYKERRQDK
26Anabaena_variabilis_ATCC_294  TRQAAEVTIY ANGSVIRAEE SSENLYASID LVADKISRQL RKYKERKQDQ
14Nostoc_sp__PCC_7107_Bacteria  PKQAAEVTIY ANGSVIRAEE SSENLYASID LVADKIARQL RKYKERRQDK
18Nostoc_sp__PCC_7524_Bacteria  PKQAAEVTIY ANGSVIRAEE SSENLYASID LVADKISRQL RKYKERRQDK
51Ectocarpus_siliculosus_Local  DNHNTEVTVF SKNHVIRATD NSDNMYASVD QVTDRIQRKL RRFKERKVGR
35Phaeodactylum_tricornutum_CC  NGHRVEVVTN LKGTSIICHN ESPDMYASID AASHALYRKL CKYKDNRVGG
15Thalassiosira_pseudonana_CCM  DAHTAEVVTT LKGTTFRVAE SSPDMYASID LVAARLARKL RKYKERRLGG

                               101
44Calliarthron_tuberculosum_Eu  SRK------- ---------- ---------- AIEKNSDLAS APVDGGEALD
46Porphyridium_cruentum_Eukary  DGPHTADLGE ESADEYVDDQ EGAKAL---- HMVEREVIPL PKMTVQEAVT
65Emiliania_huxleyi_Localdb__L  AEAAEEDEAE SLAQFAAATD ASDAAASMPD EVVREKKFAM PPISVEEAVV
48Aureococcus_anophagefferens_  RSKPKLKLAE AISTALEEEL FDA------- TLIRKKKYPM PEQSLVDAMV
01QUERY_Bigel_rps22_51185       RTATFYTISD SSVE-GAAVD VQQEYSGIPE SVVRRKKFPM PPISIEDATL
42Lotharella_amoeboformis_Euka  RASTFYRISD SSSDIGAEAD V-QEFQPVPE AVVRKR---- ----------
38Brachypodium_distachyon_Euka  VRHLKGSWQT AAAAFSESDV DEEDEDELVE KVVRTKVFEM APLTVDEALE
37Selaginella_moellendorffii_E  GR--RLPVGE ILRESSTAPP TPAEEPDFPD EIVRTKIFDM PPLKINDAMD
41Physcomitrella_patens_subsp_  GRPAKYSIGE VLNEVVDLDP ILEKPDDLPD EVVRTKYFEM RPMKPLEALE
77Galdieria_sulphuraria_Locald  GPKAVNPETD LVRRPSRGDL VDKSSDNLPE DVVKRKSFPV PRQTVAEAVL
45Cyanidioschyzon_merolae_Euka  RTRSAPVMDG LAVPATSPVP VDDGAPELPS RVVRKKRFPM PLQTVEEAVL
49Cyanophora_paradoxa_Localdb_  HPR------- -ADSPSPAPG VDEEVFAAPA PVVKKKQFPM PAMTVDDALE
33Prochlorococcus_marinus_str_  LNYKNKHTNT NELDSIDKNI FNDREAKLPE PSIKNKYFEM IPISLEEARK
31Prochlorococcus_marinus_str_  NVHTKSVEDT Q-FSSSDHSL TEGKEPHLPS PGVRRKYFEM TPMNIEQARV
20Synechococcus_sp__RCC307_Bac  GHHAEP---- --VMPLQGDM LQGRHAELPA PVVRRKYFAM PPMSVEEAIH
39Synechococcus_sp__WH_7803_Ba  HHSASETTES VLDEAIVESL LDGKEVQLPS PGVRRKYFSM PPMTLEQARQ
```

```
27Synechococcus_sp__WH_8102_Ba  HHSASTTTEA VTDSQVDGSL LQGREAELPD PGVRRKYFAM PPMSLEEARR
05Prochlorococcus_marinus_str_  HHSASSTTEA VIEAPIDGSL LDGKEAHLPS PGVRRKYFAM PPMSVEQARH
08Synechococcus_sp__RS9917_Bac  HHSASETTDA IAAAPIDGSL LDGKEVQLPD PGVRRKYFAM PPMSLEEARH
34Gloeobacter_violaceus_PCC_74  PKPKTGL--A VAQAPISLPN GDEKATALPK EVVRTKYFAM PPMSPEEALD
24Chamaesiphon_minutus_PCC_660  KDRTEGLIGE ELLELLDYNL TDNRSATLPA EVVRTKYFAL SALTIEEAMA
16Halothece_sp__PCC_7418_Bacte  KTHDSPKTAE TVPQPVDEDL VNDRAPELPD DVVRVKYFAM PAMTVMEAKD
29Synechococcus_sp__JA-2-3Ba_2  RVRHTPKTSV AVQQPVTDPL NTERQAELPA EVVRTKYFAM PPMTVQEALH
13Stanieria_cyanosphaera_PCC_7  KTQTATKTGE LVEGTVANDL FSDRSPELPN EVVRMKYFSM PPMTIEEALQ
03Cyanothece_sp__PCC_8802_Bact  KAPNPVKVGE VTATAIEGDL IGDRTPELPA EVIRMKYFAM PPMTIDEALH
02Cyanobacterium_aponinum_PCC_  KTHTAEKTSD AVEKTVGTDL IGDREPELPS EVVRMKYFAM TPMTIDQALE
10Gloeocapsa_sp__PCC_73106_Bac  KTNAHVKVTD VVEEPVTANL IGDRTPELPA EVVRMKYFTM PPMTIEEALD
07Cyanothece_sp__PCC_7424_Bact  NAHSPVKAGE VVQETIEENL IGDRQPELPP EVVRMKYFGM PPMSIDEALE
06Synechocystis_sp__PCC_6803_B  KQHGNVKTSE IVDKPVEENL IGDRAPELPS EVLRMKYFAM PPMAIEDALE
09Pleurocapsa_sp__PCC_7327_Bac  KKVASVKAGE IVEKPVSETL IGDRTPELPP EVVRMKYFAM PPMSIEEALE
04Oscillatoriales_cyanobacteri  YHPSVKTAEV LSDQPVVTEL PTNRTPELPA QVVRTKYFAM PPMSVQEALE
36Rivularia_sp__PCC_7116_Bacte  KIHDRTNEEI I-PEEMVTDL IGDRTPELPE EVVRTKYFAM PPMTVSQALE
22Synechocystis_sp__PCC_7509_B  KASVETTVDS EEQIPLVSDL IGDRTPQLPA EVVRTKYFAM PPMTVSEALE
19Cyanothece_sp__PCC_7425_Bact  HSRSK--EEP VVSVPLVTDL TGDRTPELPE EVVRTKYFAM PPMTLREALE
17Microcoleus_sp__PCC_7113_Bac  KTHDQPKTGI VVQVPVPDDL IGDRTAELPS DVVRMKYFAM PPMTMEEALE
21Oscillatoria_acuminata_PCC_6  KVQAPDTNEA LLEQPLVEDL IGERTPELPE EVVRTKYFAM PPMSVLEALE
30Oscillatoria_sp__PCC_6506_Ba  THTNPKVGDV VAEEPVVSDL IGDRTPELPS EVLRSKYFAM PPMTMQEALE
28Calothrix_sp__PCC_6303_Bacte  KNNLVENSEV VEEIPIVNDL IGSRTPELPE EVVRTKYFAM SPMTISEALE
23Chroococcidiopsis_thermalis_  KTQPKTSLEA V-EPPVVNDL IGDRTPELPS EVVRTKYFAM PPMTLNEALE
11Gloeocapsa_sp__PCC_7428_Bact  KTQVPEKTSI AVPAPVVEDL IGDRTPELPE EVVRTKYFAM PPMTITEALE
12Fischerella_sp__JSC-11_Bacte  KTHAQTGIEG VVQQTVVTDL IGNRAPELPE EVVRTKYFAM PPMTVTEALE
32Nostoc_punctiforme_PCC_73102  KTQSQPT-EV VVAESVVPDL IGDRTPELPN EVVRTKYFSM PPMTLAEALE
25Calothrix_sp__PCC_7507_Bacte  ITQSPPTTDV LVPEPVAADL IGDRTPELPN EVVRTKYFSM PPMTLAEALE
26Anabaena_variabilis_ATCC_294  KTQALPTTET IVPETVVADL IGDRTPELPE EVVRTKYFSM PPMTVTEALE
14Nostoc_sp__PCC_7107_Bacteria  KTHAQSTTEV VVPEPVVPDL IGDRTPELPS EVVRTKYFSM PPMTLAEALE
18Nostoc_sp__PCC_7524_Bacteria  KTQAQPTNEV VVPQPVVADL IGDRTPELPE EVVRTKYFSM PPMTLAEALE
51Ectocarpus_siliculosus_Local  SGVEKTADMD AAAEAADEDP FEDKYGDEPE MVVKTKSFPM PAISLEEAVT
35Phaeodactylum_tricornutum_CC  EKMNDIMLEN LEVSEYTATP VAEEYVD-PE PITKVKNFDT KPMSVQEAVF
15Thalassiosira_pseudonana_CCM  PNMENIALNA IEVDESEDGA EEESYID-PD AVTKVK---S TPISIKEAIF

                             151
44Calliarthron_tuberculosum_Eu  SAD------- ---EEEEDGL SDVYANGTSN IGVLPPV
46Porphyridium_cruentum_Eukary  VLAF-QTNFH VFREVSSNKI CVIFKKEGGG IGLVEPE
65Emiliania_huxleyi_Localdb__L  CLEYIEHDFY AFRNAATGEV NVVYKRDEGG LGWISPS
48Aureococcus_anophagefferens_  CMDGLDHDFY MFKSEETGQI NVIYKR---- -------
01QUERY_Bigel_rps22_51185       CLDYLDHDFY VFRNSETKEI NVVYRRAEGG VGLIEPE
```

```
42Lotharella_amoeboformis_Euka  -------DSR CHRSP----- ---------- -------
38Brachypodium_distachyon_Euka  QLENVDHDFY AFRNEQTGEV NILYKRKEGG FGLIIPK
37Selaginella_moellendorffii_E  HMANIGHDFY AFRNAESGEV NILYKRKEGG YGIIIPR
41Physcomitrella_patens_subsp_  QLVNVGHDFY AFRNVESGEI NILYKRTHGG YGIIVPR
77Galdieria_sulphuraria_Locald  CLEYIDHDFY VFRNSETGEV NIVYRRNHGG VGLIVPE
45Cyanidioschyzon_merolae_Euka  CLDYIDHDFY VFRNADTGEV NVVYRRRHGG VGLIEPE
49Cyanophora_paradoxa_Localdb_  CLGYLDHDFY VFRNKATNEI NVVYERHEGG YGLIEPE
33Prochlorococcus_marinus_str_  QLDLIDHDFY VFRNKKNNEL QVIYKRKHGG YGLIQSK
31Prochlorococcus_marinus_str_  QLDLIDHDFY LFREEEGSAL RVIYKRNHGG YGVIQEK
20Synechococcus_sp__RCC307_Bac  QLEVIDHDFY VFRNADNGEL QVVYQRNHGG YGVIEAR
39Synechococcus_sp__WH_7803_Ba  QLDLIDHDFY LFRDSASGDL QVIYRRNHGG YGVIEAR
27Synechococcus_sp__WH_8102_Ba  QLDLIDHDFY LFRDKDSDQL QVIYRRNHGG YGVIQAR
05Prochlorococcus_marinus_str_  QLDLIDHDFY LFREQESGQL QVIYRRNHGG YGVIQAR
08Synechococcus_sp__RS9917_Bac  QLDVIDHDFY LFRDASTGDL QVIYRRNHGG YGVIQAR
34Gloeobacter_violaceus_PCC_74  HLALVDHDFF VFRNAESGEI NVLYARNHGG YGLIVPR
24Chamaesiphon_minutus_PCC_660  QLQLVDHDFY VYRSVETGEV NVVYRRNHGG FGVIQPR
16Halothece_sp__PCC_7418_Bacte  QLQLVDHDFY VFRNEETNEI NVIYQRNHGG YGVIQPR
29Synechococcus_sp__JA-2-3Ba_2  QLELVDHDFY VFRNIENGEI NVIYVRNHGG YGLIRPR
13Stanieria_cyanosphaera_PCC_7  QLQLVDHDFY MFLNKDTNEI NVIYVRNHGG YGVIQPR
03Cyanothece_sp__PCC_8802_Bact  QLELVDHDFY MFRNKDTDEI NVIYQRNHGG YGVIQPH
02Cyanobacterium_aponinum_PCC_  QLQLVDHDFY MFRNEETGEI NVIYQRNHGG YGVIQPR
10Gloeocapsa_sp__PCC_73106_Bac  QLQLVDHDFY MFRNSATDEI NVIYMRNHGG YGVIQPR
07Cyanothece_sp__PCC_7424_Bact  QLQLVDHDFF MFRNRDTNEI NVIYIRNHGG FGVIQPR
06Synechocystis_sp__PCC_6803_B  QLQLVDHDFY MFRNKDTDEI NVIYIRNHGG YGVIQPH
09Pleurocapsa_sp__PCC_7327_Bac  QLQLVDHDFY MFRNRDTNEI NVIYLRNHGG YGVIQPR
04Oscillatoriales_cyanobacteri  QLELVDHDFY VFRNAETGEI NVVYERNHGG YGVIQPR
36Rivularia_sp__PCC_7116_Bacte  QLRLVGHDFY MFHNSETDEI NVIYERNHGG YGVIQAR
22Synechocystis_sp__PCC_7509_B  QLQNVDHDFY MFCNAESGEI NVIYERNHGG YGVIQPR
19Cyanothece_sp__PCC_7425_Bact  QLELVDHDFY MFRNAETGEI NVIYERNHGG YGVIQPR
17Microcoleus_sp__PCC_7113_Bac  QLQLVDHDFY MFRNAKTGEI NVIYERNHGG YGVIQPR
21Oscillatoria_acuminata_PCC_6  QLQLVDHDFY MFRNADTGEI NVIYERNHGG YGVLQPR
30Oscillatoria_sp__PCC_6506_Ba  QLQVIDHDFY MFRNAETGEI NVIYERHHGG YGVIQPR
28Calothrix_sp__PCC_6303_Bacte  HLQLVGHDFY MFNNSETGEI NVIYERNHGG YGVIQPR
23Chroococcidiopsis_thermalis_  QLQLVDHDFY MFRNAETGEI NVIYERNHGG YGVIQPR
11Gloeocapsa_sp__PCC_7428_Bact  QLQLVDHDFY MFRNAETGEI NVIYERNHGG YGVIQPR
12Fischerella_sp__JSC-11_Bacte  QLQLVGHDFY MFRNSETGEI NVIYERNHGG YGVIQPR
32Nostoc_punctiforme_PCC_73102  QLQLVGHDFY MFRNSETGEI NVIYERNHGG YGVIHPR
25Calothrix_sp__PCC_7507_Bacte  QLQLVGHDFY MFHNAETGEI NVIYERNHGG YGVIQPR
26Anabaena_variabilis_ATCC_294  QLQLVGHDFY MFHNAETGEI NVIYERNHGG YGVIQPR
14Nostoc_sp__PCC_7107_Bacteria  QLQLVGHDFY MFRNAETGEI NVIYERNHGG YGVIQPR
```

```
18Nostoc_sp__PCC_7524_Bacteria  QLQLVGHDFY MFRNAETGEI NVIYERNHGG YGVIQPR
51Ectocarpus_siliculosus_Local  CLEYIGHDFY LFRNEETNEV NVVYKRKSGG VGLIEPE
35Phaeodactylum_tricornutum_CC  ALDYIDHDFY VFRNAETNEI SVVYKRHVGG IGLVEP-
15Thalassiosira_pseudonana_CCM  ALDYIDHDFY VFRDEETNEV SVVYKRNAGG VGLIQPE
```

Alignment: PMP

```
007Lyngbya_sp__PCC_8106_Bacter  SWFWLKLALD IRSSVIRSIW RRVLASMIFA GLINFINQPI LSSLIPSIVL
008Xenococcus_sp__PCC_7305_Bac  QKFWLGIIFH LQESVVPVIW HRVLGIMVFA IIITLASYNF TYNLIPSIVL
035Cyanobacterium_aponinum_PCC  KRKWFREAFR VKYSVFPDVW EKALFFAIFS FVICLLQQPI LSSLIPTIVL
006Leptolyngbya_sp__PCC_7375_B  DRRWFKTLFC LRGSVIRAVM PRVGICVGFA LIVTLLSWPI LGGVVPSIVL
069Trichodesmium_erythraeum_IM  EKRWLTVIFQ MQYSVIPSII FRVIFCGLFG FIISILTLPI KNSIVPSLVL
083Leptolyngbya_sp__PCC_6406_B  ETHWFLVLFR WQGSVLPAVL PRAFCCALFG ALVADFSLPV LGSVVPSIVL
048Gloeocapsa_sp__PCC_73106_Ba  NKRWFKLLLT LKGSVIPAIL PRVIFCGLFA WVISAISLDI LSSLVPSIVL
009Lyngbya_sp__PCC_8106_Bacter  DNKWFQLAFR VSGSVIPAIL PRVIFCSLFG VGLSILPTRI LDNLVPDLVL
196Microcoleus_sp__PCC_7113_Ba  KKRWFRVALR LQGSVIFAIL PRVILCSTFG FLITGFSWPA LSGVVPSIVL
107Trichodesmium_erythraeum_IM  RNKWLKLLLT LKGSIVPQVW QRVAVAMIFS AIITILHQPI LVSLIPSVVL
037Crinalium_epipsammum_PCC_93  ERNWFQLAFN FKSSVILNIK TQVFLSMVMA FLITLLSQPI LAGLIPGIIL
063Microcoleus_vaginatus_FGP-2  DRNWFKLALD LKSSVIRDIT TQVLLSMVMA SIITIVSQPI LAGLIPGIIL
253Euglena_gracilis_Eukaryota_  RDGIFRAALG ILRSIMGRLS FEVSMIAFLA TLICVISLPL SIFSLTAPSL
039Physcomitrella_patens_subsp  KSSHVRHVCT ILSSLIRAIG PPVFLCTLVS VFVAVIKVAT LPFTLTSPVL
045Physcomitrella_patens_subsp  RSSHYRHFAS SFSSAILSLI PPVGTMTAIS VFVALYHASS LSYQLTAPAL
074Selaginella_moellendorffii_  RSSHARHMAS TFSSVIISLI PPVFTVTGIS VLVTLYHAPS LPYELTAPAL
241Brachypodium_distachyon_Euk  RSSHLRHLLS SLSSVILSLA PPVSAFTAVA AAVATYTASS LPYQLTAPAL
129Oryza_sativa_Japonica_Group  RSSHLRHLLS SLTSVILSLA PPVSAFTAFA AAIATYTASS LPYQLTAPAL
205Zea_mays_Eukaryota__Viridip  RSSHLRHLLT SLSSAILSLA PPVSAFTAFA AAIATYTASS LPYELTAPAL
225Sorghum_bicolor_Eukaryota__  RTSHLRHLLT SLSSAILSLA PPVSAFTAFA AAIATYTASS LPYQLTAPAL
181Glycine_max_Eukaryota__Viri  RSSHLRHVFS SLSSVILSLV PPVLFFTAFS AAIAAYRTSS LPYQLTAPAL
089Arabidopsis_thaliana_Eukary  RSSHVRHLLS SFSSVILSLI PPVFFFTSVA VVIASYRSSS LPYQLTAPAL
102Arabidopsis_lyrata_subsp__l  RSSHVRHLLS SFSSVILSLI PPVFFFTSVA VVIASYRSSS LPYQLTAPAL
084Vitis_vinifera_Eukaryota__V  RSSHVRHLLS SFSSVILSLI PPVIAFTSVA VIVASYRASS LPYQLTAPAL
237Populus_trichocarpa_Eukaryo  RSSHVRHLLS SLSSVVLSLV PPVIAFTSVA VVVASYRTSS LPYQLTAPAL
183Ricinus_communis_Eukaryota_  RSSHLRHLLS SLQSVILSLI PPVIAFTSVA VIIASYRASS LPYQLTAPAL
018Volvox_carteri_f__nagariens  RSQLMKRLFT IPQSVIQNAL PSILWVGIVS SALAAYNASC SAFSNTSVAL
033Chlamydomonas_reinhardtii_E  RSSFAERLFQ LSQSILQNAL PAISWVTLVA TLVASYNASC TAFSNTSVAL
239Thalassiosira_pseudonana_CC  RSSFAKNLFS MVNSVYKSLA KEVFATTAVA SAIVAWVLPL TPFTLLSPSL
042Phaeodactylum_tricornutum_C  RNSVYENLQG IFFSIVRQLK SEVSLVALMA TLVVLWMVPA LPFTLSSPAL
113Micromonas_pusilla_CCMP1545  RSSLFGNLSG TLTSVVRSLL TEVAAVATIA TFTCAWQLPA LPFTLASPAL
119Aureococcus_anophagefferens  SL-WLHCLST WPRSVLERIK NPLAAIVGWS AVVAAWPLGT GLHTLVGGAL
124Ectocarpus_siliculosus_Loca  KFRWWHNLRT LPTSILHRIK HPLLVQTFWA TAVSLV---I KPHTLLGSAL
001QUERY_Bigel_pmp_86489_N9     ---WLYILRA MPRSTLAGI- KRVITVVLWS LALAIF---F ASPALVTPAL
154Emiliania_huxleyi_Localdb__  STRWLSSLKT LRHSVLSGIL QPLLLTTTAS LGVALFKALF QMHSLLGGAL
265Porphyridium_cruentum_Eukar  ---------- ---------- ---------- ---------- ----------
257Calliarthron_tuberculosum_E  ---------- ---------- ---------- ---------- ----------
218Thalassiosira_pseudonana_CC  ---------- ---------- ---------- ---------- ----------
```

```
003Phaeodactylum_tricornutum_C  RILWFHSAIT LPNSILRDIR NPVIAITSWA SFLSILYFPT APHSLMMSAL
005Thalassiosira_pseudonana_CC  HMCWKHNMKN LPSSILRDVG NPVGHVFVWA TFWSVVTVPT TAHSMMVSAM
187Pyropia_yezoensis_Localdb__  RFLWSRNLVS LPSSVLRRIS SHVAFNVVTA TAVVVAHLSP LPHVLLGTAL
256Lotharella_amoeboformis_Euk  ---------- ---------- ---------- ---------- ----------

                                51
007Lyngbya_sp__PCC_8106_Bacter  GLLLVFRTNT AYERYWEGRK LWGVMIHNGR VLARNIYFFK TNDRESIQLV
008Xenococcus_sp__PCC_7305_Bac  GLLLVFRTNT AYARFWEGCR AWGTLKISSR ILARNISTMD DPLQRFIKLV
035Cyanobacterium_aponinum_PCC  GLLLVFRTNS ANERFWEGRK LWGGIVNHLR NLTWQIWVNE TPDQEALNLL
006Leptolyngbya_sp__PCC_7375_B  GLLLVFRTNA SYERFWEGRK LWGNIVNTTR NLARQIWVSS ---RQALKLL
069Trichodesmium_erythraeum_IM  GLLLVFRTNT AYERFWQGRQ LWGNIVNIVR NFARQIWVTN NQDREIIRLL
083Leptolyngbya_sp__PCC_6406_B  GLLLVFRTNT AYERFWEGRK IWGHLINLSR NLARHIWVSR VEDRHMLRLL
048Gloeocapsa_sp__PCC_73106_Ba  GLLLVFRTNT AYDRFWEGRK LWGTLVNTVR NLARGIWVCT KPDRIALRLL
009Lyngbya_sp__PCC_8106_Bacter  GLLLVFRTNT AYDRFWEGRK LWGTLVNTVR NFARQIWVTN DPDTQTLKLL
196Microcoleus_sp__PCC_7113_Ba  GLLLVFRTNT AYERFWEGRK LWGTLVNTVR NLARQIWAAN KPDRTTLRLL
107Trichodesmium_erythraeum_IM  GLLLVFRTNT SYDRFWEGRK ATGGIIISCR SLSRQIWVNK TPDTEVIRLL
037Crinalium_epipsammum_PCC_93  GLLLVFRTNT AYERFWEGWQ IAGMTIFTGR NLSRQMWMTR TLDSQYLRLV
063Microcoleus_vaginatus_FGP-2  GLLLVFRTNT AYERFWEGWQ IAGMTIFTGR NISRPMWLTN TLQSQFVRLI
253Euglena_gracilis_Eukaryota_  GLLLVFRTNA SYGRWDETRR LFGQACNRCR ELARKAMTKE APRLRFLRYL
039Physcomitrella_patens_subsp  ALLLVFRTNT SYQRFDEARK AWGSNVNRAR DLARQALTWP GDKKLLLRYT
045Physcomitrella_patens_subsp  ALLLVFRTEA SYSRYDEARK TWTEVISSSK DMARQALAWP ADRKKLLDYI
074Selaginella_moellendorffii_  ALLLVFRTDT SYSRYDEARK TWTEVISSTK NLARLTEAWD D----LMRYI
241Brachypodium_distachyon_Euk  ALLLVFRTEA SYARFDEGRK AWIRVLAGAA ELVGMVMHPT DEAGRLVNYI
129Oryza_sativa_Japonica_Group  ALLLVFRTEA SYARFDEGRK AWMRVIAAAA DLAGMAMRHH NPATRLLNYI
205Zea_mays_Eukaryota__Viridip  ALLLVFRTEA SYARFDEGRK AWMRVLASAA DLAGMLMRHD DELRRLVNYV
225Sorghum_bicolor_Eukaryota__  ALLLVFRTEA SYARFDEGRK AWMRVLAGAA DLAGMLMHHD DDLRRLINYI
181Glycine_max_Eukaryota__Viri  ALLLVFRTEA SYSRFVEGKK AWTNVIAGTH DFARQVAAIN NFIKHLLHYI
089Arabidopsis_thaliana_Eukary  ALLLVFRTEA SYSRYEEGRK AWVGIIAGTN DLARQVICSD ELIKDLLRYI
102Arabidopsis_lyrata_subsp__l  ALLLVFRTEA SYSRYEEGRK AWVGIIAGTN DLARQVICSD ELIKDLLRYI
084Vitis_vinifera_Eukaryota__V  ALLLVFRTEA SYSRFEEGRK AWTKIIAGTN DFARQVVAGD ALLKKLLQYI
237Populus_trichocarpa_Eukaryo  ALLLVFRTEA SYSRFEDGKT AWTKVISGTN DFARQVISGN SALKSLLRYI
183Ricinus_communis_Eukaryota_  ALLLVFRTEA SYSRYEEGRK AWTKVISGVN DFARQVIATD AVLKNLLQYV
018Volvox_carteri_f__nagariens  SLLLVFRTNS SYGRWDEARK MWGGLLNRSR DIMRQGATCD Q--VELARWV
033Chlamydomonas_reinhardtii_E  SLLLVFRTNS SYGRWDEARK MWGGLLNRSR DIMRQGATCD Q--VELARWT
239Thalassiosira_pseudonana_CC  GLLLVFRTNS SYGRWDEARK MWGLNINHTR DLNRMATAWD NQIDPVSLYT
042Phaeodactylum_tricornutum_C  GLLLVFKTNT SYARWYEARG TWSKLTSQSL NLVRMASTFA -DVTQLATAA
113Micromonas_pusilla_CCMP1545  GLLLVFRTNA SYARWVESRV AWGRVVSHCR NVMRQSSLWD REFVVLRGKA
119Aureococcus_anophagefferens  SLLLVFRTNT AYTRYWEGRE IFGRLATGTR DLVDFVGLYV LRFRRVGGGA
124Ectocarpus_siliculosus_Loca  GLLLVFRTNA AYQRFQEGRK LWEEVLNVSR DIARMSTLYG RRVQRIANLL
001QUERY_Bigel_pmp_86489_N9     GLLLVFRTNA AYNRFWEGCK LWEQLIAKSR TLIRYASMYG KEVRRISHLL
```

```
154Emiliania_huxleyi_Localdb__  SLLLVFRTNS AYNRFWEARR IWENLLNRCR ELARYAYCFQ PCVDRLAQLL
265Porphyridium_cruentum_Eukar  ---------- ---------- ---------- ---------- ----------
257Calliarthron_tuberculosum_E  ---------- ---------- ---------- ---------- ----------
218Thalassiosira_pseudonana_CC  ---------- ---------- ---------- ---------- ----------
003Phaeodactylum_tricornutum_C  GLLLVFRTNS AYQRFTEGRT IWEQIINSSR DLFRLMMLYD LVMKTVDDEA
005Thalassiosira_pseudonana_CC  SLLLVFRTNS AYQRFAEGRK IWEDIVDVAR DFSRMLKLYS LLMRRVNDPN
187Pyropia_yezoensis_Localdb__  GLLLSYRTSA AYDRFWEARK VLGGMVNETR NIMRMAVTGT MHLQGTDSED
256Lotharella_amoeboformis_Euk  ---------- ---------- ---------- ---------- ----------

                              101
007Lyngbya_sp__PCC_8106_Bacter  STLVWVIKVH VRNIETELKN LLTVANWLAD YLIRMYINYR FFIELNQSID
008Xenococcus_sp__PCC_7305_Bac  PVLMSSVKAH LRSIEQRGQL VLGIINWFSS YFYRNYISDK LFTELSGLLD
035Cyanobacterium_aponinum_PCC  TVFAIATKNH LRGISEEMRP LLSIAAYLGE YLQQEYLNEY QLTNMQQLIN
006Leptolyngbya_sp__PCC_7375_B  VAFAIATKQH LRPCAHELNH LVTIAFWLGD YLQVQQINSY QLQAMTSQLN
069Trichodesmium_erythraeum_IM  VAFAIASKLH LRKINLELQG YMPIAFLIGD YLQNQYVNPY QLTALFQLLD
083Leptolyngbya_sp__PCC_6406_B  VAFAIALKLH LRPPNAELAA MMTVAFWMAD YLQDCHINTY QLAAMMNLIN
048Gloeocapsa_sp__PCC_73106_Ba  VAFAIATKLH LREVNQELAD LMPIAFWLAD YLQKQSLSAY QLVAMQKLIN
009Lyngbya_sp__PCC_8106_Bacter  IAFAIAMKLH LRKINQEIED LLSIALWIGD YLQRQYLNTY QLTAMFKLLD
196Microcoleus_sp__PCC_7113_Ba  IAFAVATKLH LRRLNNELEP LMSIAFWIGD YLQKQHLNTY QLSAMLKLLD
107Trichodesmium_erythraeum_IM  AGFLIGTKLH LRSLDLKIRS IISIAKWIGD YFRKAYIDSI QLSTYNQILD
037Crinalium_epipsammum_PCC_93  TAFFMAMKQH LRRVDEQLKS VLSVTQWIGG YLTKQYIDSI QFAALNRLLD
063Microcoleus_vaginatus_FGP-2  PAFFMAMKQH LRGVDQSLKS VLSVTQWIGG YLTKQYIDSI QFAAINRLLD
253Euglena_gracilis_Eukaryota_  QAFPFVLKVY LRNLVEDLQG TLEVFQCLTE IIQRAKLDTA TAIAMEHSVS
039Physcomitrella_patens_subsp  KAYSFCLMHH LRCLRKELEA IVNVLQVISD IINECQITPW ERIAMDKNIT
045Physcomitrella_patens_subsp  LAFPVALKCH LLDIAEELRE ILELIQLMTL SLKSIKFEDG EGMQLDANIS
074Selaginella_moellendorffii_  VAFPLALKCH LIDMEADLRK VLDLLQMIFQ IIDGLSLGET QQVLLHENIS
241Brachypodium_distachyon_Euk  LAFPVALKCH ITDIRKDLQG LLAIIEFISQ SLQMLDFEEH RRSIMESKLS
129Oryza_sativa_Japonica_Group  LAFPLALKCH IIDIKRDLQG LLSIIEFISQ SLYMLDFDEN KRNIMESKLS
205Zea_mays_Eukaryota__Viridip  LAFPVALKCH IIDVKGDLEG LLGIIEFIAQ SLQMLDLDEQ KRSIMVSKLS
225Sorghum_bicolor_Eukaryota__  LAFPVALKCH IIDVKGDLQG LLAIIEFISQ SLQMLDLDEQ KRSIMESKLS
181Glycine_max_Eukaryota__Viri  IAFPIALKCH VLDVRRDLQH LLEIIEFISQ SIRLLKLEES RRNVLESKIT
089Arabidopsis_thaliana_Eukary  AAFPVALKCH VIDIARDLRN LIEVIEFISQ SIQLLKLDDA KRDLLESKML
102Arabidopsis_lyrata_subsp__l  AAFPVALKCH VIDIARDLRN LIEVIEFISQ SIQLLKLDDA KRDLLESKML
084Vitis_vinifera_Eukaryota__V  MAFPVALKCH VIDIRQDLQN LLEIIEFISQ SLQLLNLDDA KRHVLESKLS
237Populus_trichocarpa_Eukaryo  MAFPIALKCH VTNIGQDLQN LLEIIEFISQ CLQLLNLDES MRNLLESKIS
183Ricinus_communis_Eukaryota_  IAFPVALKCH VIDINRDLRN LLEIIEFISQ SLQLLNLEES KRHLLESKIS
018Volvox_carteri_f__nagariens  VAFARALRIH FQSIESELKN ILTAIHAISQ IIQSVPMSSI HQMQMSNNLT
033Chlamydomonas_reinhardtii_E  VAFSRALRIH FQTIESELQN ILTAIHAISQ IIQSVPMSSI HQQQMSNNLT
239Thalassiosira_pseudonana_CC  WAFVRSMKRH LSAFVEELYA RMAALYDLSV VIDKLPMHFM RKNEINKNLS
042Phaeodactylum_tricornutum_C  WLVCRSVMNE LWAYRKDVEQ AFAALAEASL ALDAIPIDEK RRVEMDKSLV
```

```
113Micromonas_pusilla_CCMP1545  WAFPRCLASH VSALARELRL RLGALADLSY CMNNLPIDEK RRVEMDKSII
119Aureococcus_anophagefferens  DGAPIPLETF LGVAAALGVP DLELCTLLAR EIKAVAFTTR ERLYVLKRVA
124Ectocarpus_siliculosus_Loca  ACHAVILQEH LQWEDLLRPE VRDVVNMISR EIKSVPWSSR ERLAILSMCN
001QUERY_Bigel_pmp_86489_N9     SAMGIVLKQH LTQETAYLER LLDISNLIAR EVHSIPGGGE ERIHMLKLVD
154Emiliania_huxleyi_Localdb__  TAFPVMLRRH LCAPLAPLPA LCDLCKRLAA EISGVSFTSR ERLMGLSLVN
265Porphyridium_cruentum_Eukar  ---------- ---------- ---------- ---------- ----------
257Calliarthron_tuberculosum_E  ---------- ---------- ---------- ---------- ----------
218Thalassiosira_pseudonana_CC  ---------- ---------- ---------- ---------- --------VN
003Phaeodactylum_tricornutum_C  YKREPENTIL LYDDAEAAAV NAEVCDRMAM ELRAVPFTNR ERLALISHVD
005Thalassiosira_pseudonana_CC  VVRDPENSLL LYTDPEIAAM YDEVCDRMAK ELAVVDFTNR ERMALIEYTA
187Pyropia_yezoensis_Localdb__  GVAEYLTPEQ LVRPMAVAML ATA--ALVNA RAGVGANLLA ERQEMEKLVT
256Lotharella_amoeboformis_Euk  ---------- ---------- ---------- ---------- ----------

                               151
007Lyngbya_sp__PCC_8106_Bacter  RILEAMIACD RIASSPLPKA YSIHLKHLLL IYCLSVPFTF VEELNWLTVP
008Xenococcus_sp__PCC_7305_Bac  AIVGALSSCE RILNTPMPKA YSIHLKHLLL IYCAGLPIQF VAQLDWWVVL
035Cyanobacterium_aponinum_PCC  QLMDMVGGCE RILKTPIPNS YSIHLKQLLL IYCLVLPFQL VDQIEWWTIP
006Leptolyngbya_sp__PCC_7375_B  SLVNSLGGCE RILNTPLPLA YSIHLKQLLV MYCLALPFQV VSELNGWTIP
069Trichodesmium_erythraeum_IM  KMVEALTSCE GILKTPIPLA YSIHLRQLLL IYCLLLPFQI VNQFYFWTGL
083Leptolyngbya_sp__PCC_6406_B  GMVDAIGGCE RILKTPIPLA YSVHLKQLLM LYCLALPFQV VDSMHGWTAL
048Gloeocapsa_sp__PCC_73106_Ba  ILVDCLGGCE RIIRTPLPMA YSIHLRQLLL IYCLSLPFQV VEELVWFTGP
009Lyngbya_sp__PCC_8106_Bacter  TMVDVLGACE RIIKTPIPLA YNIHLKQILL IYCLVLPLQL TDDLLWYTGI
196Microcoleus_sp__PCC_7113_Ba  TMVDVLGSCE RILKTPIPLA YSIHLKQLLL LYCLSLPFPM VNDLHWWTAV
107Trichodesmium_erythraeum_IM  SMVKDETACE RILNTPIPLA YAIHLKHLLF LYCFALPFQM VAKLSWITIL
037Crinalium_epipsammum_PCC_93  QLVECLSRCE RILAAPMPKA YSIHLRHLLI LYCLALPFQM VKDLQWWTIP
063Microcoleus_vaginatus_FGP-2  QLVECLSRCE RILAAPMPKA YSIHLRHLLL LYCLALPFQM VKDLQWCTIP
253Euglena_gracilis_Eukaryota_  ALADMYTALD RLLRTPIPLS YTSLTTRFLV LWLVLLPLAL SAD-------
039Physcomitrella_patens_subsp  QFHDNVGACE RIFKTPIPVA YTRLTSRVLT LWHLVLPFAL WETCGWHTIT
045Physcomitrella_patens_subsp  QFNESISVCE RLIRTPIPLA YTRLTSRILV LWHLSLPVVL WDDCHWVVVP
074Selaginella_moellendorffii_  TYNRSVSVCE RLIRTPIPLS YTRLTSRFLI LWHLGLPIAL WDTCNWLVIP
241Brachypodium_distachyon_Euk  GFLEGICVCE QIIGIPVPLS YTRLTSRFLV LWHLTLPVIL WSECKWIVVP
129Oryza_sativa_Japonica_Group  CFLEGISVCE QLIGIPIPLS YTRLTSRFLV LWHLTLPVIL WDECKWIVVP
205Zea_mays_Eukaryota__Viridip  CFLEGIGVCE QLMGIPIPLA YTRLTSRFLV LWHLTLPIIL WEECKWIVVP
225Sorghum_bicolor_Eukaryota__  CFLEGIGVCE QLMGIPIPLS YTRLTSRFLV LWHLTLPVIL WEECKWIVVP
181Glycine_max_Eukaryota__Viri  CFHEGIGICD QLMGIPIPLA YTRLTSRFLV LWHLTLPIIL WDDCHWIVVP
089Arabidopsis_thaliana_Eukary  HLHEGIGVCE QLMGIPIPLS YTRLTSRFLV FWHLTLPIIL WDECHWIVVP
102Arabidopsis_lyrata_subsp__l  HLHEGIGVCE QLMGIPIPLS YTRLTSRFLV FWHLTLPIIL WDECHWIVVP
084Vitis_vinifera_Eukaryota__V  CFHEGIGVCE QLMGIPIPLS YTRLTSRFLV LWHLTLPIIL WEDCNWIVVP
237Populus_trichocarpa_Eukaryo  CFHEGIGVCE QLMGIPIPLS YTRLTSRFLV LWHLTLPIIL WDDCHWIVVP
183Ricinus_communis_Eukaryota_  CLHEGIGVCE QLMGIPIPLS YTRLTSRFLV LWHLTLPIIL WDDCHWIVVP
```

```
018Volvox_carteri_f__nagariens  FFHDVLGGCE RLLRAPIPVS YTRHTARFLF MWLTLLPFAL YGQCGLGVVP
033Chlamydomonas_reinhardtii_E  FFHDVLGGCE RLLRAPIPVS YTRHTARFLF AWLTLLPFAL YPTTGWGVVP
239Thalassiosira_pseudonana_CC  IFEDTLGGCE RLLSSPVPLF YTRHTARFLS TWLLLLPLAM YQPFSWAMIP
042Phaeodactylum_tricornutum_C  LMGDGISVCQ RVFASPVPLV YTRHTSRFLS LWMLLLPGAL YETFAWGLIP
113Micromonas_pusilla_CCMP1545  LLGDALETCE RIFTSPVPLV YTRHTARFLS FWLLLLPLAL WEPFGWAMIP
119Aureococcus_anophagefferens  ELRATIAQAE RIVQTPVPLH YARHTSRFLS VWCFTLPLCL APMVAYVAPP
124Ectocarpus_siliculosus_Loca  KLGSTIGGCE RLVQTPVPLH YVRHTSRFLT IWCFLLPLVI VGEMGMATAP
001QUERY_Bigel_pmp_86489_N9     EITGVISACE RIVQTPVPLN YARHTSRFLT IWCLTLPIAL VANLGFSVVP
154Emiliania_huxleyi_Localdb__  QLGSYVGACE RLLQTPVPLN YARHTSRFLT LWCLTLPVSL VGSMGLLVVP
265Porphyridium_cruentum_Eukar  ------GMCE RIVKTPVPLS YSRHTSRFLS LWCLTLPVIF VKADDWLVLP
257Calliarthron_tuberculosum_E  ------GACE RIVKTPVPLS WSRHTTRLLS IWALTLPLVL VPLEGVMSVP
218Thalassiosira_pseudonana_CC  HLSDALSGCE KIVQTPVPLS YSRHTSRFLT LWCGFLPFAI VSQLGWLSIP
003Phaeodactylum_tricornutum_C  KLSRCIGGSE RIHQTVVPLN YARHTLRALT VWLFSLPFVV VKDLKLLTGP
005Thalassiosira_pseudonana_CC  KLSRSIGACE RIHQTLVPLN YARHALRSLT VWLWTLPFAL VKDLGLLTGP
187Pyropia_yezoensis_Localdb__  LLVDYLGMCE RIVLTPIPLN YSRHTSRFLS LWTASLTVVL VELQGPFLIL
256Lotharella_amoeboformis_Euk  ------GACE RIQGCPVPLS YSRHLSRYLT IWSMTLPYVL TPILGWYSIP

                                201
007Lyngbya_sp__PCC_8106_Bacter  AVGVITFSLL GIEEIGLEIE NPFNDLPLEK FCYILQNDS
008Xenococcus_sp__PCC_7305_Bac  VTGVTSFALL GIEAIGLEIE DPFNDLPLDL YCQKIYGEF
035Cyanobacterium_aponinum_PCC  FVSIVSYIVY GIEAIALEIE NPFNDLPLDQ ICQAINQN-
006Leptolyngbya_sp__PCC_7375_B  VVALVAFAVF GVEEIGLEIE NPFNDLPLNA ICQTMEHNI
069Trichodesmium_erythraeum_IM  VVAIISFTVL GIEEIALEIE NPFNDLPLDS ICTTMHHNV
083Leptolyngbya_sp__PCC_6406_B  IVGLISFAVF GIEEIGIEIE NPFNDLPLDL ICLTMKRNC
048Gloeocapsa_sp__PCC_73106_Ba  FVALISFTVF GIEEIGIEIE NPFNDLPLDQ ICHTMQVNV
009Lyngbya_sp__PCC_8106_Bacter  VVGIISFTLF GIEEIGREIE NPFNDLELDA ICQTMQRNV
196Microcoleus_sp__PCC_7113_Ba  VVGLISFAVF GIEEIGIEIE NPFNDLPLDT ICATMERNV
107Trichodesmium_erythraeum_IM  VVGIISFALL GIEAIGLEIE NPFNDLPLDN MCDKLLLEK
037Crinalium_epipsammum_PCC_93  VVGGVAFALF GIEAIGLEIE NPFNDIPLDR LCQKLDSDQ
063Microcoleus_vaginatus_FGP-2  VVGVVAFALF GIEAIGLEIE NPFNDIPLDR LCQKLHNDE
253Euglena_gracilis_Eukaryota_  ---------- ---------- ---------- ---------
039Physcomitrella_patens_subsp  VSFVSSAALF YIEEVGVMIE EPFSILALST ICTGIVAAW
045Physcomitrella_patens_subsp  ATFISAASLF CIEEVGVLIE EPFPILALDR MCAKARENS
074Selaginella_moellendorffii_  STFFSSAALF CIEEVGVLIE EPFPMLALDR MCEVALIN-
241Brachypodium_distachyon_Euk  ATFISAASLF CIEEVGVLIE EPFPMLALDE QCKQLHDSV
129Oryza_sativa_Japonica_Group  ATFISAASLF CIEEVGVLIE EPFPMLALDE LCKQLHDSA
205Zea_mays_Eukaryota__Viridip  ATFISAASLF CIEEVGVLIE EPFPMLALDA LCKQLHDGV
225Sorghum_bicolor_Eukaryota__  ATFISAASLF CIEEVGVLIE EPFPMLALDA LCKQLHDGV
181Glycine_max_Eukaryota__Viri  ATFISAASLF CIEEVGVLIE EPFATLALDD LCQKAQTDV
089Arabidopsis_thaliana_Eukary  ATFISAASLF CIEEVGVLIE EPFPMLALDE LCDLVHSNI
```

```
102Arabidopsis_lyrata_subsp__l  ATFISAASLF CIEEVGVLIE EPFPMLALDE LCDLVHSNI
084Vitis_vinifera_Eukaryota__V  ATFISAASLF CIEEVGVLIE EPFPMLALDE LCKLVHNNI
237Populus_trichocarpa_Eukaryo  STFISAASLF CIEEVGVLIE EPFPMLALDE LCNVVQSNV
183Ricinus_communis_Eukaryota_  ATFISAASLF CIEEVGVLIE EPFPMLALDE LCNLVNSNI
018Volvox_carteri_f__nagariens  VCTGIAAVLC GIEEIGVQCE EPFGILPLEV ICNRI---M
033Chlamydomonas_reinhardtii_E  VCTGIAAVLC GIEEIGVQCE EPFGILPLDV ICNRI---M
239Thalassiosira_pseudonana_CC  ATALTSVFLF GIDELSTQLE EPFTILPMQG FCDKIWC-I
042Phaeodactylum_tricornutum_C  AVSVVALFLF GIEELAIQLE EPFSILPLDR IVEGVWCIF
113Micromonas_pusilla_CCMP1545  SATLVAIFFF GIEELAVQLE EPFSILPLKR LCDNVW--F
119Aureococcus_anophagefferens  VVGVVSWALL GLREIGLLIE NPALDLPYGS PALNVPTDV
124Ectocarpus_siliculosus_Loca  VVFLATWALF GIQEIGLLIE DPFSALSLDI MCNSVYTDQ
001QUERY_Bigel_pmp_86489_N9     VTALVVWAMF GIQEIGLMIE EPFKSLSLHV FCNTIHNDQ
154Emiliania_huxleyi_Localdb__  VTAFVTWCLF GIQEIGLFIE HCADNGD--I FMDQLSD--
265Porphyridium_cruentum_Eukar  IMFFVSFSLF SIEEIGHLIE DPFIDLPLEA MASTINGDG
257Calliarthron_tuberculosum_E  TVAVISWGIF SIEEIAHIME DPFYSLPLEG FCKTIESD-
218Thalassiosira_pseudonana_CC  VMLTVSWLLY GLEEIGHLIE QPFTDRPTYL ISDDVHDDA
003Phaeodactylum_tricornutum_C  VLFLVSWMLF GVFEIGSAIE DPF--LRLSI LCDTIRRDA
005Thalassiosira_pseudonana_CC  VVAVVSWVLF GVYEIGTRIE DPF--LRLSI YCNSVRRDQ
187Pyropia_yezoensis_Localdb__  DMLFIAWALF GLEEVGHMIE DPLSSLPFIN VCNTIRRDV
256Lotharella_amoeboformis_Euk  VIGLICWSLF SIEEIGHMIE EPFDQVQTKR VADVIKRDV
```

Alignment: PDP

```
54Cyanidioschyzon_merolae_Euka  TPPCVVKVFG VGGGGCNAIS RMLDGEFRGV RFAIANTDHQ ALIENATVVP
40Ostreococcus_tauri_Eukaryota  --NAKIKVLG CGGGGSNAVN RMISGGLQGV EFWTVNTDSQ ALVNLANKLQ
23Micromonas_sp__RCC299_Eukary  VSEARIKVIG CGGGGGNAVN RMINSGLQGV EFWSLNTDAQ ALVQQANRIQ
34Volvox_carteri_f__nagariensi  GGDARIKVIG VGGGGGNALN RMISSGLQGV EFWAINTDAQ ALAAQANKVQ
39Chlamydomonas_reinhardtii_Eu  GGDARIKVIG VGGGGGNALN RMINSGLQGV EFWAINTDAQ ALAAQANKVQ
36Brachypodium_distachyon_Euka  VETARIKVVG VGGGGNNAVN RMIGSGLQGI EFYAINTDSQ ALVNQAHPLQ
29Physcomitrella_patens_subsp_  LSGAKIKVIG VGGGGNNAVN RMIGSGIQGV DFWAINTDVQ ALQKQAHRVQ
30Selaginella_moellendorffii_E  MDSARIKVVG IGGGGNNAVN RMIGSGLQGV DFWAINTDAQ ALVQSANRLQ
47Prochlorococcus_marinus_subs  SQNAKIEVIG VGGGGSNAVN RMIDSDLEGV SFRVLNTDAQ ALLQSSRRVQ
51Synechococcus_sp__CC9311_Bac  SQSARIEVIG VGGGGSNAVN RMILSDLEGV AYRVLNTDAQ ALIQSANRVQ
21Synechococcus_sp__JA-3-3Ab_B  SNAAKIKVVG VGGGGGNAVS RMAASNLKGV EFWSINTDAQ ALAQSTNRLQ
38Chamaesiphon_minutus_PCC_660  SNTARIKVIG VGGGGSNAVN RMIASDIEGI EFWTMNTDAQ ALSHSDRRIQ
10Cyanothece_sp__PCC_8802_Bact  NNVAKIKVIG VGGGGCNAVN RMIESSLTGI EFWAINTDAQ ALSQSAQRLQ
20Stanieria_cyanosphaera_PCC_7  SNIAKIKVMG VGGGGCNAVN RMIDSGVSGI EFWAINTDAQ ALSHAGQRLQ
32Gloeobacter_violaceus_PCC_74  SSVALIKVVG VGGGGGNAVN RMIASNVVGV EFWAINTDAQ SLTQSSQRLQ
25Gloeocapsa_sp__PCC_73106_Bac  SNVAQIKVIG VGGGGCNAVN RMIERDVSGV EFWAINTDAQ ALAHAAYRLQ
24Chroococcidiopsis_thermalis_  SRVANIKVIG VGGGGGNAVN RMIESGVTGV EFWSINTDAQ ALTHSARKLQ
09Cyanobacterium_stanieri_PCC_  SNVAQIKVIG VGGGGCNAVN RMIQSSVSGV EFWQINTDAQ ALTESMYCLQ
28Synechocystis_sp__PCC_6803_B  SNIAKIKVIG VGGGGCNAVN RMIASGVTGI DFWAINTDSQ ALTNTNDCIQ
15Pleurocapsa_sp__PCC_7327_Bac  SNVAKIKVIG VGGGGCNAVN RMIESGVSGI EFWAINTDAQ ALSQSEQRLQ
11Cyanothece_sp__PCC_7822_Bact  SNVAQIKVIG VGGGGCNAVN RMIASGIVGV EFWSINTDAQ ALAHSAQRLQ
13Microcystis_aeruginosa_PCC_9  SNVAKIKVIG VGGGGCNAVN RMIASGVTGI EFWAINTDAQ ALAHSSQRLQ
41Leptolyngbya_sp__PCC_7376_Ba  SSVAQIKVIG VGGGGGNAVN RMLESGVSGI DFWSINTDAQ ALTNALNRLQ
22Synechococcus_sp__PCC_7502_B  SSAAKIKVIG VGGGGGNAVN RMIASEISGV EFWSLNTDAQ ALLQSSKRFQ
44Raphidiopsis_brookii_D9_Bact  GRVANIKVIG VGGGGGNAVN RMIESDVTGV EFWSINTDAQ ALTWANSRLQ
18Rivularia_sp__PCC_7116_Bacte  GRVANIKVIG VGGGGGNAVN RMIASDLSGV EFWSINTDAQ ALTMAACRLQ
37Fischerella_sp__JSC-11_Bacte  GRVANIKVIG VGGGGSNAVN RMIASDVSGV EFWSINTDAQ ALTLAASRLQ
43Calothrix_sp__PCC_6303_Bacte  GRVANIKVIG VGGGGSNAVN RMIASDVNGV EFWSINTDAQ ALTLADSRLQ
31Oscillatoriales_cyanobacteri  GSVAKIKVIG VGGGGGNAVN RMIASDVTGV EFWSINTDAQ ALEGSDKRLQ
45Geitlerinema_sp__PCC_7407_Ba  SSIARIKVIG VGGGGCNAVN RMIASEVSGV EFWSINTDAQ ALTNPRQHLQ
17Synechocystis_sp__PCC_7509_B  GRVANIKVIG VGGGGGNAVN RMIASNVSGI EFWSINTDAQ ALVQSAKRLQ
26Oscillatoria_acuminata_PCC_6  SSIAKIKVIG VGGGGCNAVN RMIASEVSGV EFWAVNTDAQ ALVQSTKRLQ
16Gloeocapsa_sp__PCC_7428_Bact  GRVANIKVIG VGGGGGNAVN RMIASEVSGI EFWSINTDAQ ALTNTSRRLQ
19Coleofasciculus_chthonoplast  GSVARIKVVG VGGGGGNAVN RMIASEVAGI EFWSINTDSQ ALSQNSKRLQ
33Chlamydomonas_reinhardtii_Eu  SDQAIIKVLG VGGGGSNAVN NMVNSDVQGV EFWIANTDAQ ALATPVCKVQ
50Ostreococcus_lucimarinus_CCE  TNAATIKVVG VGGGGSNAVN RMVDADINGV EFWIVNTDAQ ALETVANHLQ
27Ricinus_communis_Eukaryota__  SDEAKIKVIG VGGGGSNAVN RMIESSMTGV EFWVVNTDIQ AMKTLVNRLQ
35Selaginella_moellendorffii_E  FNEAKIKVIG VGGGGSNAVN RMVQSEMKGV EFWIVNTDAQ AMAMPVNRLQ
```

```
42Physcomitrella_patens_subsp_  YNEAKIKVIG VGGGGSNAVN RMLESEMQGV EFWIVNTDAQ AMALPVNRLQ
06Thalassiosira_pseudonana_CCM  TNPCIIKVLG VGGGGGNAVN RMIQTRIDGV SFWAVNTDAQ ALAKLANVLN
12Phaeodactylum_tricornutum_CC  A-PCVIKVIG VGGGGGNAVN RMIQTRIEGV SFWALNTDAQ ALSKLANVLN
100Ectocarpus_siliculosus_Loca  VSPVSIKVVG VGGGGGNAVD GMITRKLSGV EFVAMNTDTQ ALTKVK--IA
07Thalassiosira_pseudonana_CCM  LSPCVIKVLG VGGGGSNAVD RMLDTRISGV EFWSINTDAQ ALGRKAQILN
08Phaeodactylum_tricornutum_CC  LSPCVIRVLG VGGGGCNAVD RMLETAVGGV EYWAINTDAQ ALGRKANVLN
05Thalassiosira_pseudonana_CCM  LSPCVIKVVG VGGGGCNAVD RMLDTRVSGV DFWAINTDAQ ALGRKARVLN
14Phaeodactylum_tricornutum_CC  LSPCVIKVIG VGGGGCNAVD RMLDTAVGGV DFWALNTDAQ ALGRKAKVLN
108Emiliania_huxleyi_Localdb__  MAPCVIKVVG VGGGGGNAVN RMIQAQTGAV EYWALNTDIQ ALDALSNRLG
52Lotharella_amoeboformis_Euka  ---------- ---------- ---------- ---------- ----------
53Lotharella_amoeboformis_Euka  ---------- ---------- ---------- ---------- ----------
01QUERY_Bigel_39262             -----VQVVG IGGGGGNTVN RMIDAYDSGV EFWSINTDVQ ALSRLANRIQ
55Cyanidioschyzon_merolae_Euka  APPCLIKVIG VGGGGGNAVN RMATGISG-V EFWAINTDVQ ALKRAAHTLS
096Aureococcus_anophagefferens  TAPCCIKVVG VGGGGGNAVN RMVTDAGSFV DFWAMNTDAQ ALSR-LNTMN
04Cryptomonas_paramecium_Eukar  SSPCIIKVIG VGGGGGNAVN RMIGSVEG-V EFWSINTDSQ ALSRLANTCN
02Hemiselmis_andersenii_Eukary  ASPCLIKVIG VGGGGGNAVN RMVGCVEG-V EFWSINTDAQ ALSRLANTCN
03Guillardia_theta_Eukaryota__  SSPCVIKVIG VGGGGGNAVN RMVGGVEG-V EFWSINTDAQ ALSRLANTCN

                                51
54Cyanidioschyzon_merolae_Euka  LGESICRGLG AGGNPEVGCA AAEESHDRIA QAIGGTDLLF ITAGMGGGTG
40Ostreococcus_tauri_Eukaryota  IGEQVTRGLG AGGNPELGEI AANESRDALE QAVSGSDLVF ITAGMGGGTG
23Micromonas_sp__RCC299_Eukary  IGKQVTRGLG TGGNPELGKK AAEESATEIQ QAVRGADLVF VTAGMGGGTG
34Volvox_carteri_f__nagariensi  IGTELTRGLG CGGNPELGRQ AALESEDALR RMVQGADLVF ITAGMGGGTG
39Chlamydomonas_reinhardtii_Eu  IGSELTRGLG CGGNPELGRR AAMESEEALR RMVQGADLVF ITAGMGGGTG
36Brachypodium_distachyon_Euka  IGEQLTRGLG TGGNPNLGEQ AAEESKEVIA NALRDSDLVF ITAGMGGGTG
29Physcomitrella_patens_subsp_  IGEALTRGLG TGGKPFLGEQ AAEESIDIIA EAVVDADLVF ITAGMGGGTG
30Selaginella_moellendorffii_E  IGEELTRGLG TGGKPSLGEE AAEESKDDLK VAVADSDLVF ITAGMGGGTG
47Prochlorococcus_marinus_subs  LGQNLTRGLG AGGNPSIGQK AAEESKDELQ QTLEGSDLVF IAAGMGGGTG
51Synechococcus_sp__CC9311_Bac  LGQTLTRGLG AGGNPSIGQK AAEESRADLQ QALQGADLVF IAAGMGGGTG
21Synechococcus_sp__JA-3-3Ab_B  IGQKLTRGLG AGGNPAIGQK AAEESSEEIA AALKGADLVF IAAGMGGGTG
38Chamaesiphon_minutus_PCC_660  LGQKLTRGLG AGGNPAIGQK AAEESREEIA HALEGADLVF ITAGMGGGTG
10Cyanothece_sp__PCC_8802_Bact  IGQKLTRGLG AGGNPSIGTQ AAEESRDEIA QALENTDLVF ITAGMGGGTG
20Stanieria_cyanosphaera_PCC_7  IGQKITRGLG AGGNPAIGQK AAEESRDEIA HALENTDLVF ITAGMGGGTG
32Gloeobacter_violaceus_PCC_74  IGQKLTRGLG AGGNPSIGQK AAEESREEIM TALEGADLVF ITAGMGGGTG
25Gloeocapsa_sp__PCC_73106_Bac  VGKKITRGLG AGGNPAIGQK AAEESREEIA GALENTDMVF ITAGMGGGTG
24Chroococcidiopsis_thermalis_  IGQKLTRGLG AGGNPAMGEK AAEESRDEIA NAIGEADLVF ITAGMGGGTG
09Cyanobacterium_stanieri_PCC_  IGQKLTRGLG AGGNPSIGQK AAEESREEIA KALENTDLVF ITAGMGGGTG
28Synechocystis_sp__PCC_6803_B  IGQKLTRGLG AGGNPAIGQK AAEESRDEIA RSLEGTDLVF ITAGMGGGTG
15Pleurocapsa_sp__PCC_7327_Bac  IGQKLTRGLG AGGNPAIGQK AAEESREEIA HALENTDLVF ITAGMGGGTG
11Cyanothece_sp__PCC_7822_Bact  IGQKITRGLG AGGNPAIGQK AAEESRDEIA HALENTDLVF ITAGMGGGTG
```

```
13Microcystis_aeruginosa_PCC_9  IGTKLTRGLG AGGNPAIGQK AAEESRDEIA QALEGTDLVF ITAGMGGGTG
41Leptolyngbya_sp__PCC_7376_Ba  IGQKITRGLG AGGNPAIGQK AAEESRDEIA QALEGADLVF ITAGMGGGTG
22Synechococcus_sp__PCC_7502_B  IGQKITKGLG AGGNPAIGQK AAEESRAEIA HALEGADLVF ITAGMGGGTG
44Raphidiopsis_brookii_D9_Bact  IGQKLTRGLG AGGNPSIGQK AAEESRDEIA TALEGADLVF ITAGMGGGTG
18Rivularia_sp__PCC_7116_Bacte  IGQKLTRGLG AGGNPAIGQK AAEESRDEIA TALEGADLVF ITAGMGGGTG
37Fischerella_sp__JSC-11_Bacte  IGQKLTRGLG AGGNPAIGQK AAEESRDEIA TALDGADLVF ITAGMGGGTG
43Calothrix_sp__PCC_6303_Bacte  IGQKLTRGLG AGGNPAIGQK AAEESRDELA AALEGADLVF ITAGMGGGTG
31Oscillatoriales_cyanobacteri  VGQKLTRGLG AGGNPAIGQK AAEESRDEIA TALEGADLVF ITAGMGGGTG
45Geitlerinema_sp__PCC_7407_Ba  IGQKLTRGLG AGGNPAIGQK AAEESRDELA AAIEGADLVF ITAGMGGGTG
17Synechocystis_sp__PCC_7509_B  IGQKLTRGLG AGGNPAIGQK AAEESRDEIA AALENADLVF ITSGMGGGTG
26Oscillatoria_acuminata_PCC_6  VGQKLTRGLG AGGNPAIGQK AAEESRDEIA HALEHSDLVF ITAGMGGGTG
16Gloeocapsa_sp__PCC_7428_Bact  IGQKLTRGLG AGGNPAIGQK AAEESREEIA AALENADLVF ITAGMGGGTG
19Coleofasciculus_chthonoplast  VGQKLTRGLG AGGNPAIGQK AAEESRDEIA QALAESDLVF ITAGMGGGTG
33Chlamydomonas_reinhardtii_Eu  IGGKLTRGLG AGGNPEIGAK AAEESRDSIA AALQDTDMVF VTAGMGGGTG
50Ostreococcus_lucimarinus_CCE  IGAELTRGLG AGGNPEIGQK AAEESRAAIE QALSGSDMVF VTAGMGGGTG
27Ricinus_communis_Eukaryota__  IGKELTRGLG AGGKPDVGKN AANESKLAIE EALSGADMVF VTAGMGGGTG
35Selaginella_moellendorffii_E  IGQKLTRGLG AGGNPEIGMS AAEESKAIVE EAVRGADMVF VTAGMGGGTG
42Physcomitrella_patens_subsp_  IGQKLTRGLG AGGNPEIGCS AAEESKAMVE EALRGADMVF VTAGMGGGTG
06Thalassiosira_pseudonana_CCM  IGRMVTRGLG AGGVPDVGKK SALENGEEIK QICKGADMVF ITAGMGGGTG
12Phaeodactylum_tricornutum_CC  IGRQLTRGLG AGGDPGVGRG AGEENIIEMQ HICDNTDLVF ITAGMGGGTG
100Ectocarpus_siliculosus_Loca  LGSKVTRGLG AGGKPEVGLA AATESLPEIE KTLAGADLVF VTAGMGGGTG
07Thalassiosira_pseudonana_CCM  IGSSVTRGLG AGGDPEIGRL AAEESREEIN AMVSGADLCF ITSGMGGGTG
08Phaeodactylum_tricornutum_CC  IGSAVTRGLG AGGDPDVGRM AAEESAQDIA AMIQGTDLCF VTSGMGGGTG
05Thalassiosira_pseudonana_CCM  IGTTATRGLG AGGNPDIGQL AAEESRAEIA AMVEGTDLCF VTSGMGGGTG
14Phaeodactylum_tricornutum_CC  IGASATRGLG AGGNPEIGRI AAEESRKEIA AMVTGTDLCF VTSGMGGGTG
108Emiliania_huxleyi_Localdb__  LGAGTSRGLG AGGSPDTGAA SAKESFEEIS KMVAGSDMVF VVAGMGGGTG
52Lotharella_amoeboformis_Euka  ---------- ---------- ---------- ---------- ----------
53Lotharella_amoeboformis_Euka  ---------- ---------- ---------- ---------- ----------
01QUERY_Bigel_39262             IGPEETKGLG AGSKPEVGKI AAEESAEVIG AIVRDTDMVF VTAGMGGGTG
55Cyanidioschyzon_merolae_Euka  IGNKLTRGLG AGGNPEVGRK AAEESCDQIA EAVRGADLVF VTAGMGGGTG
096Aureococcus_anophagefferens  IGRETTRGLG AGGKPSQGEA AAEESRAEIA AALSGADMVF VTAGMGGGTG
04Cryptomonas_paramecium_Eukar  IGAKLTRGLG AGGNPVIGKK AAEESKQLIG EIVSSGDLVF ITAGMGGGTG
02Hemiselmis_andersenii_Eukary  IGAKLTRGLG AGGNPEIGRK AAEESRDLIG EAVSAGDLVF VTAGMGGGTG
03Guillardia_theta_Eukaryota__  IGAKLTRGLG AGGNPEIGRK AAEESRDLIA EAVSAGDLVF VTAGMGGGTG

                               101
54Cyanidioschyzon_merolae_Euka  TGAAPVVARI AKSLGALTVG VVTKPFSFEG RHRMQQALDG VAALRENVDT
40Ostreococcus_tauri_Eukaryota  SGSAPVVAKL SKAKGILTVG VVTYPFSFEG RRRIQQATEA IEALRANVDT
23Micromonas_sp__RCC299_Eukary  SGSAPVVARL SREAGNLTVG VVTQPFTFEG RRRFIQAQES IEQLRANVDT
34Volvox_carteri_f__nagariensi  TGAAPVVARI SKELGILTVG VVTYPFNFEG RRRAGQALEG IEGLRAAVDS
```

```
39Chlamydomonas_reinhardtii_Eu   TGAAPVVARL SKELGILTVG VVTYPFNFEG RRRAGQALEG IEALREAVDS
36Brachypodium_distachyon_Euka   SGAAPVVAQI AKEAGYLTVG VVTYPFSFEG RKRSLQALEA LEKLERSVDT
29Physcomitrella_patens_subsp_   SGAAPVVARV AKEAGQLTVG VVTYPFTFEG RRRSQQAVEA IENLRKSVDS
30Selaginella_moellendorffii_E   SGAAPVVARL SKEKGQLTVG VVTYPFTFEG RRRSQQALDA IERLRSNVDT
47Prochlorococcus_marinus_subs   TGAAPVVAEV AKQSGALTVG IVTKPFSFEG KRRMRQAEEG IARLAENVDT
51Synechococcus_sp__CC9311_Bac   TGAAPVVAEV AKESGALTVG IVTKPFSFEG RRRMRQADEG IERLAEHVDT
21Synechococcus_sp__JA-3-3Ab_B   TGGAPIVAQI AKASGALTVG VVTRPFSFEG KRRTKQAEEG IQALQEAVDT
38Chamaesiphon_minutus_PCC_660   TGAARIVAEV AKEMGALTVG VVTRPFTFEG RRRTNQAEEG ISGLQSQVDT
10Cyanothece_sp__PCC_8802_Bact   TGAAPIVAEV AKEMGCLTVG VVTRPFTFEG RRRTSQASQG VEKLQNNVDT
20Stanieria_cyanosphaera_PCC_7   TGAAPIAAEV AKEMGCLTVG VVTRPFTFEG RRRTNQAEEG IAAFGTRVDT
32Gloeobacter_violaceus_PCC_74   TGAAAIVAEA AKEVGALTVA VVTRPFTFEG RRRMQQADSG IEALQGRVDT
25Gloeocapsa_sp__PCC_73106_Bac   TGAAPIVAEV AKEMGCLTVG VVTRPFTFEG RRRTNQAEEG INALQTRVDT
24Chroococcidiopsis_thermalis_   TGAAPTVAEI AKEKGILTVG VVTRPFGFEG RRRANQAHQG IDALKDRVDT
09Cyanobacterium_stanieri_PCC_   TGAAPIVAEV AKEMGCLTVG VVTRPFTFEG RRRTTQADDG ISALQSRVDT
28Synechocystis_sp__PCC_6803_B   TGAAPIVAEV AKEMGCLTVG IVTRPFTFEG RRRAKQAEEG INALQSRVDT
15Pleurocapsa_sp__PCC_7327_Bac   TGAAPIVAEV AKEIGCLTVG VVTRPFTFEG RRRNSHAEEG VSNLQSRVDT
11Cyanothece_sp__PCC_7822_Bact   TGAAPIVAEV AKEMGCLTVG VVTRPFTFEG RRRTNQAEDG ISGLQSRVDT
13Microcystis_aeruginosa_PCC_9   TGAAPIVAEI AKEIGCLTVG VVTRPFTFEG RRRTNQADEG VGGLQSRVDT
41Leptolyngbya_sp__PCC_7376_Ba   TGAAPVVAEI AKDLGCLTVG VVTRPFKFEG RRRTNQAEEG ITALQSRVDT
22Synechococcus_sp__PCC_7502_B   TGAAPVIAEI AKEAGALTVG IVTRPFTFEG RRRLQQAEEG IDALQSRVDT
44Raphidiopsis_brookii_D9_Bact   TGAAPIVAEV AKEMGALTVG VVTRPFVFEG RRRTSQAEQG IEGLKSRVDT
18Rivularia_sp__PCC_7116_Bacte   TGAASVVAEV AKEMGALTVG VVTRPFVFEG RRRTTQAEQG VEALKSRVDT
37Fischerella_sp__JSC-11_Bacte   TGAAPIVAEV AKEMGALTVG VVTRPFIFEG RRRISQAEQG IEGLKSRVDT
43Calothrix_sp__PCC_6303_Bacte   TGAAPVVAEV AKEMGALTVG VVTRPFIFEG RRRISQSEQG IEGLKSRVDT
31Oscillatoriales_cyanobacteri   TGAAPIVAEV AKELGALTVG IVTRPFTFEG RRRASQAAEG IEALQSRVDT
45Geitlerinema_sp__PCC_7407_Ba   TGAAPVVAEV AKEAGALTVG VVTRPFTFEG RRRTNQAEEG TAALQGRVDT
17Synechocystis_sp__PCC_7509_B   TGAAPIVAEI AKEMGALTVG VVTRPFIFEG RRRTSQAEQG IEGLESRVDT
26Oscillatoria_acuminata_PCC_6   TGAAPIVAEA AKEVGALTVG VVTRPFMFEG RRRTNQAEEG IAALQSRVDT
16Gloeocapsa_sp__PCC_7428_Bact   TGAAPIVAEV AKELGALTVG VITRPFMFEG RRRTSQAEQG IEALQSRVDT
19Coleofasciculus_chthonoplast   TGAAPIVAEI AKEMGALTVG VVTRPFTFEG RRRTSQAEEG IAALQSRVDT
33Chlamydomonas_reinhardtii_Eu   SGAAPVVAQV ARELGILTVG IVTTPFTFEG RQRAQQARSA LANLRAAVDT
50Ostreococcus_lucimarinus_CCE   SGAAPVVAQV AKSAGILTVG IVTMPFKFEG RQRYNQAMEA VERLRQNVDT
27Ricinus_communis_Eukaryota__   TGGAPVVAGI SKSLGLLTVG IVTTPFSFEG RKRTIQAQEG IAALRNNVDT
35Selaginella_moellendorffii_E   SGAAPVIAGV AKELGVLTVG IVTTPFSFEG RRRSIQAQEA TALLKNNVDT
42Physcomitrella_patens_subsp_   SGAAPIIAGV AKQLGILTVG IVTTPFAFEG RRRAVQAHEG IAALKNNVDT
06Thalassiosira_pseudonana_CCM   SGAGPVVAEI ARDEGCLTVG VVTKPFAFEG KKRMQQAEGA IKELRKHVDT
12Phaeodactylum_tricornutum_CC   SGAAPVLAKI AKQDGCLTVG VVTKPFAFEG RKRMMQAEAA IEELRKNVDT
100Ectocarpus_siliculosus_Loca   TGAAPVIASA AKGMGCVTVA VVTEPFGFEG RQRSRQAAAG LAELREAADT
07Thalassiosira_pseudonana_CCM   SGAAPVVAEV SKESGALTVA IVTKPFAFEG RRRMRQATEA IDRLRQNVDT
08Phaeodactylum_tricornutum_CC   SGAAPVVSEI AKESGALTVA IVTKPFAFEG RRRMRQATDA IDRLRQHVDT
```

```
05Thalassiosira_pseudonana_CCM  SGAAPVVAEV SKEAGALTIG IVTKPFRFEG KRRMRQAVEA IGRLRDHVDT
14Phaeodactylum_tricornutum_CC  SGAAPVVAEV AKEEGCLTVG IVTKPFAFEG KRRMKQAIAA IERLRENVDT
108Emiliania_huxleyi_Localdb__  SGGAPIVAEA AKAAGALTVG VVTKPFSFEG QRRMRQADAA IDQLRQHVDA
52Lotharella_amoeboformis_Euka  ---------- ---------- ---------- ---------- ----------
53Lotharella_amoeboformis_Euka  ---------- ---------- ---------- ---------- ----------
01QUERY_Bigel_39262             SGAAPVVAAI SKHTGCLTVG VVTKPFHFEG KKRLRQAREA IQELRKHVDI
55Cyanidioschyzon_merolae_Euka  SGAAPVVAEA AREQGCLTVG VVTKPFAFEG RKRMNQALEA IEALRESVDT
096Aureococcus_anophagefferens  SGAAPIVASV AKELGALTVG VVTKPFGFEG RKRAQQAQVA TRNLQEAVDT
04Cryptomonas_paramecium_Eukar  SGAAPVIAEI AKELGCLTIA VVTKPFVFEG KKRMQQAIDG IAELKNRVDT
02Hemiselmis_andersenii_Eukary  SGAAPVVAEV AKEMGCLTVG VVTKPFGFEG RRRMQQATDA ITNLRERVDT
03Guillardia_theta_Eukaryota__  SGAAPIVAEV AKEMGCLTVG VVTKPFAFEG KRRMQQANDA ILNLRNKVDT

                               151
54Cyanidioschyzon_merolae_Euka  LIVVSNDRLM HVVPKNMPLK RAFRVADDVL KNGVRGISEL ITRPGLINVD
40Ostreococcus_tauri_Eukaryota  LIVIPNDRLL DVVEEGTPLQ EAFLLADDVL RQGVQGISDI ITIPGLVNVD
23Micromonas_sp__RCC299_Eukary  LIVIPNDRLL DVVMDDAPLQ EAFLLADDVL RQGVQGISDI ITISGLVNVD
34Volvox_carteri_f__nagariensi  VIVIPNDRLL DVASASTALQ DAFALADDVL RQGVQGISDI ITVPGLINVD
39Chlamydomonas_reinhardtii_Eu  VIVIPNDRLL DVAGASTALQ DAFALADDVL RQGVQGISDI ITVPGLINVD
36Brachypodium_distachyon_Euka  LIVIPNDRLL DVADENMPLQ DAFLLADDVL RQGVQGISDI ITIPGLVNVD
29Physcomitrella_patens_subsp_  LIVIPNDRLL DVSGDKTPLQ EAFSLADDVL RQGVQGISDI ITTPGLVNVD
30Selaginella_moellendorffii_E  LIVIPNDRLL DLVQEHTPLQ EAFLLADDVL RQGVQGISDI ITIPGLVNVD
47Prochlorococcus_marinus_subs  LIVIPNDRLK DVIAG-APLQ EAFRNADDVL RMGVKGISDI ITCPGLVNVD
51Synechococcus_sp__CC9311_Bac  LIVIPNDRLR DAIAG-APLQ EAFRSADDVL RMGVKGISDI ITLPGLVNVD
21Synechococcus_sp__JA-3-3Ab_B  LIVIPNDKLL SVISEQTPVH EAFRVADDVL RQGVQGISDI ILIPGMINVD
38Chamaesiphon_minutus_PCC_660  LIIIPNDKLL QAINEQTPVQ EAFRIADDVL RSGVQGISDI ITIPGLINVD
10Cyanothece_sp__PCC_8802_Bact  LIVIPNNQLL QVIPPDTPLQ QAFLAADNIL RQGVQGISDI ITIPGLVNVD
20Stanieria_cyanosphaera_PCC_7  LIVIPNNQLL TVISPETPMQ EAFRIADDVL RQGVQGISDI ITIPGLINVD
32Gloeobacter_violaceus_PCC_74  LIVIPNDKLL SVISEQTPVQ EAFRIADDIL RQGVQGISDI ITIPGLINVD
25Gloeocapsa_sp__PCC_73106_Bac  LIVIPNNQLL SVINPDTPMQ EAFRTADDIL RQGVQGISDI ITIPGLVNVD
24Chroococcidiopsis_thermalis_  MILIPNDKLL SVISEQTALR DAFLTADEVL RQGVQGISDI ITIPGLVNVD
09Cyanobacterium_stanieri_PCC_  LIVIPNNKLL SVIPSDTPLQ ESFRIADDIL RQGVQGISDI ITIPGLVNVD
28Synechocystis_sp__PCC_6803_B  LIVIPNNQLL SVIPAETPLQ EAFRVADDIL RQGVQGISDI IIIPGLVNVD
15Pleurocapsa_sp__PCC_7327_Bac  LIVIPNNQLL AVANAETPMQ EAFRMADDIL RQGVQGISDI ITIPGLINVD
11Cyanothece_sp__PCC_7822_Bact  LIVIPNNQLL AVIPQDTPLQ DAFRAADDIL RQGVQGISDI ITIPGLVNVD
13Microcystis_aeruginosa_PCC_9  LIIIPNNQLL QVIPADTPLQ EAFRVADDVL RQGVQGISDI ITIPGLVNVD
41Leptolyngbya_sp__PCC_7376_Ba  LLVIPNNQLL NVIAPETPMQ EAFRIADDIL RQGVQGISDI ITIPGLVNVD
22Synechococcus_sp__PCC_7502_B  LIIIPNNKLL SVTAEQTPIQ EAFRVADDIL RQGVQGISDM ITIPGLVNVD
44Raphidiopsis_brookii_D9_Bact  LIIIPNNKLL EVIPEQTPVQ EAFRYADDVL RQGVQGISDI ITIPGLVNVD
18Rivularia_sp__PCC_7116_Bacte  LIIIPNNKLL EVIPEQTPVQ EAFRYADDVL RQGVQGISDI ITIPGLVNVD
37Fischerella_sp__JSC-11_Bacte  LIIIPNNKLL EVIPEQTPMQ EAFRYADDVL RQGVQGISDI ITIPGLINVD
```

```
43Calothrix_sp__PCC_6303_Bacte  LIIIPNNKLL EVIPEQTPMQ EAFRYADDVL RQGVQGISDI ITIPGLINVD
31Oscillatoriales_cyanobacteri  LIMIPNDKLL SVISEQTPVQ EAFRAADDIL RQGVQGISDI ITIRGLVNVD
45Geitlerinema_sp__PCC_7407_Ba  LIIIPNDKLL SVISEQTPVQ EAFRVADDIL RQGVQGISDI ITIPGLVNVD
17Synechocystis_sp__PCC_7509_B  LIVIPNDKLL SVISEQTPVQ EAFRFADDIL RQGVQGISDI ITIPGLVNVD
26Oscillatoria_acuminata_PCC_6  LIVIPNDKLL SVISEQTPVQ EAFRVADDIL RQGVQGISDI ITIPGLVNVD
16Gloeocapsa_sp__PCC_7428_Bact  LIVIPNDKLL SVISEQTPVQ EAFRIADDIL RQGVQGISDI ITIPGLVNVD
19Coleofasciculus_chthonoplast  LIVIPNNKLL SVISEQTPVQ DAFRVADDIL RQGVQGISDI ITVPGLVNVD
33Chlamydomonas_reinhardtii_Eu  LIVIPNDRLL SAMDSNVPIK DAFKIADDVL RQGVKGISEI ITVPGLVNVD
50Ostreococcus_lucimarinus_CCE  LIVIPNDRLL AAVDASLPVQ DAFLLADDIL RQGVRGITDI ITLPGLINVD
27Ricinus_communis_Eukaryota__  LIVIPNDKLL AAVSPSTPVT EAFNLADDIL RQGVRGISDI ITIPGLVNVD
35Selaginella_moellendorffii_E  LITIPNDKLL TAVSQSTPVT EAFNLADDIL RQGVRGISDI ITIPGLVNVD
42Physcomitrella_patens_subsp_  LITIPNNKLL TAVAQSTPVT EAFNLADDIL RQGVRGISDI ITVPGLVNVD
06Thalassiosira_pseudonana_CCM  LIVVSNDKLL RIVPENTPVT DAFLVADDIL RQGVVGISEI IIKTGLVNVD
12Phaeodactylum_tricornutum_CC  LIVVSNDKLL RIVPDNTPVT EAFLVADDIL RQGVVGISEI ILKTGLVNVD
100Ectocarpus_siliculosus_Loca  VLVVANDKLL EIVPGRMTMK DAFLVADDVL RQGVIGTSEL IVRPGLINVD
07Thalassiosira_pseudonana_CCM  VIIVSNNKLL DIIPENTPLE ASFRVADDIL RQGVVGISEI IVRPGLINVD
08Phaeodactylum_tricornutum_CC  VIIVSNNKLL EIIPDDTPVT AAFRVADDIL RQGVVGISEI IVRPGLINVD
05Thalassiosira_pseudonana_CCM  VIVVSNDRLL DIIPEDTPMN RAFAVADDIL RQGVVGISEI IVKPGLINVD
14Phaeodactylum_tricornutum_CC  VIVVSNDRLL EIIPDDTPME RAFAVADDIL RQGVVGISDI IVKPGLINVD
108Emiliania_huxleyi_Localdb__  MIVVSNDRLL DLVEEGTPLQ EAFCVADDIL RQGVVGISDI IIQPGLINVD
52Lotharella_amoeboformis_Euka  ---------- ---------- ---------- ---------- -----LINVD
53Lotharella_amoeboformis_Euka  ---------- ---------- ------DDIL RQGIVGISEI ITKPGLINVD
01QUERY_Bigel_39262             LIVVSNDKLL EVAPTGMALQ DAFAQADDIL RQGIVGISEI ITKPGLINVD
55Cyanidioschyzon_merolae_Euka  LIVVSNDKLL QIVPENTPLQ DAFRVADDIL RQGVVGISDI IIRPGLINVD
096Aureococcus_anophagefferens  LIVISNDRLL QIVPEGTTME GAFLVADDIL RQGVVGISEI IIKPGLINVD
04Cryptomonas_paramecium_Eukar  LIVVSNDKLL KIIPENTPLQ DAFSVADDVL RQGVVGISEI IIKPGLINVD
02Hemiselmis_andersenii_Eukary  LIVVSNDKLL QIVPDNTPLQ DAFSVADDIL RQGVVGISEI IVRPGLINVD
03Guillardia_theta_Eukaryota__  LIVVSNDKLL QIVPDNTPLQ DAFSVADDIL RQGVVGISEI IVRPGLINVD

                                201
54Cyanidioschyzon_merolae_Euka  FADVRSVMAE KGYALLGLGT GSGERRAKEA ALAAVSSPLL DFPLNSAKGA
40Ostreococcus_tauri_Eukaryota  FADVRTVMKD SGTAMLGVGV ASGKNRAEEA ARAAMSAPLV EHSIDRAMGI
23Micromonas_sp__RCC299_Eukary  FADVKAVMKG SGTAMLGVGV AQGKNRAEEA ATAAISAPLI EHSIDRATGI
34Volvox_carteri_f__nagariensi  FADVKAIMSN SGTAMLGVGA ASGPDRAEQA AMAATSAPLI QRSIEKATGI
39Chlamydomonas_reinhardtii_Eu  FADVKAIMSN SGTAMLGVGA ASGPDRAEQA AVAATSAPLI QRSIEKATGI
36Brachypodium_distachyon_Euka  FADVKAVMKN SGTAMLGVGV SSSKNRAQEA AEQATLAPLI GSSIEAATGV
29Physcomitrella_patens_subsp_  FADVRAVMSN SGTAMLGVGS SSGKNRAEEA AIQAASAPLI ERSIEQATGI
30Selaginella_moellendorffii_E  FADVKAIMTN SGTAMLGVGT ASGKNRAEEA AQQATSAPLI ERSIERATGV
47Prochlorococcus_marinus_subs  FADVRSVMTE AGTALLGIGI GSGRSRALEA AQAAMNSPLL EAAIDGAKGC
51Synechococcus_sp__CC9311_Bac  FADVRSVMTE AGTALLGIGV GSGRSRAVEA AQTAINSPLL EAAIDGASGC
```

```
21Synechococcus_sp__JA-3-3Ab_B  FADVRSVMAD AGTALMGIGM GSGKSRAREA AITAVSSPLL ETSIEGAKGV
38Chamaesiphon_minutus_PCC_660  FADVRTVMAD AGSALMGIGE GSGKSRAREA AVQAINSPLL ESSIEGARGV
10Cyanothece_sp__PCC_8802_Bact  FADVRAVMAD AGSALMGLGI GSGKSRASDA AVAAISSPLL EHSIKGARGV
20Stanieria_cyanosphaera_PCC_7  FADVRAIMAD AGSALMGIGI GSGKSRAREA AIAAVSSPLL EASIEGAKGV
32Gloeobacter_violaceus_PCC_74  FADVRAIMAD AGSALMGIGM GSGKSRAREA AMTAISSPLL ESSIEGANGV
25Gloeocapsa_sp__PCC_73106_Bac  FADVRAIMAD AGSAMMGIGI GTGKSRAKEG AIAAISSPLL ESSIEGAKGV
24Chroococcidiopsis_thermalis_  FADVKAVMAD AGSALMGIGT GSGKTRAREA ANAAISSPLL ESSIEGAKGV
09Cyanobacterium_stanieri_PCC_  FADVRAVMAD AGSALMGIGI GSGKSRAKES AVAAISSPLI ESSIQGAKGV
28Synechocystis_sp__PCC_6803_B  FADVRAVMAD AGSALMGIGV GSGKSRAKEA ATAAISSPLL ESSIQGAKGV
15Pleurocapsa_sp__PCC_7327_Bac  FADVRAIMAD AGSALMGIGI GTGKSRAKEG AIAAISSPLL ESSIEGAKGV
11Cyanothece_sp__PCC_7822_Bact  FADVRAVMAD AGSALMGIGV GSGKSRAKEG AIAAISSPLL EHSIEGAKGV
13Microcystis_aeruginosa_PCC_9  FADVRAVMAD AGSALMGIGI GSGKSRAKEG AIAAISSPLL ESSIEGAKGV
41Leptolyngbya_sp__PCC_7376_Ba  FADVRAVMAD AGSALMGIGV GSGKSRAREA AIAAISSPLM ESSVEGAKGV
22Synechococcus_sp__PCC_7502_B  FADIRAVMAD AGSALLGIGI GSGKSRAREA AMTAISSPLL ESSIEGANGV
44Raphidiopsis_brookii_D9_Bact  FADVRAVMAD AGSALMGIGV SSGKSRAREA AIAAISSPLL ESSIEGARGV
18Rivularia_sp__PCC_7116_Bacte  FADVRAVMAD AGSALMGIGI GSGKSRSREA AIAAISSPLL ECSIEGARGV
37Fischerella_sp__JSC-11_Bacte  FADVRAVMAD AGSALMGIGI GSGKSRAREA AIAAISSPLL ECSIEGARGV
43Calothrix_sp__PCC_6303_Bacte  FADVRAVMAD AGSALMGIGI GSGKSRAREA AIAAISSPLL ECSIEGARGV
31Oscillatoriales_cyanobacteri  FADVRAVMAD AGSALMGIGV GTGKSRAREA AVASISSPLL ESSIDGARGV
45Geitlerinema_sp__PCC_7407_Ba  FADVRAVMAD AGSALMGIGV GSGKSRAREA AMAAIASPLL ESSIDGAKGV
17Synechocystis_sp__PCC_7509_B  FADVRAIMAD AGSALMGIGV GSGKSRAQEA ANAAISSPLL ESSIEGARGV
26Oscillatoria_acuminata_PCC_6  FADVRAVMAD AGSALMGIGV GSGKSRAREA ALSAISSPLL ESSIEGAKGV
16Gloeocapsa_sp__PCC_7428_Bact  FADVRAIMAD AGSALMGIGI GSGKSRAREA ANAAISSPLL ESSIEGAKGV
19Coleofasciculus_chthonoplast  FADVRAVMAD AGSALMGIGV GSGKSRAREA AVAAISSPLL ESSVEGARGV
33Chlamydomonas_reinhardtii_Eu  FADVRAIMAG AGSSLMGQGY GSGPRRASDA ALRAISSPLL EVGIERATGV
50Ostreococcus_lucimarinus_CCE  FADVRAVMAD AGSSLMGIGR ASGKNRAREA AEAAISSPLL DLGIDRATGI
27Ricinus_communis_Eukaryota__  FADVQAIMKD SGSSLMGIGT ATGKSRARDA ALNAIQSPLL DIGIERATGV
35Selaginella_moellendorffii_E  FADVRAIMAN AGSSLMGIGT ATGKSRARDA ALNAIQSPLL DVGIERATGI
42Physcomitrella_patens_subsp_  FADVRAIMAN AGSSLMGIGT ATGKSRAREA ALSAIQSPLL DVGIERATGI
06Thalassiosira_pseudonana_CCM  FADVRAVMKD AGTALMGVGT GVGKTRATDA AVAAISSPLL DFPISEAKRI
12Phaeodactylum_tricornutum_CC  FADVRAVMKD AGTALMGVGT GVGKNRASDA ALAAISSPLL DFPIQRAKRI
100Ectocarpus_siliculosus_Loca  FADVRQVITN SGTALIGIGM GSGKTRAEDA AVGAIVSPLL EFSIDQAAGV
07Thalassiosira_pseudonana_CCM  FADVRSVMQD AGTALMGIGT GSGKTSAEDA AVAAISSPLL DAPVDEATGV
08Phaeodactylum_tricornutum_CC  FADVRSVMKD AGSALMGIGT GVGKTSAEDA AIAAISSPLL DEPVQDATGV
05Thalassiosira_pseudonana_CCM  FADVRSVMSD AGTALMGIGI GSGKTGAEDA ATAAISSPLL DSSIDNAKGV
14Phaeodactylum_tricornutum_CC  FADVRSIMSG AGTALMGIGI GAGKTAAEDA AAAAISSPLL DSTIENAKGV
108Emiliania_huxleyi_Localdb__  FADVQSVMGA SGLALMGIGT AEGKTRARDA AFAA---PLI DFPIGQATGI
52Lotharella_amoeboformis_Euka  FADVRSVMQN AGLALMGIGR GSGKNRAQDA ALAAVTSPLL DFPIQQAKGV
53Lotharella_amoeboformis_Euka  FADVNSIMSN AGLALMGMGE GAGPSRARDA ALAAITSPLL DFPLRDAKGV
01QUERY_Bigel_39262             FADVNSVMSN AGLALMGIGV GSGPSRAQDA AMAAITSPLL DFPLENAKGV
```

```
55Cyanidioschyzon_merolae_Euka  FADVRSVMAH AGSALMGIGT GSGKSRAHDA AVAAISSPLL DFPIERAKGI
096Aureococcus_anophagefferens  FADVRSIMSD AGTALMGIGQ SKGKDRAAEA AGLATSCPLL DSQFMNAKAV
04Cryptomonas_paramecium_Eukar  FADIRSIMAE SGNALMGIGT ASGKNRAHDA SIAAISSPLL DFSIKDAKGI
02Hemiselmis_andersenii_Eukary  FADVRSVMAD AGSALMGIGT GSGKTRAQDA AVAAISSPLL DFPIEKAKGI
03Guillardia_theta_Eukaryota__  FADVRSVMAD AGSALMGIGT GSGKTRAQDA AVAAISSPLL DFPIEKARGI

                               251
54Cyanidioschyzon_merolae_Euka  VFNICGGPDM TLSEVNQCAE VIFQHLDPDA SIIFGATVDP TLPRDISVTV
40Ostreococcus_tauri_Eukaryota  VFNITGGPDM TLMEVNAVSE VVTSLADPNA NVIFGSVVDE KHRGEIAVTI
23Micromonas_sp__RCC299_Eukary  VYNITGGSDL TLQEINTVSE VITSLADPAA NIIFGAVVDD QYKGELQVTV
34Volvox_carteri_f__nagariensi  VYNITGGRDL TLAEVNRVSE VVTALADPSC NIIFGAVVDE QYDGELHVTI
39Chlamydomonas_reinhardtii_Eu  VYNITGGRDL TLAEVNRVSE VVTALADPSC NIIFGAVVDE QYDGELHVTI
36Brachypodium_distachyon_Euka  VYNITGGKDI TLQEVNKVSQ IVTSLADPSA NIIFGAVVDD RYTGEIHVTI
29Physcomitrella_patens_subsp_  VYNITGGSDL TLQEVNTVSQ IVTGLADPSA NIIFGAVVDD KYTGEVHVTI
30Selaginella_moellendorffii_E  VYNITGGRDL TLQEVNRVSQ VVTGLADPAA NIIFGAVVDE RYDGQVHVTI
47Prochlorococcus_marinus_subs  VINITGGKDM TLEDMTSASE IIYDVVDPEA NIIVGAVIDE SMEGEIQVTV
51Synechococcus_sp__CC9311_Bac  VINISGGRDM TLEDMTTASE VIYDVVDPEA NIIVGAVVDE RLEGEIHVTV
21Synechococcus_sp__JA-3-3Ab_B  LFNITGGLDL SLHEVTVAAE IIAEAVDPEA NIIFGTVQDE RMQGEVRITV
38Chamaesiphon_minutus_PCC_660  VLNITGGSDM TLIEVSTAAD TIYEVVDPNA NIIFGAVIDP QMQGEMRITV
10Cyanothece_sp__PCC_8802_Bact  VFNITGGDDL TLHEVNTAAE TIFEVVDPDA NIIFGAVIDP TLQGEVIITV
20Stanieria_cyanosphaera_PCC_7  VINITGGTDL SLHEVNAAAE SIYDVVDPDA NIIFGAVIDE RMQGEVCITV
32Gloeobacter_violaceus_PCC_74  VLNVTGGHDL TLHEVNEAAA VIYEVVDPNA NIIFGAVIDE KLQGELRITV
25Gloeocapsa_sp__PCC_73106_Bac  VLNITGGKDL TLHEVNAAAE IIYEIVDPNA NIIFGAVIDP EMQGEIRVTV
24Chroococcidiopsis_thermalis_  VINITGGSDM TLHEVNMAAD TIYEVVDPNA NIIFGAVIDD KLQGEMKITV
09Cyanobacterium_stanieri_PCC_  VLNITGGNDL TLHEVNTVAE TIYDIVDPNA NIIFGAVIDE SMQGEIRITV
28Synechocystis_sp__PCC_6803_B  VFNVTGGTDL TLHEVNVAAE IIYEVVDADA NIIFGAVIDD RLQGEMRITV
15Pleurocapsa_sp__PCC_7327_Bac  ILNITGGHDL TLHEVNAAAD IIYEIVDPNA NIIFGAVIDE KMQGEIRITA
11Cyanothece_sp__PCC_7822_Bact  VLNITGGSDL TLHEVNTAAE TIYEVVDPNA NIIFGAVIDE KMQGEILITV
13Microcystis_aeruginosa_PCC_9  VFNITGGQDL TLHEVNAAAE IIYEVVDPNA NIIFGAVIDE KMQGEVRITV
41Leptolyngbya_sp__PCC_7376_Ba  VLNITGGHDL TLHEVNAAAE AIYEVVDPNA NIIFGAVIDE QLQGEIRITV
22Synechococcus_sp__PCC_7502_B  VFNITGGSDL TLHEVNAAAE IIYEVVDPNA NIIFGAVIDE KMAGEVRITV
44Raphidiopsis_brookii_D9_Bact  VFNITGGSDL TLHEVNAAAE TIYEVVDPNA NIIFGAVIDD RLQGEVRITV
18Rivularia_sp__PCC_7116_Bacte  VFNITGGSDL TLHEVNAAAE AIYEVVDPNA NIIFGAVIDD RLEGEVRITV
37Fischerella_sp__JSC-11_Bacte  VFNITGGSDL TLHEVNAAAE TIYEVVDPNA NIIFGAVIDD RLQGEVRLTV
43Calothrix_sp__PCC_6303_Bacte  VFNITGGSDL TLHEVNAAAE TIYEVVDPNA NIIFGAVIDD RLQGEVRLTV
31Oscillatoriales_cyanobacteri  VFNITGGTDL TLHEVNAAAE IIYEVVDPNA NIIFGAVIDE RLQGELRITV
45Geitlerinema_sp__PCC_7407_Ba  VFNITGGHDL TLHEVNSAAE IIYEVVDPNA NIIFGAVIDE RMQGEIRITV
17Synechocystis_sp__PCC_7509_B  IFNITGGSDL TLHEVNAAAE IIYEVVDPNA NIIFGAVIDE RLQGEVRITV
26Oscillatoria_acuminata_PCC_6  VLNITGGTDL TLHEVNAAAE TVYEVVDPNA NIIFGAVIDE RLQGEIRITV
16Gloeocapsa_sp__PCC_7428_Bact  VFNITGGHDL TLHEVNAAAE TIYEVVDPNA NIIFGAVIDE KLQGEIRITV
```

```
19Coleofasciculus_chthonoplast  VFNITGGSDL TLHEVNAAAE TIYEVVDPNA NIIFGAVIDD RLQGEIRITV
33Chlamydomonas_reinhardtii_Eu  VWNITGPPNM TLHEVNEAAE IIYDMVDPNA NLIFGAVVDS TLDDTVSITI
50Ostreococcus_lucimarinus_CCE  VWNITGGSDL TLHEVNEAAE VIYDLVDPSA LIIFGAVVKD ATDGEVSITL
27Ricinus_communis_Eukaryota__  VWNITGGSDL KLFEVNTAAE VIYDLVDPSA NLIFGAVIDQ SLSGQVSITL
35Selaginella_moellendorffii_E  VWNITGGTDM TLFEVNAAAE VIYDLVDPNA NLIFGAVVDD SFNGHVSITL
42Physcomitrella_patens_subsp_  VWNITGGSDM TLFEVNAAAE VIYDLVDPNA NLIFGAVVDE ALHGQVSITL
06Thalassiosira_pseudonana_CCM  VFNVVGGPGL GLSEINAASE VIYENAHEDA NIIFGALIDP DMGEEVSITV
12Phaeodactylum_tricornutum_CC  VFNIVGGADM GLQEINEASE VIYENADDNA NIIFGALVDP QMDGQISITV
100Ectocarpus_siliculosus_Loca  IFNIVGGADM SLTEVNAAAS IIQRNVHPDA NIIIGALVDE RCGKEVSVTV
07Thalassiosira_pseudonana_CCM  VFNIIGGESL SLQEVDRAAK VIYNNVHEDA NVIFGALVDD EITDTVSITV
08Phaeodactylum_tricornutum_CC  VFNILGPRNL SLQEVNRAAR VIYDNVHEDA NVIFGALVDD DIEDEVSITV
05Thalassiosira_pseudonana_CCM  VFNISGGEGL SLTDVNRAAR LIYDSVEEDA NVIFGALIDE SLEDSISITV
14Phaeodactylum_tricornutum_CC  VFNISGGQNL SLNEVNQAAK LIYSTVEADA NVIFGALVDD TLEDNISITV
108Emiliania_huxleyi_Localdb__  VFTITGSSDM TLQEVNAAAE AIYEMADPDA NIIFGAQIDD SMGNVLSITV
52Lotharella_amoeboformis_Euka  VYTVTGNKDM SLTEVAQVSN VIQKMVGPDA NVIFGALIDE NMGDEMSVIV
53Lotharella_amoeboformis_Euka  VYTITGGPDM SLTEVNRVAE VIAEMVHPAA NVIFGANTDE SMGDKMKVVV
01QUERY_Bigel_39262             VYTINGGPDM SLQEVNRVAE VIAEMVHPEA NVIFGASTDE SMGNSNK---
55Cyanidioschyzon_merolae_Euka  VFNVTGGEDM TLHEINQAAE VIYEAVDPNA NIIFGALIDQ QMESEISITV
096Aureococcus_anophagefferens  VFNICGPPDL TLAEVNSAAG VIYENVAPDA NIIFGASVDE NMGQDVSVTV
04Cryptomonas_paramecium_Eukar  IFSIVGGHTM TLHEINTAAE IIYQAVDSNA NIIFGALVDD GMEDKISITV
02Hemiselmis_andersenii_Eukary  VFNITGGHDM TLHEINSAAE VIYEAVDPNA NIIFGALVDE NMENEISITV
03Guillardia_theta_Eukaryota__  VFNITGGQDM TLHEINSAAE VIYEAVDSNA NIIFGALVDD NMENEISITV

                                301
54Cyanidioschyzon_merolae_Euka  VATGF----- --
40Ostreococcus_tauri_Eukaryota  VATGFWNRGF LG
23Micromonas_sp__RCC299_Eukary  IATGFWQRSF LD
34Volvox_carteri_f__nagariensi  IATGFWSADF LG
39Chlamydomonas_reinhardtii_Eu  IATGFWSADF LG
36Brachypodium_distachyon_Euka  IATGFWSS-- --
29Physcomitrella_patens_subsp_  IATGFLFNGF L-
30Selaginella_moellendorffii_E  IATGFRFR-- --
47Prochlorococcus_marinus_subs  IATGFNKNEF LR
51Synechococcus_sp__CC9311_Bac  IATGFAPRDF LR
21Synechococcus_sp__JA-3-3Ab_B  IATGFSGDEF LR
38Chamaesiphon_minutus_PCC_660  IATGFNPDAF LQ
10Cyanothece_sp__PCC_8802_Bact  IATGFSTDDF LQ
20Stanieria_cyanosphaera_PCC_7  IATGFQIDDF LQ
32Gloeobacter_violaceus_PCC_74  IATGFEDDEF LR
25Gloeocapsa_sp__PCC_73106_Bac  IATGFSSDEF LQ
```

```
24Chroococcidiopsis_thermalis_  IATGFKPDEF LQ
09Cyanobacterium_stanieri_PCC_  IATGFLADEF LQ
28Synechocystis_sp__PCC_6803_B  IATGFAPDDF LQ
15Pleurocapsa_sp__PCC_7327_Bac  IATGFKPDPF LQ
11Cyanothece_sp__PCC_7822_Bact  IATGFKPDDF LQ
13Microcystis_aeruginosa_PCC_9  IATGFKPDDF LQ
41Leptolyngbya_sp__PCC_7376_Ba  IATGFRPDEF LQ
22Synechococcus_sp__PCC_7502_B  IATGFKPDDF LQ
44Raphidiopsis_brookii_D9_Bact  IATGFAPQDF LQ
18Rivularia_sp__PCC_7116_Bacte  IATGFKPEPF LQ
37Fischerella_sp__JSC-11_Bacte  IATGFGSDDF LR
43Calothrix_sp__PCC_6303_Bacte  IATGFKPDDF LR
31Oscillatoriales_cyanobacteri  IATGFGDDDF LR
45Geitlerinema_sp__PCC_7407_Ba  IATGFVGDEF LQ
17Synechocystis_sp__PCC_7509_B  IATGFKTDDF LQ
26Oscillatoria_acuminata_PCC_6  IATGFPPDDF LQ
16Gloeocapsa_sp__PCC_7428_Bact  IATGFKPDEF LQ
19Coleofasciculus_chthonoplast  IATGFRPDEF LQ
33Chlamydomonas_reinhardtii_Eu  IATGFGAEAF LR
50Ostreococcus_lucimarinus_CCE  IATGFGKESF LR
27Ricinus_communis_Eukaryota__  IATGFGSEKF LR
35Selaginella_moellendorffii_E  IATGFNNESF LR
42Physcomitrella_patens_subsp_  IATGFSNNSF LR
06Thalassiosira_pseudonana_CCM  LACDFVETGF FK
12Phaeodactylum_tricornutum_CC  LACDFADSNF LK
100Ectocarpus_siliculosus_Loca  LATGF----- --
07Thalassiosira_pseudonana_CCM  LATGFKYGDF LR
08Phaeodactylum_tricornutum_CC  LATGFRGADF LS
05Thalassiosira_pseudonana_CCM  LATGFEAESF LR
14Phaeodactylum_tricornutum_CC  LATGFPLDSF LK
108Emiliania_huxleyi_Localdb__  VAMFL----- --
52Lotharella_amoeboformis_Euka  VATNFAKMAL GR
53Lotharella_amoeboformis_Euka  VATNFNNQEE ED
01QUERY_Bigel_39262             ---------- --
55Cyanidioschyzon_merolae_Euka  VATGFPGGDF LR
096Aureococcus_anophagefferens  LATGF----- FG
04Cryptomonas_paramecium_Eukar  IATGF----- -K
02Hemiselmis_andersenii_Eukary  VATGF---DF WG
03Guillardia_theta_Eukaryota__  VATGF---DL WK
```

7-hp.txt

Alignment: HP

```
01QUERY_Bigel_hp_88555             WELDVYSRPV VN-GGKKLWE FMLCDATGRW KYLESLSPDE VNSKGIKKIL LN
62Lotharella_amoeboformis_Euka     ---------- ---------- ---------- ---------- ---------- --
08Guillardia_theta_Eukaryota__     WELDFFSRPV ILDDGKKLWE LIIVNKDKSL QIIESVPNNM VNSKELRRKL LN
11Hemiselmis_andersenii_Eukary     WELDFFSRPV VDENGKKLWE IIIVDQKGNF EHIETVPNNL VNSKELKKRI KI
64Cyanidioschyzon_merolae_Euka     WELDFYSRPV VGADGKRLWE LVVCDRDGSF VHVEAFPNNM VNSRELARAV KT
093Aureococcus_anophagefferens     WELDCFSRPV L-VKGKKLWE LLITDASGQW RDVVALPATG VNSVAVRKAI ED
095Ectocarpus_siliculosus_Loca     WELDVYSRPV VGADGKKLWE LLICDSTGNF RHVSPIPSNM VNSREVRRTI EG
02Phaeodactylum_tricornutum_CC     WELDCYSRPV -AVAGKKLWE VLITDSAGSF RFRQTLPSNQ VNSKTLRQIV DD
03Thalassiosira_pseudonana_CCM     WELDCYSRPV LVDGKKKLWE ILMTDSSGNM KVCRALPSNK VNSREVRRVV EE
63Calliarthron_tuberculosum_Eu     WELDFYSRPV IGLDGKKLWE LIITDTTGAF EHVEAIPNSL VNSRDLKKRI SA
67Porphyridium_cruentum_Eukary     WELDFYSRPV LGEDGKKIWE LLITDKDGVL EHVEQVPSSS VNSTELRKRV AA
115Pyropia_yezoensis_Localdb__     WELDFYSRPV YGSDGKKRWE LLITDKNGVW SHVEVFANAD VNSANLRSRV AA
09Leptolyngbya_sp__PCC_6406_Ba     WELDFYSRPI LDERNKRRWE VLISEGLQRF RFSQFLANTD VNSLKLKEVI ET
05Oscillatoriales_cyanobacteri     WELDFYSRPI LDEHGKKVWE VVLCESPTQF RFAEYCASTE VNSERLVQAL QT
04Oscillatoria_acuminata_PCC_6     WELDFYSKPI LDENGKKRWE VLICESPTDL RFSKYCSSSE VNSIWLGNAI NE
38Gloeocapsa_sp__PCC_73106_Bac     WELDFYSRPI LDENQKKLWE VLICESPQQY KYAQFCPSTS VNSLWLAEAI KQ
06Synechocystis_sp__PCC_7509_B     WEIDFYSRPV LDENNKKLWE ILVCESPLSF KYSEYCSSSQ VNSAWLKAAL EK
17Gloeocapsa_sp__PCC_7428_Bact     WEIDFYSRPI LDENQKKIWE VLVCESLTDF RFAKYCPSTQ VNSVWLRTAL EE
14Chroococcidiopsis_thermalis_     WEIDFYSRPI LDENQKKVWE VVVCESPLDF RYAQYCPSTQ VNSAWLRTAL QE
20Calothrix_sp__PCC_6303_Bacte     WELDFYSRPI LDENQKKVWE LLLCESPKDF RYAQYCPSTE VNSAWLRTAI QE
21Rivularia_sp__PCC_7116_Bacte     WELDYYSRPI LDENKKKVWE VLICETPLDF RYAKYCSSAT VNSVWLQTAL QE
27Nostoc_sp__PCC_7107_Bacteria     WEIDFYSRPI LDENQKKVWE VVVCESPLDF RYAQYCPSTE VNSGWLRTAL QE
16Cylindrospermopsis_raciborsk     WELDFYSRPI LDANQKKVWE VLICESPTDF RYAQYCPSTQ VNSVWLRQAL QE
29Fischerella_sp__JSC-11_Bacte     WELDFYSRPI LDENQKKVWE VLVCESPLDF RYAQYCPSTQ VNSVWLRTAL QE
10Cyanobacterium_aponinum_PCC_     WELDFYSRPI IDENNKKRWE ILICESPTTF RYSQFCANTE VNSITLQNAI AT
24Halothece_sp__PCC_7418_Bacte     WELDFYSRPI RDENNKKLWE VLICESPLQF RYSKFCSAQN VNSIFLQEAL NE
15Crinalium_epipsammum_PCC_933     WELDFYSRPI IDENQKKIWE VLVCESPVDF RYAQYCPSTQ VNSVSLQNAL TE
07Microcoleus_sp__PCC_7113_Bac     WELDFYSRPI LDENQKKIWE ILVCESPLDF QYTQFCPSQQ VNSIWLREAL AE
25Microcystis_sp__T1-4_Bacteri     WELDFYSRPV LDENNKKRWE LLICETPATF KYASYCPNTM VNSQWLGEAI TA
28Stanieria_cyanosphaera_PCC_7     WELDFYSRPI LDEENKKVWE VLICESLTDF RYSQYCSSKT VNSLWLREAI EK
30Cyanothece_sp__PCC_8801_Bact     WELDFYSRPI LDENQKKLWE VVICETPLTF KYSQFCSSQT VNSVWLREAI ES
19Pleurocapsa_sp__PCC_7327_Bac     WELDFYSRPI LDENNKKLWE VLICETPTDF KYSQFCSNQS VNSLWLQQEI EK
23Cyanothece_sp__PCC_7424_Bact     WELDFYSRPI LDENKKKLWE VLICQAPTEF KYSEFCSNTT VNSLWLGEAI KK
12Chlamydomonas_reinhardtii_Eu     WEIDFCSRPL LDERGKKVWE LLICDPERNF EYSEYFPNSK INSAELKRTI ER
13Volvox_carteri_f__nagariensi     WEIDFCSRPL LDERGKKVWE LLICDPERKF EYSEYFPNSK INSAELKRAI ER
22Micromonas_sp__RCC299_Eukary     WQLDFCSRPM KDERGKKMWE LLICDETRSF EHSEFFPNNR INSVELAKAI DR
41Ostreococcus_tauri_Eukaryota     WQIDFCSRPL RDDRGKKVWE LLVTDDERSF EHAEYFPNNR INSVELARAL ER
36Selaginella_moellendorffii_E     WQLDFCSRPI FDDRGKRMWE LIICDAKRQL EFARFYPSNV INSTTLKNAI AE
```

```
26Physcomitrella_patens_subsp_  WELDFCSRPI LDSRGKKLWE LVVCDSRRQL QFTRFFPNNV INSVTLRDAL LY
33Vitis_vinifera_Eukaryota__Vi  WELDFCSRPI LDIRGKKIWE LLVCDSSLSL QYTKYFPNNV INSVTLKNAI ES

                                PTNIRFFRKQ AVSIIRKAMD SLEIRAIKSR STYALFDWLE ERSKNVYPFM EG
                                ---------- ---------- ---------- ---------- ---------- --
                                PDVIKFFRAQ MFNMISIALS DLDINVKPSR RTYALFEIIR EREKTIYPEM IG
                                PKVIKFFRSQ MFNMINIALS DLDLIVRPSR RTFSLYNKIS EREEKIYPNM KG
                                PRIIRFFRAQ MRNMIQIAMQ NISVETRPSR RTYALFLALA YRERNVYPRL PG
                                PTVIRFFRRQ MLNMLTIALN GVALRVTPSR ATHALYDWIE EREADVYPGM EG
                                PTVIRFFRNA MFNMIDIALK EVEVAVKPCR TTYAMYQWLE ERERDVYPAM AG
                                PNTIRFFRGA MFNMINIALM ELPVTSKPSR CTFALASWLE DRHENVYPQM EG
                                PSTIRFFRGA MFNMINIALS EIDVIAKPSR CTFALAQWIE DRNRDVYPKM EG
                                PTIIRFFRMQ MRNMISIALA DLNIQVRPSR RTYALQQAIR YREANVYPGM PG
                                PKVIRFFRGA MRNMISIALS EFDVVVKPSR RTYSLFALQR HRDKNVYPAM PG
                                PTTIRFFRAA MFNMISIALS GLDVVAAPSR RTYALFDMVA DRQANVYPSL EG
                                PSRVRFFRFS MQTMITRACE DLGLAATPSR RTLALQDWID YRQREVYPQD PG
                                PSRIRFFRQA MKNMITKACN DLNLPSVLSR RTYALNQWLQ QRFAEEYPKH PG
                                PTQIRFFRRQ MNNMITKACK DLGINSKPSR RTVALYRWLQ DRMDTVYPLE PG
                                PSKIRFFRRQ MKNMITKACE EVAVIPVPSR RTHTLNHWIV ERLKNHYPTL DN
                                PLKFRFFRTS MNNMIVKACQ DLGIPAQPSR RTLALHQWLQ QRNLDVYPLE PG
                                PVKFRFFRRQ MNNMITKACE DLGIPAQPSR RTLALNQWLQ QRMEEVYPHE PG
                                PTKFRFFRRQ MNNMITKACK DLGIPAQPSR RTLALLQLLK ERMDEVYPQE PG
                                PTRIRFFRRQ MNNMITKACQ DSGIPAQSSR RILVLHQWLQ QRMDEVYPQE PG
                                PVKIRFFRRQ MNNMITKACE EIGIPAQTSR RTLALNQWLQ QRMDEVYPQE AG
                                PIKVRFFRRQ MNNMITKACQ DLGIPAQPSR RTLLLNQWLQ QRMEEVYPQE PG
                                PIKIRFFRRQ MNNMITKACQ DMGIPALPSR KTLVLNQWIQ QRMEEVYPQE PG
                                PIKIRFFRRQ MNNMITKACG DIGIPAQPSR RTLVLNQWLQ QRIEQVYPQE PG
                                PSKIRFFRRQ MNNMILKGCE DAGIPALASR HTYTLNQWLE ERMTSFYPLQ EG
                                PKKIRFFRRQ MNNMITKACE DLEITALPSR RTYALQKWLQ ERLDQVYPQQ EG
                                PQKIRFFRRQ MNNMIVKACT DLGILAEPSR RTYAVHQWLR ERMQDVYPSH PN
                                PEKIRFFRRQ MTNMITKACE ELGIQVIPSR RTYTLERWLE QRILGFYPKH PG
                                PKKIRFFRRQ MNNMISKACE DIGIPASPSR RTHALTRWIE ERMVNFYPQE VG
                                PKKIRFFRRQ MNNMITKACE DAGIAAAPSS RTYALNHWLA TRMKEVYPQE PG
                                PQKIRFFRRQ MNNMITKACE DAGIAAVPSR RTYTLTHWLA ERDQQFYPTQ PG
                                PKKIRFFRRQ MNNMIVKACE DLGIPPAPSR RTYALERWLS QRLDEFYPNQ PG
                                PKKIRFFRRQ MNNMISKACE DAGIDPAPSR RTYALNQWIE ERMRDVYPQQ EG
                                PEKARFFRSQ MQTIITKALT DCQIKAVPSR RCFTVMSWIN ERLESVYKQD PR
                                PEKARFFRSQ MQTIITKALT DCQIKAVPSR RCFTVMSWIN ERLDSVYKTD PR
                                PRRFKFFRSQ MQTIITRACG EVGVNPLPSR RCQTMSRWLD ERLETVYKTH PG
                                PRRFKFFRAQ MQTIITRACT EVDVEALASR RCQTMTNWLD ERVESVYKKH PG
```

```
PTRVRYFRSQ VKTIISKACG ELGIQVTSSQ RCTALVRWLH ERYDQVYRQH PG
PEKIRFFRSQ MQTIITKACK ELDIQPVPSQ RCVTLIKWLE ERFETVVYSQH PG
PEKIRFFRSQ MQTIVTKACK ELGIKPIPSK RCLSLILWLE ERYETVVYTRH PG

FVYTRP-SEK LPMYLEGEDW SFSEVQLADI IENTKDMKEA GRVCPISIPK ST
---------- ---------- ----VQLDEI LRNTKDMTEP GRVCEVGYDG KT
EDLSKRFPQR MPDILLGENF SFVLASLEEI NVILKDQSVM KDSFKINKYD ID
FNASKKVPQK MPDALRGEKY IFASLSSDEL SSINSSDIAF SGFCPLEFDK NQ
LSLDLKVAAR LPDELQGDRF AFVTILLRDV TAA-----GF GELCPVGAPS ET
RMTAPVTASR LPEGLRGEQY AFVTLPLSEV LSGGGINVGV GKLINVAYEV DA
-FFDIRTPTP LPDALRGEQY AFVTMPVSEF RQ-GNINVGV GRLCPLSLPD DA
SFLDVRTPVR LPDALRGEKY AFVALPVAEF LPGGSVNIGV GRICTIDIPA DA
TFLDIRTAVK LPDALRGEKY AFVGLPLAEF LPGGGINIGV GRLCPVTLAA DS
TFLDLALTRR LPDALRCDSF AFARFPLAQL EEFFSERDYF GEKCLVDVAR DA
MQLNVSISEK LPDALRGERY AFVQLPYALL KEFSKL---F GELCPVELKD DE
GVMDVSTTTP MPDALRGESF AFVQLPMSTA LGFFEAGEFF GDACRVAMAD DP
VGAPPPSPRR LPDALVGQRW AFVTLPARDF ADMPDWPMDF GEGFPLGIGD DT
VSFAATTAQS LPDALIGQKW AFVSLEAGML EEMDEWAIDF GEAFPLNLSP DA
VQFETPKPER LPDALQGDRW AFVSLEAGSF AEMSEWEIDF SEAFPIQITP DT
VQYPPLNAIA LPDAVKGDKW TLVTLPVQDF IEMDQWDIAF GEAFPLDLDP QL
VRGQKSDPQR LPDALIGQKW VVASLTGADL AQMPEWEIGF GEAFPLEVAS DT
VRMEVPLPQR LPDALIGQQW AFVTLEAAAF ADMPEWEIGF GEAFPLGVKP ET
VKMESSPPQR LPDALTGQQW AFVNLEATAL ADMDEWEIAF GEAFPLGLSP ET
VRLDAPVPQR LPDALELENW AFVRLTAKDF LDMPEWEIGF GEGFPLQISD DT
VRLESPLPQR LPDALEGEQL QFVTLSAADF ADMPEWNIDF GEAFPLGISS EN
VRLDSPLPQR LPDALEGQQW VFVSLSAGEL AEMPEWDIGF GEAFPLQLSP EA
VRLERPLPQR LPDALEGKQW TFVSLGASDI TDMPEWEIAF GEAFPLGLSP EI
VRLEAPLPQR LPDALEWQQW GFVTLLGSEF ADMPDWEIDF GEGFPLQVSP ET
VQYPQTNPVN LPDALKKDKW ALVSLNGKDL EEMPEWDIGF REAFPLNISP DT
VQYPAENAVI LPDAIKGDKW AFVTLEAQAF QEMEDWDISF GEGFPLELAP ET
VQFEVQPPQP LPDALIGQKW MFVSLDASAF AEMHEWNIGF SEAFPLHLSP QT
VQYQPQIPQP LPDALEYDKW AFVTLEAGAF EEMNEWDIGF SEAFPLGLAP DT
VNYPPLNAVP LPDAVKADKW AFVTLELSSF NDLKDWDISF GENLPIGLDE NL
VQYPDLNAIP LPDAVRGDKW AFVSLEASAF AEMNEWEIGF KEAFPLNLSS ET
VAYPELNAIP LPDAVKADKW AFVTLEASAL EEMNEWEIGF GEGFPLGVTS EQ
VQYPPLNATP LPDAVKGDKW AFVSLEASAF EEMNEWDIAF GEAFPLGMTP DT
VQYPALNAVP LPDAIKGDKY AFVSLEAEAF AQMKEWDIAF GEAFPLGVTS EV
FQLDLGPPEA LPDALRGEQW AFVQLPLGTL LQMLKRAEIF GSGFTLDLPA DI
FQLDLGPPEA LPDALRGEQW AFVQLPLGTL LQMLRKGEIF GGTFSLDLPM DI
MGFEGGGPRP LPDALRGESW AFVALPLVGV REEAEQNRVF GDLLEITLED DT
```

```
MAFEPTAPKR LPDALRGESW AFVALPLVGV ---------F GALLDINLPD DT
LSMGVNVPKE VPPNYRGEKW AFVQLSFQAL QEEIKLGSNF GEVSLLP-SP DT
LLQQQSLPLD LPDALRGEEW AF--LALAAV LEEMEGGDVF GSVLDLDLSP GI
LTLDNPFPMQ LPENLFGEKW AFVQLPFSAV QEEVSSRLVF GASLDLEVDA NT

STRARALAVA LQGVDISSVS IDEDRGEIVY DVGVEDKWKV GRLDERLLRE AA
SPRAKALAVA LKGADLSSVS VDGDRNEVVY DIGVEDKWRI GKLDDRLVAE GT
SNRANSLANW INGLEVFSIS FDQEKSSIVL DCSLDTKFLF AKIDIKKIQD GT
SERAKSLSGW LDGVELCNVF CDLENKNLIL ECGLDIQFLF AKFSETKNSK NS
SRRALPLAAW FSGTELAYII ADEQQKEIYL ECGLDAAYLF ARIQPSLEAE AR
TRRSDALAMS LASTELAGVR ADAAQRQLVL DVALDESFLV AKLDDDQRVE AA
TARAEPLATW MTGLEVAYFK ADLKNRELAL ECGINTQYLV ARVQGDQRKE AQ
TNRAEALASW LAGTEVVALT ADLRKRVLVM ETDIDTQYLM AKLNESQRVE AA
TPRAKALASW LAGTEVAGLK ADLRKRELVM ETDIDNQYLM AKLNDDQRRE AA
ASRAAAIAGW MSGVELAAES SPRQAGHHPR V--------- ---------- --
SKRATPIAAW MSGLELSCIS ADMKKREILV QCDLDTSYLF GRVVPSIVDE AR
SKRAKGVAAW MSGAEVVAVK ADVATKEVVL LCGLSTSYLF ARLVGRIREE GK
SPRAVAMAGW MSGLELSELR VETKSPRLIL ETGAADSWIL SPLDSTLQTE AK
SPRAVPMAGW MSGLELGSLK LDTSTPRLLL ETGGSDRWIL ASLNAQVQTE AQ
SNRAKAIAAW MSGLELGFLK PELEPGQVVL ETGFNERWIL ANLDKTTRAE AQ
SNRAIPLAGW LSGLEIGSCY VEDQLSRLRL ETGLSDSWIL ADIDEQGQSE AR
SPRAVPLAGW MSGLEIAALK VDTNPARLIL ETGASDSWLL ANVNPQTLQM AQ
SPRAMPLAGW MSGLELANIK FDSETPQLLL ETGVTESWIL ASFDPQMIAE AK
SERALPLAGW MSGLELAFIR VETPVARLLL ETGASESWIL ANLNPQTVAE AQ
SSRSLPLAAW MSGLELGYLK FDQEGGRLLL ETGATESWIV ANINSQVINE AK
SNRALPIAAW MSGLELAWLR FDSKTGRLLL ETGATESWIL ANINPQMLLE AQ
SPRALPLAGW MSGLELAFLR VDQVGTRLIL ETGATESWIV ANINPQLLVE AK
SPRALPIAGW MSGLELAYLR LDSQGDRLVL ETGGTESWIL ANLTPQLLAE AK
SPRALPLAGW MSGLDLAWLR FDDQGGRLLL ETGATESWIL ANLNPQILAE AR
SSRALPLAGW MSGLELGYLR LDRKFPSICL ETGVSDSWIL VNLDKNTLSE AE
SPRAMPFAGW MSGIELSQIQ LQESLPRLVL QTGSSDCWIL ADINPETLKE AQ
SPRAIPMAAW MSGIEPALIK FYPPQARLLL ETGGSDSWFL VKQNGSSQTE AA
SSRATPLAGW MSGLELAFVR FDSESARLLL ETGASDSWIL ATLDSQTLAE AQ
SPRALPLAGW MSGLEMAYLK LEASRPLLRL ETGASDSWIL VNVNAETLNE AK
SPRATLLAAW LSGLEMGFLH LESPRPRICL NTGLSDSWVL VNLTPSTLTE AK
SDRALPLAAW MSGLELGFLK FEEPRPIVRL ETGTSDSWIL VNIDAPTLAE AQ
SSRALPLAGW MSGLELAFLK FEGSRPIVRL ETGASDSWIL ASLDPKMLAE AK
SSRALPLAGW MSGLEMGYLK LEESRPILRL ETGVSDSWIL LNVNPQTLAE AK
SRRALPLAAW TNGLEIAAVK ADVARSCLIL ETGVNQRWKY GSWNEDSIGE AE
SRRALPLAAW TNGLEIAAVK ADVQRSCLIL ETGVNQRWKY GSWNEDSIGE AE
```

```
TRRAAALAGW TKGLELGGIS VDFDMGTLLL DTGVSDSWQY ARFTKELMKE AR
TSRAAVSAA- ---------- ------HIVL ETGVNDSWSY AFFTPELTKE PK
SSRDLALAAW TNSLELASLS VDKKNSALVL LSGASRQWFY SYYSKQADEE AD
SSRATPLAAW TNALELASLE VDTQRSCLVL STGVADRWRY AFYSRQTDAE GE
SSRAKPLAAW MNGLEVCSIE ADTARACLIL SVGISTRYIY ATYTPVTTSE AE

LGGLHFLAIH QLPKDEIEGF WLMRQ
LGGLHFLAIH QL-------- -----
NSGFHFISVM SGLPNKIYGF WLLNE
SQGIHFVAVQ SYSKNEIAGI WTLKS
ARGLHFLAIQ EKPDEDVCGF WLLRD
LGGLHFVVVQ SPEDGEPAGF WLLRE
LGGFHFVAVQ SNPDDDVAGF WLLKE
LKGLHFVSVQ ENEDSDPTGF WLLRE
LNGLHFISVQ KDEDDDPAGF WLLRE
---------- ---------- -----
LNGLHFVAVQ KDEEDEVDGF WLLQE
SGGIHFLAVM VEEGDEVEGF WLLRE
ANQVHFLALQ ENPAEAFAGF WLMQG
ANGVHFLAIQ AAPDETFAGF WLLQE
AQGVHFLAIQ TDPNESFAGF WLLQE
VQQIHFLAIQ SSPEDSFAGL WLLKD
ANQVHFLAVQ SSPEEVFAGF WLLQE
ANGVHFLAVQ ANPEEAFAGF WLLQE
ANGVHFIAVQ SNPQESFAGF WLLQE
ANGVHFIGVQ ANPQESFAGF WLLQE
ANGVHFIGVQ SDPTESFAGF WLLRE
ANGVHFIGVQ SSPQESFAGF WLLQE
ADGVHFIGVQ SDPQQSFAGF WLLKE
ANGVHFIGVQ SDPQQSFAGF WLLCE
ANGVHFLAIQ SSPEQSFEAF WLLLE
AKGVHFLAIQ TDPSESFAGF WLLA-
AKGVHFLAIQ SSPQEDFAGF WLLQE
AEGVHFLAIQ STPTESFAGF WLLQE
ANNLHFLAVQ SNPEESFAGF WLLQE
AQGVHFLAIQ SSTEESFAGF WLLLA
GQQVHFLAIQ SSPDESFAGF WLLKE
AQQVHFLAIQ SNPEQSFAGF WLLKE
ANNVHFLAVQ SSPEESFSGF WLLKE
VKGVHFLAVQ PDPDEELNGL WLLQD
```

```
VKGLHFLAVQ PDPDEELNGL WLLQD
VNGLHFLAIQ TDEDETTDGF WILQD
CGGVT----- -------DGF WILRD
AGGLHFLAIQ PSLENSCSGL WILYD
CGGLHFLAVQ SSLDELCTGF WLLID
CGGLHFLAIQ DDLNDDCVGF WLLLD
```

Alignment: PGK

```
108Aureococcus_anophagefferens  RGKLGLEGLA SKDLGSSRVL IRLDLNVPLD DGITDDTRLR AVVPTMNFLK
156Phytophthora_capsici_Locald  TSKLSLELLA QRSLKGARVL VRADLNVPLN ATITDDTRIR AVLPTLKLLQ
162Schizochytrium_limacinum_Lo  TSKLSLEQLA QRSLKGARVL VRADLNVPLN QTITDDTRIR AVLPTLKLLQ
18Phaeodactylum_tricornutum_CC  EAKASIEDLS DAELKGKKVL VRCDVNVPLD GKITDDTRIR SSIPTIEYLK
22Thalassiosira_pseudonana_CCM  DAKKSIEDLS EADLKGKKVL VRCDVNVPLD GKITDDTRIR SSIPTIEYLK
24Thalassiosira_pseudonana_CCM  ATKCCISDLP AADLKGKKVL VRCDLNVPLD GKITDDTRIR SSIPTIEYLT
02Galdieria_sulphuraria         SKKKSLKDLS AADLKGKRVL VRCDLNVPLD GKITDDTRIK ASLPTIQYLI
49Cyanidioschyzon_merolae_Euka  MRVRSIRDLQ DAEIRGKRVF VRSDLNVPLD GKITDDTRIR ASVPTIKYLA
13Physcomitrella_patens_subsp_  EKKASIKDVP ESEFQGKVVF VRADLNVPLN DAITDDTRIR ASLPTIQHLT
12Ostreococcus_lucimarinus_CCE  AKKKSVGDLA ESDLKGQTVF VRCDLNVPMD ADITDDTRIR AAIPTLEYLA
14Micromonas_pusilla_CCMP1545_  AAKKSVGDLK EADLKGKTVF IRCDLNVPLD GAITDDTRIR AAIPTLEYLV
01Cyanophora_paradoxa           LAKKSVKNLT EADLKGKRVF IRCDLNVPLD KEITDDTRIR AAVPTIQYLA
10Volvox_carteri_f__nagariensi  AVKKSVGDLG KADLEGKRVF VRADLNVPLD KKITDDTRIR AAVPTLKYLL
11Chlamydomonas_reinhardtii_Eu  AVKKSVGDLH KADLEGKRVF VRADLNVPLD KAITDDTRIR AAVPTLKYLL
08Ectocarpus_siliculosus        VKKLSVGDLK DEDLKGKKVL VRCDLNVPLS DKIGDDTRIR ASIPTIEHLL
09QUERY_jgi_Bigna1_92730_estEx  EKKMSVGDLK KEELDGKVAL VRCDLNVPLD GKITDDTRIR ASIPTIKHLV
99Lth_pgk_92730                 ---------- ---------- ---------- ---------- ----------
07Guillardia_theta_CCMP2712     -KKKSVGDLK GADLKGKKVL IRCDLNVPLD GKITDDTRIR ASVQTLKYLL
05Ectocarpus_siliculosus        KPKKSVGDLT SEDLKGKRVL VRCDLNVPLD GSITDDTRIR ASIPTVKFLV
03Isochrysis_galbana            --KKSVGDLT KADLEGKRVL VRCDLNVPLD GKITDDTRIR ASIPTIKYLI
157Pyropia_yezoensis_Localdb__  TAKKSIKDLT AADLAGKRVL VRCDLNVPLD GKITDDTRIR ASIPTIEYLT
06Pyropia_yezoensis             TAKKSIKDLT AADLAGKRVL VRCDLNVPLD GKITDDTRIR ASIPTIEYLT
04Chondrus_crispus              --KKSIKDLS PADIAGKRVL VRCDLNVPLD GKITDDTRIR ASVPTIKFLM
30Synechococcus_sp__JA-3-3Ab_B  MAKQTLGSLL SSDLKGKRVL VRADFNVPLD PQITDDTRIR ASLPTIQALA
34Synechococcus_elongatus_PCC_  MSKRTLASLT AADLEGKRVL VRVDFNVPLD GNITDDTRIR AALPTIRYLS
23Synechococcus_sp__PCC_7335_B  MAKKALADLS ASDVSGKRVL VRADFNVPLD DAITDDTRIR AALPTIQDLT
26Thermosynechococcus_elongatu  MSKKSVAQLS AADLEGKRVL VRVDFNVPVD EKITDETRIR AALPTIQDLI
21Microcystis_sp__T1-4_Bacteri  MPKKTVANLT EADLAGKRVL VRVDFNVPMD KAISDDTRIR AALPTIEDLI
17Microcoleus_chthonoplastes_P  MSKKTVANLN ESDLAGKRVL VRADLNVPLD ENITDDTRIR AALPTIQDLI
37Cyanothece_sp__PCC_7425_Bact  MSKKTVANLS AADLAGKRVL VRVDFNVPLD DQITDDTRIR AALPTIQDLT
25Microcoleus_vaginatus_FGP-2_  MTKKTVANLS ASDLSGKRVL VRADFNVPLD N-ITDDTRIR AALPTIQDLA
27Raphidiopsis_brookii_D9_Bact  MSKKSLASLS AADISDKRAL VRVDFNVPVD DQITDDTRIR AALPTIQDLI
16Fischerella_sp__JSC-11_Bacte  MSKKTLANLS ATDLSGKRAF VRVDFNVPVD DAITDDTRIR AALPTIQDLV

                                51
108Aureococcus_anophagefferens  EQGAQVVVAT HIGRPKGPDA KFSVAPIVGR FSELLDTDVV KVDDCVGDDV
156Phytophthora_capsici_Locald  GAGARTVLCS HLGRPGGAKD ELRLTPVAVR LGELLGSPVK KLDDCIGSQV
162Schizochytrium_limacinum_Lo  GAGARTVLCS HLGRPGGAKD ELRLTPVAAR LGELLGSPVK KLDDCIGPQV
```

```
18Phaeodactylum_tricornutum_CC  NKGAIVSVCS HLGRPKDPED KFSLGPCAER MGELLGQTVT LAPDCIGEEV
22Thalassiosira_pseudonana_CCM  GKGAIVTVCS HLGRPKDPED KFSLGPCAER MGELLGQEVK LAPDCIGEEV
24Thalassiosira_pseudonana_CCM  KAGAIVTVCS HLGRPKDPED KFSLAPCATR MGELLGQEVK LAPDCIGDEV
02Galdieria_sulphuraria         NHGAKVILSS HLGRPKGFED RYSLAPVAER LGQLLGKSVR MAPDCIGDQV
49Cyanidioschyzon_merolae_Euka  DKGAKVIVVS HLGRPKGFED KYSLAPVAER LSELLGKKVR MAKDCIGDAV
13Physcomitrella_patens_subsp_  KAGAKVVLAS HLGRPKKPED KFSLKPVAGR LTELLGQTVE LAPDCIGAEV
12Ostreococcus_lucimarinus_CCE  KNGAKVLVTS HLGRPKNPED KFRLTPVGAR LSEKLSCPVT KVDDCVGDEV
14Micromonas_pusilla_CCMP1545_  KNGAKVLVTS HLGRPKDPED KFRLTPVGDR LAELLDCSVT KIDDCVGDVV
01Cyanophora_paradoxa           SKGAKVLVTS HLGRPKSPED KFRLTP-AAR LSELLGKPVK KTNDCIGAEV
10Volvox_carteri_f__nagariensi  DNGAKVLLTS HLGRPKGPED KYRLTPVVAR LSELLGKEVK KVDDCIGPSV
11Chlamydomonas_reinhardtii_Eu  DNGAKVLLTS HLGRPKGPED KYRLTPVVAR LSELLGKPVT KVDDCIGPEV
08Ectocarpus_siliculosus        SKGARVALSS HLGRPKDPED KFSLGPVAER LTKLLGKDCK MAPDCVGDEV
09QUERY_jgi_Bigna1_92730_estEx  ESGAKVLLTS HLGRPKDPED KFSLAPVAAR LSELLGQDVT FVKDCIGEPV
99Lth_pgk_92730                 ---------- ---------- ---------- ---------- ----------
07Guillardia_theta_CCMP2712     DNGARVAVSS HLGRPKDPED KFSLAPCATR LGELLGKDVK MAKDCIGDDV
05Ectocarpus_siliculosus        DKGAKVLLSS HLGRPKDPED KFSLAPVSER LTKLLGKKVT MAPDCIGPEV
03Isochrysis_galbana            DNGAKVLLSS HLGRPKGPED KFSLGPVAPR LSELLGKEVT MAPDCIGDAV
157Pyropia_yezoensis_Localdb__  AAGAKVLLSS HLGRPKSPED KFSLAPVATR LSELLKKDVK MAPDCIGDGV
06Pyropia_yezoensis             AAGAKVLLSS HLGRPKSPED KFSLAPVATR LSELLKKDVK MAPDCIGDGV
04Chondrus_crispus              EAGAKVLLSS HLGRPKDPED KFSLSPVAAR LTQLLGKDVT MAPDCIGDGV
30Synechococcus_sp__JA-3-3Ab_B  QAGAKVILTS HLGRPKAARE GNSLAPVAVR LAQLLGQPVA FAPDCIGPEA
34Synechococcus_elongatus_PCC_  ESGAKVILVS HFGRPKGPVE SMRLTPVAER LSELLGRPVV KTTDAVGAGA
23Synechococcus_sp__PCC_7335_B  SKGAKVILAS HFGRPKGVVE SMRLTPVAAR LSEKLGQDVI KCDDCIGDEV
26Thermosynechococcus_elongatu  SKGAKVILVS HFGRPKGVDE KLRLTPVAQR LSELLHKPVA KLDDCIGDAV
21Microcystis_sp__T1-4_Bacteri  KKGAKVILCS HMGRPDGVKE NLRLTPVAKR LSELLGQEVV MCPDCIGEGV
17Microcoleus_chthonoplastes_P  KKNAKVVMCS HLGRPKGVKD SLRLTPVANR LSELLGQTVV KCDDCIGDAV
37Cyanothece_sp__PCC_7425_Bact  SKGAKVILSS HFGRPKGVKD KFRLTPVAAR LSELLGKPVP KPNDCIGEEV
25Microcoleus_vaginatus_FGP-2_  SKGAKVILCS HFGRPKGVTE KLRLTPVAKR LSELLGKEVK KTDDCIGDEV
27Raphidiopsis_brookii_D9_Bact  SKGAKVILAS HFGRPKGVDE KFRLTPVAKR LSELLGQEVI KTNDCIGDDV
16Fischerella_sp__JSC-11_Bacte  QKGAKVILAS HFGRPKGVDE KLRLTPVAKR LSELLGQEVV KTDDCIGDEV

                               101
108Aureococcus_anophagefferens  AQACGAMPAG GVVLLENVRF HTGETKNFDD FAAALAGSAT AYVNDAFGTA
156Phytophthora_capsici_Locald  DADIDELKDG EIVLLENVRF YKQETKNDPE FAKKLAHGAD IYVNDAFGTA
162Schizochytrium_limacinum_Lo  DAGIDALQDG EIVLLENVRF YKQETENDPE FAKQLAHSAD IYVNDAFGTA
18Phaeodactylum_tricornutum_CC  AKIVNDAKEG DVIMLENTRF YKEETKNDPE FVEKLAAPFD LYVNDAFGTA
22Thalassiosira_pseudonana_CCM  STLVNAAKEG DVIMLENTRF YKEEEKNEAG FVEKLAAPFD LFVNDAFGTA
24Thalassiosira_pseudonana_CCM  ATMVAEAKEG DVVMLENTRF YKEETKNDPE FVEKLAKPFD MYVNDAFGSA
02Galdieria_sulphuraria         NNMVLAMNDG DIILLENVRF YTEETDNDPD FSRKLSMNMD LYVNDAFGSA
49Cyanidioschyzon_merolae_Euka  ATEVAAMSPG DVVLLENVRF YPEEEKNDPD FARKLAANAD LYVNDAFGTA
```

```
13Physcomitrella_patens_subsp_  ESKIAALKNG EVLLLENVRF YKEEEKNDSE FSQKLAKGVD IFVNDAFGTA
12Ostreococcus_lucimarinus_CCE  AKAVASMPNG SVCLLENTRF YKEEEKNDSD FAKKLAGSAT IFVNDAFGTA
14Micromonas_pusilla_CCMP1545_  KDAIAGMENG SVCLLENVRF YKDEEKNGAD FAKQLAEHAD LYVNDAFGTA
01Cyanophora_paradoxa           ESAVAAMSEG DVILLENVRF YPEEEKNDAE FAKKLAANAD LYVNDAFGTA
10Volvox_carteri_f__nagariensi  EQAVASLKSG ELLLLENVRF YKEEEKNDPE FAKKLASNAD LYVNDAFGTA
11Chlamydomonas_reinhardtii_Eu  EKAVGAMKNG ELLLLENVRF YKEEEKNEPE FAKKLAANAD LYVNDAFGTA
08Ectocarpus_siliculosus        AKIADGLGDG EVMLLENVRF YPAETKNDKD FAEKLAAPFD LFVNDAFGTA
09QUERY_jgi_Bigna1_92730_estEx  KEAVATMTPG SVALLENVRF YKEETKNEPE FAKKLAANAD LFVNDAFGTA
99Lth_pgk_92730                 ---------- ---------- ---------- ---------- ----------
07Guillardia_theta_CCMP2712     TKLVDSLKDG EICLLENTRF YKQEEKNDKE FSKKLAAPFD IYVNDAFGTA
05Ectocarpus_siliculosus        ASLVDDMKNG GVLLLENVRF YKEETKNDSD FAKKLAANAD LFVNDAFGTA
03Isochrysis_galbana            ADCVTKMANG DVLLLENTRF YKEEEKNDPE FAEKLAANAD MFVNDAFGTA
157Pyropia_yezoensis_Localdb__  AEIVSGMSNG DVTLLENVRF YKEETANDTE FAKKLAANAD MFVNDAFGTA
06Pyropia_yezoensis             AEIVSGMSNG DVTLLENVRF YKEETANDTE FAKKLAANAD MFVNDAFGTA
04Chondrus_crispus              TKIVSGMANG DVTLLENVRF YKEETKNDPD FAKKLAANAD FFVNDAFGTA
30Synechococcus_sp__JA-3-3Ab_B  EAVVSSLENG QVALLENVRF HPEEEANDPE FARKLASLAD LFVNDAFGSA
34Synechococcus_elongatus_PCC_  EAQVAATSNG QVVLLENVRF HAEEEANDAE FAKALASLAD IYVNDAFGAA
23Synechococcus_sp__PCC_7335_B  AAKIAEMKDG DVALLENVRF YDGETNNEPE FAKQLAANAD LYVNDAFGTA
26Thermosynechococcus_elongatu  IAHTQAMANG DVCLLENVRF HPGEEKNDPE FAKQLAACAE VYVNDAFGTA
21Microcystis_sp__T1-4_Bacteri  TAAISQMSNG QVALLENLRF HGEEEANDPD FAKKLAANAD LYVNDAFGTA
17Microcoleus_chthonoplastes_P  KSQIDSLDNG QVALLENLRF HPGEEKNDPE FTKKLAALAD VYVNDAFGTA
37Cyanothece_sp__PCC_7425_Bact  KAQVAAMQNG DVLLLENVRF HPGEEANTPE FAQELASVAD LYVNDAFGTA
25Microcoleus_vaginatus_FGP-2_  AATVAGMQDG DVLLLENVRF YPEEEANDPE FAKKLASVAD LYVNDAFGTA
27Raphidiopsis_brookii_D9_Bact  TARVAALEQG QVLLLENVRF HKEEEKNDPE FARQLAANAD FYVNDAFGTA
16Fischerella_sp__JSC-11_Bacte  AAKVEALQNG QVLLLENVRF YKEEEKNDPE FAKKLAANAD LYVNDAFGTA

                               151
108Aureococcus_anophagefferens  HRAHASTEGV VKFIGPAAGG YLMEKELVYI KGAIDDPKRP LTAILGGAKV
156Phytophthora_capsici_Locald  HRAHASTEGV TNYIKTNVAG FLIEKELKYL SGAVDAPVRP LGAIIGGAKV
162Schizochytrium_limacinum_Lo  HRAHASTEGV TNYVKTNVAG FLIEKELKYL SGAVDAPVRP LGAVIGGAKV
18Phaeodactylum_tricornutum_CC  HRAHASTEGV TKFLKPSVGG FLLAKELEYL DGAISNGKKP MAAIVGGSKV
22Thalassiosira_pseudonana_CCM  HRAHASTEGV TKFLQPSVSG FLLAKELEYL DGAIENGKKP MAAIVGGSKV
24Thalassiosira_pseudonana_CCM  HRAHASTEGV TKYLHPSVSG FLLAKELKYL AGAIDNGEKP MAAIVGGSKV
02Galdieria_sulphuraria         HRAHASTEGV TQFIRPAVAG FLLEKELEYL QNAIQNPERP FAAIIGGSKV
49Cyanidioschyzon_merolae_Euka  HRAHASTEGV TAFLKPAVAG FLLEKELDYL SNAIESPKRP FAAIVGGSKV
13Physcomitrella_patens_subsp_  HRAHSSTAGI AEYVGKTVAG FLLEKELAYL AGAVKAPARP FVAIVGGSKV
12Ostreococcus_lucimarinus_CCE  HRAHASTEGV TKYCKTNVAG FLLQKELDYL DGAVSNPERP FCAIVGGSKV
14Micromonas_pusilla_CCMP1545_  HRAHASTAGV TEFLSPSVAG FLLQKELDYL DGAVSNPERP FCAIVGGSKV
01Cyanophora_paradoxa           HRAHGSTAGV TAYLKPSVAG FLLEKELEYL AGAVDEPVRP LVAIVGGSKV
10Volvox_carteri_f__nagariensi  HRAHASTEGV TKFLKPSVAG FLLQKELDYL DGAVSAPKRP FVAIVGGSKV
```

```
11Chlamydomonas_reinhardtii_Eu   HRAHASTEGV TKFLKPSVAG FLLQKELDYL DGAVSNPKRP FVAIVGGSKV
08Ectocarpus_siliculosus         HRAHCSTEGV TKFLSPSVAG FLLQKELDYL AGAVQEPKRP FAAIVGGSKV
09QUERY_jgi_Bigna1_92730_estEx   HRAHGSTEGV TAHLKPSVAG FLLQKELDYL QGAVAEPARP FAAIVGGSKV
99Lth_pgk_92730                  ---------- ---------- ---------- ---------- --AIVGGSKV
07Guillardia_theta_CCMP2712      HRAHSSTAGV TEFIPTSVAG FLLQKELDYL DGAVANPKRP FAAIVGGSKV
05Ectocarpus_siliculosus         HRAHGSTEGV TKYLSPSVAG FLLQKELDYL QGAVAEPKRP FAAVVGGSKV
03Isochrysis_galbana             HRAHGSTEGV TKFLSPSVAG FLLQKELDYL DGAVSDPKRP FAAIVGGSKV
157Pyropia_yezoensis_Localdb__   HRAHGSTAGV TEFLRPSVAG LLLEKELAYL AGAVSNPKRP FAAIVGGSKV
06Pyropia_yezoensis              HRAHGSTAGV TEFLRPSVAG LLLEKELAYL AGAVSNPKRP FAAIVGGSKV
04Chondrus_crispus               HRAHGSTAGV TAHLKPSVAG LLLEKELAYL AGTVSDPARP FAAIVGGSKV
30Synechococcus_sp__JA-3-3Ab_B   HRAHASTAGV TAYLQPAVAG YLVEKELQFL SGAIENPQRP LAAIIGGSKV
34Synechococcus_elongatus_PCC_   HRAHAPTAGV TEYLSPCVAG YLLEKELQYL QAAIDNPQRP LAAIVGGSKV
23Synechococcus_sp__PCC_7335_B   HRAHASTAGV TEYLSPAVAG SLIEKELKFL QETVDNPQRP LAAIVGGSKV
26Thermosynechococcus_elongatu   HRAHASTAGV TQYLSPCVAG FLMEKELEYL QNAIEHPRRP LAAIVGGSKV
21Microcystis_sp__T1-4_Bacteri   HRAHASTEGV THYLSPSVAG YLIEKELNYL QAAIETPQRP LAAIIGGSKV
17Microcoleus_chthonoplastes_P   HRAHASTEGV THHLQPSVAG YLIEKELQYL QNAIENPQNP LAAIIGGSKV
37Cyanothece_sp__PCC_7425_Bact   HRAHASTEGV TRYLRPSVAG YLIEKELQYL QSAIENPQRP LAAIVGGSKV
25Microcoleus_vaginatus_FGP-2_   HRAHASTEGV TKYLSPSVAG FLMEKELQYL GSAIDNPQRP LAAIIGGSKV
27Raphidiopsis_brookii_D9_Bact   HRAHASTEGV TKYLSPSVAG YLVEKELQYL QAAIEFPQRP LAAIIGGSKV
16Fischerella_sp__JSC-11_Bacte   HRAHASTEGV TQFLSPCVAG YLVEKELQYL QRAIESPQRP LAAIIGGSKV

                                201
108Aureococcus_anophagefferens   STKIPVIESM LDKCDNVLVG GGMIFTFYKA MGHSVGASLI EEDMVPMAKD
156Phytophthora_capsici_Locald   STKIPVLKSL LQKCDKIMIG GGMIFTFYKA QGLDVGKSII EEDKVELAKD
162Schizochytrium_limacinum_Lo   STKIPVLKSL LQKCDKIMIG GGMIFTFYKA QGLDVGKSII EEDKVELAKE
18Phaeodactylum_tricornutum_CC   SSKITVLEAL LDKCDKVIIG GGMVFTFLKA KGFNVGTSLV EDDFVDTAKE
22Thalassiosira_pseudonana_CCM   SSKITVLNAL LDKCDKIIIG GGMVFTFLKA KGLNVGTSLV EDDYVETAKE
24Thalassiosira_pseudonana_CCM   SSKITVLNAL LDKCDKIIIG GGMVFTFLKA KDLSVGTSLV EDDYVETAKE
02Galdieria_sulphuraria          SSKIGVLKSL IEKCDVIIIG GGMVFTFLKA RGLSVGSSLV EEDKLYLAKE
49Cyanidioschyzon_merolae_Euka   STKIAVLEKL LQKCDTIIIG GGMVFTFLRA RGLKTGSSLV EEDKIELARV
13Physcomitrella_patens_subsp_   SSKITVIESL MNVCDKVILG GGMIFTFFKA DGKDVGSSLV EDDKIDLAKD
12Ostreococcus_lucimarinus_CCE   SSKIGVIESL LQKTDKIILG GGMIFTFYKA LGKSVGASLV EDDKIDLAKE
14Micromonas_pusilla_CCMP1545_   SSKIGVIESL LEKTDKIILG GGMIFTFYKA LGKSVGSSLV EDDKIDLAKE
01Cyanophora_paradoxa            SSKIGVIESL LNKCDKLII- GGMVFTFYKA RGLPTGSSLV EEDKVELAKK
10Volvox_carteri_f__nagariensi   SSKITVIEKL MEKCDKIIIG GGMIFTFYKA RGLKVGSSLV EEDKLELAKN
11Chlamydomonas_reinhardtii_Eu   SSKITVIEAL MEKCDKIIIG GGMIFTFYKA RGLKVGSSLV EDDKIELAKK
08Ectocarpus_siliculosus         SSKIGVIESL LAKCDKLIIG GGMVFTFLKA RGLGVGSSLV EEDKLELAKE
09QUERY_jgi_Bigna1_92730_estEx   SSKIGVIESL LEKVDKLVIG GGMVFTFLKA RGLEVGSSLV EEDKLELAKD
99Lth_pgk_92730                  SSKIGVIESL LEKCDKLIIG GGMVFTFLKA RGLEVGTSLV EEDKLELAKN
07Guillardia_theta_CCMP2712      SSKIGVIESL LGKCDKLVIG GGMVFTFLKA RGLNVGSSLV EEDKLELAKS
```

```
05Ectocarpus_siliculosus         SSKIGVIDSM LNKVDMLVIG GGMVFTFLKA RGLSVGSSLV EDDKLDLARE
03Isochrysis_galbana             SSKIGVIESL MEKVDKIVIG GGMVFTFLKA RGLETGSSLV EEDQLELAKK
157Pyropia_yezoensis_Localdb__   SSKIGVIESM LDKVDKLVIV GGMVFTFLKA RGLSTGSSLV EDDKIELAKE
06Pyropia_yezoensis              SSKIGVIESM LDKVDKLVIV GGMVFTFLKA RGLSTGSSLV EDDKIELAKE
04Chondrus_crispus               SSKIGVIDSL LQKVDKLIIG GGMVFTFLKA KGLNVGSSLV EDDKLELARE
30Synechococcus_sp__JA-3-3Ab_B   STKIGVIERL LEKVDKLLLG GGMIFTFYQA QGIPTGKSLV ETDKLDLARS
34Synechococcus_elongatus_PCC_   SSKIGVIETL LDKCDKLLIG GGMIFTFYKA QGLSVGGSLV EEDKLDLARS
23Synechococcus_sp__PCC_7335_B   SSKIGVIETL LDKVDKLFIG GGMIFTFFKA RGIGVGSSLV EEDKLDLAKK
26Thermosynechococcus_elongatu   SSKIGVIEAL LEKVDKLLIG GGMIFTFYKA RGLNVGKSLV EEDKLELAKH
21Microcystis_sp__T1-4_Bacteri   SSKIGVIETL LEKCDKLLIG GGMIFTFYKA RGLNVGGSLV EEDKLELAKS
17Microcoleus_chthonoplastes_P   SSKIGVIETL LDKVDKLLIG GGMIFTFYKA RGLSVGKSLV EEDKLELAKS
37Cyanothece_sp__PCC_7425_Bact   SSKIGVIETL LEKVDKLLLG GGMIFTFYKA RGLNVGKSLV EEDKLELART
25Microcoleus_vaginatus_FGP-2_   SSKIGVIEKL LEKCDKLLLG GGMVFTFYKA RGLSVGKSLV EDDKLELAKS
27Raphidiopsis_brookii_D9_Bact   SSKIGVIETL LEKCDKLIIG GGMIFTFYKA RGLNVGKSLV EQDKLELASS
16Fischerella_sp__JSC-11_Bacte   SSKIGVIETL LEKCDKLIIG GGMIFTFYKA RGLSVGKSLV EEDKLELAKS

                                 251
108Aureococcus_anophagefferens   LIAKAKAKGV NFLLPTDVVV ADKFAADANY KVVPVDGIPD GWLGLDVGPA
156Phytophthora_capsici_Locald   ILKEAKQRGV QIVLPTDVVI ADKFEADAQS KVVSANAIPA DWLGLDIGPD
162Schizochytrium_limacinum_Lo   ILEEAKQRGV QIVLPTDVVV ADKFEADAQS KVVPANAIPA DWLGLDIGPD
18Phaeodactylum_tricornutum_CC   VLAKAEKLGK EILLPIDIVI ADKFDAEAET KVVSAEEIPD GWMGLDNGPE
22Thalassiosira_pseudonana_CCM   VLAKAEKLGK EILLPSDIVI ADKFAADAET KVVSSDAIPD GWMGLDNGPA
24Thalassiosira_pseudonana_CCM   VLAKAEKLGK KILLPCDIVV ADKFAADAET KIVATDEIPD GWMGLDNGPA
02Galdieria_sulphuraria          IEQMAAQKKV ELIFPLDLLV ADKFDNHANV KTVSFSQIEE GWMGLDIGPA
49Cyanidioschyzon_merolae_Euka   IEEEARKRGI ELLLPLDVVV ADKFANDANT KVVPVEEIPD GWMGLDNGPE
13Physcomitrella_patens_subsp_   LVALAKKKGV ELILPVDVTA ADKFSPEANT QVCSSSNIPA GWMGLDIGPK
12Ostreococcus_lucimarinus_CCE   LMAKAEAKGV KILLPTDVVV ADKFAEDAKT QIVSVDNIPE GWMGLDIGPD
14Micromonas_pusilla_CCMP1545_   LMAKAEKKGV KILLPTDVVV ADAFAADAKT QTVSVDDIPE GWMGLDIGPD
01Cyanophora_paradoxa            LEEVAKAKGV EILLPTDVLL ADKFDPEANT QVVDADKIPD GWMGLDIGPK
10Volvox_carteri_f__nagariensi   LEAIAKAKGV QLLLPSDVVV ADKFDANANT QTVSVEAIPD GWMGLDIGPD
11Chlamydomonas_reinhardtii_Eu   LEEMAKAKGV QLLLPTDVVV ADKFDANANT QTVPITAIPD GWMGLDIGPD
08Ectocarpus_siliculosus         LEEKAKTAGV EIILPSDVVV ADAFAADAKT QVVKADAIPD GWMGLDNGPE
09QUERY_jgi_Bigna1_92730_estEx   LERIAKEKGV ELILPTDVVL ADAFAKDANT QVADATAIPE GWMGLDQGPK
99Lth_pgk_92730                  LERIAKEKGV ELILPSDVIL ADAFSADANT QVADASAIPE GWMGLDQGPK
07Guillardia_theta_CCMP2712      LEKKAAEKGV KFILPTDVVL ADKFAADANT KVASVSDIPD GWMGLDNGPE
05Ectocarpus_siliculosus         LEKTAASKGV EIILPTDVVL ADAFSADANT QVASVDAIPD GWMGLDNGPD
03Isochrysis_galbana             LEVIAKEKGI DFILPTDVIV ADKFAADAAT QIVSVDAIPE GWMGLDNGPE
157Pyropia_yezoensis_Localdb__   LEEIAAKKGV KIILPVDVVA ADKFAPDAKT QLVSVDAIPD DTMGLDQGPK
06Pyropia_yezoensis              LEEIAAKKGV KIILPVDVVA ADKFAPDAKT QLVSVDAIPD DTMGLDQGPK
04Chondrus_crispus               LVKTAEAKGV SIILPTDVVL ADKFAADANT QVTSVDSIPD GWMGLDQGED
```

```
30Synechococcus_sp__JA-3-3Ab_B  LMEKAKARGV ELLLPVDVVV ADRFDKDANA QTVSIHAIPE DWMGLDIGPE
34Synechococcus_elongatus_PCC_  LMAKAQEKGV QLLLPVDVVV ADKFAPDANA KTVAIDAIPD GWMGLDIGPE
23Synechococcus_sp__PCC_7335_B  LEAKAKEKGV EFLLPTDVIV ADKFAADANT QTVSVENIPD GWMGLDIGPD
26Thermosynechococcus_elongatu  LETKAQEKGV ELLLPTDVVV ADNFANDANS QVVSIEAIPD DWMGLDIGPA
21Microcystis_sp__T1-4_Bacteri  LEAKAKEKGV EFLLPTDVVL ADKFDKDAKS QIVKVENIPD GWMGLDIGPD
17Microcoleus_chthonoplastes_P  LEAKAKEKGV DLLLPTDVVV ADNFAADANS QTVSVESIPD GWMGLDIGPD
37Cyanothece_sp__PCC_7425_Bact  LEAKAKERGV ALLLPTDVVV ADAFAADANA QTVSVESIPD GWMGLDIGPD
25Microcoleus_vaginatus_FGP-2_  LEAKAKERGV TFLLPTDVVV ADKFAADANT QTVSVENIPE GWMGLDIGPD
27Raphidiopsis_brookii_D9_Bact  LEKKAAERGV TLLLPTDIVA ADKFAPDADA VTVSIENIPD DRMGLDIGPK
16Fischerella_sp__JSC-11_Bacte  LEAKAKEKGV TFLLPTDVVV ADNFAADANS QTVSIENIPD GWMGLDIGPD

                                301
108Aureococcus_anophagefferens  TQATFSATAA ASTTVVWNGP MGVFEFDAFS KGTFGVADAL AAN--ESAIT
156Phytophthora_capsici_Locald  SQAAFAKELK ECKTVVWNGP MGVFEFPEFA KGTFGVAKAL AESTETGVTT
162Schizochytrium_limacinum_Lo  SQATFAKELK ECKTVVWNGP MGVFEFPEFA KGTFGVAKAL AESTESGVTT
18Phaeodactylum_tricornutum_CC  TTAQQKEALS DCKTIIMNGP MGVFEFEKFA KGTFDLVNIL ADLSKKGAIT
22Thalassiosira_pseudonana_CCM  STAEQKEFLS DCQTVIMNGP MGVFEMKAFE KGTFGLVDIL ADLSKKGAIT
24Thalassiosira_pseudonana_CCM  STAEQKEFLS DCKTVIMNGP LGVFEMKAFE KGTMGIIDIL ADLTKKGAIT
02Galdieria_sulphuraria         TIEMIESALA KCKTILWNGP MGVFEMENFA RGTFQVARIL AKLTEQGAIT
49Cyanidioschyzon_merolae_Euka  TTRMIQEKLR TMKTVLWNGP LGVFEMPNFA KGTFAIAETL AEITQNGCIT
13Physcomitrella_patens_subsp_  AIDQFQDALK GAKTVLWNGP MGVFEFEKFA DGTTAVAKTL AGLTKEGAIT
12Ostreococcus_lucimarinus_CCE  SLKAFQGELN ECKSVIWNGP MGVFEMDAFA KGTFGIADTL ANLNG---IT
14Micromonas_pusilla_CCMP1545_  SVKSFQDELN ECKSVIWNGP MGVFEMDAFA KGTFAIADTL ANLDG---IT
01Cyanophora_paradoxa           SIESFSKALS DAKTVIWNGP MGVFEFDKFA KGTFAIADLL AELTPKGCKT
10Volvox_carteri_f__nagariensi  SIKTFQDALA DAKTVVWNGP MGVFEFPKFA VGTVAIANTL SELTPKGAIT
11Chlamydomonas_reinhardtii_Eu  SVKTFNDALA DAKTVVWNGP MGVFEFPKFA NGTVSIANTL AGLTPKGCIT
08Ectocarpus_siliculosus        ATKEIQAALK ECKTVVWNGP MGVFEYEAFA KGTFAIADTL AEITSEGCTT
09QUERY_jgi_Bigna1_92730_estEx  ATEEIIAALK DCKTVIWNGP MGVFEFEAFS KGTFGVADAL ADLTKGGATT
99Lth_pgk_92730                 ATEEIVKALG ECKTVIWNGP MGVFEFEKFA AGTMDVANAL AKLTKDGATT
07Guillardia_theta_CCMP2712     STKMIQKELS DCNTIIWNGP MGVFEFEKFA KGTNDVAKTL AECTSKGAIT
05Ectocarpus_siliculosus        STSLIQGKLA DCKTVIWNGP MGVFEYEAFA K--FGVGENL GAAPGKGWLT
03Isochrysis_galbana            STKMIQQKLS ECKTIIWNGP MGVFEFEAFA KGTFDVADTL AELTGKGCTT
157Pyropia_yezoensis_Localdb__  STELINAELA DCKTVLWNGP AGVFEFDAFA TGTYAIANTL AEVTANGATT
06Pyropia_yezoensis             STELINAELA DCKTVLWNGP AGVFEFDAFA TGTYAIANTL AEVTANGATT
04Chondrus_crispus              STKLIQNELK ECKTVIWNGP MGVFEMEKFA KGTFAIADTL ADLTESGCIT
30Synechococcus_sp__JA-3-3Ab_B  SVKAFQAALQ GCKTVVWNGP MGVFEFDRFA AGTEAIARTL ADLTQAGAIT
34Synechococcus_elongatus_PCC_  SVKQFEGALA DCRSVIWNGP MGVFEFDQFA VGTEAIARSL AGLTRKGATT
23Synechococcus_sp__PCC_7335_B  SVKTFQDALA DCKSVIWNGP MGVFEMEAFA KGTTGIATTL AGLTDKGCIT
26Thermosynechococcus_elongatu  SVKRFQEALQ DCKTVIWNGP MGVFEFDQFA KGTEAIARYL AELTSQGVCT
21Microcystis_sp__T1-4_Bacteri  SVKVFQKALS SCKSVLWNGP MGVFEFDKFA AGTDAIAHTL ADLTATGTTT
```

```
17Microcoleus_chthonoplastes_P  SVKFFQDALG DCKSVIWNGP MGVFEFDKFA AGTEAIARTL ADLTKKGTTT
37Cyanothece_sp__PCC_7425_Bact  AVKTFQEALS DCKTVIWNGP MGVFEFDKFA VGTEAIAHSL AGLTGKGAST
25Microcoleus_vaginatus_FGP-2_  SVKTFQDALA DCKTVIWNGP MGVFEMEKFA AGTEGIAHSL AGITKTGTTT
27Raphidiopsis_brookii_D9_Bact  SVKLFQDALA DCKTVIWNGP MGVFEFDKFA VGTEAIAQTL AEIGKTGATT
16Fischerella_sp__JSC-11_Bacte  SVKLFQEALA DCKTVVWNGP MGVFEFDKFA VGTEAIARTL ADLSKNGATT

                                351
108Aureococcus_anophagefferens  IIGGGDSVAA VEKAGLADKM SHISTGGGAS LELLEGKVLP GVAALDDA
156Phytophthora_capsici_Locald  IVGGGDSVAA VEQAGLGSKM SHISTGGGAS LELLEGKVLP GVAALNDA
162Schizochytrium_limacinum_Lo  IVGGGDSVAA VEQAGLGSKM SHISTGGGAS LELLEGKVLP GVAALNDA
18Phaeodactylum_tricornutum_CC  IIGGGDSVAA TEQSGRAGDM SHISTGGGAS LELLEGKVLP GVAALNDK
22Thalassiosira_pseudonana_CCM  IIGGGDSVAA TELSGRAGDM SHISTGGGAS LELLEGKVLP GVAALNDK
24Thalassiosira_pseudonana_CCM  IIGGGDSVAA TEQSGRAKDM SHISTGGGAS LELMEGKVLP GVAALDEK
02Galdieria_sulphuraria         IIGGGDSVAA VEKANLADKM SHISTGGGAS LELIEGRVLP GVAALDDA
49Cyanidioschyzon_merolae_Euka  IVGGGDSVAA MEQSGKADKV SHISTGGGAA LELIEGRVLP GVAALQRA
13Physcomitrella_patens_subsp_  IIGGGDSVAA VEKAGLADQM SHVSTGGGAS LELLEGKVLP GVAALDNA
12Ostreococcus_lucimarinus_CCE  IIGGGDSVAA VEKAGLADKM SHISTGGGAS LELLEGKVLP GVAALDEA
14Micromonas_pusilla_CCMP1545_  IIGGGDSVAA VEKAGLADKM SHISTGGGAS LELLEGKVLP GVAALDEA
01Cyanophora_paradoxa           IIGGGDSVAA VEKAGLAEKM SHISTGGGAS LELLEGKVLP GVAALDDA
10Volvox_carteri_f__nagariensi  IIGGGDSVAA VEQAGVAEKM SHISTGGGAS LELLEGKVLP GVAALDEK
11Chlamydomonas_reinhardtii_Eu  IIGGGDSVAA VEQAGVAEKM SHISTGGGAS LELLEGKVLP GVAALDEK
08Ectocarpus_siliculosus        IVGGGDSVAA VEKANLADKM SHISTGGGAS LELLEGKVLP GVAALNDA
09QUERY_jgi_Bigna1_92730_estEx  IIGGGDSVAA VEQAGLADQM SHISTGGGAS LELLEGKVLP GVAALNAA
99Lth_pgk_92730                 IIGGGDSVAA VEKAKVADQM SHISTGGGAS LELLEGKVLP GVAALNAK
07Guillardia_theta_CCMP2712     IIGGGDSVAA VEKAGLADKM SHISTGGGAS LELLEGLVLP GVAALTDA
05Ectocarpus_siliculosus        IIGGGKSVGA VEKAGLADKM SHISTGGGAS LELLEGKVLP GVAALD--
03Isochrysis_galbana            IIGGGDSVAA VEKAGLAEKM SHISTGGGAS LELLEGKVLP GVAALQEA
157Pyropia_yezoensis_Localdb__  IIGGGDSVAA VEKAGLAEKM SHISTGGGAS LELLEGKVLP GVAALDDQ
06Pyropia_yezoensis             IIGGGDSVAA VEKAGLAEKM SHISTGGGAS LELLEGKVLP GVAALD--
04Chondrus_crispus              IIGGGDSVAA VEKAGLAEKM SHISTGGGAS LELLEGKVLP GVAALD--
30Synechococcus_sp__JA-3-3Ab_B  IIGGGDSVAA VEKVGLADKM THISTGGGAS LELLEGKELP GIAALSEA
34Synechococcus_elongatus_PCC_  IIGGGDSVAA VEKVGVASEM SHISTGGGAS LELLEGKVLP GVAALDDA
23Synechococcus_sp__PCC_7335_B  IIGGGDSVAA VEQAGLADKM SHISTGGGAS LELLEGKTLP GIAALDEA
26Thermosynechococcus_elongatu  IIGGGDSVAA VEKVGVADRM SHISTGGGAS LELLEGKQLP GIAALDDA
21Microcystis_sp__T1-4_Bacteri  IIGGGDSVAA VEKVGVAEKM SHISTGGGAS LELLEGKVLP GIAALDEA
17Microcoleus_chthonoplastes_P  IIGGGDSVAA VEKVGVADQM SHISTGGGAS LELLEGKVLP GIAALDDA
37Cyanothece_sp__PCC_7425_Bact  IIGGGDSVAA VEKVGVAEQM SHISTGGGAS LELLEGKELP GIVALDDA
25Microcoleus_vaginatus_FGP-2_  IIGGGDSVAA VEQLNLGEQM SHISTGGGAS LELLEGKDLP GVAALDEA
27Raphidiopsis_brookii_D9_Bact  IIGGGDSVAA VEKVGLADQM SHISTGGGAS LELLEGTVLP GIAALDEA
16Fischerella_sp__JSC-11_Bacte  IIGGGDSVAA VEKVGLADQM SHISTGGGAS LELLEGKVLP GIAALDEA
```

Alignment: PS2SAF

```
39Synechococcus_sp__JA-2-3Ba_2  MVPWQQVEVP TQNILLDIAF TTPSHGWLVG DKATLLESRD GGLHWQVREL
12Cyanophora_paradoxa_Eukaryot  TK-WEQIPLN TDEILLDIGF VQPQRGWLLG TRSTLFETTD KGKTWELRSL
21Synechococcus_elongatus_PCC_  ESPWQVIDLG TEKTILDIAF TNKQHGWLVG TDLALYETLD GEQSWSERAL
15Thermosynechococcus_elongatu  INPWEAIQLP TTATILDMSF IDRHHGWLVG VNATLMETRD GGQTWEPRTL
35Acaryochloris_sp__CCMEE_5410  -LSWETVALP TDSTVLDVSF A-PDHGWLVG TNSALLETFD GGKSWEQRPL
24Leptolyngbya_sp__PCC_7375_Ba  LNPWQMIALE TDATFSDVAF TDASHGWLVG SRTSLFETND GGETWGQRTL
34Leptolyngbya_sp__PCC_6406_Ba  LNPWEVISLE TEATFSDIAF TDPSHGWLVG SRTTLLETTD GGKTWEPRSL
32Dactylococcopsis_salina_PCC_  VNPWRVLNLP TEETMLDLAF TDPNHGWMVG NQATLLETTD GGETWERKEL
18Chamaesiphon_minutus_PCC_660  ISPWKPIQLP TESNIQDLTF TNPQHGWVVG SDAAILETND GGKTWENRKL
31Gloeocapsa_sp__PCC_73106_Bac  LNPWKMIQLP TEATFADVAF TDPKHGWLVG TKATLFETKD AGETWTPVVI
22Cyanobacterium_aponinum_PCC_  LNPWQLISLD AESTFADIDF TDSNHGWLVG TRATLFETKD GGDNWQQKII
28Microcystis_aeruginosa_DIANC  MSPWQILTLD TDSTFADIAF TDLQHGWLVG TKSTLFETSD GGDSWQQKVL
19Stanieria_cyanosphaera_PCC_7  LNPWQIKTLP TEAILSDIAF TNSNHGWLVG TQASLFETTD GGNTWQQKVL
17Pleurocapsa_sp__PCC_7327_Bac  VNPWSVIQLE TESTFADLAF TDPNHGWLVG SQAALFETTD GGKTWQEKKL
27Cyanothece_sp__PCC_7822_Bact  VNPWKLINLE TESTFADVAF TDPNHGWIVG TQATLFETTD GGDNWEERKL
13Geitlerinema_sp__PCC_7407_Ba  LSPWRSVSLP EDITLLDIAF ANSQHGWLVG SRSTLLETQD GGTTWDAKVL
33Calothrix_sp__PCC_6303_Bacte  LNPWKVISVD ADTKLLDIAF ANQEHGFMVG SNATLLETKD AGETWEAIKL
29Rivularia_sp__PCC_7116_Bacte  VNPWEVISLP TDSKLFDIAF TDPNHGFIVG NKATLLETKD AGETWNPIVL
36Nodularia_spumigena_CCY9414_  VNPWAIISVP TDSKLLDIAF TNSQHGFLVG GDATLLETND GGDTWKPLTL
14Fischerella_sp__JSC-11_Bacte  VNPWEVISVD TDAKLLDIAF TNPQHGFLVG SNATLLETKD GGETWQPIKL
23Oscillatoriales_cyanobacteri  LNPWQVVTVP TESNLLDIAF VDSSHGWVVG ANASLFETTD NGETWQERTL
25Oscillatoria_nigro-viridis_P  INPWQVISLP TEANLQDIAF TNPQHGWVVG SEATLLETLD GGTSWKSIAL
38Oscillatoria_sp__PCC_6506_Ba  INPWKVVPLP TKANLQDVAF TNPQHGWIVG SDTTLLETKD GGENWQAIAL
20Crinalium_epipsammum_PCC_933  VSPWQVINVP TQATLADIAF TDSNHGWLVG SDSTLVETTD GGKTWQVRRV
30Synechocystis_sp__PCC_7509_B  VNPWQVLTVP SEANFQDITF TDSQHGWLVG SKSTILETHD GGESWQQVSL
16Gloeocapsa_sp__PCC_7428_Bact  TNPWKVITVP TESNLQDIAF TDT-HGWLVG SKAALLETTD GGETWQPRTL
26Chroococcidiopsis_thermalis_  VNPWEVVTLP TDVNLQDVGF TDTQHGWLVG NKSALFETKD GGKTWQQKSL
08Ostreococcus_lucimarinus_CCE  ESYWEQVELP LEPILLDIAF SDPKHGFLLG TRQTVLETKD GGKTWDVRDL
09Micromonas_pusilla_CCMP1545_  -KFWEQVDLP LEPILLDIAF EDPNHGFLLG TRQTILETFD GGKTWDFKAI
10Chlamydomonas_reinhardtii_Eu  ---------- ---------- -NPTKGFLLG TRQTLLETND GGKTWAPRSI
07Brachypodium_distachyon_Euka  ASEWQRVKLP IDPVLLDIAF VDPSHGFLLG TRQTILETKD GGNSWFPRSI
06Selaginella_moellendorffii_E  ASQWEKVPLP IDPVLLDLSF VEPNRGFLLG TRQTLLETKD GGRSWSPRSV
11Physcomitrella_patens_subsp_  LSSWEQVVLP VDPVLLDMAF VQPDRGFLLG TRQTLLETKD AGRSWSPRSI
48Cyanidioschyzon_merolae_Euka  GAAWKQVPLP TESVLFDIDF SDPNHGWLVG TRGLVLETRD GGETWEPRAF
67Ectocarpus_siliculosus_Local  TSAWEQIQLP VASVLYDIAF DHPDHGLVVG AQGTFLETFD GGYKWSVRTF
65Aureococcus_anophagefferens_  IKNWERVSLP VSTTLFDIAF -TDTHGYLVG AKGTFLETVD GGKNWKPRTF
03Phaeodactylum_tricornutum_CC  SQLWSKVPLP FEDTLFDIDF -SPTHGYLVG ARGAFAETND GGKTWEARSF
05Thalassiosira_pseudonana_CCM  SRQWRQVKVP FEDTVYDIDF -SPTHGYIVG ARGSFAETND GGATWEPRSF
```

```
96Pyropia_yezoensis_Localdb__L   PPSWEQVPLS AETTLFDMSF SDPNHGWLVG SRGTVLETND GGDSWKPRAF
02Hemiselmis_andersenii_Eukary   SKKWEQVEIP VDTVLFDLDF VDPQHGWLVG SKGTFLETTD AGKTWIPRTF
04Guillardia_theta_Eukaryota__   NTSWTKVDLP VDSVLFDIEF TECKHGWLVG SKGTFLETDD GGNTWVPRTF
68Emiliania_huxleyi_Localdb__L   QPTWAPVQLP LATILFDIEF DNPKNGFIVG NKGTFLQTTD GGSSWQAKSF
01QUERY_Bigel_p2saf_91760        ---------M QQLVLFDIEF -DDKNGYLVG SQGTFLRTND GGKTWSNADL
47Lotharella_amoeboformis_Euka   ---------- ---------- ---------- ---------- ----------

                                 51
39Synechococcus_sp__JA-2-3Ba_2   PLEPEAY-LA SISFAGAEGW VAGQPKILLH TLNEGSDWTS IRLNNQLPGE
12Cyanophora_paradoxa_Eukaryot   NLEDDKYRLN SISFSGKEGW VTGKPAILLH TTDGGSSWSR IPLSNQLPGD
21Synechococcus_elongatus_PCC_   DLDET-YRLN SISFKGDEGW VVGQPSLMLH TTDGGKNWLR IPLSEKLPGS
15Thermosynechococcus_elongatu   VLDHSDYRFN SVSFQGNEGW IVGEPPIMLH TTDGGQSWSQ IPLDPKLPGS
35Acaryochloris_sp__CCMEE_5410   ALGDLDYRFS SISFSGDEGW IVGEPSILLH TTDGGKSWSR VSLSTQLPGT
24Leptolyngbya_sp__PCC_7375_Ba   DLGDQRYTFT SVSFVGDEGW VSGLPSVLLH TTDGGKSWAN VPLSNQLPGS
34Leptolyngbya_sp__PCC_6406_Ba   NLGDQRYTFT SVSFADQEGW VAGQPSILLH TRDGGGSWEK VPLSEKLPGT
32Dactylococcopsis_salina_PCC_   ELENEKLDFL SVSFVGEEGW IAGEPSVLLH TKDGGEHWSR IPLSKKLPGE
18Chamaesiphon_minutus_PCC_660   ELDSSKYRFT SISFAGDEGW IVGEPAILLH TKDAGKSWER IALSSKLPGI
31Gloeocapsa_sp__PCC_73106_Bac   DFGEEKISFT GISFYQQEGW ITGDPLVLLH TTNGGKTWER VRLSAKLPGA
22Cyanobacterium_aponinum_PCC_   DLGEEKVNFT GVSFYDNEGW ITGMPSVLLH TNNGGETWER IPLSEKLPGS
28Microcystis_aeruginosa_DIANC   NLGDEKVSFS AVSFHDQEGW IVGKPSILLH SEDGGSSWSR IPLSEKLPGS
19Stanieria_cyanosphaera_PCC_7   NLEDEKVSFS AISFAGEEGW IVGKPSILLH TEDGGTTWSR IPLSSKLPGA
17Pleurocapsa_sp__PCC_7327_Bac   DLGEEKASFT AVSFNGQEGW ITGKPSILLH TEDGGQSWSR IPLSEKLPGA
27Cyanothece_sp__PCC_7822_Bact   DLGEEKVSFD GVSFNGDEGW ITGKPAILLH TEDGGQTWSR IPLSEKLPGA
13Geitlerinema_sp__PCC_7407_Ba   DLDEAKYSLT SVSFAGKEGW VVGQPSLLLH TTDEGKSWSQ VPLSEKLPGA
33Calothrix_sp__PCC_6303_Bacte   SLDSEKSRFN GVSFAGKEGW IVGEPALVLH STDAGKSWLQ IPLDEKLPGN
29Rivularia_sp__PCC_7116_Bacte   QVGDENYRFN AIDFHGNEGW IAAEPSILLH TSDEGKTWTS IPLSEKLPGN
36Nodularia_spumigena_CCY9414_   ELDDPKSRFD SVSFAGKEGW ILGEPSLLLH TTDEGSSWSR IALSEKLPGS
14Fischerella_sp__JSC-11_Bacte   QLDEDKYRFN SVSFKGQEGW IVGEPALLLH STDEGKSWLR IPLSEKLPGN
23Oscillatoriales_cyanobacteri   ELDQP-YRFT SVSFYEQEGW IVGQPSILLH TEDEGRSWSR IPLSEKLPGA
25Oscillatoria_nigro-viridis_P   DIDDPRSRFA SVSFSGSEGW IVGQPSVLLH TNDEGKSWTR IALSSQLPGS
38Oscillatoria_sp__PCC_6506_Ba   NLEDARSRFS SVSFSGSEGW IVGQPSVLLH TEDEGKSWTR IALSSQLPGS
20Crinalium_epipsammum_PCC_933   DVDSQNYRFT SVSFAGDEGW VTGEPAILLH TTDGGTSWSR IPLSNKLPGA
30Synechocystis_sp__PCC_7509_B   DLDEKNNLFT SVSFDGDEGW ITGEPSILLH TTDKGETWSR IPLSEKLPGK
16Gloeocapsa_sp__PCC_7428_Bact   DLGEQNYLFS SISFAGQEGW IVGEPALLLH TTDGGKSWEQ IPLSEKLPGN
26Chroococcidiopsis_thermalis_   DLGDKNFILS SVSFSGQEGW IVGEPSLLLH TNDGGETWSR ILLSEKLPGN
08Ostreococcus_lucimarinus_CCE   SDDDVNYRFN SVSFCGDEGW IIGKPAVLLH TTDGGANWER VGLSPRLPGA
09Micromonas_pusilla_CCMP1545_   TDEDVNYRFN SITFKGKEGW IVGKPAILLH TIDGGANWER IGLSPRLPGA
10Chlamydomonas_reinhardtii_Eu   DDEGFNYRFN SISFAGKEGW IVGKPAILLH TSDGGANWER IPLSSKLPGK
07Brachypodium_distachyon_Euka   PDEDFNYRFN SVSFMGKEGW IVGKPAILLH TKDAGESWER IPLSAQLPGD
06Selaginella_moellendorffii_E   AEEDFNYRFN SISFKGKEGW IIGKPAILLH TSNAGETWER IPLSSRLPGN
```

```
11Physcomitrella_patens_subsp_  PDEDFNYRFN SISFQGQEGW IIGKPAILLH TSNGGESWER IPLSIRLPGN
48Cyanidioschyzon_merolae_Euka  EEEELNYRFS NVSFSGDEAW VIGKPPVMLR STDGGKNWSR ILLSPKLPGE
67Ectocarpus_siliculosus_Local  GEEEINYRFQ KVSMYNDEIF IIGKPPILLH SKDAGKTWER VPLSPKLPGE
65Aureococcus_anophagefferens_  AEEEVGYRFE KVSFQDGEGW VVGKPPILLH TVDAGKSWER IPLSPKLPGE
03Phaeodactylum_tricornutum_CC  SEEEVTYRFQ VLSFKDGEGW VLGKPTLLLH TKDSGKTWER IPLSPKLPGE
05Thalassiosira_pseudonana_CCM  SEEEITYRFQ VVSFNNGEGW VLGKPTLLLH TKDGGKTWER IPLSPKLPGE
96Pyropia_yezoensis_Localdb__L  KDDEINYRFE VVTFRGDEGW VIGKPSLLIH TRDGGVSWER VPLSPKLPGD
02Hemiselmis_andersenii_Eukary  ADEELTYRFE NISFFQNDGW VIGKPAILLH TKDGGKTWLR VPVSPKLPGE
04Guillardia_theta_Eukaryota__  ADEELTYRFE NISFEGQEGW VIGKPAIILY TKDGGKTWFR VPVSPKLPGE
68Emiliania_huxleyi_Localdb__L  ADEEINYRFT KMSFRDGEGW IIGKPAILLH TRDSGASWER VPLSPKLPGD
01QUERY_Bigel_p2saf_91760       SDDEIKYRWE SVSFKDNEGW AVGKPAIMVH STDGGKSWER IPFSSQLPGD
47Lotharella_amoeboformis_Euka  ---------- ---------- ---------- ---------- ----------

                               101
39Synechococcus_sp__JA-2-3Ba_2  PLLIQALGPG AAEMVTNVGA IYRTEDGGQT WHAQVDEPIG AIKNIARGPE
12Cyanophora_paradoxa_Eukaryot  PALITALGTG KAELATDIGA IYRTENSGQT WKAQIQEPLG VIRTVARSEN
21Synechococcus_elongatus_PCC_  PLLVTALGKG EAEMATDVAA IYRSRDGGKS WQAQVPDAAG VARSVSRSRD
15Thermosynechococcus_elongatu  PRLIKALGNG SAEMITNVGA IYRTKDSGKN WQALVQEAIG VMRNLNRSPS
35Acaryochloris_sp__CCMEE_5410  PYQVTALGPK SVEMITDLGA IYRTQDEAQH WTALVEEATG ATRNINRSED
24Leptolyngbya_sp__PCC_7375_Ba  PLMVTALAKG AVELATDVGA IYRTEDSGRH WSALVQAAVG VVRNMTRSED
34Leptolyngbya_sp__PCC_6406_Ba  PFMVTALKAN EVELATDVGA IYRTKDGGKN WKALVLGAVG VVRNMTRSED
32Dactylococcopsis_salina_PCC_  PYSIIAKGED TAEMTTNVGA IYETTNGGKN WQALVQEAVG VARNISRSPD
18Chamaesiphon_minutus_PCC_660  PATIKAIGEN SAEMTTDIGA IYATQDAGKN WKSLVSGAVG VFRTINRSPE
31Gloeocapsa_sp__PCC_73106_Bac  PYGIIALGPK SAEMVTKLGA IYKTVDGGQN WKALVEGSVG VARTLNRSPN
22Cyanobacterium_aponinum_PCC_  PYDITALDDQ TAEMVTNLGA IYKTTDGGKT WKALVEGAVG VARSITRSAD
28Microcystis_aeruginosa_DIANC  PYGIIALNDK TAEMVTDLGA IYRTKDGGKT WQALVEGAVG VARTIVRSHD
19Stanieria_cyanosphaera_PCC_7  PDGIVALGTA SAEMVTDLGA IYKTTDGGKT WKALVEGAVG VARHINRSAD
17Pleurocapsa_sp__PCC_7327_Bac  PNGIVALGSK MAEMVTDLGA IYKTSNGGRT WQALVEGAVG VARTIARSAD
27Cyanothece_sp__PCC_7822_Bact  PYNIVALAPQ TAEMVTDLGA IYRTTDGGRT WKALVEGAVG VARNIRRSSD
13Geitlerinema_sp__PCC_7407_Ba  PQVITALGPN SAELTTNVGA IYRTQDGGKN WKALVQEAVG VFRNISRSSD
33Calothrix_sp__PCC_6303_Bacte  PINIAALSGN TAEMATDVGA IYQTSDGGKN WKAQVQDAVG VVRNITRSPD
29Rivularia_sp__PCC_7116_Bacte  PVNIVAFGDN KAEMATDVGA IYKTEDGGKN WKAQVAESVG VVRNIERSED
36Nodularia_spumigena_CCY9414_  PIAIYALGEN TAEMATDVGA IYRTQDGGIN WKAQVEGAVG VVRNIERSPD
14Fischerella_sp__JSC-11_Bacte  PISIIAQGAN SAEMATDVGA IYKTTDGGQN WKAQVEEAVG VVRNLERSAD
23Oscillatoriales_cyanobacteri  PNTIEALGPQ SAEMTTNIGA IYRTSDGGKT WKAMVEEAVG VVRNISRSPN
25Oscillatoria_nigro-viridis_P  PSTIAALGPN SAQMTTDVGA IYRTSDGGKN WKAVVQDSFG VVRNINRSED
38Oscillatoria_sp__PCC_6506_Ba  PSTIVALGPQ SAEMTTDVGA IYQTADGGKT WKALVQEAFG VVRNINRSPD
20Crinalium_epipsammum_PCC_933  PNTILALDSN SAEMTTNVGA IYRTSDGGRT WQALVQDALG VVRNISRSPD
30Synechocystis_sp__PCC_7509_B  PDTIIALGAS SAEMTTDVGA IYRTTDGGKN WKAMVQEAVG VVRNIERSDD
16Gloeocapsa_sp__PCC_7428_Bact  PNTIVALGSH AAEMTTDVGA IYRTTDSGKT WKAMVQEAVG VVRNIARAAD
```

```
26Chroococcidiopsis_thermalis_  PNTIVALGQQ SAEMTTDVGA IYRTTDGGKN WKAMVQEAVG VLRNVARSED
08Ostreococcus_lucimarinus_CCE  PVLITAVQDG TAEMVTDEGA IYFTKDAARN WKAAVEETVG TFSTISRNDN
09Micromonas_pusilla_CCMP1545_  PVLITAVSDG VAELVTDEGA IYLTTDTARN WKAAVEETIG TLSTIARNPD
10Chlamydomonas_reinhardtii_Eu  GVVLDSLAPG QAEMITDQGA IYVTDNTAYT WTAAVQETVG SFSNLARSSS
07Brachypodium_distachyon_Euka  MVYIQATGEQ SAEMVTDEGA IYVTSNRGYN WKAAVQETVG TFNTVNRSPD
06Selaginella_moellendorffii_E  PVVIQATGDK QAEMVTDEGA IYVTSNAGYN WKAAVEETVG TFNTVNRSPQ
11Physcomitrella_patens_subsp_  PITIRGTGPD SAEMVTDEGA IYVTSNKGYN WKAAVEETVG TLNTVNRSPA
48Cyanidioschyzon_merolae_Euka  PLLVTALGPN CAEMVTSSGA IYVTENGGIN WKALVRETIG SIVSVSRDVH
67Ectocarpus_siliculosus_Local  PSNIVALGGA KAEMTTSSGA IYYTKNAGMN WSAQVKETVG SIIGTTRAED
65Aureococcus_anophagefferens_  PTGVVALGKS KAEMTTSTGA IYSTTNAGRN WKAQVKETIG SVATIQREKD
03Phaeodactylum_tricornutum_CC  PTAIVALGPS RAEMTTSSGA VYVTDNAGRN WKAQVKETIG SIINQVRDDL
05Thalassiosira_pseudonana_CCM  PTGILATGPN SAEMITSSGA VYTTENAGRN WKAQVKETIG SIVNEQRDSK
96Pyropia_yezoensis_Localdb__L  PVLITALDKE KAEMTTTAGA VYLTENGGRN WKALVKETIG TIISVLRDGQ
02Hemiselmis_andersenii_Eukary  PILITALGPD TAEMTTSAGA IYVTNNGGRN WKAQVKETIG NIIGVIRDKN
04Guillardia_theta_Eukaryota__  PCLIKALGSE SAELTTTSGA IYVTNNAGRN WKAQVKETIG NVINVIRNSE
68Emiliania_huxleyi_Localdb__L  PYNVVALGPG KAEMSTSAGA IYTTDNSGRN WKAQVRETIG SINDIQRDAD
01QUERY_Bigel_p2saf_91760       FLGVTALGDG QAETITNAGG IYRTVNAGKN WKAQVLETIG SINSVVRDNM
47Lotharella_amoeboformis_Euka  ---------- ---VITSAGA IYKTVNGGKN WKAQVVETIG SINSVVRDTK

                                151
39Synechococcus_sp__JA-2-3Ba_2  GEYLAVSSRG SFYFLYTPES RTWKPYPRES SRRIQNMGFG PHGSAWKLNQ
12Cyanophora_paradoxa_Eukaryot  GSYVAVSAKG NFYSTWKEGD DKWISHPRQS SRRIQSMGFT NNNRLWMLTR
21Synechococcus_elongatus_PCC_  GRYLAVSARG NFYSTWKPGD TTWTPHQRTS SRRLQLMGFG PDDRTWLIAR
15Thermosynechococcus_elongatu  GEYVAVSSRG SFYSTWEPGQ TAWEPHNRTT SRRLHNMGFT PDGRLWMIVN
35Acaryochloris_sp__CCMEE_5410  GKYVAISSRG SFYTTFDPSE TSWQSHDRNG ARRIQTMGFD PNGNPWILNK
24Leptolyngbya_sp__PCC_7375_Ba  GRYVAVSSRG NFYSTWEPGQ DSWQPHNREN SKRLQNMGFT SDGDLWLIAR
34Leptolyngbya_sp__PCC_6406_Ba  GSYVAVSSRG NFYSTWTPGE LDWEPHNRQN SRRLQNIGFD KSGGLWLIAR
32Dactylococcopsis_salina_PCC_  GKYITVSARG NFYSTWTPGD QAWQAHERNT SRRVQNMGFT PDGRVWLLAR
18Chamaesiphon_minutus_PCC_660  GKYVAVSAKG NFYSTWAPGQ ESWEPHNRNS SRRVSNMGFA DDDRLWMLAR
31Gloeocapsa_sp__PCC_73106_Bac  GEYVAVSARG NFYSTWEPGQ AEWTPHDRTS SRRLQNMGFG KDGRLWLLAR
22Cyanobacterium_aponinum_PCC_  GKYVAVSARG NFYSTWQPGD TEWTPHQRTS SRRLQNMGFL DGGRLWLIAR
28Microcystis_aeruginosa_DIANC  GKYVAVSARG NFYSTWEPGS TEWQPHNRLS SRRLQKVGYG EKGELWALAR
19Stanieria_cyanosphaera_PCC_7  GKYVAVSARG NFYSTWEPGQ TEWTPHQRTS SRRLQNMGFN SDDSLWLLAR
17Pleurocapsa_sp__PCC_7327_Bac  GKYVAVSARG NFYSTWEPGQ TEWTPHNRTS SRRLQTMGYS EDGRLWLLAR
27Cyanothece_sp__PCC_7822_Bact  GKYVAVSARG NFYSTWEPGQ SEWTPHNRNS SRRLQTMGYA DNGGLWLIAR
13Geitlerinema_sp__PCC_7407_Ba  GKYVTVSARG NFYSTWEPGA DRWVQHNRNS SRRLQNMGFT ADGNLWLIAR
33Calothrix_sp__PCC_6303_Bacte  GKYVAISAKG NFYSTWEPGT AAWVPRNRNS SRRLENMGFT SEGKLWMLAR
29Rivularia_sp__PCC_7116_Bacte  GKYIAVSGKG NFYSTWEPGQ NAWVQHNRNS SRKVENMGFA QNGQMWMLAR
36Nodularia_spumigena_CCY9414_  GKYVAVSAKG SFYSTWEPGQ TSWEPHNRNN SRRLENMGFA ENGQLWLLAR
14Fischerella_sp__JSC-11_Bacte  GKYVAVSAKG NFYSTWEPGQ NAWVPHNRNS SRRVENMGFD ENGRLWMLAR
```

```
23Oscillatoriales_cyanobacteri  GEYVAVSSRG NFYSTWQPGL SAWEPHNRNS SRRVQNMGFT SDGRLWMLAR
25Oscillatoria_nigro-viridis_P  GQYVAVSSKG NFYSVWKPGQ AAWEPHNRNS SRRLQNMGFT KDGRLWMLAR
38Oscillatoria_sp__PCC_6506_Ba  GQYVAVSSKG NFYSIWQPGQ DAWTPYNRNS SRRLQNMGFG KDGRLWMLER
20Crinalium_epipsammum_PCC_933  GKYVAVSTKG NFYSTWEPGL DAWVGHNRNS SRRVQNMGFT QDGRMWMLAR
30Synechocystis_sp__PCC_7509_B  GKYLAVSAKG NFYSTWEPGQ EAWQGHNRNS SRRVQNMGFG KDGRLWMLAR
16Gloeocapsa_sp__PCC_7428_Bact  GRYLAVSAKG NFYSIWEPGK EAWEGHNRNS SRRVQNMGFA PDGRLWMLAR
26Chroococcidiopsis_thermalis_  GKYIAVSAKG NFYSTWEPGQ TAWQGHNRNS SRRLQNMGFG KDGRVWMLAR
08Ostreococcus_lucimarinus_CCE  GEYLGLSSRG NFYMSWAPGQ AYWQPHNRTS ARRVQSMGWR PDGGIWELTR
09Micromonas_pusilla_CCMP1545_  GEYLGLSSRG NFYMTWTPGQ AYWQPHNRSS GRRVQSMGWR PDGGIWELTR
10Chlamydomonas_reinhardtii_Eu  GDYVAVSSRG NFYMTWSPGQ TYWMPHNRPA PRRLQNMGFT PAGEVWVTTR
07Brachypodium_distachyon_Euka  GRYVAVSSRG NFYLTWEPGQ LYWQPHNRAV ARRIQNMGWR ADGGLWLLVR
06Selaginella_moellendorffii_E  GDYVAVSSRG NFYMTWEPGQ PYWQPHNRRS ARRIQNMGWR ADGGLWLVVR
11Physcomitrella_patens_subsp_  GDYVAVVSRG NFFLTWEPGQ PYWQPHNRTS ARRIQNMGWR ADGGLWLVVR
48Cyanidioschyzon_merolae_Euka  GNYIAIPSRG NFFLTWVPGS DFWTPHARST SRRISAIGFD ATKGIWETIR
67Ectocarpus_siliculosus_Local  GSYLAVSSRG NFYLTWSPGQ DFWIPHNRGT SRRIQNMGFR AAEGLWMTLN
65Aureococcus_anophagefferens_  GTYLAVSSRG NFYLTWSPGQ DFWLPHNRGT PRRIQAMGFD SSKGVWMTLN
03Phaeodactylum_tricornutum_CC  GAYLAVSSRG NFFLTWEPGQ DFWIPHNRGT PRRIQNMGFD IKKGLWMTLN
05Thalassiosira_pseudonana_CCM  GAYIAVSSRG NFFLTWEPGQ DFWIPHNRGT PRRIQNMGFD IKNGLWMTLN
96Pyropia_yezoensis_Localdb__L  GSYLAVSSRG NFFLTWSPGQ DFWVPHGRDS SRRIQAMGFD VNEGLWMSTR
02Hemiselmis_andersenii_Eukary  GKYLAISSRG NFFLTWQPGQ EFWIPRARDT SRRIQSMGFD LEQGLWMSTR
04Guillardia_theta_Eukaryota__  GKYLAISSRG NFYLTWEPGQ DFWIPRARET SRRIQSMGFD NQKGIWMSTR
68Emiliania_huxleyi_Localdb__L  GSYLAVSSRG NFYLTYRPGD EFWIPHNR-- -------GFR LADGLWMSTA
01QUERY_Bigel_p2saf_91760       GQYIGVNARG NFYLTFKPGS DAWMPHNSRT IRRIVTMGVD LTNSMWMTTR
47Lotharella_amoeboformis_Euka  GQYIGVNARG NFYLTFKPGD NAWMPHNRKT IRRIVTMGVD LTEGMWMTTR

                               201
39Synechococcus_sp__JA-2-3Ba_2  GAEIAFTDDF TSGQWSSPLR PGLSFGYLNA AYQNDHDLWV VGGGATLIHS
12Cyanophora_paradoxa_Eukaryot  GGQLWFSSND SFPNWEGPKT PEFGLGLLNL AFKTPTEIWV SGGSGILLSS
21Synechococcus_elongatus_PCC_  GGRLQFSIDN WEDAWGTAIE PEAGWGFLDL AYRSKQEIWL SGGSGTLLVS
15Thermosynechococcus_elongatu  GGKIAFSDPD NSENWGELLS PLNSVGFLDL AYRTPNEVWL AGGAGALLCS
35Acaryochloris_sp__CCMEE_5410  GGQVQFSENG DY-AWDEAFT PGNKIGLLDL AYRTPDEVWI SGGSGTLLCS
24Leptolyngbya_sp__PCC_7375_Ba  GGQMQFSQTD EDESWGEPLR PETSWGFLDV GYRTDNELWV SGGSGTLLMS
34Leptolyngbya_sp__PCC_6406_Ba  GGQLQFSPTG SEDGWQDPLN PESSWGLLDM GYRTPDEIWV SGGSGNLLNS
32Dactylococcopsis_salina_PCC_  GGQVQFTESE DFDSWQEPEY PESSKGLLDI GYRTEDEFWV SGGSGDLFRY
18Chamaesiphon_minutus_PCC_660  GGQLQFSSPG ATDKWEEEVF PATNIGLLDL AYRTPGEVWV SGGSGDLLVS
31Gloeocapsa_sp__PCC_73106_Bac  GGQLQFSEPE AWEEWGEVKY PETSWGLLDM GYRTDTELWA AGGSGNLLRS
22Cyanobacterium_aponinum_PCC_  GGQIQFSKPN NFEEWEEAIT PETSWGFLDL TVQNQEQIWL AGGSGNLLLS
28Microcystis_aeruginosa_DIANC  GGQLQFTSPN DLDTWEDKVF PETSWGLLDL NYRTPEEIWV AGGSGNLLVS
19Stanieria_cyanosphaera_PCC_7  GGQIQFSEPE DFEAWQEPIY PETSWGLLDI AYRNPEEIWV AGGSGNLLAS
17Pleurocapsa_sp__PCC_7327_Bac  GGQIQFASTD GQEEWGEVIY PETSWGLLDL AYRTPEEIWV SGGSGNVLVS
```

```
27Cyanothece_sp__PCC_7822_Bact   GGQLQFTSPE DLETWDEALY PETSWGLLDL SYRTPEEMWI AGGSGNLLVS
13Geitlerinema_sp__PCC_7407_Ba   GGELQFTQ-G DYEAWDEPVN PETSWGLLDL AYRTPDELWV TGGSANLLRS
33Calothrix_sp__PCC_6303_Bacte   GGQIQFTDPE DDEKWLDPQK PEYSWALLDM AYRNSDDVWA TGGSGSLLHS
29Rivularia_sp__PCC_7116_Bacte   GGQVQFSEPN SLEEWLDAQY PETSWGLLDL AYRTPSEIWL SGGSANLLHS
36Nodularia_spumigena_CCY9414_   GGQVQFSEPN NPEEWQEAQN PETSWGLLDL AYRTPDELWI GGGSGNLLSS
14Fischerella_sp__JSC-11_Bacte   GGQIQFSETA DGEKWLEPQN PETSWGLLDM AYRTPDEIWV SGGSGNLLRS
23Oscillatoriales_cyanobacteri   GGQIQFTSPE NSEEWTDPIN PETSWGLLDL AYRTPDEVWV AGGSGNLLLS
25Oscillatoria_nigro-viridis_P   GGQIQFSDPE SPEGWGKPFF PDASWGLLDM AYRTDNEVWV AGGGGNLLCS
38Oscillatoria_sp__PCC_6506_Ba   GGQIQFSDPQ NRDEWGEPFS PDASWGLLDL VYRTNDEIWL AGGGGNLLCS
20Crinalium_epipsammum_PCC_933   GGQVQFSKSD DIESWDEVQY PETSWGLLDL AYRTPDEIWL AGGSGNLLCS
30Synechocystis_sp__PCC_7509_B   GGQIQFSQPN TLDEWDEAQY PETSWGLIDL AYRTPEEIWL AGGSGNLLCS
16Gloeocapsa_sp__PCC_7428_Bact   GGQVQFTKPD NPDEWEEAQY PETSWGLLDL AYRTPEEIWI AGGSGNLLCS
26Chroococcidiopsis_thermalis_   GGQVQFTKAD NTEEWEEVQN PETSWGLLDL AYRTPEEIWV TGGSGNLLCS
08Ostreococcus_lucimarinus_CCE   GGGIFFSAET GLSEFNEGRI GSRGFGLLDL GYTSGKTFWT VGGSGSVFYS
09Micromonas_pusilla_CCMP1545_   GGGIYFSNTA SLDDFSEGRI GSRGFGLLDL SYTDGKQFWI VGGSGSVFVS
10Chlamydomonas_reinhardtii_Eu   GGDVLVSEDA GLEKFKDVTI NSRGFGILDV GFRDKVA-FA CGGSGSLYRS
07Brachypodium_distachyon_Euka   GGGLFLSKGT G-EEFEEASV QSRGFGILDV GYRKDEA-WA AGGSGVLLKT
06Selaginella_moellendorffii_E   GGGLYLGKCL G-EDFEEASI PSRGFGILDL GYRKDEA-WA AGGSGMLLKT
11Physcomitrella_patens_subsp_   GGGLYVSKGT GVEDFEEQKI PSRGFGILDV GYRKDEA-WA AGGSGILLRT
48Cyanidioschyzon_merolae_Euka   GGGLGFTKPN VNIAFDMVDS KTGGYGILDV AFQDDRHVWA AVGGGSMYRS
67Ectocarpus_siliculosus_Local   GGAMQMSTTL P-PLFSETAI KSGGYGIIDV AWKNDNEAWA VGGGGSLWYS
65Aureococcus_anophagefferens_   GGQMALGGKS AVPTFNVINL NSGGYGILDA AWRDSKEAWA VGGGNTMYVS
03Phaeodactylum_tricornutum_CC   GGKLFISSEE P-FDFQEADI KTGGYGITDV AWRNPKEVWA VGGSNTMYVS
05Thalassiosira_pseudonana_CCM   GGKLLVSSSD SVFPFDEANI KTGGYGLTDV AWRDDNEVWA VGGSNTMYVS
96Pyropia_yezoensis_Localdb__L   GGGLQTTSGS PDLTFNKADL KSGGYGILDI AYRGTNEVWA ALGGGALYVS
02Hemiselmis_andersenii_Eukary   GGGLSFTKSQ PDFDFSSSDI KTGGYGILDV NFKNKDELWA ACGGGILYSS
04Guillardia_theta_Eukaryota__   GGGLSVSTKN FDFNFENIDI KTGGYGILDA AFVNDKDIWI ICGGGIVYNS
68Emiliania_huxleyi_Localdb__L   GGEISKTTSG QPIPFDRCQI KSGGYGILDV LFLDSNKAWA VGGGGTIFGS
01QUERY_Bigel_p2saf_91760        AGGVSFVKGD AN--FYETSI NSGGYGILDV TRRNGD-LWC VGGGGKIFLS
47Lotharella_amoeboformis_Euka   AGGVSFAKGK SD--FVDTTI NSGGYGILDV TKLNGD-MWC VGGGGKSS-S

                                 251
39Synechococcus_sp__JA-2-3Ba_2   PDGGKTWEQA KKLSNIPANF YSIEFFGPEQ GFILGQRGTL LRYSS
12Cyanophora_paradoxa_Eukaryot   QDTGNTWKKE TSTDNIPSNF YKISFIDKEV GFVLGNQGTL LRY--
21Synechococcus_elongatus_PCC_   EDGGEHWQRD RVIAKLPSNL YTIKFFAPKQ GFVLGQRGLL LRYEA
15Thermosynechococcus_elongatu   QDGGQTWQQD VDVKKVPSNF YKILFFSPDQ GFILGQKGIL LRYTA
35Acaryochloris_sp__CCMEE_5410   LDGGQTWQKD STVEDVPSNL YKIIFVSSDQ GFIIGQSGTL LRFPK
24Leptolyngbya_sp__PCC_7375_Ba   PDGGETWLKD EPAGDVPSNL YRVKFFNQDQ GFILGQRGYL LRYEA
34Leptolyngbya_sp__PCC_6406_Ba   QDGGKTWYKD KELSAVPSNF YRILFMSPEQ GFILGQRGYL LRYKA
32Dactylococcopsis_salina_PCC_   AQTKGVWEKD REMQNVPGNF YRVKFFSPEK GFILGDRGTV LKYTN
```

```
18Chamaesiphon_minutus_PCC_660  TDSGKTWLKD SNVEQVPSNL YKILFFSPDR GFIIGQRGII LKYQG
31Gloeocapsa_sp__PCC_73106_Bac  LDDGETWEKD RDVENIASNF YRVVFISPEQ GFVLGQNGIL LKYEK
22Cyanobacterium_aponinum_PCC_  KDNGQSWFKD REIESVPSNL YKIVFVGDNK GFILGERGVL LKYQS
28Microcystis_aeruginosa_DIANC  DDGGQSWQKD RAVESVPSNL YRIVFINSDK GFVLGQNGVL LKYPS
19Stanieria_cyanosphaera_PCC_7  FDAGKTWQKD REVEEVPSNF YKVVFLSPEQ GFVLGERGVL LKYTT
17Pleurocapsa_sp__PCC_7327_Bac  FDNGKTWQKD REIEDTPSNL YKIVFVTPEK GFILGQKGVL LRYPA
27Cyanothece_sp__PCC_7822_Bact  FDNGKTWEKD REVENVPSNL YKIVFVNSEK GFVLGQKGVL LKYPT
13Geitlerinema_sp__PCC_7407_Ba  VDGGKTWEKD RSVEDIPSNF YRVVFFDGDK GFVLGQRGTL LKYNS
33Calothrix_sp__PCC_6303_Bacte  PDGGKTWEKD TEMEEVPANF FKVVFFSPEQ GFAIGDRGYL LKYVA
29Rivularia_sp__PCC_7116_Bacte  SDGGQTWEKD RDVEDVATNF YKVVFLSPEK GFVIGDRGFL LKYES
36Nodularia_spumigena_CCY9414_  ADGGQTWEKD RDVEGVAANL YKVVFFNSDQ GFVIGDRGVL LKYSP
14Fischerella_sp__JSC-11_Bacte  SDGGKTWEKD RDVEGIPANF YKIVFFSPEQ GFVIGDRGYL LKYKS
23Oscillatoriales_cyanobacteri  RDGGKTWEKD RFVEDVPSNF YRIKFFGTEK GFIIGQQGTL LKYNS
25Oscillatoria_nigro-viridis_P  FDGGKTWQKD REVEDVPANF YRIVFMGPDR GFVLGASGTL LKYSA
38Oscillatoria_sp__PCC_6506_Ba  FDGGKTWQKD REVEDVPANF YKIVFMTPEK GFIIGATGTL LKYSA
20Crinalium_epipsammum_PCC_933  LDGGKTWQKD REVEDVASNF YKIVFVTPEQ GFIIGQRGIL LKYEA
30Synechocystis_sp__PCC_7509_B  FDGGKTWLKD REIENVPSNL YKIVFVKPDQ GFIIGQRGVL LKYAV
16Gloeocapsa_sp__PCC_7428_Bact  FDGGKTWQKD REVEEVPSNL YKIVFLTPEK GFVVGQRGIL LKYPS
26Chroococcidiopsis_thermalis_  PDGGKTWQKD RGVESVPSNL YKIVFLSPEK GFIIGQRGIL LRYQA
08Ostreococcus_lucimarinus_CCE  TDAGKSWKRD RGTDNVAANL YNVKFQSEDQ GFILGNDGIL LRFTG
09Micromonas_pusilla_CCMP1545_  NDAGKSWKRD RGTDNVAANL YRVRFQSNKQ GFVLGNDGIL LRFTG
10Chlamydomonas_reinhardtii_Eu  EDGGKNWKRD RSTDDVAGNL YAIKFFNPQL GFVLGNDGIL LRYTG
07Brachypodium_distachyon_Euka  MNGGKSWVRD KAADNIPGNL YSVKFVGDNQ GFVLGNDGVL LRYVG
06Selaginella_moellendorffii_E  TDGGNTWSRD RVADSIAANL YSVKFLDNKK GFVLGNDGVL LRYLS
11Physcomitrella_patens_subsp_  TDGGKSWIRD KVADKIAANL YSVKFIDN-K GFVLGNDGVL LRYLG
48Cyanidioschyzon_merolae_Euka  DDGGKTWRRD PLVSKVGANL YKIKFFGSQR GFVLGADGVL LKFHP
67Ectocarpus_siliculosus_Local  TDGGETFKFS SGATNIGANL YDVKFFGGDK GFAIGSDGVL LKYIP
65Aureococcus_anophagefferens_  KDGGKNFKFD DSADKIPGNL YAIKFFGPNT GFALGSDGVL MKFNA
03Phaeodactylum_tricornutum_CC  FDGGKKFQFD KSGNAYPGNL YNVKFFPQNA GWALGSNGLL LRYTG
05Thalassiosira_pseudonana_CCM  KDNGRNFVFD KSANDIPGNL YNVKFFRENA GWALGSNGLL LKYVG
96Pyropia_yezoensis_Localdb__L  KDGGKSFKPD KTVGKIGTNL YKIKFFSKDV AFALGASGVL LRYNP
02Hemiselmis_andersenii_Eukary  IDGGKSWKK- DDEDKVIGSL YKIKFFNSKL GFVLGSNGVL LRYVE
04Guillardia_theta_Eukaryota__  TDKGKNWTKV DGIDKLSGNL YKIKFVNNNK GFILGSNGLL LRYQ-
68Emiliania_huxleyi_Localdb__L  TDGGRSWQKD KSADDLPTNL YKIKSFSNGR VYILGSNGAL SPRRA
01QUERY_Bigel_p2saf_91760       RNNGKTWTAD SSGDNQPANF YKIKFYDDKR GFILGSAGVL MKFND
47Lotharella_amoeboformis_Euka  RATGEELDC- ---------- ---------- ---------- -----
```

Alignment: PRK

```
01Lth_prk        ---------- ---------- ---------- ---------- ---------- ----------
40Gymnochl       ---------- ---------- VNAIFGTTTI TTICLDDYHT LDRTGRKDTG ISALDVKANN
38Chlorarac      ---------- ---------- VNAIFGTTMI TTICLDDFHT LDRTGRKDTG ISTLDVKANN
39Bigelowi       PVIFGVAADS GCGKSTFLRR VNAIFGTTTI TTICLDDFHT LDRTGRADTG ISALDVRANN
10Prymnesi       PVVVGVAADS GCGKSTFMRR LTSIFGIGRT TVICLDDYHK WDRTGRKSNG ITALHEACQD
11Emiliani       PVVIGLAADS GCGKSTFMRR VTGIFGIGRT TVICLDDYHK WDRTGRKSNG ITALHEACQN
02Eut            ---------- ---------R LTGIFGGGFT TVMCLDDYHL NDRAGRKVTG LTALDERENN
03Euglena        TVLIGVAADS GCGKSTFMRR LTGIFGGGFT TVMCLDDYHL NDRAGRKVTG LTALDQRENN
04Guillardia     PVVIGLAADS GCGKSTFMRR VTACFGIGRT TVICLDDYHL NDRQGRKKTG LTALDPRENN
41Cschyzon       PVMVGVAADS GAGKSTFLRR VMRMFGSDII TVICLDDWHN RDRQGRKEDN ITALDENCQN
12Pavlova        AFVVGVAADS GCGKSTFMRR LTNIFGIGRT TVICLDDYHL YDRKGRSANK ITALHKDCQK
44Chondrus       PVIIGVAADS GCGKSTFLRR VTGIFGTEVI SVICLDDYHL HDRMGRREAR VTALDEAANN
42Porphyrid      ---------- ---------- ---------- -------LPL PRPQGPQGRQ RTALDEKANN
07Thalassi       PIVIGLAADS GCGKSTFMRR VTSTFGGGFA TVICLDDYHL NDREGRKVSG LTALNTAEQK
05Odontella      PIVIGVAADS GCGKSTFMRR LTSIFGGGFT TVLCLDDYHL NDRNGRKVTQ RTALDPLENN
06Phaeodact      PIIIGVAADS GCGKSTFMRR LTNIFGGGFT TVICLDDYHL NDRAGRKVTM RTALDPEENN
08Vaucheria      VVIIGVAADS GCGKSTFMRR LTNIFGGGFT TVICLDDYHL NDRNGRKVSG RTALHTDEQK
09Ectocarpus     VVLIGVAADS GCGKSTFMRR LTSIFGGGFT TVLCLDDYHL NDRGGRKVSG RTALHTEEQK
43Galdieria      PVIIGVAADS GCGKSTFLRR VNEIFGTKVV TVICLDDFHT LDRKGRAEKK VTALNPEANN
13Microm_p       PVIIGLAADS GCGKSTFMRR MTSLFGGGNT TVLCLDDYHL NDRNGRKESG LTALNLKEQN
14Microm_stn     PVLIGLAADS GCGKSTFMRR MTSLFGGGNT TVLCLDDYHL NDRNGRKESG LTALNLKEQN
15Ostreoc_l      PVIIGLAADS GCGKSTFMRR MTSLFGGGNT TVLCLDDYHL NDRAGRKTSG LTALNLKEQN
16Ostreoc_t      PVIIGLAADS GCGKSTFMRR MTSLFGGGNT TVLCLDDYHL NDRQGRKDSG LTALNLKEQN
17Chlorella      SVMIGLAADS GCGKSTFMRR MIGVFGGGNA TVICLDDYHC LDRTGRKEKG VTALDPKAQD
18Chlorel_sp     IVVIGLAADS GCGKSTFMRR VTGIFGGGNT TVICLDDYHS LDRNGRKEAG VTALAPESQN
19Chlamydom      TVVIGLAADS GCGKSTFMRR MTSIFGGGNT TVICLDDYHC LDRNGRKVKG VTALAPEAQN
20Volvox         TVVIGLAADS GCGKSTFMRR MTSIFGGGNT TVICLDDYHC LDRNGRKVKG VTALAPEAQN
27Oryza_s2       TVVIGLAADS GCGKTTFVRR LTSVLGGGNA TVICLDDYHS LDRAGRKERG VTALDPRAND
34Closterium     TVVVGLAADS GCGKSTFMRR MTGVFGGGNT TVICLDDYHS LDRNGRKEAN VTALDPKANN
26Arabidopsi     TIVIGLAADS GCGKSTFMRR LTSVFGGGNT TVICLDDYHS LDRYGRKEQK VTALDPRAND
24Zea_mays       PVVIGLAADS GCGKSTFMRR LTSVFGGGNT TVICLDDYHS LDRTGRKEKG VTALDPRANN
23Oryza_s1       PVVIGLAADS GCGKSTFMRR LTSVFGGGNT TVICLDDYHS LDRTGRKEKG VTALDPRAND
25Triticum       PIVIGLAADS GCGKSTFMRR LTSVFGGGNT TVICLDDYHS LDRTGRKEKG VTALDPKAND
31Selaginel      TIVIGLAADS GCGKSTFMRR LTSVFGGGNT TVICLDDYHS LDRTGRKEKG VTALDPKANN
29Spinacia       TIVIGLAADS GCGKSTFMRR LTSVFGGGNT TVICLDDFHS LDRNGRKVEK VTALDPKAND
30Beta_vu        ---------- --GKSTFMRR LTSVFGGGNT TVICLDDFHS LDRTGRKEKG VTALDPRAND
22Populus        TVVIGLAADS GCGKSTFMRR LTSVFGGGNT TVICLDDYHS LDRTGRKEKG VTALDPRANN
28Pisum_sa       TIVIGLAADS GCGKSTFMRR LTSVFGGGNT TVICLDDYHS LDRTGRKEKG VTALDPKAND
```

```
21Arteminis      TIVIGLAADS GCGKSTFMRR LTSVFGGGNT TVICLDDYHS LDRTGRKEEG VTALDPRANN
32Physcomi       TVVIGLAADS GCGKSTFMRR LTSVFGGGNT TVICLDDYHS LDRYGRKEKA VTALDPRANN
33Chara_braunii  TVVIGLAADS GCGKSTFMRR LTSVFGGGNT TVICLDDYHS LDRTGRKVHG VTALDPRANN
36Gloeochaete    ---------- ---------- CMTFFGQEFL TVICLDDYHT LDRKGRTEAG VTALDPRANN
37Cyanophora     PVIIGVAGDS GCGKSTFLRR LYDIFGTEVL TVICLDDYHT LDRTGRKEAG VTALDPKANN
35Glaucocyst     ---------- ---------- ----FGSDVL TVICLDDYHT LDRQGRKEAG VTALDPRANN
47Synechoco      VVLIGVAGDS GCGKSTFLNR LADLFGTELM TVICLDDYHS LDRKGRKEAG VTALDPRANN
54Nostoc_pun     VVLIGVAGDS GCGKSTFLRR LIDLFGEDLM TVICLDDYHS LDRKQRKETG ITALDPRANN
55Nodularia      VVLIGVAGDS GCGKSTFLRR LIDLFGEEFM TVICLDDYHS LDRKQRKETG ITALDPRANN
52Nostoc_sp      VVLIGVAGDS GCGKSTFLRR LIDLFGEEFM TVICLDDYHS LDRKQRKETG ITALDPRANN
53Anabaena_v     VVLIGVAGDS GCGKSTFLRR LIDLFGEEFM TVICLDDYHS LDRKQRKETG ITALDPRANN
56Synech2        VVLIGVAGDS GCGKSTFLRR LADLFGEEFM TVICLDDYHS LDRKQRKEKK VTALNPKANN
46Microcys       VVLIGVAGDS GCGKSTFLRR LTDLFGAEFM TVICLDDYHC LDRKQRKEVG VTALNPKANN
48Microco_ch     VVLIAVAGDS GCGKSTFLHR LADLFGEEFM TVICLDDYHS LDRKGRKAAG VTALNPKANN
45Cyanothe       VVLIAVAGDS GCGKSTFLRR LTDLFGEEFM TVICLDDYHS LDRKGRKAAG VTALNPKANN
49Synech1        VVLIGVAGDS GCGKSTFLRR LTDLFGEEFM TVICLDDYHS LDRQGRKAAG VTALDPRANN
57Trichodes      VVLIGVAGDS GCGKSTFLRR ITDVFGPEFV TVICLDDYHS LDRKQRKETG ITALDPRANN
58Lyngbya        VVLIGVAGDS GCGKSTFLRR ITDIFGEDFV TVICLDDYHS LDRKQRKETG ITALDPRANN
50Thermosyn      VVLIGVAGDS GCGKSTFLRR LADLFGEDFM TVICLDDYHS LDRKQRKEMG ITALDPRANN
51Acaryochl      VVLIGVAGDS GCGKSTFLRR LEDLFGEQFI TVICLDDYHS LDRYQRKETG ITALDPRANN

                61
01Lth_prk        ---------- ---------- ---------- ---------- ---------- ----------
40Gymnochl       FKLMADQLKA LKQGRAIKKP IYNHDTGAID PVETIHPNHI VIVEGLHPML DKDVIDSLDF
38Chlorarac      FKLMADQLKA LKQGRAIKKP IYNHDTGAID PVEVIHPNHI IIVEGLHPML DKDVVESLDF
39Bigelowi       FALMADQLKA LKQGRAIKKP IYNHDTGAID PVETIHPNHI IIVEGLHPML DKDVIESLDF
10Prymnesi       WDKMAADVLD LKAGKSVSKP IYNHVTGELD PYEDVDPTPI VIFEGLHPMY DERVNKALDL
11Emiliani       WDKMAADVTD LKAGKAVEKP IYNHITGELD PDEPVQPTPI VIFEGLHPMH DERVNEALDL
02Eut            FDLMYEQLNA LR-----TKP IYNHVNGTLD TPEKIDSSPL MIIEGLHPLL DDRVAGLVDF
03Euglena        FDLMFEQMSS LKRGETIAKP IYNHVNGTLD TPEEIAPASI MIIEGLHPLL DDRVAGLLDF
04Guillardia     FDLMYEQVKA LKEGKKVMKP IYNHVNGTLD EAEEITPTPI IIFEGLHPFY DKRVEELMDF
41Cschyzon       FDLMAEQLEA LKNGFDIMKP IYNHETGRID PPELVSPNHI VVVEGLHPMY DERVRKLLDF
12Pavlova        WDLMAEQVAA IKAGNSVMKP IYNHITGELD APEEIVPTDI VIFEGLHPML DDKVRSMLDL
44Chondrus       FELMASQIQA LREGKSIMKP IYNHETGEID ECELVEPNHI IVIEGLHPMY DTRVKKSLDF
42Porphyrid      FTLMAAQVKA LKEGKAIYKP IYNHDTGEHD PSELIEPNHL VVIEGLHPMY DPRVAELLDL
07Thalassi       FDLMFEHVKA LKEGKTIMKP IYNHVNGTLD TPEEIEPTPV IIIEGLHPFV DERVRELIDF
05Odontella      FDLMYEQIAA LKNGESIEKP IYNHVNGTLD TPETIVPTPI VIIEGLHPMY DERVRDLLDF
06Phaeodact      FDLMYEQVKA LKDGKTVEKP IYNHVNGTLD TPETIEPTPI IIFEGLHPMH DKRVLDLLDF
08Vaucheria      FDLMTEQLSA LKRGESVMKP IYNHVNGTLD TPEEVKPTPI VIIEGLHPFV DERVRDLLDF
09Ectocarpus     FDLMYEQLND LKNGKSVMKP IYNHVNGTLD TPEEIKPTPI VIIEGLHPFV DKRVRDLLDF
```

```
43Galdieria       FELMYQQIAA LKEGYDIMKP IYNHQTGLID PPELIQPNHI IVIEGLHPWY DARMKQLLDF
13Microm_p        FDLMYDQVKA LKEGKAVEKP IYNHVTGVFD PAEKIESPDI LILEGLHPFA DERVRDMFDF
14Microm_stn      FDLMYEQTKA LMEGKAVDKP IYNHVTGVFD PAEKIESPSI LILEGLHPFA DERVRDMFDF
15Ostreoc_l       FDLMYDQVKA LKEGKSVDKP IYNHVTGVFD PAEKIESPEV LILEGLHPFA DTRVRDMFDF
16Ostreoc_t       FDLMYEQVKA LKEGKSVDKP IYNHVTGVFD PAEKIESPEV LILEGLHPFA DTRVRDMFDF
17Chlorella       FELMYEQAKA IKEGKSIDKP IYNHVSGLLD PAETIKPPKI MVLEGLHPFY DERVRDLYDF
18Chlorel_sp      FDLMYEQVKA LKEGKAVDKP IYNHVTGLLD PPEPTSSPNI LVIEGLHPFY DERVNELVDF
19Chlamydom       FDLMYNQVKA LKEGKSVDKP IYNHVSGLID APEKIESPPI LVIEGLHPFY DKRVAELLDF
20Volvox          FDLMYNQVKA LKEGKAVDKP IYNHVTGLID APEKIDSPNI LVIEGLHPFF DKRVADLLDF
27Oryza_s2        FDLMYRQLKA IKEGRAVAKP IYNHATGLLD PPELITPPKI LVVEGLHPMY DERVRGLLDF
34Closterium      FDLMYEQVKA LKEGKSVMKP IYNHVSGLLD PPELIESPKI LVIEGLHPMY DERVRDLIDF
26Arabidopsi      FDLMYEQVKA LKNGIAVEKP IYNHVTGLLD PPELIQPPKI LVIEGLHPMF DERVRDLLDF
24Zea_mays        FDLMYEQVKA IKQGQAVQKP IYNHVTGLLD PPELITPPKI FVIEGLHPMF DERVRDLLDF
23Oryza_s1        FDLMYEQVKA IKEGKAIEKP IYNHVTGLLD PPELIQPPKI FVIEGLHPMF DERVRDLLDF
25Triticum        FDLMYEQVKA IKEGKAIEKP IYNHVTGLLD PAELIQPPKI FVIEGLHPMY DERVRELLDF
31Selaginel       FDLMYEQVKA LKEGKAVQKP IYNHVSGLLD PPELIQPPKI LVIEGLHPMF DSRVRELLDF
29Spinacia        FDLMYEQVKA LKEGKAVDKP IYNHVSGLLD PPELIQPPKI LVIEGLHPMY DARVRELLDF
30Beta_vu         FDLMYDQVKA LKQGKPVDKP IYNHVSGLLD PPEVIQPPKI LVIEGLHPMY DARVRELLDF
22Populus         FDLMYEQVKA IKDGTAVEKP IYNHVTGLLD PPELIKPPKI LVIEGLHPMY DQRVRDLLDF
28Pisum_sa        FDLMYEQVKA IKDGKSVQKP IYNHVTGLLD APELIKPPKI LVIEGLHPMY DSRVRELLDF
21Arteminis       FDLMYEQVKA LKDGTAVQKP IYNHVSGLLD PPELIKPPKI LVIEGLHPMY DERVRDLLDF
32Physcomi        FDLMYEQVKA LKEGKSVEKP IYNHVTGLLD APETIHPPKI LVIEGLHPMY DERVRELLDF
33Chara_braunii   FDLMYEQVKA LKEGKTVEKP IYNHVSGLLD PAETIAAPKI LVIEGLHPMY DERVRELLDF
36Gloeochaete     FDLMYEQIKA LKNLEAIEKP IYNHETGKLD PPETVYPNHI IVVAAFTEMF DPRVRELLDF
37Cyanophora      FDLMYEQIKA LKNGEAIQKP IYNHETGKID PAETVDPNHI IVVEGLHPMY DKRVRDLLDF
35Glaucocyst      FDLMYEQIKA LKNLEAIEKP IYNHETGKLD PPETVYPNHI IVVAAFTDMF DPRVRELLDF
47Synechoco       FDLMYEQVKA LKNGETIMKP IYNHETGLID PPEKIEPNRI IVIEGLHPLY DERVRELLDF
54Nostoc_pun      FDLMYEQIKT LKSGQAIDKP IYNHETGLID PPERVEPNHI IVVEGLHPLY DERVRSLIDF
55Nodularia       FDLMYEQIKA LKEGQVIQKP IYNHETGMID PPERVEPNHI IVVEGLHPLY DERVRSLLDF
52Nostoc_sp       FDLMYEQIKA LKEGQTINKP IYNHETGLID PPEIVKPNHI VVVEGLHPLY DERVRSLLDF
53Anabaena_v      FDLMYEQIKA LKEGQTINKP IYNHETGLID PPEIVKPNHI VVVEGLHPLY DERVRSLLDF
56Synech2         FDLMYEQIKA LKEGNTIDKP IYNHETGELD PPEKVEPNKI IVIEGLHPLY DERVRNLLDF
46Microcys        FDLMYEQIKA LKEGQAINKP IYNHETGMID PPEIIEPNKV IVIEGLHPLY DERVRALLDF
48Microco_ch      FDLMYEQIKA LKNGQAIDKP IYNHETGELD PPERVEPNKV VVIEGLHPLY DERVRSLVDF
45Cyanothe        FDLMYEQIKA LKSGQAIDKP IYNHETGELD PPERIEPNKV VVIEGLHPLF DERVRELVDF
49Synech1         FDLMYEQIKT LKSGQSIMKP IYNHETGLLD PPEKVEPNKV VVIEGLHPLY DERVRELVDF
57Trichodes       FDLMYEQIKA LKNLESIDKP IYNHETGMID PPEKIHPNHI IVIEGLHPLY DERVRELIDF
58Lyngbya         FDLMAEQIKA LKNGQSIMKP IYNHETGLID PPERIDPNHI IVIEGLHPLY DERVRELLDF
50Thermosyn       FDLMYEQIKA LKNGESIMKP IYNHETGTID PPEKVDPNHV IVIEGLHPLY DERVRSLIDF
51Acaryochl       FDLMYEQIKT LKGGQSIDKP IYNHETGLID PPERIDPNHV IVIEGLHPLH DERVRGLIDF
```

```
                121
01Lth_prk         ---------- ---------- ---------- ---------- -DKFVAPQKA NADVIISVEP
40Gymnochl        TFYIDVSDEV KKAWKIQRDV AERGHKVEDI IASIESRKED FEKFVAPQKA NADVIISVEP
38Chlorarac       TFYIDVSDPV KKAWKIERDM VERGHKKEDI IASIESRKPD FEKFVEPQKE TADVIISIEP
39Bigelowi        TFYIDVSDPV KKAWKIERDM VERGHKKEDI IASIESRKPD FEKFVEPQKA NADVIISIEP
10Prymnesi        TVYLDITDEV KFAWKAQRDI AERGATMEEV QKAIDGRKPD FAAYVEPQKA KADIIVQVLF
11Emiliani        TIYLDITDDV KFAWKAQRDI AERGATPEEV QAAIDGRKPD FAAYVEPQKA KADIIIQVLP
02Eut             SIYLDISERV KFAWKIQRDM AERGWKLEDV KAEIEKRKPD FHKYVAPQKS KGDIVIEVLP
03Euglena         SIYLDISDRV KFAWKIQRDM AERGWALEDI KKDIEKRKPD FDKYVAPQRA KADMVIEVLP
04Guillardia      KIYVDITPEV KFNWKVQRDH EERGHSIESI KQQIEARKPD FDAYIDPQKN KADCVIQVLP
41Cschyzon        SVYLDLDDEV KIAWKIQRDM AERGHTLEAI LASIEARKPD FERFILPQRQ YADAVIQVKP
12Pavlova         SIYLDISGXV KFAWKIQRDM QERGHSLESI KASIEGRKPD FDAFVAPQRA NADIVIEVLP
44Chondrus        SVYLDLADEV KIAWKIQRDM AERGHSLENI LASIESRKPD FAQFVDPQKR DCDVVMEILP
42Porphyrid       SVYMDVSDEV KLSWKVQRDM AERGHTLENI LASIESRKPD FAQFVDPQKK KADIVLNILP
07Thalassi        SLYLDISPDV KLNWKIQRDM EERGHSLESI MASIEARKPD FDAFIEPQKK FADYVIEVLP
05Odontella       SLYLDISDEV KLNWKIQRDM EERGHSLESI LASIEARKPD FDAYIAPQKE FADLTIEVLP
06Phaeodact       SLYLDISDDV KLNWKVQRDM EERGHSMESI LASIEARKPD FDAYIDPQKQ LADLIIEVLP
08Vaucheria       TIYLDISDEI KFAWKIQRDM MERGHSLESI QASIEARKPD FDAYIAPQRA QADVVLQVLP
09Ectocarpus      TIYLDISDEI KFAWKIQRDM MERGHSLESI QASIEARKPD FDAFIAPQRS EADVVIQVLP
43Galdieria       TVYLDISDEV KVAWKIQRDM AERGHKLENI LASIESRKPD FQQYIDPQKK DAVAVIQVLP
13Microm_p        KIYLDISDDV KFAWKIQRDM AERGHSLESI KASIEARKPD FDEFVDPQKQ YSDVIIQVLP
14Microm_stn      KIYLDISDDV KFAWKIQRDM AERGHSLESI KASIEARKPD FDEFVDPQKQ YADIVIQVLP
15Ostreoc_l       KIYLDISDDV KFAWKIQRDM AERGHSLESI KASIEARKPD FDAFVDPQKE HADVVIEVLP
16Ostreoc_t       KIYLDISDEV KFAWKIQRDM AERGHSLESI KASIEARKPD FDAFVDPQKE FSDVIIQVLP
17Chlorella       KIYLDISDEV KFAWKIQRDM KERGHSLESI KASIEARRPD FDAYIDPQKK HADVIIQVLP
18Chlorel_sp      RIYLDISDEI KFAWKIQRDM AERGHSLESI KASIEARKPD FDAYIDPQKK KADMIIQVLP
19Chlamydom       KIYLDISDDI KFAWKIQRDM AERGHSLESI KSSIAARKPD FDAYIDPQKK DADMIIQVLP
20Volvox          KIYLDISDDI KFAWKIQRDM AERGHSLQAI KSSIEARKPD FDAYIDPQKK DADMIIQVLP
27Oryza_s2        SIYLDISSDI KFAWKIQRDM AERGHSLESI KASIEARKPD FDAYIDPQKQ YADAVIEVLP
34Closterium      SIYLDISDEV KFAWKIQRDM AERGHSLESI KASIQARKPD FDAYIDPQKQ HADVVIQVLP
26Arabidopsi      SIYLDISNEV KFAWKIQRDM AERGHSLESI KASIEARKPD FDAFIDPQKQ YADAVIEVLP
24Zea_mays        SIYLDISDEV KFAWKIQRDM AERGHSLESI KASIEARKPD FDAYIDPQKQ YADAVIEVLP
23Oryza_s1        SIYLDISDEV KFAWKIQRDM AERGHSLESI KASIEARKPD FDAFIDPQKQ YADAVIEVLP
25Triticum        SIYLDISNEV KFAWKIQRDM AERGHSLESI KASIEARKPD FDAFIGPQKQ YADAVIEVLP
31Selaginel       SIYLDISDAV KFAWKIQRDM AERGHSLESI KASIAARKPD FDAYIDTQKQ YADVVIQVLP
29Spinacia        SIYLDISNEV KFAWKIQRDM KERGHSLESI KASIESRKPD FDAYIDPQKQ HADVVIEVLP
30Beta_vu         SLYLDISNEV KFAWKIQRDM KERGHSLESI KASIEARKPD FDAYIDPQKQ YADVVIEVLP
22Populus         SIYLDISNEV KFAWKIQRDM AERGHSLESI KASIEARKPD FDAYIDPQKQ YADAVIEVLP
28Pisum_sa        SIYLDISNEV KFAWKIQRDM AERGHSLESI KASIEARKPD FEAYIDPQKQ YADAVIEVLP
```

```
21Arteminis       SIYLDISDDV KFAWKIQRDM AERGHSLESI KASIEARKPD FDAYIDPQKQ YADAVIEVLP
32Physcomi        SIYLDISDDV KFAWKIQRDM AERGHSLESI KASIASRKPD FDAYIDPQKQ YADVVIQVLP
33Chara_braunii   SVYLDISDDV KFAWKIQRDM AERGHSLESI KASIAARKPD FDAYIDTQKQ YADVVIQVLP
36Gloeochaete     SIYLDLSDEV KLAWKVQRDM AERGHRLEDI WASIESGKPD FSAYFDPQKE NADVMAQLLR
37Cyanophora      SIYLDLSDEI KLAWKVQRDM AERGH----- ---------- ---------- ----------
35Glaucocyst      TVYLDLSDEV KLAWKIQRDM AERGHQLEDI LRSIEARKPD FAAYIDPQKQ YADVVMQILP
47Synechoco       SVYLDIDDEV KIAWKIQRDM AERGHSYEDV LASIEARRPD FKAYIEPQRG HADIVIRVMP
54Nostoc_pun      SVYFDISDEV KIAWKIQRDM AERGHRYEDV LAQINSRKPD FEKFIEPQRE FADVVLQVLP
55Nodularia       SVYFDISDQV KIAWKIQRDM AERGHRYEDV LAQINSRKPD FEKFIEPQRE FADVVLQVLP
52Nostoc_sp       SVYFDISDEV KIAWKIQRDM AERGHRYEDV LAAINSRKPD FQKYIEPQRE FADVVLQVLP
53Anabaena_v      SVYFDISDEV KIAWKIQRDM AERGHRYEDV LAAINSRKPD FQKYIEPQRE FADVVLQVLP
56Synech2         SVYLDISDEV KINWKIQRDM AERGHTYEDV LAAINARKPD FEAYIDVQKQ YADVVIQILP
46Microcys        SVYLDISDEV KVNWKIQRDM AERGHTYDDV MAAINSRKPD FSAYIDPQRQ YADVVIQVLP
48Microco_ch      GVYLDISDEV KINWKIQRDM AERGHTYEDI LASINARRPD FSAYIEPQKE FADVVIQILP
45Cyanothe        GVYLDISDEV KINWKIQRDM AERGHSYDDV LASINARKPD FTAYIEPQKE HADVVIQVLP
49Synech1         GVYLDISEEV KINWKIQRDM AERGHTYEDI LASINARKPD FTAYIEPQKQ YADVVIQVLP
57Trichodes       SVYLDIGDEV KIAWKIKRDL AERGHRYEDV LASINARRPD FESYIDPQKV HADVVIQVLP
58Lyngbya         SVYLDISDEV KIAWKIQRDM AERGHRYEDV LASINARRPD FEAYIDTQKQ YANVVIQILP
50Thermosyn       SVYLDISDDV KIAWKIKRDM AERGHSYEDV IASINARRPD FMAYIDPQKQ YADVVLQILP
51Acaryochl       SVYLDISDEV KIAWKIQRDM AERGHTYEDV LASINARRPD FEAYIDPQKQ HADVVIQILP

                 181
01Lth_prk         TKLDPGEETK FLCTRLIQKE NTHGIRPVYL FEECAKVDWD PCPNSMACPY PGTRVRYYEE
40Gymnochl        TKLDAGEETK FLCTRLIQKE NSHGVRPVYM FEEGANVDWD PCPGTMVCPY PGTRVRYYSE
38Chlorarac       TKLDPGEETK FLNTRLIQKE NTHGVRPVFM FEEGANVDWD PCPGAMACPY PGTRVRYYGE
39Bigelowi        TKLDPGEETK YLNTRLIQRE NQHGIRPVYM FEEGSTVDWD PCPGAMACPY PGTRVRYYNE
10Prymnesi        SDLIDDPTGK FLKVRLITKN NLKHISPAYL MDEGASITWK PNPNKLTTSA PGVLFKSYQD
11Emiliani        SDLIEDPTGK FLKVKYIQKK SVTCAETPYL FDEGSELTWV PNGDKLTTSP PGVVIKSYDD
02Eut             SDIAPNGPVK FLKGRFIQKG GTKNFDPVYL VEKGSSITWK PCGDSLQCEY PGVQFAYYPE
03Euglena         SRLAETAPLE YLRVRLIQKT TTKHFDPVYL IEKGSSVTWK PCGDNLQCEY PGLQLAYYTE
04Guillardia      TNLV--NDKT HLNVKLIQCK GVDHYAPTYL WDEGSDIEWV PPRNKLASSA PGLKIYQKTE
41Cschyzon        TELIEDKERK ILKVRLLQRE GVQGFKTAYL FDEGSTIEWI PCGRRLTCSY PGIKFHYGPE
12Pavlova         TQLVNDAEGK FLRVRFIQKA GLDLIKAPFL FDEGSTIEWT PCGKKLTCAY PGIKFRYGTE
44Chondrus        TRLIDDDEKK VLRVRLLQDE NSENFDPIFL YDEGSTIDWV PCGRKLTCSY PGIKFHYGPD
42Porphyrid       TQLVDDKENK YLRVQMIQRE AATNFAKVSI FDEGSTIDWV PCGRKLSCSY PGIKFHYGPD
07Thalassi        TDL-DKEDKK TLKVRAIQKK GVADFTPTYL FDEGSEIEWA PSADKLSSPA PGIKLSYKQE
05Odontella       TQL-DEEDKK TLRVRCIQKE GVSDFSPCYL FDEGSTIAWT PAPSKLSSSG PGLTMAYGTE
06Phaeodact       TRL-DQDDKK TLRVRCIQKE GVENFDPCFL FDEGSSIEWT PAPTKLSSPA PGIKLAYYPE
08Vaucheria       TKLVEDKEGK ILRTRLIQKE NVKNFETAYL FDEGSTINWI PCGRKLTCSF PGIKFAYGPD
09Ectocarpus      TQLIGDTEGK ILRTRMVQKE GLDFFDPAFL FDEGSTISWV PCGRKLTCSF PGIKFAYGPD
```

```
43Galdieria      TRLIDDTEKK VLRVRLIQRE GIQGFQSVYL YDEGSTIDWI PCGRKLTCSY PGIKFHYGPD
13Microm_p       TQLIDDNEGK ILRVRMIMKE GVENFDAPYL FDEGSTISWI PCGRKLTCSY PGIKFFYGPD
14Microm_stn     TQLIDDNEGK ILRVRMIMKE GVENFDAPYL FDEGSTISWI PCGRKLTCSY PGIKFFYGPD
15Ostreoc_l      TQLIDDNEGK ILRVRMIMKE NVENFDAPYL FDEGSTISWI PCGRKLTCSY PGIKFFYGPD
16Ostreoc_t      TQLIDDNEGK ILRVRMIMKE GLENFDAPFL FDEGSTISWI PCGRKLTCSY PGIKFFYGPD
17Chlorella      TQLIDEKEGK VLRVRFIQKE GVNFFKPTFL FDEGSTISWI PCGRKLTCSF PGIKFFYGPD
18Chlorel_sp     TQLVDEKEGK ILRVRLIMKD GKKLFDPVYL FDQGSTVSWI PCGRKLTCSF PGIKMFYGPD
19Chlamydom      TQLVDDK-GQ YLRVRLIMKE GSKMFDPVYL FDEGSTISWI PCGRKLTCSF PGIKMFYGPD
20Volvox         TQLVDDK-GQ YLRVRLIQKE GSKMFDPVYL FDEGSTISWI PCGRKLTCSF PGIKMFYGPD
27Oryza_s2       TRLIDGDEGK VLRVKLIMKE GVEHFAPAYL FDEGSTISWI PCGRKLSCSY PGIKFSYFPD
34Closterium     TQLIDDNEGK ILRVRMIQKE GLDNFDPVYL FDEGSSISWI PCGRKLTCSY PGIKFYYGPD
26Arabidopsi     TTLIDDNEGK VLRVRLIMKE GVKYFSPVYL FDEGSTISWI PCGRKLTCSY PGIKFNYEPD
24Zea_mays       TQLINDDEGK VLRVKLIMKE GVDNFNPVYL FDEGSTISWV PCGRKLTCSY PGIKFAYGPD
23Oryza_s1       TQLIDDNEGK VLRVKLIMKE GVKNFNPVYL FDEGSSITWV PCGRKLTCSY PGIKFAYGPD
25Triticum       TQLIDDNEGK VLRVKLIMKE GIKFFNPVYL FDEGSTINWI PCGRKLTCSY PGIKFSYGPD
31Selaginel      TQLIDDNEGK VLRVRMIMKE GVDNFEPVYL FDEGSTISWI PCGRKLTCSY PGIKFFYGPD
29Spinacia       TELIDDDEGK VLRVRMIQKE GVKFFNPVYL FDEGSTISWI PCGRKLTCSY PGIKFSYGPD
30Beta_vu        TQLIDDDEGK VLRVRMIQKE GVKFFNPVYL FDEGSTISWI PCGRKLTCSY PGIKFSYGPD
22Populus        TQLIDDNEGK VLRVKLIMKE GVEFFSPVYL FDEGSSISWI PCGRKLTCSY PGIKFSYGPD
28Pisum_sa       TQLIDDNEGK ILRVRLIQKA GVKYFSPVYL FDEGSTISWI PCGRKLTCSY PGIKFFYGPE
21Arteminis      TQLIDDNEGK VLRVKLIMKE GVKYFSPVYL FDEGSTISWI PC-------- ----------
32Physcomi       TQLIDDNEGK VLRVRMVMKE GVPFFEPVYL FDEGSTISWI PCGRKLTCSY PGIKFFYGPD
33Chara_braunii  TQLIDDNEGK ILRVRMIMKE GLENFVPAYL FDEGSTVSWV PCGRKLTCSY PGIKFFYGPD
36Gloeochaete    KVLFEDLKGK VLGGKWPQRG GGRPFDPVYL YDEGSTITWI PCGRKLTCSY PGIKFFYGPE
37Cyanophora     ---------- ---------- ---------- ---------- ---------- ----------
35Glaucocyst     TQLIDDTERK VLRVRLIQRR VFLVLTPHTF SMRAPPLTGF H-GRKLTCSY PGIKFFYGPD
47Synechoco      TQLINDTERK VLRVQLIQRE GRDGFEPAYL FDEGSTIQWT PCGRKLTCSY PGIRLAYGPG
54Nostoc_pun     TNLINDTERR VLRVRMLQRE GKEGFDPTYL FDEGSTINWT PCGRKLTCSY PGMQLYYGSD
55Nodularia      TNLINDTERK VLRVRMLQRE DKEGFEPTYL FDEGSTINWT PCGRKLTCSY PGMQVYYGSD
52Nostoc_sp      TNLIDDTERK VLRVRMLQRE GKEGFEPAYL FDEGSTINWT PCGRKLTCSY PGMQLYYGSD
53Anabaena_v     TNLIDDTDRK VLRVRMLQRE GKEGFEPAYL FDEGSTINWT PCGRKLTCSY PGMQLYYGSD
56Synech2        TNLINDKENK ILRVRLIQRE GIEGFEPVYL FDEGSTINWI PCGRKLTCSY PGLRMQYGPD
46Microcys       TQLLEDHESK LLRVRLIQKE GVENFEPAYL FDEGSTIDWR PCGRKLTCAY PGIKLYYGPD
48Microco_ch     TQLIDDKECK ILRVRLVQKE GVEGFEPVYL FDEGSTIDWR PCGRKLTCGY PGIKLYYGPD
45Cyanothe       TQLIEDHESK LLRVRLVQKE GVANFEPAYL FDEGSTIDWR PCGRKLTCAY PGIKMYYGPD
49Synech1        TRLIEDKESK LLRVRLVQKE GVKFFEPAYL FDEGSTIDWR PCGRKLTCTY PGIKMYYGPD
57Trichodes      TKLINDQEHK VLRVRLIQRE GVKNFDPVYL FDEGSTINWI PCGRKLTCSY PGIKMFYGPD
58Lyngbya        TQLIDDKERK VLRIRLVQRE GIEGFEPAYL FDEGSTIYWT PCGRKLTCSY PGIKMYYGPD
50Thermosyn      SQLAEEKVGN ILRVRMLQRE GIPGFEPVYL FDEGSTITWI PCGRKLTCSY PGIRLSYGPD
51Acaryochl      TQLLEEKPGS ILRVRLIQKD GVPDFAPVYL FDEGSTINWV PCGRKLTCSY PGIRMAYGPD
```

```
                241
01Lth_prk        VAQEKNAHVI EIDGVFGEIE ELFYIEERLS NTNTKYFGEL TKQMIKNKSS PGSGDGSGLI
40Gymnochl       MNQGKHSNVL EVDGVFGETE ELFIIEERLS NTNTKYFGEL TKQMIKNKQA PGSGDGSGLI
38Chlorarac      MHGERPASVL EVDGVFGDTE ELFFIEERLS NTNTKYFGEL TKQMLKNKAA PGSGDGSGLI
39Bigelowi       MSGEKHAHVL EVDGVFGETE ELFFIEERLS NTNTKYFGEL TKQMLKNKAA PGSGDGSGLI
10Prymnesi       EWFGQSVSVL EMDGKIDSLE ELIYVESQLC NTGTKYYGEL TEQMVNNKAS PGSENGSGLF
11Emiliani       EWFGAPVSVL EMDGKVDVLD ELVYVESAIS ATGTKFYGEL TEQMIKMKDA PGSENGTGLF
02Eut            EYMGNKVEVL EMDGVISNLK EGLYVEKFLH NTAAKEFGEL TQELMKLKSS PGGDNGTGFM
03Euglena        EYMGHPAEVL EMDGVIHNLK EGLYVEKFLH NTGAKEFGEL TQELLKGQNS PGGDNGTGFM
04Guillardia     KWAGKDAAVI GMDGKYDKID EMMYVEKQFA STGSKFVGEI TKKMLEYEGQ PGSNDGTGFL
41Cschyzon       SFYGADFSTI EVDGEFAKLE ELVYIESHLS NTGTKYYGEL TQLMLAAQNT PGAYNGTALF
12Pavlova        MYMGSEVTVL EMDGRFDKLD ELIYVESALT NTGAKFYGEL TQQILKNKDA VGSDNGTGFF
44Chondrus       TYYDKEVAVF EVDGGFEKLD EMVYVETHIE RAGTKFFGEI TQSLLRNPTA PGSKNGTGLI
42Porphyrid      SYFGEDVTVL EMDGVVEKLD EMIYVESHLN NTETKFYGEM TQQLLKNPTA PGTFNGTGLF
07Thalassi       QYFGADVAVV EMDGTFDNIQ ELVYVESNLG NTNSKFYGEV TQAMLSLADS PGSNNGTGLM
05Odontella      DYYGKPAQVV EMDGTFDNIQ ELVYVESQLS NTSTKFYGEL TQAMLKLADA PGSNNGTGLM
06Phaeodact      EFFGKDAQVL EMDGNFDNIQ ELVYVESALS NTKTKFYGEM TQAMLALATA PGSNNGTGLM
08Vaucheria      TYFDNEVSVL EMDGQFDNLQ ELIYVEGFLS NTGTKFYGEL TQNILKLSDS PGSNNGTGFL
09Ectocarpus     TYYDNEVSVI EMDGQFDNLQ ELIYVESHLS NIGTKFYGEL TQNILKQADS PGSNNGTGFL
43Galdieria      NWYNHDVSVL EVDGNFEKLE ELIYIESHLN NTSTKFYGEI TQQLLRNSSA PGSNNGTGLF
13Microm_p       TFFGEEVSVL EMDGQFDKLE ELIYVESHLS NTSTKFYGEI TQQMLKYQNG PGSNNGTGLF
14Microm_stn     TFFGEEVSVL EMDGQFDKLE ELIYVESHLS NTSTKFYGEI TQQMLKYQNG PGSNNGTGLF
15Ostreoc_l      TYYGKEVTVL EMDGQFDKLE ELIYVESHLS NTSSKFYGEI TQQMLKYQNG PGSNNGTGFF
16Ostreoc_t      TFYGKEVTVL EMDGQFDKLE ELIYVESHLS NTSTKFYGEI TQQMLKYQNG PGSNNGTGFF
17Chlorella      TYYGEEVSIL EMDGQFDKIE ELIYVESHLS NTSAKFYGEI TQQMLKNSQF PGSTNGTGLF
18Chlorel_sp     TYYGEEVSVL EMDGQFDKLE ELIYMESHLS NTSAKFYGEI TQQMLKNSSF PGSNNGTGLF
19Chlamydom      TWYGQEVSVL EMDGQFDKLE ELIYVESHLS NTSAKFYGEI TQQMLKNSGF PGSNNGTGLF
20Volvox         TWYGQEVSVL EMDGQFDKLE ELIYVESHLS NTSAKFYGEI TQQMLKNSGF PGSNNGTGLF
27Oryza_s2       TYFGHEVSVL EMDGKFDKLD ELIYVESHLS NLSTKYYGEV TQQMLKHADF PGSNNGTGLF
34Closterium     TYFGNEVSVL EMDGQFDKLE ELIYVESHLS NTSTKFYGEV TQQMLKNSDF PGSTNGTGLF
26Arabidopsi     SYFDHEVSVL EMDGQFDRLD ELIYVESHLS NLSTKFYGEV TQQMLKHADF PGSNNGTGLF
24Zea_mays       IYFGNEVSVL EMDGQFDRLD ELIYVESHLS NLSTKFYGEV TQQMLKHADF PGSNNGTGLF
23Oryza_s1       TYFGHEVSVL EMDGQFDRLD ELIYVESHLS NLSTKFYGEV TQQMLKHADF PGSNNGTGLF
25Triticum       TYFGQEVSVL EMDGQFDRLD ELIYVESHLS NLSTKFYGEV TQQMLKHADF PGSNNGTGLF
31Selaginel      TYYDNEVSVL EMDGQFDKLD ELIYVESHLS NISTRFYGEI TQQMLKHADF PGSNNGTGLF
29Spinacia       TFYGNEVTVV EMDGMFDRLD ELIYVESHLS NLSTKFYGEV TQQMLKHQNF PGSNNGTGFF
30Beta_vu        TYYGNEVTVL EMDGIFDRLD GLIYV----- ---------- ---------- ----------
22Populus        AYYGHEVSVL EMDGQFDRLD ELIYVESHLS NISTKFYGEV TQQMLKHADF PGSNNGTGLF
28Pisum_sa       TYKGNEVSVV EMDGQFDRLD ELIYVESHLS NLSSKFYGEV TQQMLKHADF PGSNNGTGLF
```

```
21Arteminis      ---------- ---------- ---------- ---------- ---------- ----------
32Physcomi       TYYGNEVSVL EMDGQFDKLD ELIYVESHLS NISTKFYGEI TQQMLKHADF PGSNNGTGLF
33Chara_braunii  TYYGQEVSVL EMDGQFDKLE ELIYVESHLS NTSTKFYGEI TQQMLRHADF PGSNNGTGLF
36Gloeochaete    TSYGREVSVL EMDGGFEKLE ELIYFESLLN NTNTKYYGEF TQQLLKNQSF PGSTNGTGLF
37Cyanophora     ---------- ---------- ---------- ---------- ---------- ----------
35Glaucocyst     TWYGNEVSVL EMDGSFDKLE ELIYVESHLN NTSTKYYGEL TQQILKNQSF PGSGNGTGLF
47Synechoco      TYYGHGVSVL EVDGQFENLE EMIYVEGHLS KTDTQYYGEL THLLPQHKDY PGSNNGTGLF
54Nostoc_pun     VYYGRYVSVL EVDGQFDNLE EVIYIETHLS NTSTKYQGEL THLLLQHREY PGSNNGTGFF
55Nodularia      VYYGRYVSVL EVDGQFDNLD EVIYVETHLS KTSTKYEGEM THLLLQHREY PGSNNGTGLF
52Nostoc_sp      VYYGRYVSVL EVDGQFDNLE EVIYIETHLS NTSTKYQGEL TQLLLQHREY PGSNNGTGFF
53Anabaena_v     VYYGRYVSVL EVDGQFDNLE EVIYIETHLS NTSTKYQGEL TQLLLQHREY PGSNNGTGFF
56Synech2        SYFGNEVSVL ELDGEFDRLE EVIYVESHLS NTSTNHYGEM TELLLNHRDY PGSNNGSGLF
46Microcys       GFLGNEVSIL ELDGQFDNLE EMIYIESHLS KTGTKYYGEM TELLLKHKDY PGSTNGTGLF
48Microco_ch     SYFGHDVSVL EVDGQFDNLE EMIYIESHLS NTSTKYYGEM TELLLKHKDY PGSNNGSGLF
45Cyanothe       NFMGNEVSIL EVDGQFDNLE EMIYIESHLS KTGTKYYGEM TELLLQHKDY PGSTNGTGLF
49Synech1        NFMGNEVSLL EVDGRFENLE EMVYVENHLS KTGTKYYGEM TELLLKHKDY PGTDNGTGLF
57Trichodes      SYDGNEVSIL EVDGNFDNLE EMIYVEGHLS NTSTKYYGEM THAMMKHQDY PGSNNGSGLF
58Lyngbya        ASYGNEVSIL EVDGKFDNLD EMIYVEGHLS NISTKYYGEL THMMREHQDY PGSNDGSGLF
50Thermosyn      EYYGHPVSVL EVDGRFEKLD ELIYIESHLS NTSTKHYGEV TELLLKHRDY PGSDNGSGLF
51Acaryochl      DYYGNAVSVL EVDGQFEKLD EVIYIEGHLS NTSTKFEGEM TELLLKHRDY PGSNNGSGLF

               301
01Lth_prk        QALVALKMRE SYERFGR
40Gymnochl       QALVALKMRE SYERFGR
38Chlorarac      QALVALKMRE SYERFGR
39Bigelowi       QALVALKMRE SYERFGR
10Prymnesi       QTICAFKIRE AFEAIA-
11Emiliani       QSICAFKIRE AYESLK-
02Eut            QTLAALKIRE IYERTGE
03Euglena        QTLAALKIRE IYERAGE
04Guillardia     QTITALKVRE VYEGIKV
41Cschyzon       QTIIALKIRE IYEQLGR
12Pavlova        QTLCSFKIRE AYEKAGK
44Chondrus       QVIMAMMMRS VYEKVGK
42Porphyrid      QVLIALKMRE VYEKCGR
07Thalassi       QTLAAFAIRE LYNKKAA
05Odontella      QTLAAFAIRE LYEKKAA
06Phaeodact      QTLAAFAIRD IYEKKAA
08Vaucheria      QTVIALKLRE IYEKITS
09Ectocarpus     QTLVALKLRE VYERIKK
```

```
43Galdieria      QVLTALKMRQ LYEELGK
13Microm_p       QTLCGLKVRE LYERIEK
14Microm_stn     QTLCGLKVRE LYERIEK
15Ostreoc_l      QTIVGLKVRE VYERIGK
16Ostreoc_t      QTIVGLKVRE VYERIEK
17Chlorella      QTIVGLKIRE VYEAVDT
18Chlorel_sp     QTIVGLKCRE VYERIQK
19Chlamydom      QTIVGLKVRE VYERIKK
20Volvox         QTIVGLKVRE VYERIKK
27Oryza_s2       QTIVGLKIRD LYEQVAE
34Closterium     QTMVGLKLRE AYERISK
26Arabidopsi     QTIVGLKIRD LYEQLAN
24Zea_mays       QTIVGLKIRD LYEQIAE
23Oryza_s1       QTIVGLKIRD LYEQIAE
25Triticum       QTIVGLKIRD LYEQIAE
31Selaginel      QTIVGLKIRD VFERITS
29Spinacia       QTIIGLKIRD LFEQLAS
30Beta_vu        ---------- -------
22Populus        QTIVGLKIRD LFEQIAS
28Pisum_sa       QTIVGLKIRD LFEQIAS
21Arteminis      ---------- -------
32Physcomi       QTICGLKIRE VYERIEK
33Chara_braunii  QTIVGLKIRE VYERIAK
36Gloeochaete    RGLLGFKIRK LYEKF--
37Cyanophora     ---------- -------
35Glaucocyst     QVLLGLKIRK IYEKL--
47Synechoco      QVLTGLKMRA AYERLSQ
54Nostoc_pun     QVLTGLKMRA AYERLAT
55Nodularia      QVLTGLKMRA AYERLTK
52Nostoc_sp      QVLTGLKMRA AYERLTK
53Anabaena_v     QVLTGLKMRA AYERLAK
56Synech2        QVLVGLKMRA TYEKLTA
46Microcys       QVLVGLKMRE TYEKLAA
48Microco_ch     QILVGLKMRS TYERLTS
45Cyanothe       QVLVGLKMRE TYEKLTA
49Synech1        QVLVGLKMRE VYEQLTA
57Trichodes      QVLVGLKMRA TYESLAT
58Lyngbya        QVLVGLKMRA TYERLGS
50Thermosyn      QVLTGLKMRA TYERLSR
51Acaryochl      QVIAGLKMRA TYEKLAT
```

Alignment: SBP

```
Gymnochlora       veeflsnsep klqkavmamf nackeigyki rtascdkqac fnafgdeqla idvladnvif
Thalassiosira     ---------- ---------- ---------- ---------- -NSSGDEQLH LDIDCDKAVF
Pinus             ---------- NLSRLLVCMG EAIRTIAFKV RTASCGATSC INTFGDEQLA VDLIANKLLF
Galdieria         ---------- ---------- ---------- ---------- ---------- ----------
Cyanophora        ---------- ---------- ---------- ---------- ---------- ----------
Guillardia        LDEVLNMKDE GVRQVTREML DAAVKITEAL RVNLVTVADA QNSFGDVQLG VDVIADNIMW
Prymnesium        LEEVLEMSDK DLGSVIKTMF DACGTITEAL RKELVTVAEK QESFGDVQLG VDVLADDLMW
Lingulodinium     IEEVMELKDP KLQGMVKELL AACVKITEAL RVNLVTVNDS SNTFGDTQLT VDVIADDLLW
Euglena           LEDFMKVPDP KLRIIMATMA ESGRTIDHKV TTASCGGTAC TNVFGDEQLA VDMLADKVLF
Marchantia        LEEFLFTKNI NLRQLMLSMG LAIKKISFKV RTASCGATAC VNTFGDEQLA VDMLANKVLF
Chondrus          LEDWLQEKD- -LGKCTRGIF AACKEVAYKI RTASCDKMSC FNDFGDEQLA IDVLADKVIF
Porphyra          LEEFLAGSDP KLAKCVAAMF ASCKEVAYKI RTASCDKMAC FNDFGDEQLA IDMLADKIIF
Arabidopsis       leeflatpdk glrtllmcmg ealrtiafkv rtascggtac vnsfgdeqla vdmladkllf
Spinacia          leeflattdk glirlmmcmg ealrtigfkv rtascggtqc vntfgdeqla idvladkllf
Oryza             leeflttpdk nlirllicmg eamrtisfkv rtascggtac vnsfgdeqla vdmladkllf
Triticum          leeflttpdk nlirllicmg eamrtiafkv rtascggtac vnsfgdeqla vdmladkllf
ChlamydomonasSP   laeflatpdt klrqlmtsms eatrtiahkv rtascggtac vnsfgdeqla vdmvadgilf
Bigelowiella      ieeflantep klqkaimgmf sackeigyki rtascdkqac fnafgdeqla idvladnvif
TrypanoB          itdtlrgvpc dvvgivetva gacraiaagl rndgvtaaks knnfgddvls vdvmadkiis
TrypanoC          fleelegvps svadvvaaia eacreisidl rtdtvravga nnsfgdevls vddmaekhis
Neurospora        lasllpsrds lrtsvlpsll tsiaatstal r-aaqdvags snsfgddqln vdvlaeeair
Gibberella        lktvlpdrss lresvipqll davsaigdel r-rsydvvgt enafgdeqln vdvlaeniir
Magnaporthe       ldslvparae ltgsvlpalv haigdvsall r-rahtvvgt tnafgdqqln vdlaaddvir
Zeamays           LEEFLTTPDK NLIRLLICMG EAMRTIAFKV RTASCGGTAC VNSFGDEQLA VDMLANKLLF
Physcomitrella    leeflattdk nlarllvcmg ealrtiafkv rtascgatac vntfgdeqla vdmladkllf
ChlamydoR         LAEFLVTPDP KLRQLMMSMA EATRTIAHKV RTASCAGTAC VNSFGDEQLA VDMVADKLLF
Volvox            laeflvtpdp klrqlmmsma eavrtighkv rtascagtac vnsfgdeqla vdmvadkllf
schyzon1          LHEFLAEEDK ELAKAIEALF MACKVVGYEV RTASCNKEEC VNAFGDQQLA VDLLADRTIE
schyzon2          LENFLEVKDP DLRQIVHAIA EASVSIDQVL RSSSAVAPGQ QHNFGDAQLS EDLTADHLAR
Emiliania         ---------- ---------- ---------- ---------- ---------- ----------
Phaeodactylum     ladvlgcmde tlricitdml dvcaditeal rtalvtvegn indfgdaqls vdmiadnliw
Toxoplasma        ldqllkgadq tltdlilail drcgkiasal qgtsvdkvgs vnefgdeqlt vdviaenllw
Neospora          ---------- ---------- ---------- ---------- ---------- ----------
Tetrahymena       ---------- ---------- ---------- ---------- --afgdiqle cdtksdeiif
Paramecium        mhsqhvsddp elsavcsall lafiensrvl rycsgggtnt qndfgdhqle mdvqcelnvn

                  enlkasgava tasseeepte vnmggdgysv afdpldgssi idtnfavgti fgvwpgsklt
                  HAIREAGVFA VAASEETPEE TDVLGPWYSV GFDPLDGSSV IDANFSVGST FGLWPGKGLL
```

```
ESLRYSHFCK YACSEENPEP EDMGGPGFSV AFDPLDGSSI VDTNFTVGTI FGVWPGDKLT
---------- ---------- ---------- ---------- ---------- ----------
---------- ---------- ---TRGKYSV AFDPLDGSSI IDTNFAVGTI FGVWPGKDLV
DAAKASKVVK EAASEEEPVL VETNNGRFTI CWDPLDGSSI VDNNWAVGTM IGIWDKKTML
EVCKTDPLIK EGASEEEPEV REMHDGKFCI CWDPLDGSSI VDNNWAVGTI LGIWGAETIL
DLAKSSQLVC EASSEEEPEI VKTNDGQYVL CWDPLDGSSI VDNNWAVGTI VGVWDKSTLL
EGLRHCGVCE IACSEENPVP LPMGDPGYSV CFDPLDGSSI VDTNFAVGTI FGVWPGNKII
EALLHSHVCK YACSEEEPNL VDMGGPGFSV AFDPLDGSSI VDTNFTVGTI FGVWPGDKLT
DNLTESGVVA VASSEELPIE KPITGGKFAV AFDPLDGSSI IDTNFSVGTI FGVWKGDRFV
DNLEASGVVA TASSEEVPKE VTITGAPYSV AFDPLDGSSI IDTNFTVGTI FGVWPGDKLV
ealryshvck yacseevpel qdmggpgfsv afdpldgssi vdtnftvgti fgvwpgdklt
ealnyshfck yacseelpel qdmggpgfsv afdpldgssi vdtnfsvgti fgvwpgdklt
ealeyshvck yacseevpel qdmggpgfsv afdpldgssi vdtnftvgti fgvwpgdklt
ealeyshvck yacseevpel qdmggpgfsv afdpldgssi vdtnftvgti fgvwpgdklt
ealkyshvcs yacseevpep vamggsgysv afdpldgssi vdtnfsvgti fgvwpgdklt
enlrasgava tasseeepte vplggegysv afdpldgssi idtnfavgti fgnlgldplt
ealnscqhva syvseespsl astasgthsv sydpldgssi itsnftvgsi favwpgntpi
scltgcrhvl afvseerptl tstprgmytv sydpldgssi iatnfsvgsi falwpgatpi
lclaqcpsvv tasseedpie kpvqaevytv afdpldgssi iapnwtvgti fslwdgtsal
drcakspaik tasseedpve kpardeqyti gfdpldgssi igpnwsvgsi igiwdgvtai
aackscpsvv tasseevpie ealragrytv aydpldgssi iapnwtvgai lgvwdgatal
EALEYSHVCK YACSEEVPEL QDMGGPGFSV AFDPLDGSSI VDTNFTVGTI FGVWPGDKLT
ealrhshvck yacseeepil qdmegegfsv afdpldgssi vdtnftvgti fgvwpgdklt
EALKYSHVCK LACSEEVPEP VDMGGEGFCV AFDPLDGSSI VDTNFAVGTI FGVWPGDKLT
ealkyshvck lacseevpep vdmggegfcv afdpldgssi vdtnfsvgti fgvwpgdklv
AALRASRVVA IGSSEEQPIE KDLGGSTFAV AWDPLDGSSI VDTNFSVGTI FGVWRGKRLT
SRLLQVPCVS YVSSEENPTE QRGPGHKYAV AFDPLDGSSV LESNFSVGSI FGVWRTAELV
---------- ---------- ---------- ---------- ----WAVGTI LGIWGAETIL
davkrstvir egaseedpvv rnvdnkeytv cwdpldgssi vdnnwavgtm igvwpkstll
aqssegsavr avcseedihl qechngefil cwdpldgssi idcnwavgsi vsiwrqgali
---------- ---------- ---------- ---------- ---------- ----------
nhlkktgeva yglseeqpkl velggnkyiv tfdpldgssi igcnwtvgti fgiwkndeli
telkktgfvs hsaseetpem kllsggkfiv tfdpldgssi igtnfavgti vaiwksdeli

gikgteqkaa glgvygprtt vtlavddgah efiliddgqw vlsqsfptig egklfapgnl
GRTGREQVAS VVSVYGPRTT LIIALPDVTF EVTLVNDSHW EVSRDKCTLA PKKVFAPGNL
GVTGRDQ--- ---------- ---------- ---------- ---------- ----------
---GRDMAAA ALVVYGPRTT ISLALKDHVH EFTLVDDGQW VHTNEFHSVE EGKLFAPGNL
GVTGAQQVAA GLGIYGPRTQ FMLSVKNGTH EFTLEQDGSW VHTKEIYEIA EGKLFAPGNL
GATGRDQVTS IVVLYGPRTT ALVACDDGVY EFTCGAGNKW IASREKIQIK KSKIFSPANL
```

```
GATGRDQKAS MVALYGPRTT VFVTLDDGVY EFTYANGDGW LCSRERCEIA PSKIFAPANM
GATGRDQVMS LVALYGPRTT VFMTMDDGVY EFTLGPGNQW ICSREKIEIK KCKIFAPANM
GSTGRDLAAS GICVYGPRTV LCVAFKPGTH DFLLGDDGKW TYVKAYTHIG EGKLFAPGNL
GVTGREQVAA AMGIYGPRTT YVLCLAPGTH EFLLMDDGTW QHVKETTTIG EGKLFSPGNL
GQDGHGVVCA GLTIYGPRTT ITLAIDDGAH EFLLIDDGSW IHTAAFSTVN EGKLFAPGNM
GTTGRELVAG GMVLYGPRTT VTLAMDEGAH EFLLIDDGSW VHTATFTSVN EGKLYAPGNL
gitggdqvaa amgiygprtt yvlavkpgth eflllldegkw qhvketteia egkmfspgnl
gvtgrdqvaa amgiygprtt yvlalkpgth eflllldegkw qhvkettein egklfcpgnl
gvtggdqvaa amgiygprtt yiialkpgth eflllldegkw qhvkdtttig egkmfspgnl
gvtggdqvaa amgiygprtt fvvalkpgth eflllldegkw qhvkdttsig egkmfspgnl
gitgrdqaaa gmgvygprtv fviainpgth efllqddgkw llvksteeig egklfapgnl
gikgteqkaa glgvygprtt itlavdqgah eflliddgqw vlsqsfmsig esklfapgnl
gltvrdmvas vvavygprvv lfvgqelgva effcgadgew klakrtaagr gatvfspgnl
glrvrdmvas vvavygprtv lfvslrvgvv efyygdr--w trvqngvprt latlfapgnl
gasprdqiga vlgvygprtt avvalrpgee kfevlsdqew dlirpsvsya ptryfapanl
eqptekqias ilgvfgprtl aivalrpvcf evslnnshmf tvarpelrfa etryfapanl
gqsprrmvaa ilgvfgprtt aivalrpgig efelidgesl tmvqpnirla stryfapanl
GVTGGDQVAA AMGIYGPRTT YIVALKPGTH EFLLLDEGKW QHVKDTTTIG EGKMFSPGNL
gitgrdqaas amgiygprtt yvvainpgth efllmddgkw qhvketteik egklfspgnl
NITGREQVAA GMGIYGPRTV FCIALKPGCH EFLLMDDGKW MHVKETTHIG EGKMFAPGNL
nitgreqaaa gmgiygprtv fcialkpgth efllmddgkw mhvketteig egkmfapgnl
GITGRELEAA GMAVYGPRTS ISVSVRPGTH EFLLVDNGMW VLVQSFYTIN EGKLFAPGNL
GCQGRDMVAA AVTLYGPRLV LVVAVDGGVA SFRWTSNARW RLLKQWSPDL LASLFAPGNL
GATGRDQKAS LVALYGPRTT VFVTLDDGVY EFTYANGDGW LCSRDRIQIS QSKIFAPANM
gatgrdqvts lvalygprtt vlvalddgay efsygctepw ictrhkiqin hskifspanl
qktgrqqvas livvygprtt gvvavngtal dlemkdngkf ic-rgkpiik pakifspanl
-------VAS LIVVYGPRTT GVVAVNAGVI KAGSLDLGSF VCRGTPV-IA PAKIFSPANL
ghktkdlias gccmygprtt aviynektvn eysllnkvew ilslpnivik pgklfapgnl
gkrgrdmvsa ccclygsrtn vvfwneqkiq eytlfdgghw eltkdnikik pgklfspgnt

ratldnpgya elfqhwydnq yqlrytggmv pdvnqimvkg kgvfvnaask nakaklfffy
RATNDNPKYD ALVKHWISDR YTLRYTGGMV PDVYHMFAKS GGVFSNVSSD KARAKLRLLY
-----NPEYE KLINYYVSEK YTLRYTGGMV PDVNQIIVKE KGIFTNVISP TSKAKLRLLF
RAIQDNPGYS RLLQFWLNEK YQLRYTGGMV PDVNQILVKG RGVFCNPASA SAKAKLRLLY
RATMDNANYK ALVDYWLREK YQLRYTGGMV PDVNQILIKG KGVFCNPASA AAPAKLRVLY
RCCQEDAGYD ALVKHWMEKR YTLRYSGGLV PDVYQHFTKE MGVFANPTSP KSPAKLRVAF
RAAQDLPEYA ALIDYWMANK YTLRYTGGLV PDICQQFTKK MGVFANPTSE SSPAKLRLAF
RAAQEVDGYA KLIDHYMTNR YTLRYSGGLV PDVCQQFTKT QGVFTNPTSK ASPAKLRLAF
RCTLDNPEYE RLISYYTRQQ YTLRYTGGMV PDVYQILVKE KGVFTNVISP STKAKLRLSF
RATYDNPEYE KLINYYVSEK YTLRYTGGMV PDVNQIIVKE KGIFTNVISP TTKAKLRLLF
```

```
RAAQDNPGYA KLLEHYYSNK YQLRYTGGMV PDVNQILVKG KGVFCNPASP SAKAKLRVLY
RAAEDNPGYA DLIDYYRKEK FQLRYSGGMV GDVNQILVKG KGVFCNPSSP SAKAKLRLLY
ratfdnseys klidyyvkek ytlrytggmv pdvnqiivke kgiftnvtsp takaklrllf
ratsdnadya kliqyyikek ytlrytggmv pdvnqiivke kgiftnvisp takaklrllf
ratfdnpeyd klinyyvkek ytlrytggmv pdvnqiivke kgiftnvtsp takaklrllf
ratfdnpdyd klvnyyvkek ytlrytggmv pdvnqiivke kgiftnvtsp takaklrllf
raifdnpeys klvsyyleeq ytlrytggmv pdvfqilvke kgvftnvtsp stkaklrllf
ratkdnkgye elfnywydnq yqlrytggmv pdvnqimvkg kgvfvnaesk aakaklrlly
raarhlpwyk qlitmymqeg atlrytggmv pdvcqiivkg dgiymtpasp qhkmklrllf
ravmylpwyk elvtsymnsg atlrytggmv advcqiivkg dgiymtpesp hhkvklrllf
rstnthaaya klvahymaen ytlrycgglv pdvvhalvkg hgvylspvte tskaklrsly
raaaendkym slvtkfindk ytlrysggli pdvyhalvkg hgvyvspvts vskaklrkyy
raaaecdkys alvshfisqk ytlrysgglv pdvvhalvkg hgv------- ----------
RATFDNPEYD KLINYYVKEK YTLRYTGGMV PDVNQIIVKE KGIFTNVTSP TAKGQLRLLF
ratfdnadye klinyyvsek ytlrytggmv pdvnqiivke rgiftnvtsp ttkaklrllf
RATFDNPAYE RLINFYLGEK YTLRYTGGMV PDVFQIIVKE KGVFTNVTSP TTKAKLRILF
ratfdnpaye rlinfylgek ytlrytggmv pdvfqiivke kgvftnvtsp ttkaklrilf
KATQDNPGYA KLVQYWMENK YQLRYTGGMV PDCNQILIKG KGVFANPASP SAPAKLRVLY
RATQELPPYD RMVRSWIESR YTLRYSGAMA PDVFQIIAKG QGIFCNPSVP G-KAKLRLVY
RAAQDLPNYA KLIDYWMTNK YTLRYSGGLV PDICQQFTKR QGVFANPTSE SSPAKLRLAF
raaqelpgyk klvdhflenr ytlrytgglv pdvyqqftkg qgvfsnptag gspaklrlaf
raaqdlpayk qliefwmekr ytlrytgglv pdvyqifvkq qgvfcnpask aapaklrmcf
RAAQDLPAYK RLIDYWMEKR YTLRYTGGLV PDVYQIFVKQ QGVFCNPASK AAPAKLRMCF
raasenpnyr qcinnwidqg ytlrytggmv ---------- ---------- ----------
rcivdhipyr evvdywihng ytlrysggma pdicqiflke vgvfscfgda knpsklryly

eaaptgsiie kaggkffdge qsvfnlevig tearpqvayg sagevarfee
EVAAMGLLVE CAGGVTTHES ISVLDLEVDD LDRRLGVCF- ----------
EVAPLGMLVE KAGGYSSDG- ---------- ---------- ----------
ELAPTAFVVE KAGGKSSDGK QSVLDIVIQS TDQRSQVCYG SAGEVERFEE
ECAPIAKLIE GAGGKSSDGK QSVLNIKIEN TEDRSQVCYG SKGEVERFER
EIAPFSLLVE KAGGKTSDGV NSCLDIKIEA VDQRTPACLG SANEVDLFNK
EAAPFGLLVE KAGGKTSDGV GSVLDVKITG IDQRTPLCIG SAEEVDRFNT
EAAPFGRLVE MAGGKTSDGV QSVLDVKIEA VDQRTALAIG SANEVDRFNE
EAAPIALLVE KAGGASSCDG VSALDVQING IDQRTQVCFG SRTEVARFEH
EVAPLGLLVE KAGGYSSDGK ISVLDKVVVN TDDRTQVAYG SRNEIIRFEE
EVLPIGYVIE KAGGKSSNGA GSVLDIKIKS CEDRSQVCYG SADEVARFEE
EVAPIGYVME KAGAKSSDGS GSVLDIKVQT TEDRSQVCYG SAGEVARFEQ
evaplgllie naggfssdgh ksvldktiin lddrtqvayg skneiirfee
evaplgflie kagghssegt ksvldievkn lddrtqvayg slneiirfek
```

```
evaplgflie kaggyssdgk qsvldkvinn ldertqvayg skneiirfee
evaplgflie kagghssdgk qsvldkvisv ldertqvayg skneiirfee
evaplallie naggasscdg craldvevta heqrtqicyg skgevarfek
evapigyvie kaggkssdge qsvlnievvg tedrtqvayg saaevdrfeq
eaapmaflih caggrsttgl tnmmnvrvvs meqttpialg cardveryer
eaapmaflve caggrsttgt knmmdvrvve meqktpialg clwdveryes
elfplalvve ccggravdpv nilgdkgveg cderggivcg taeeveyake
ellpvalvie caggqaidpa srifditlkg cddraglvcg tseevevvkk
---------- ---ggvicgn nievdlaipe lrlksplq-- ----------
EVAPLGFLME KARRYSSDGQ QSVLDRVIN- ---------- ----------
evaplgllie naggyssdgk qsvldkvvvn tddrtqvayg srdeiirfee
EVAPLALLIE KAGGASSCDG VSALDIPILV CDQRTQICYG SIGE------
evaplallve kaggasscdg vsaldipiln cdqrtqicyg sigevrrfee
EVAPVAFLIE RAGGASSDGE RSALDIPIPG TDVRSQVCYG SKGEVARFNE
EALPLAFLVE AVGGRSSDGS TSLLDKRITL LIERTPVVLG SPSEVQRYIE
EAAPFGLLVE KAGGKTSDGV ASILDTKITG IDQRTPLCIG SASEVDRFNL
eaapfgllve kaggktsdgv gsildvqins vdqrtalclg ssdevnrfne
evlaialvve aaggrtsngq kslldvaieh mdhrsalccg sadeikrmee
EVLPIALLVE AAGGRTSNGK TSLLDVGIEH MDHRSALCCG SADEIQRMEE
---------- ---------- ---------- ---------- ----------
ecaplsflie kaegksfngk hsvldteitg yqqkseiivg sadeieffks
```
