# Supplementary material for: Phylogenomic Analysis of “Red” Genes from Two Divergent Species of the “Green” Secondary Phototrophs, the Chlorarachniophytes, Suggests Multiple Horizontal Gene Transfers from the Red Lineage before the Divergence of Extant Chlorarachniophytes
Source: PLoS One. 2014 Jun 27;9(6):e101158. doi: 10.1371/journal.pone.0101158 (PMC4074131; doi:10.1371/journal.pone.0101158)
Supplement: File S2 — Phylogenetic trees of ABC, GGR, RPS22, RNABP, PMP, HP, PRK, and SBP sequences (Figures S2–S9). The trees were inferred using the RaxML method with the WAG+I+gamma model. Numbers at branches represent support values (bootstrap values ≥50% or posterior probability ≥0.95) from RaxML/PhyloBayes. Thick branches represent RaxML and PhyloBayes support values of 100% and 1.00, respectively. Colors of taxa: dark blue-Cyanobacteria; navy blue-Glaucophyta; green-Chloroplastida; red-Rhodophyceae; pink-Cryptophyta; yellow-Haptophyta; light pink-Alveolata; orange-stramenopiles; brown-Chlorarachniophyta; purple-Euglenophyta; black-Fungi; violet-Kinetoplastida. (A) Lacking alveolate OTUs. (B) Containing alveolate OTUs. (PDF) [file pone.0101158.s005.pdf]

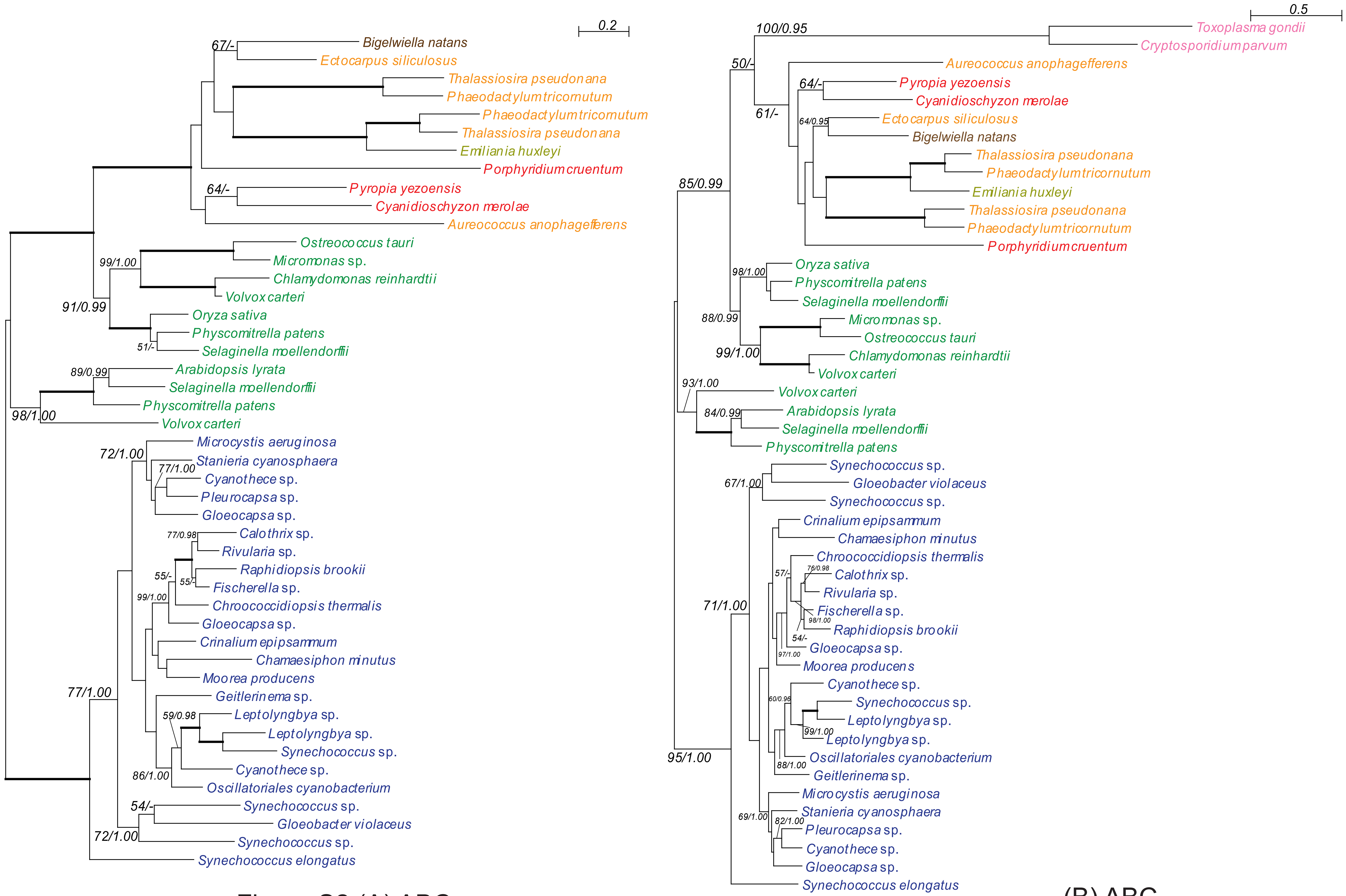

Figure S2 (A) ABC

(B) ABC

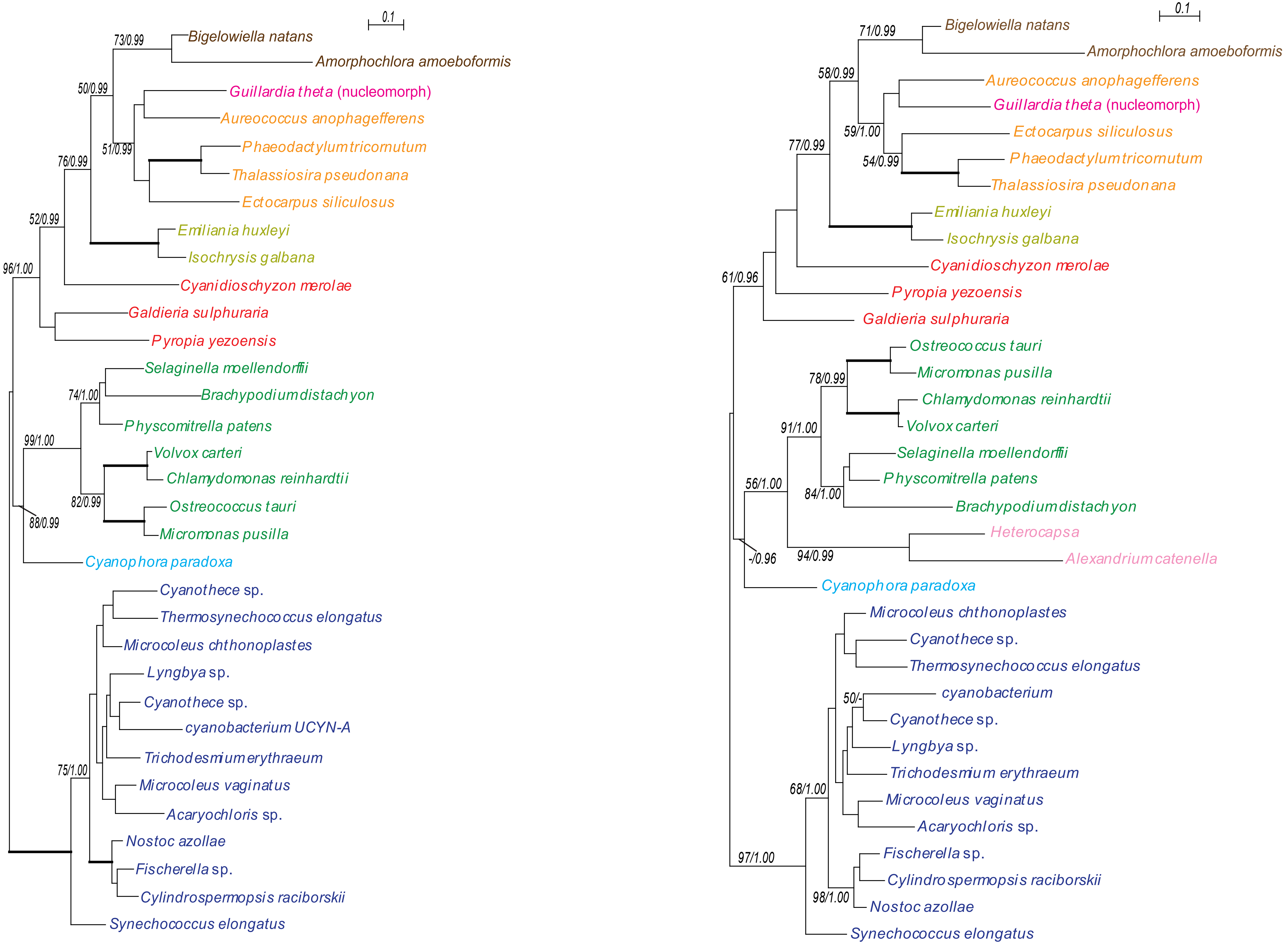

Figure S3 (A) GGR

(B) GGR

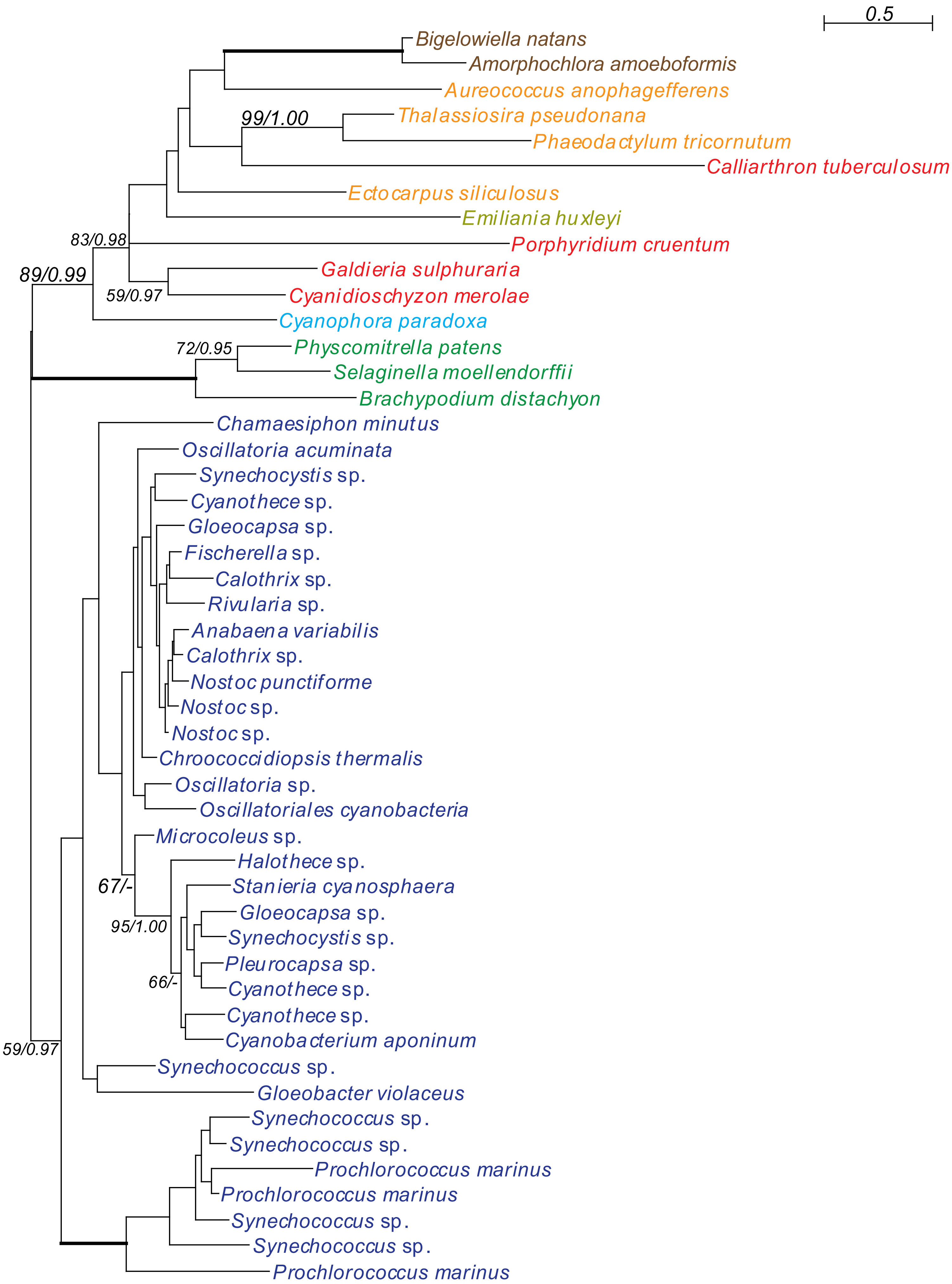

Figure S4 RPS22

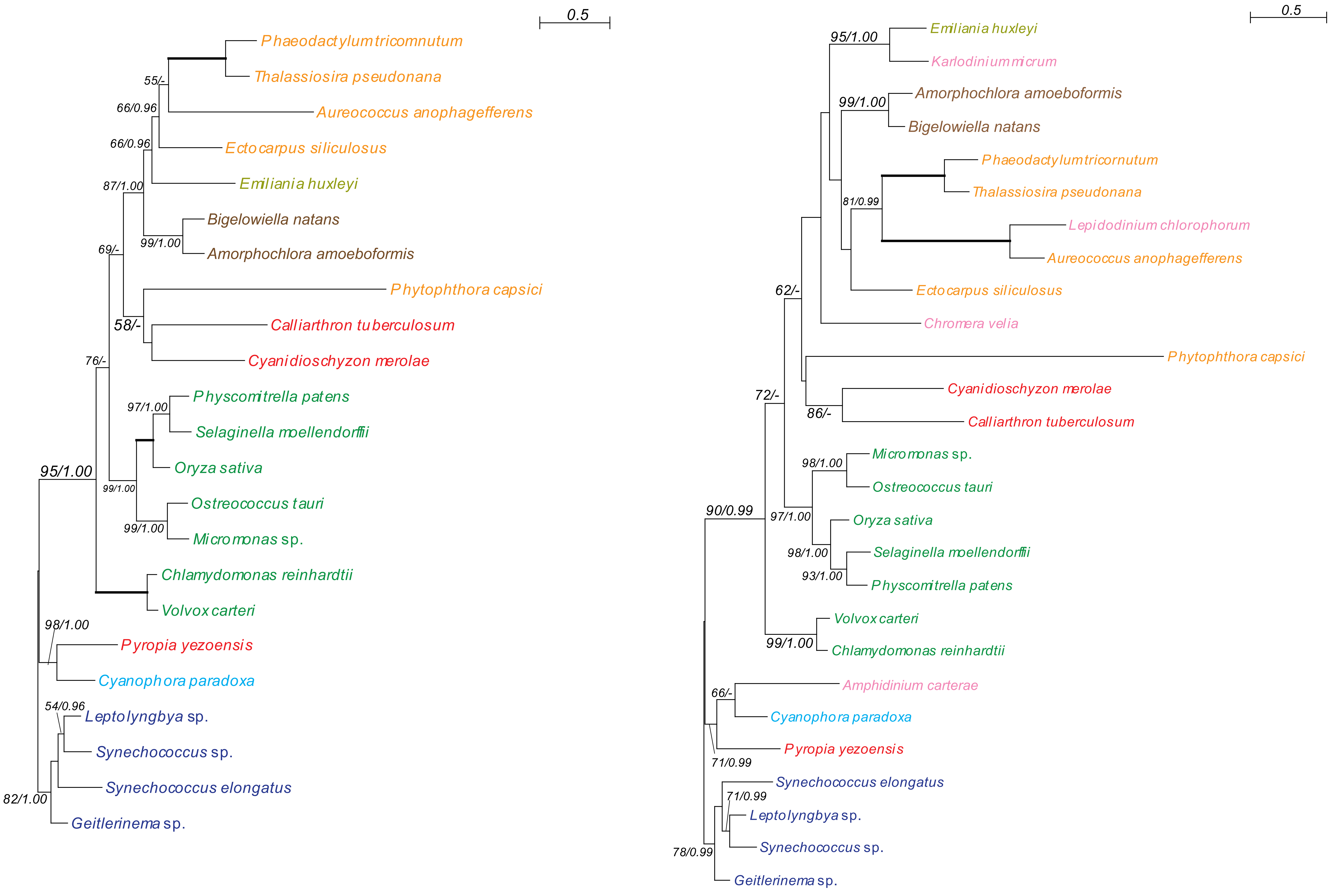

Figure S5 (A) RNABP (B) RNABP

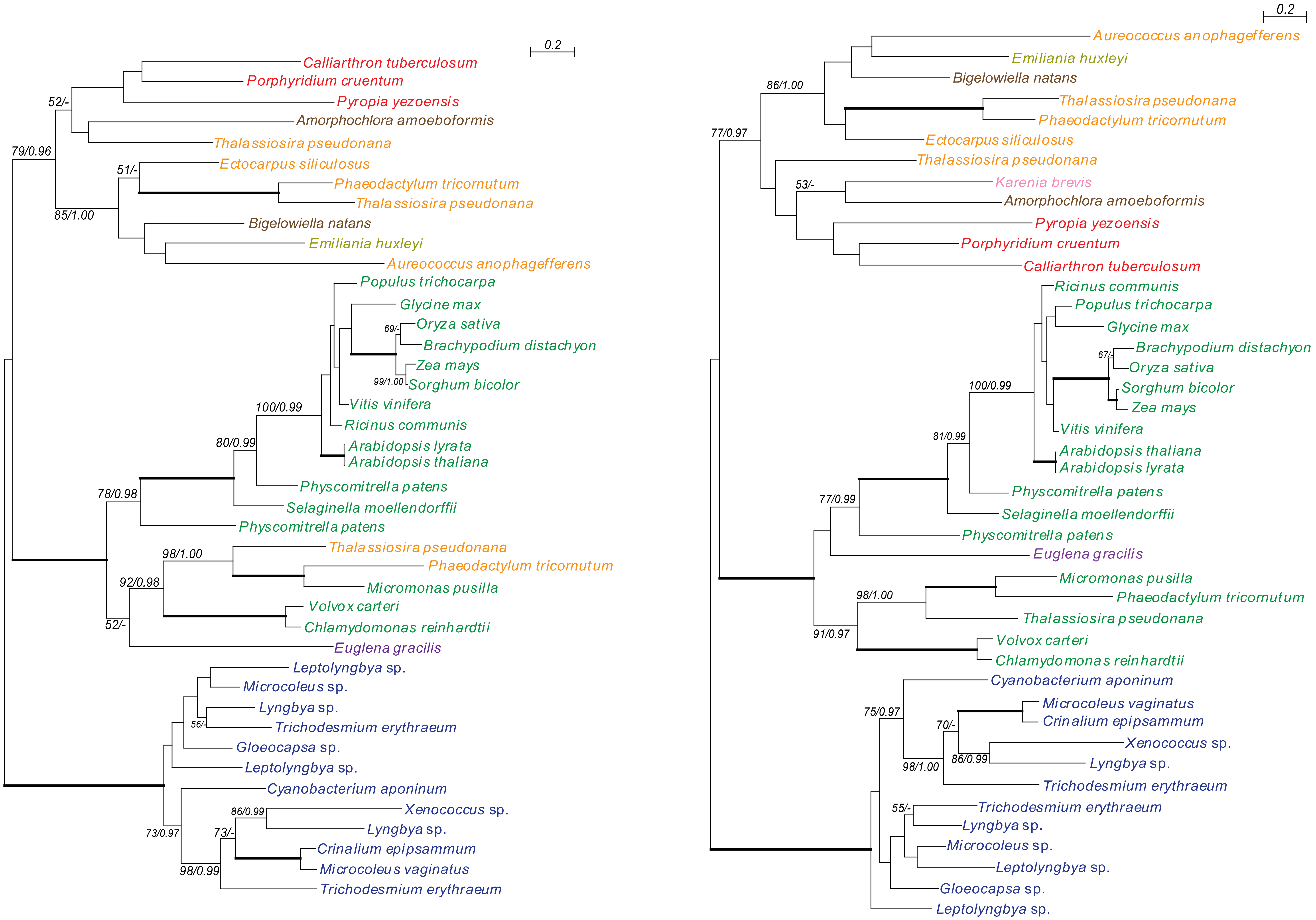

Figure S6 (A) PMP

(B) PMP

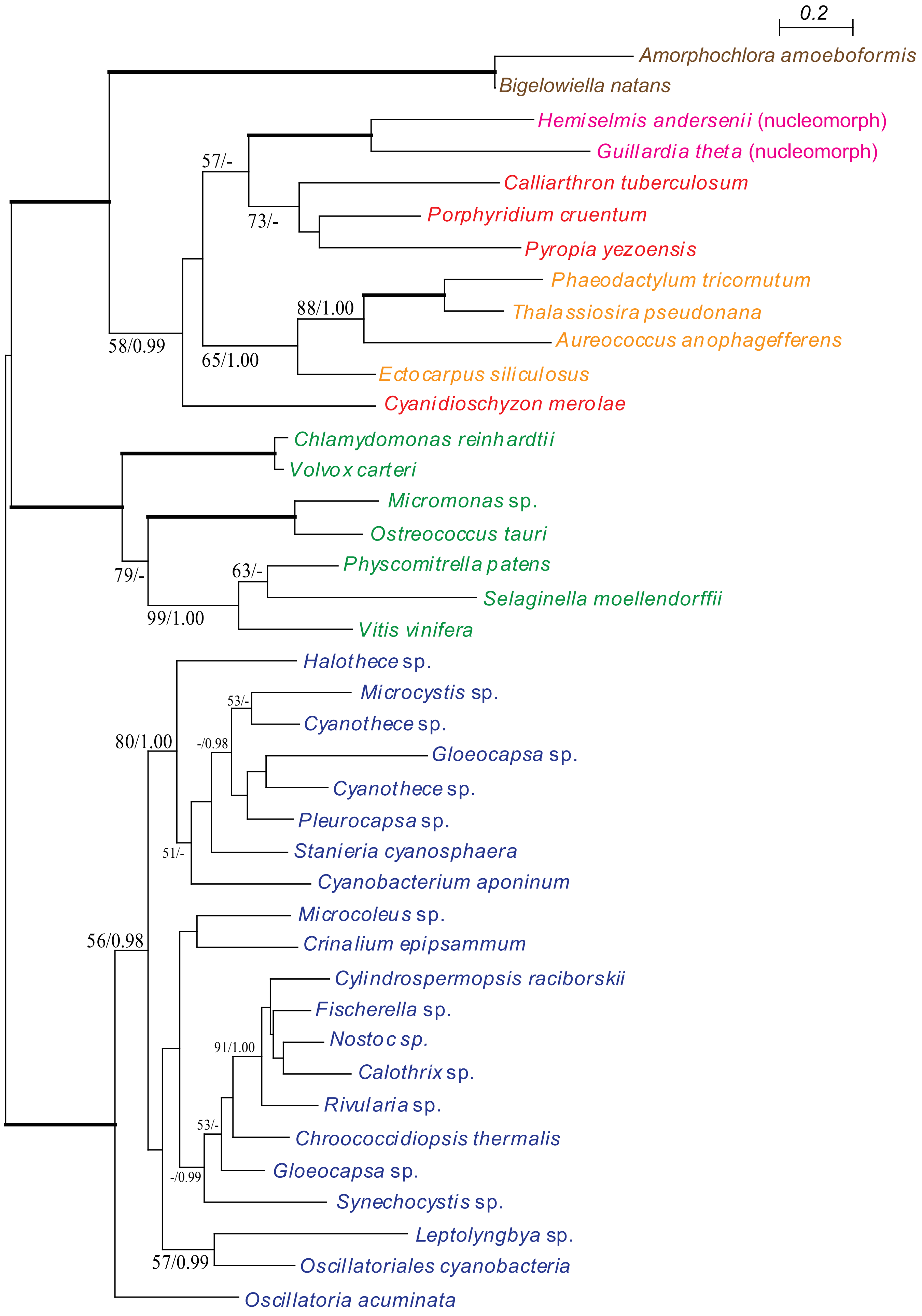
0.2
Amorphochlora amoeboformis
Bigelowiella natans
Hemiselmis andersenii (nucleomorph)
Guillardia theta (nucleomorph)
57/-
Calliarthron tuberculosum
73/-
Porphyridium cruentum
Pyropia yezoensis
Phaeodactylum tricornutum
88/1.00
Thalassiosira pseudonana
Aureococcus anophagefferens
58/0.99
65/1.00
Ectocarpus siliculosus
Cyanidioschyzon merolae
Chlamydomonas reinhardtii
Volvox carteri
Micromonas sp.
Ostreococcus tauri
79/-
63/-
Physcomitrella patens
Selaginella moellendorffii
99/1.00
Vitis vinifera
Halothece sp.
Microcystis sp.
53/-
Cyanothece sp.
80/1.00
-/0.98
Gloeocapsa sp.
Cyanothece sp.
Pleurocapsa sp.
51/-
Stanieria cyanosphaera
Cyanobacterium aponinum
Microcoleus sp.
56/0.98
Crinalium epipsammum
Cylindrospermopsis raciborskii
Fischerella sp.
Nostoc sp.
91/1.00
Calothrix sp.
Rivularia sp.
53/-
Chroococcidiopsis thermalis
Gloeocapsa sp.
-/0.99
Synechocystis sp.
Leptolyngbya sp.
57/0.99
Oscillatoriales cyanobacteria
Oscillatoria acuminata

Figure S7 HP

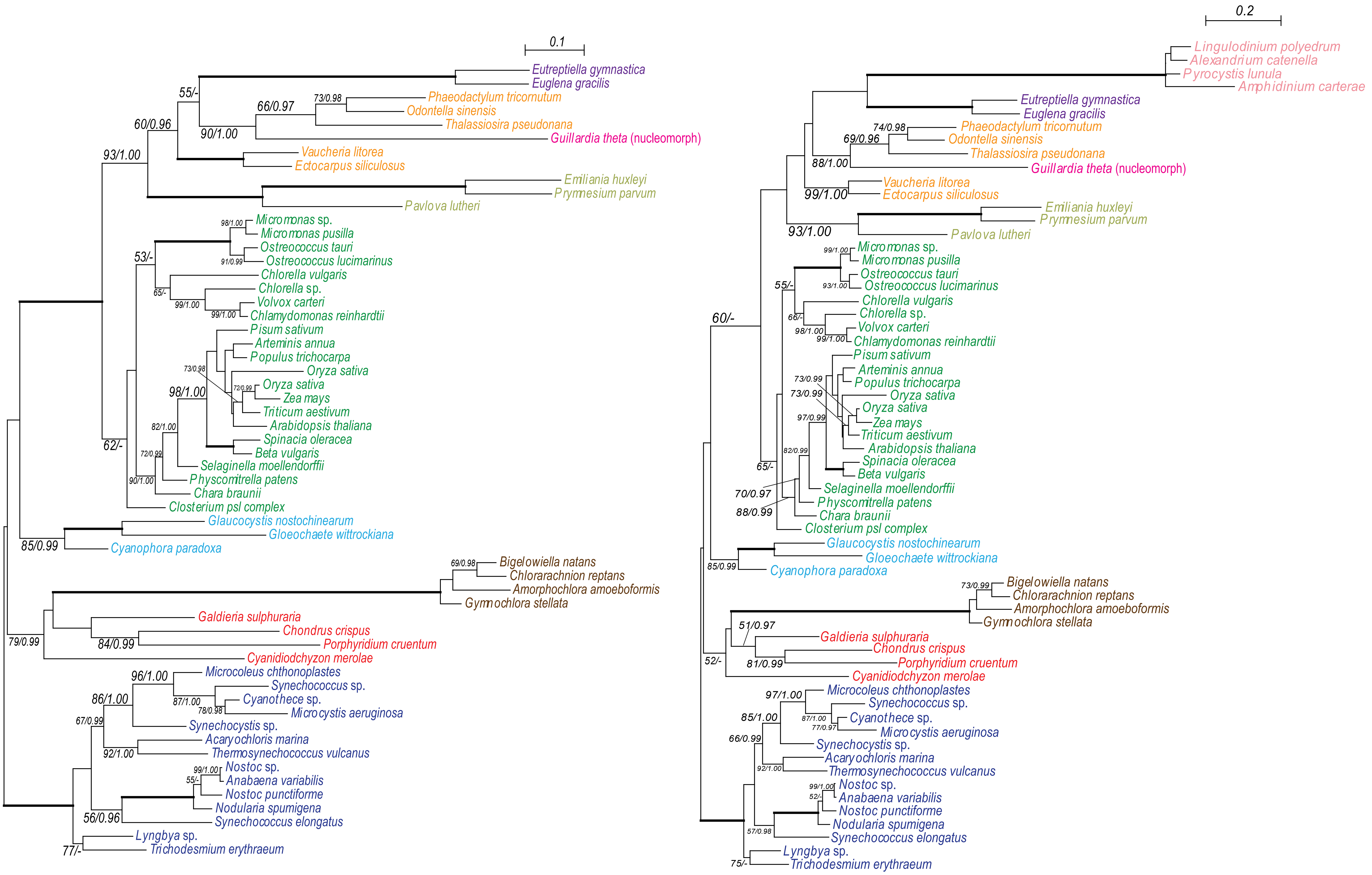

Figure S8 (A)PRK (B)PRK

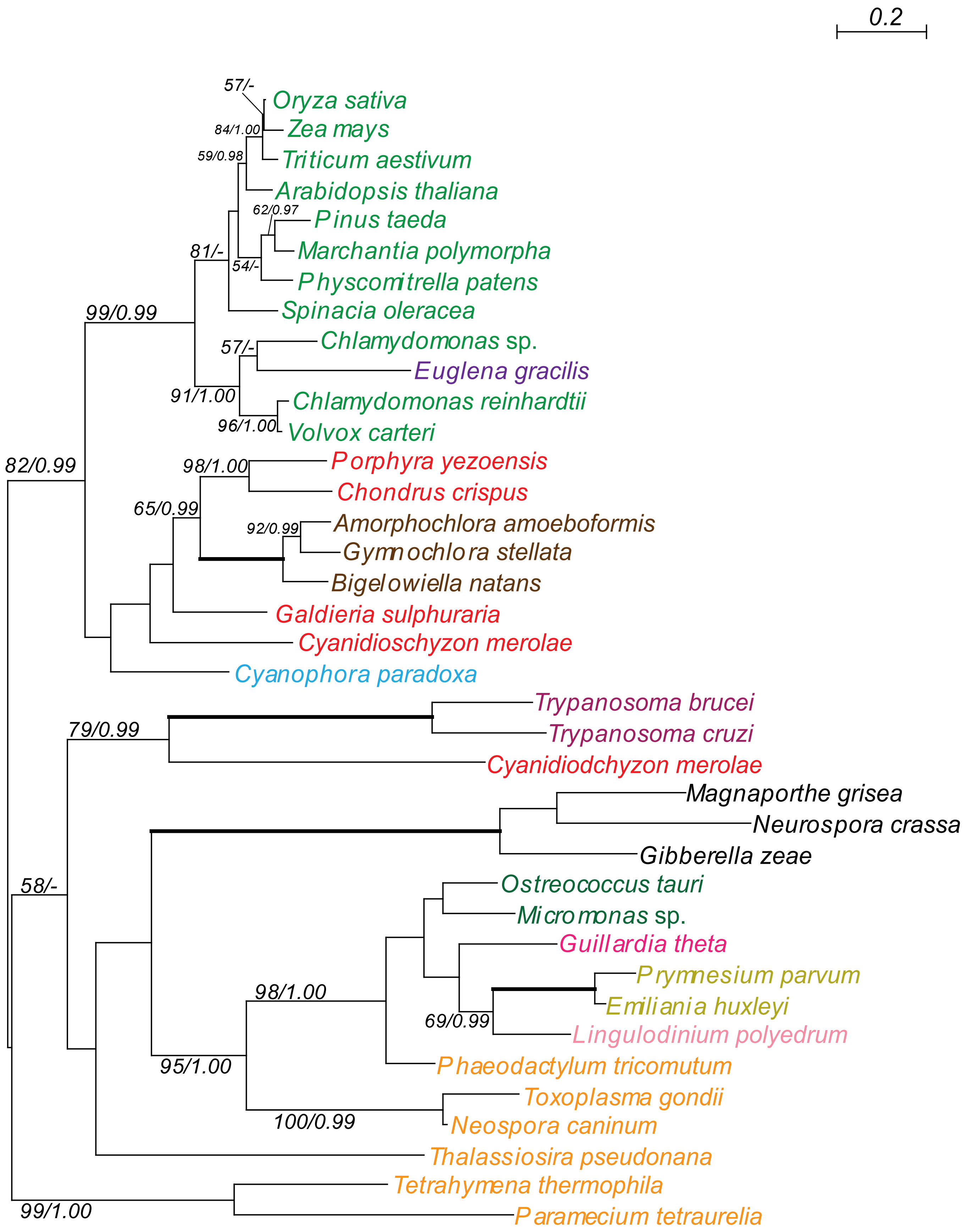

Figure S9 SBP
