# Supplementary material for: Phylogenomic Analysis of “Red” Genes from Two Divergent Species of the “Green” Secondary Phototrophs, the Chlorarachniophytes, Suggests Multiple Horizontal Gene Transfers from the Red Lineage before the Divergence of Extant Chlorarachniophytes
Source: PLoS One. 2014 Jun 27;9(6):e101158. doi: 10.1371/journal.pone.0101158 (PMC4074131; doi:10.1371/journal.pone.0101158)
Supplement: File S3 — Results of the AU tests for assessing placement of chlorarachniophyte lineage in nine trees: PDP, PS2SAF, PGK, ABC, GGR, RPS22, RNABP, HP, and PRK (Figures S10–S18). The trees were inferred using the RaxML method with the WAG+I+gamma model. Branches that were rejected by AU test (p≤0.05) for placement of chlorarachniophyte lineage are indicted with dashed lines. Colors of taxa: dark blue-Cyanobacteria; navy blue-Glaucophyta; green-Chloroplastida; red-Rhodophyceae; pink-Cryptophyta; yellow-Haptophyta; baby pink-Alveolata; orange-stramenopiles; brown-Chlorarachniophyta; purple-Euglenophyta; black-Fungi; violet-Kinetoplastida. (PDF) [file pone.0101158.s006.pdf]

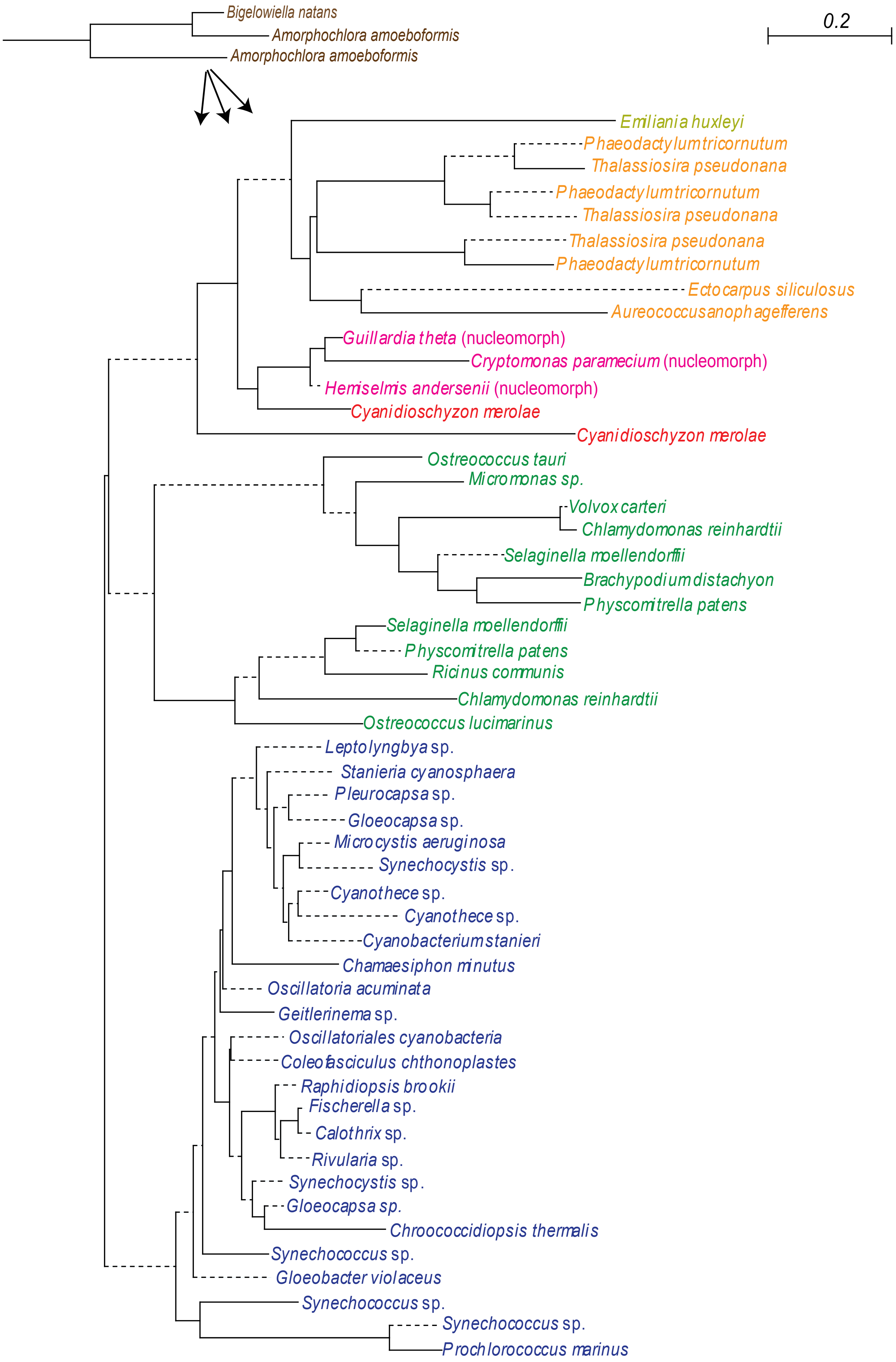

Figure S10 PDP/FtsZ

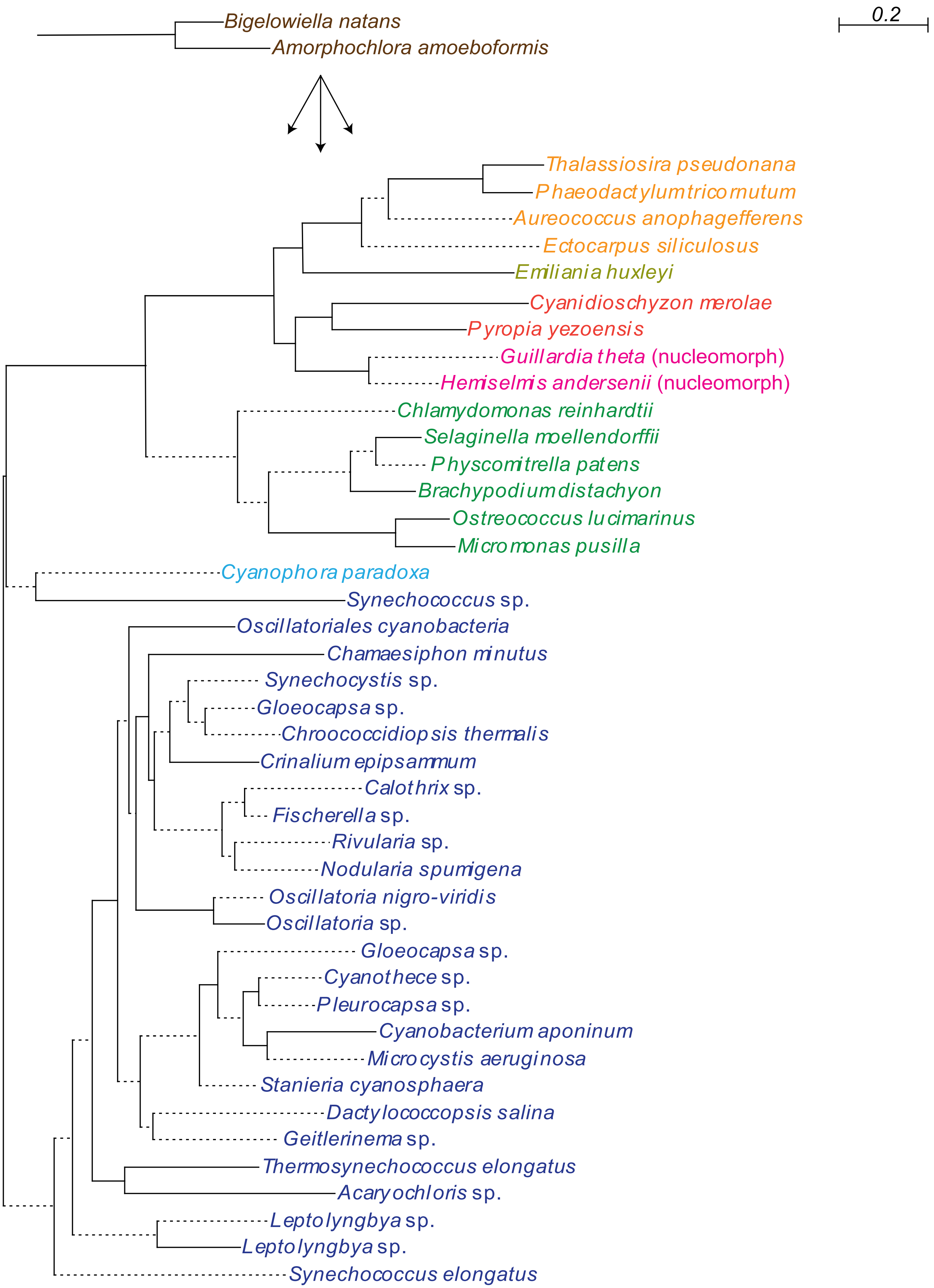

Figure S11 PS2SAF

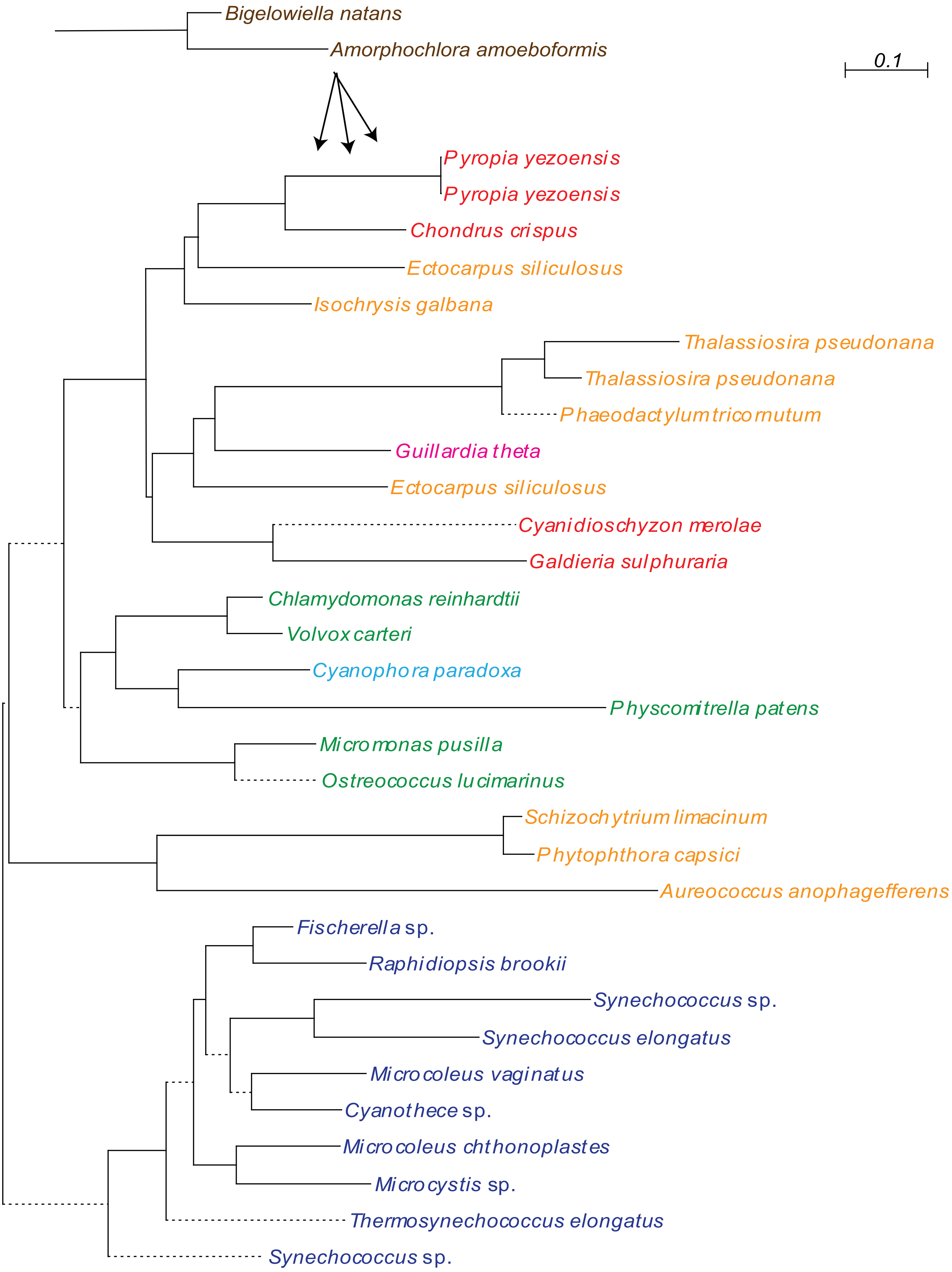

Figure S12 PGK

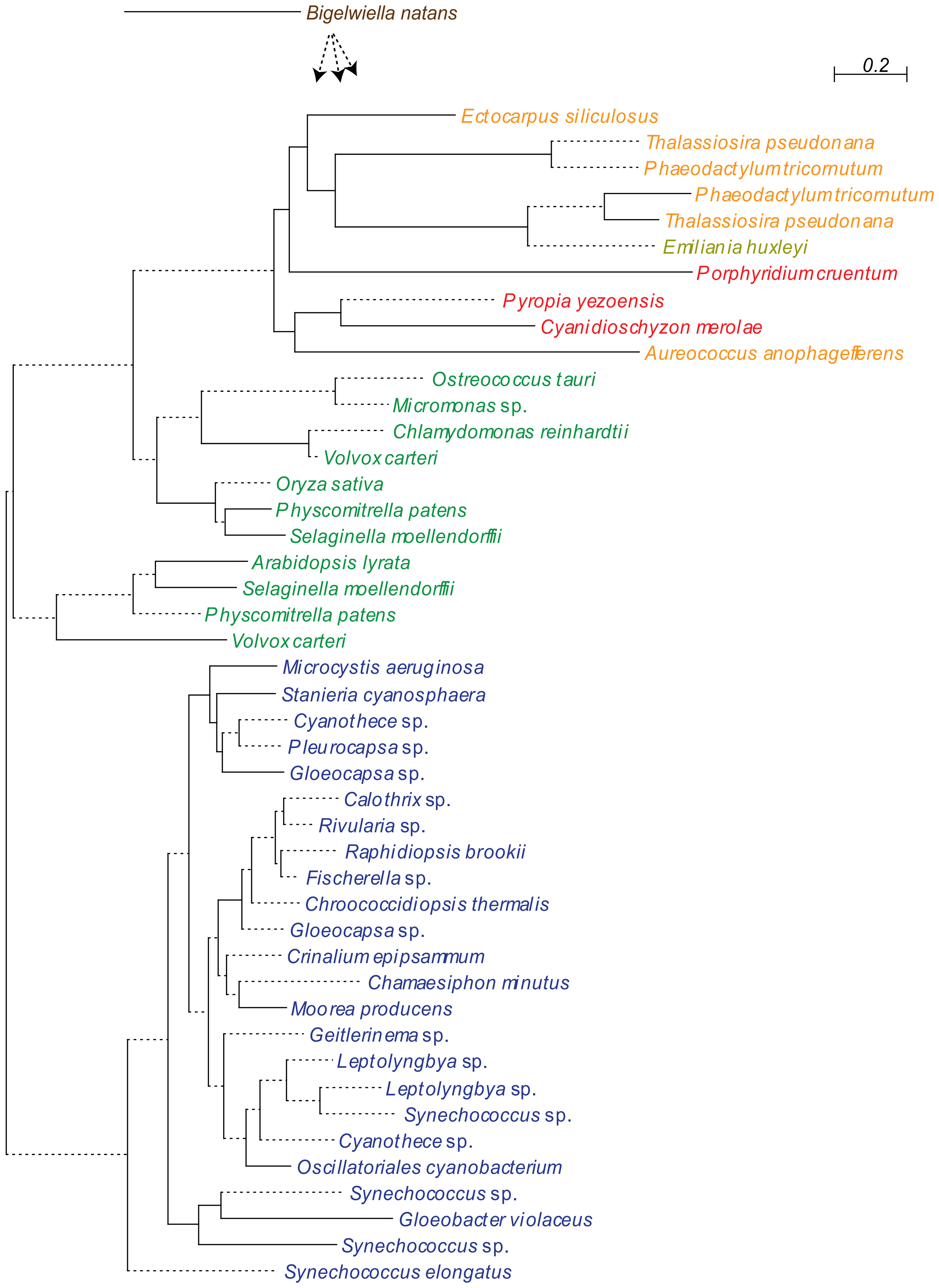

Figure S13 ABC

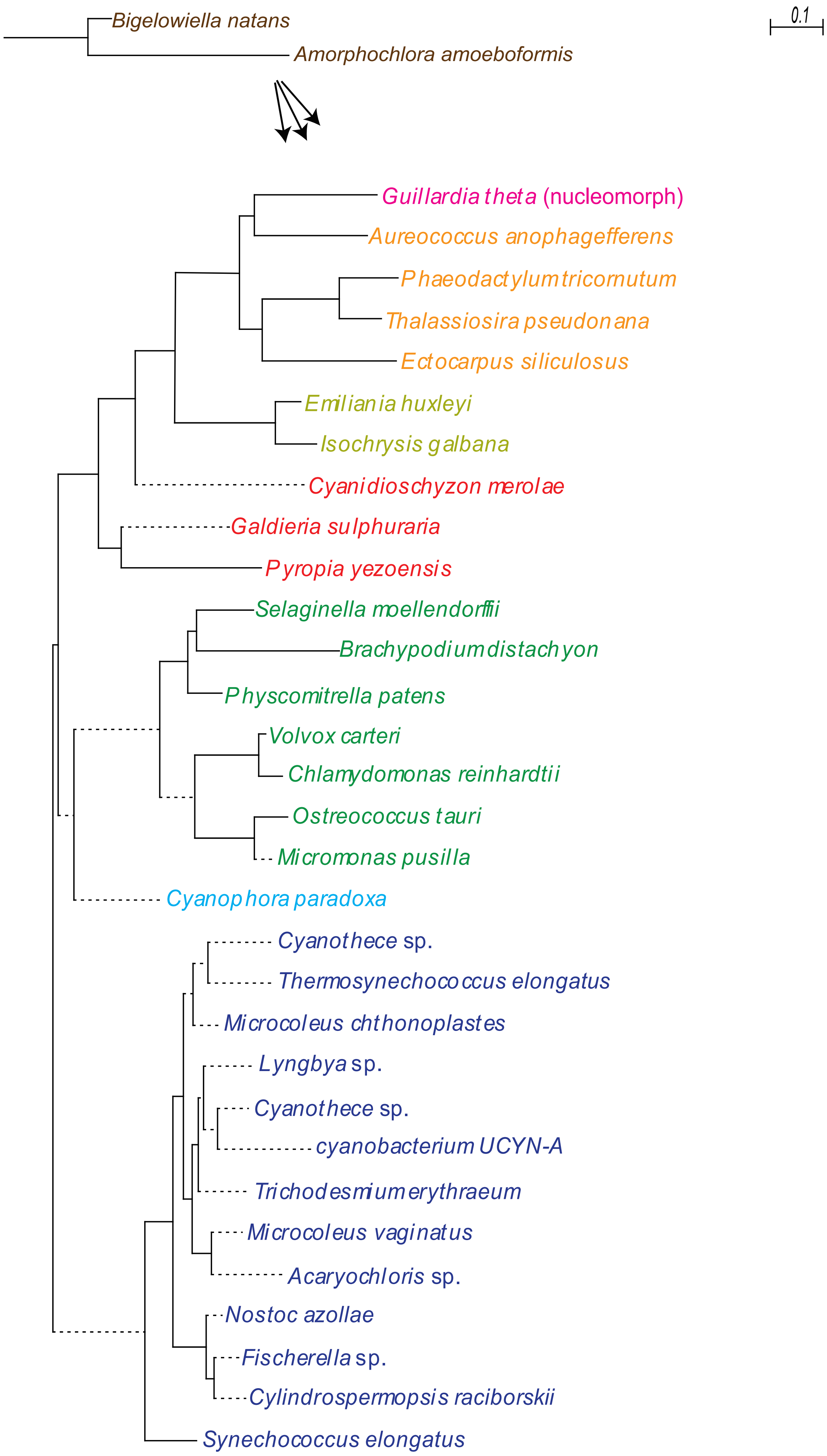

Figure S14 GGR

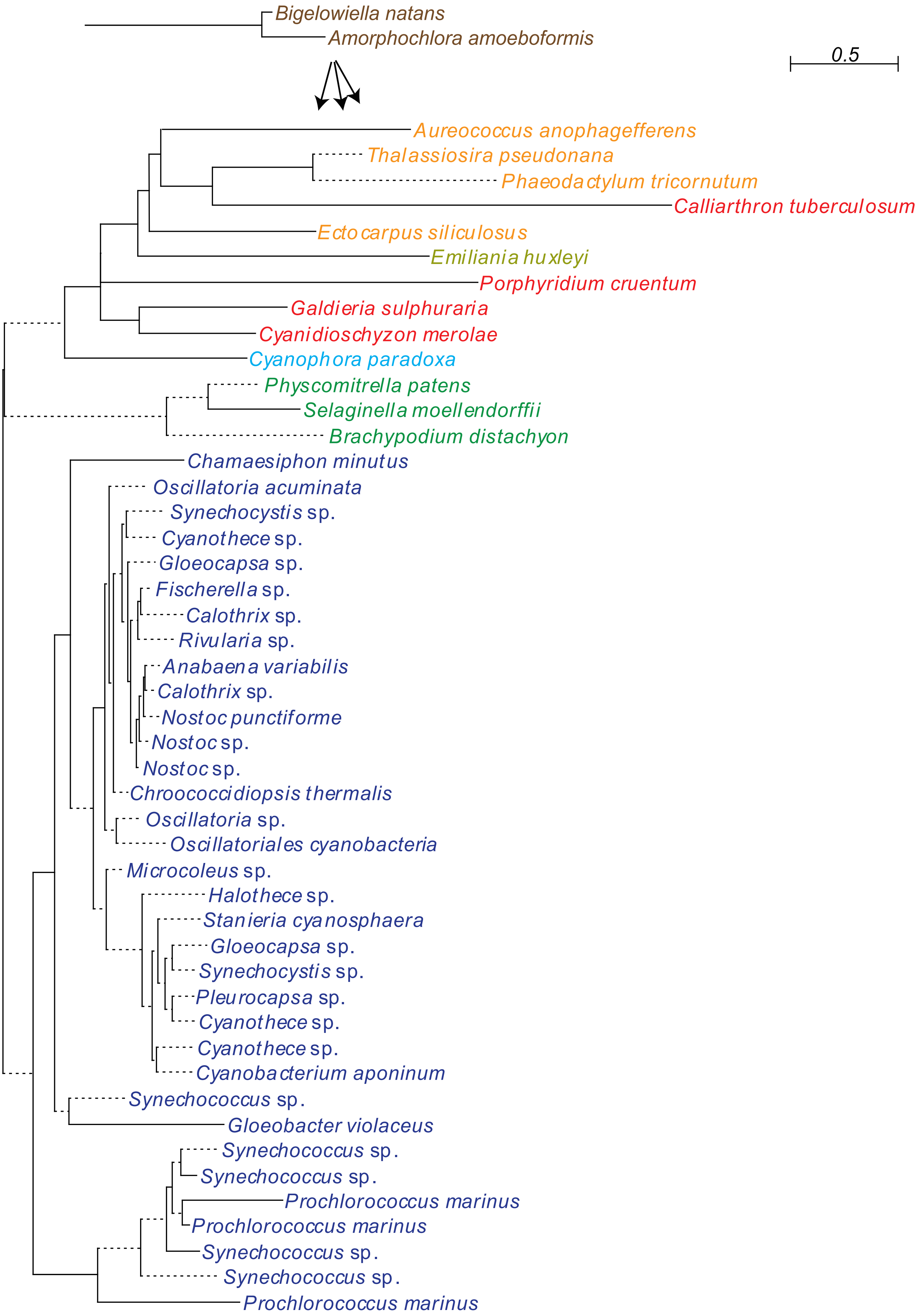

Figure S15 RPS22

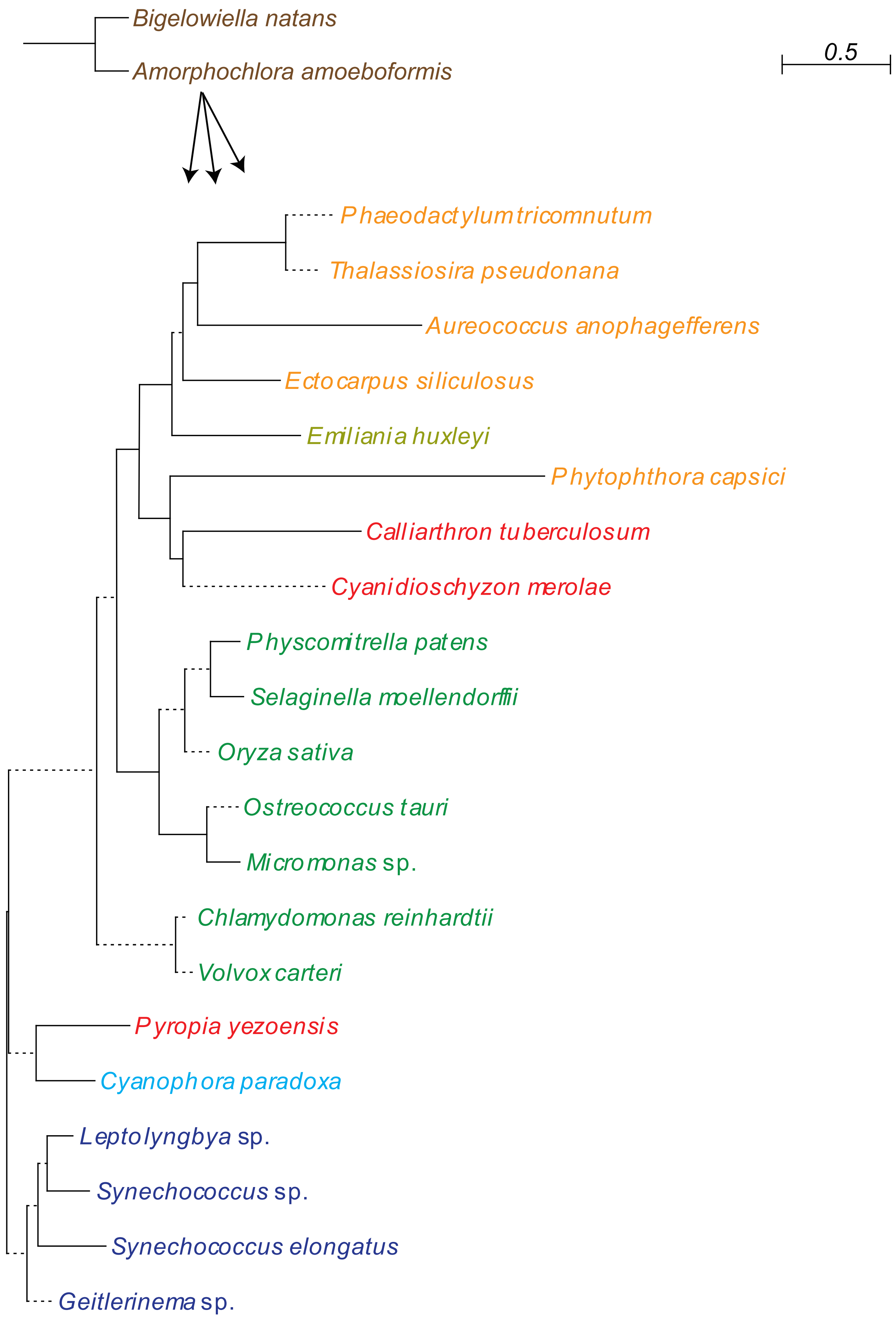

Figure S16 RNABP

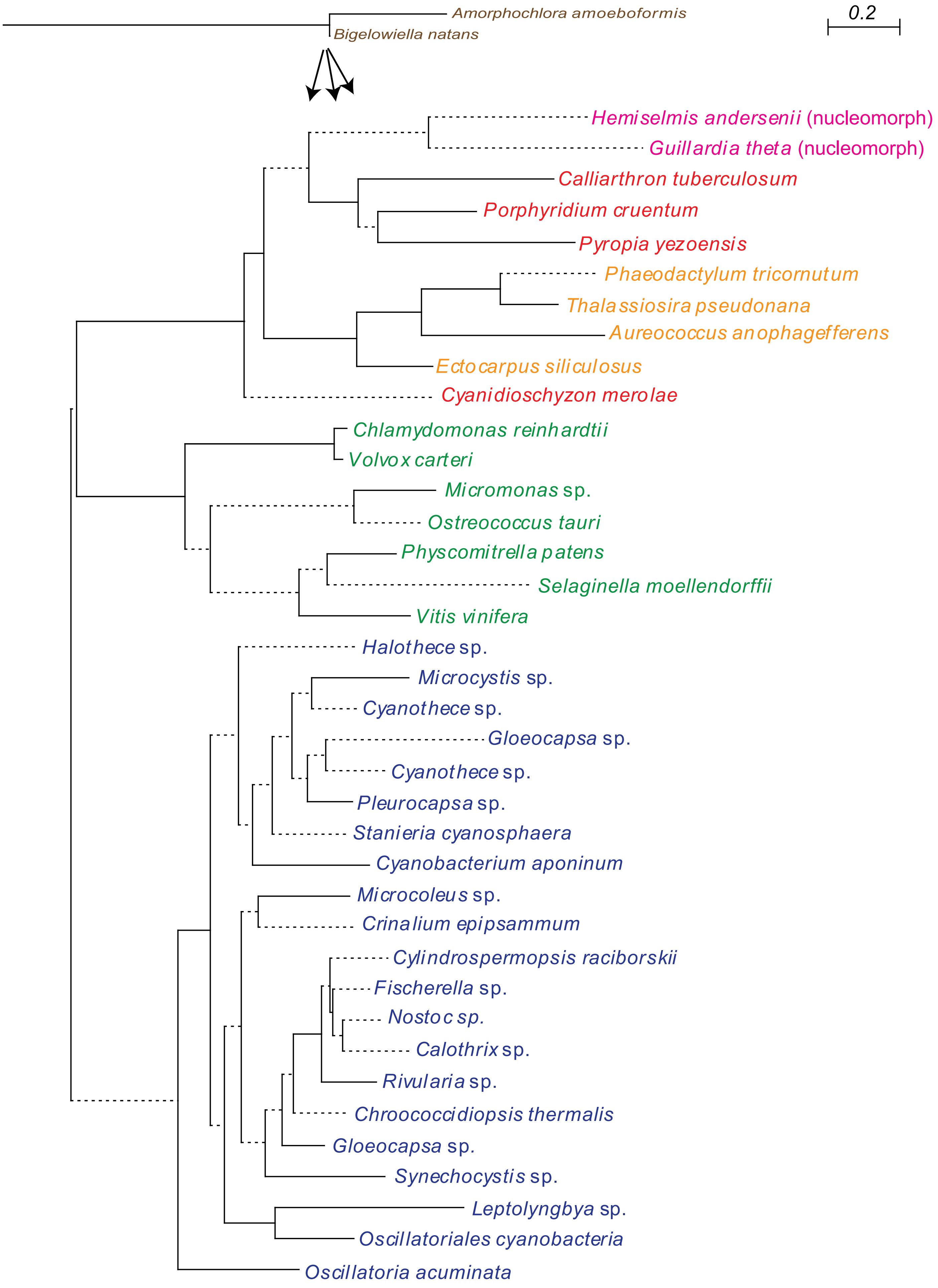

Figure S17 HP

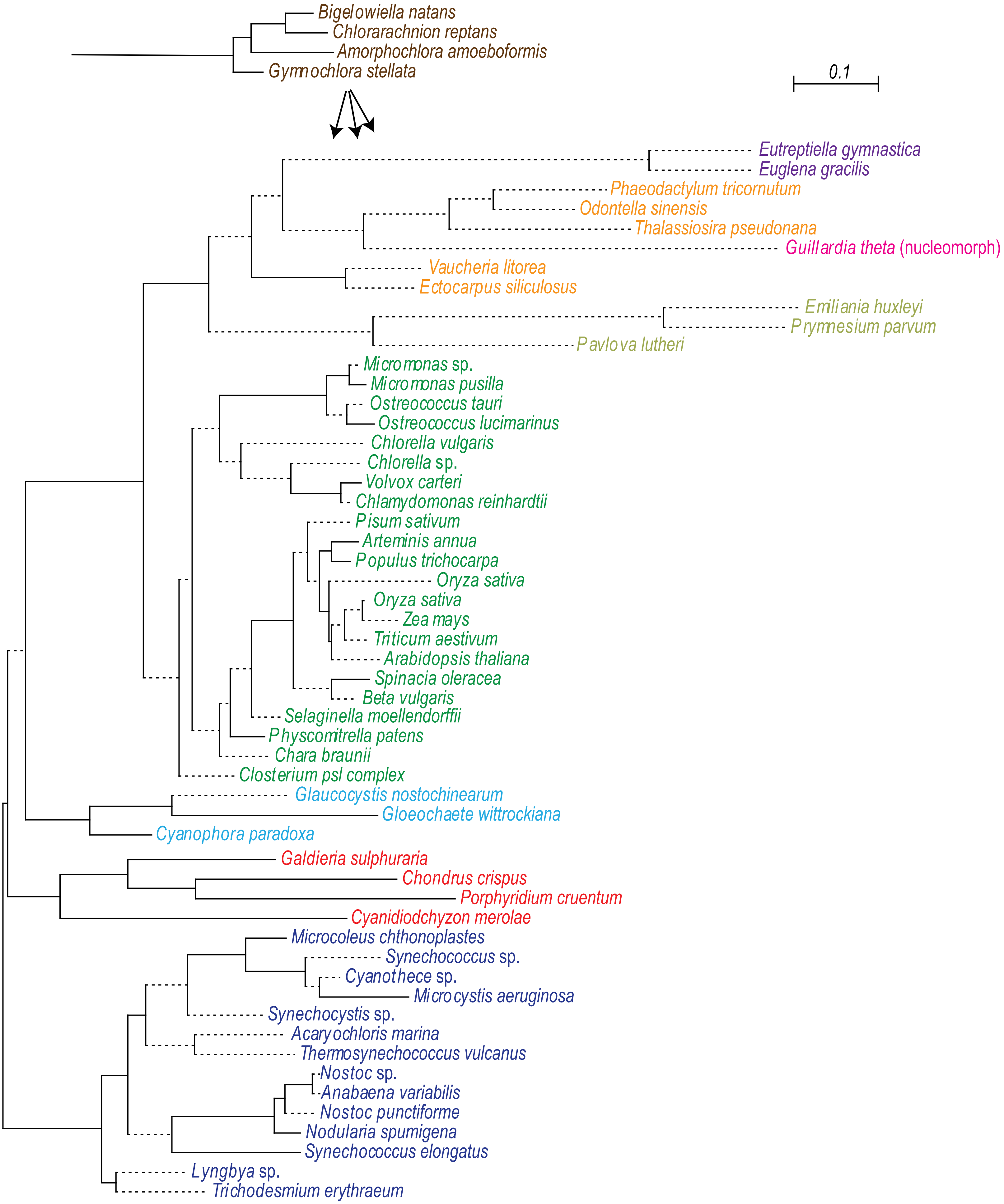

Figure S18 PRK
